# Palladium carbene complexes as persistent radicals†
†Electronic supplementary information (ESI) available: Characterization data for all new compounds, computational results, single crystal X-ray structure analysis of complexes {**2**}_2_, **3–6**, **8**, **9**, **11**, **12**. CCDC 1002269, 1058170–1058177. For ESI and crystallographic data in CIF or other electronic format see DOI: 10.1039/c5sc01441g



**DOI:** 10.1039/c5sc01441g

**Published:** 2015-05-18

**Authors:** C. C. Comanescu, M. Vyushkova, V. M. Iluc

**Affiliations:** a Department of Chemistry and Biochemistry , University of Notre Dame , Notre Dame , IN 46556 , USA . Email: viluc@nd.edu; b Notre Dame Radiation Laboratory , University of Notre Dame , Notre Dame , IN 46556 , USA

## Abstract

A series of palladium(ii) radical carbene complexes, [PC˙(sp^2^)P]PdI, [PC˙(sp^2^)P]PdBr, and [PC˙(sp^2^)P]PdCl (PC(sp^3^)H_2_P = bis[2-(di-iso-propylphosphino)-phenyl]methane), is described. Compound [PC˙(sp^2^)P]PdI dimerizes to {[PC(sp^2^)P]PdI}_2_ in the solid state, akin to the formation of Gomberg's dimer. While the bromo and the iodo derivatives could be obtained from the oxidation of [PC(sp^2^)P]Pd(PMe_3_) by the respective dihalogens, a halogen transfer reaction from CH_2_Cl_2_ was used for the formation of [PC˙(sp^2^)P]PdCl. The halogen transfer from CH_2_X_2_ (X = Cl, Br, I) could be used to obtain all three radical carbene palladium complexes and also allowed the isolation of [PC(CH_2_)P]Pd(PMe_3_), which is the result of methylene group transfer from CH_2_X_2_. Compound [PC(CH_2_)P]Pd(PMe_3_) was independently synthesized from [PC(CH_3_)HP]PdCl_2_, which contains a supporting ligand analogous to that of the radical carbene complexes but has one of the hydrogen atoms replaced by a methyl group. All three carbene radical species abstract a hydrogen from 9,10-dihydroanthracene or ^*n*^Bu_3_SnH.

## Abstract 2

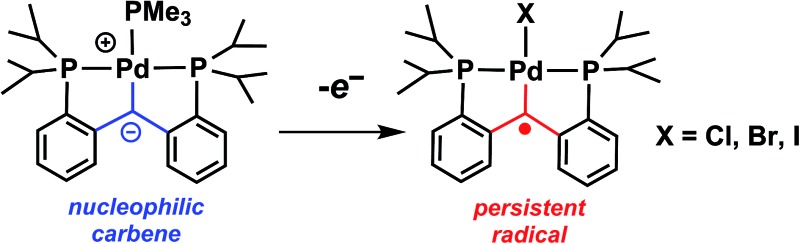
A series of palladium(ii) persistent radical carbene complexes, [PC˙(sp^2^)P]PdX (X = Cl, Br, I), was synthesized from the nucleophilic carbene [PC(sp^2^)P]PdPMe_3_.

## Introduction

Radicals have long fascinated scientists in general and chemists in particular.^1^ Controlling the reactivity of such species has been challenging but rewarding; the isolation of stable radicals opens new avenues for finding interesting reactions.^2^ One way to control these species is by coordination to transition metals, which can impart selectivity to the reactions of these radicals *via* metal control and/or auxiliary ligands.^3^

Although transition metal carbene complexes, which can display electrophilic or nucleophilic character, have been studied for some time,^4^ the corresponding radical species have been known mostly for electrophilic carbenes (Fischer type) of late transition metals. They are obtained by the reduction of the corresponding complexes,^5^ and are mostly observed as transient species with intricate reactivity.^6^ However, these radicals are very reactive and their characterization proved to be challenging.^5a,7^ A few examples of two-coordinate metal complexes containing cyclic alkylamino carbene ligands with singlet biradicaloid character were recently reported.^8^ Herein, we report the synthesis of such a series, *i.e.*, palladium(ii) carbenes as persistent radicals originating from a nucleophilic carbene.

## Results and discussion

### Synthesis and characterization of palladium radical carbene complexes

**Scheme 1:**
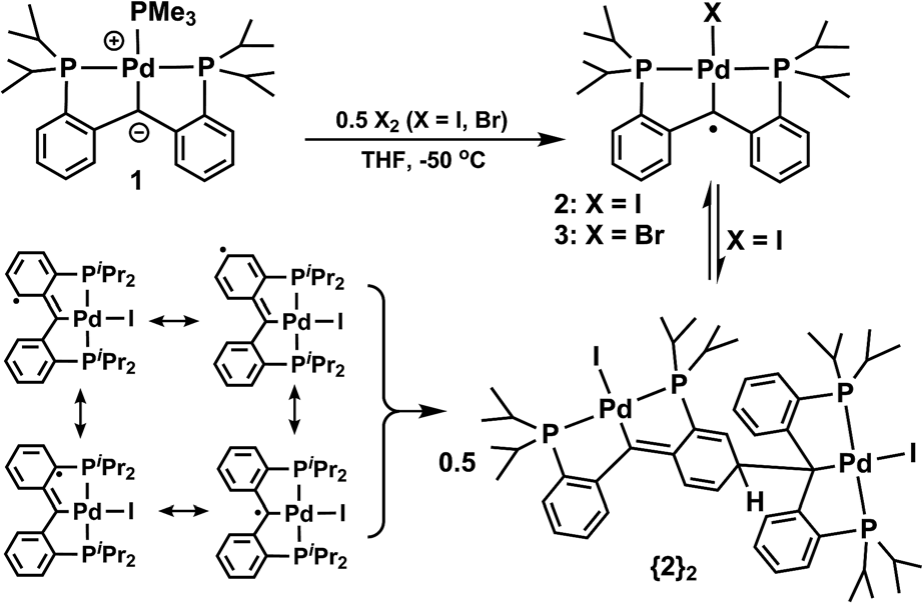
Synthesis of radical carbene palladium complexes.

**Fig. 1:**
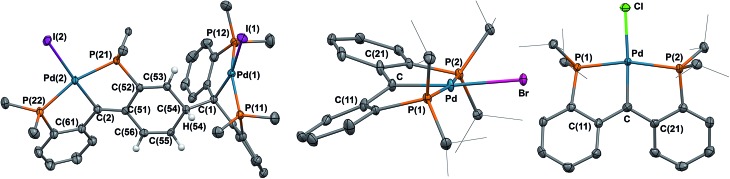
Molecular structure of {**2**}_2_, **3**, and **5** with thermal ellipsoids at 50% probability. Most hydrogen atoms were omitted for clarity. Selected distances (Å) and angles (°) for {**2**}_2_: Pd(1)–C(1) = 2.127(3), Pd(2)–C(2) = 2.043(3), C(2)–C(51) = 1.380(4), C(2)–C(61) = 1.481(4); for **3**: Pd–C = 2.020(3), Pd–Br = 2.5117(4), C(11)–C–C(21) = 123.0(3), Pd–C–C(11) = 118.1(2), Pd–C–C(21) = 118.7(2); for **5**: Pd–C = 2.005(2), Pd–Cl = 2.3884(6), C(11)–C–C(21) = 122.2(2), Pd–C–C(11) = 118.78(17), Pd–C–C(21) = 118.87(17).

We previously established that the carbene carbon in [PC(sp^2^)P]Pd(PMe_3_) (**1**, PC(sp^3^)H_2_P = bis[2-(di-iso-propylphosphino)-phenyl]methane)^9^ has nucleophilic character.^10^ DFT calculations indicated that the HOMO of **1** is localized on the carbene carbon atom, therefore, the loss of an electron might occur from the same orbital. Compound **1** shows a reversible oxidation wave at –0.15 V *vs.* Cp_2_Fe/Cp_2_Fe^+^ by cyclic voltammetry (ESI: Fig. S2†). Although the chemical oxidation of **1** with [Cp_2_Fe]^+^ did not result in an isolable product, the analogous reaction with I_2_ (Scheme 1) allowed the observation in solution of a green paramagnetic species, [PC˙(sp^2^)P]PdI (**2**), in good yield (70%). Compound **2** represents, to the best of our knowledge, the first example of a palladium radical carbene complex. The radical nature of **2** in solution is supported by the value of the magnetic moment of 1.76 *μ*_B_, corresponding to one unpaired electron. Interestingly, **2** dimerizes in the solid state to form {**2**}_2_ (Fig. 1), the result of radical coupling.

The formation of {**2**}_2_ from **2** (Scheme 1) is analogous to the formation of Gomberg's dimer.^11^ Metrical parameters for {**2**}_2_ agree with this interpretation. For example, the C–C distances C(51)–C(52), C(52)–C(53), C(53)–C(54), C(54)–C(55), C(55)–C(56), and C(56)–C(51) of 1.465(4), 1.345(4), 1.496(4), 1.508(4), 1.339(4), and 1.465(4) Å, respectively, in the dearomatized phenyl ring show bond alternation. In addition, the C(2)–C(51) distance of 1.380(4) Å indicates double bond character, while the C(2)–C(61) distance of 1.481(4) Å is consistent with a single bond. Moreover the C(1)–C(54) distance of 1.588(4) Å indicates an elongated C–C bond, in agreement with a weak interaction between the two monomers in the solid state. The Pd(1)–C(1) distance of 2.127(3) Å is slightly longer than the corresponding value in **1** (2.086(4) Å). To investigate the dimerization process further, a variable temperature magnetization study in solution indicated that by lowering the temperature, the magnetic moment of **2** decreases from 1.76*μ*_B_ at room temperature to 1.39*μ*_B_ at 220 K, in agreement with the formation of the dimer at lower temperatures.

The dimerization of **2** is the consequence of radical coupling of one of its resonance structure (radical on the *para* position of the phenyl ring, Scheme 1). The resonance structures possible for **2** show that the radical creates an increased electron density on the *ortho* and *para* positions of the phenyl ring; both the central carbene carbon and the two *ortho* positions are sterically hindered, therefore, the contribution of the negatively charged *para* resonance structure is significant in determining the coupling position.

We also pursued the synthesis of the chloro and bromo analogues of **2**. In a similar manner, the reaction between **1** and Br_2_ (Scheme 1) generated a new paramagnetic species, [PC˙(sp^2^)P]PdBr (**3**). In the reaction mixture we also observed [PC(sp^3^)HP]PdBr (**4**), likely due to the presence of small amounts of HBr in Br_2_. We previously reported the protonation of carbene **1** with HCl^10*a*^ and, in a similar reaction, the formation of [PC(sp^3^)HP]PdBr (**4**) could be accomplished from **1** and HBr. Interestingly, in this case, the radical carbene **3** is monomeric in both solution and the solid state. The solution magnetic moment (2.19*μ*_B_) confirms a one electron radical species. While mononuclear Pd(iii) complexes are known,^12^ in this case the oxidation takes place on the ligand, similar to the oxidation of PNP or ^Me^PNP (PNP = (*o*-P^i^Pr_2_-C_6_H_4_)_2_N; ^Me^PNP = (2-P^i^Pr_2_-4-MeC_6_H_3_)_2_N) complexes that generate a nitrogen-based radical.^13^ In the solid state (Fig. 1), the Pd(ii) metal center is distorted square planar, with a Pd–C distance of 2.020(3) Å, slightly shorter than that observed for **1** (2.086(4) Å) or [PC(sp^3^)HP]PdBr (**4**, 2.071(4) Å). The carbene carbon is planar with a sum of angles of 359.8°.

**Scheme 2:**
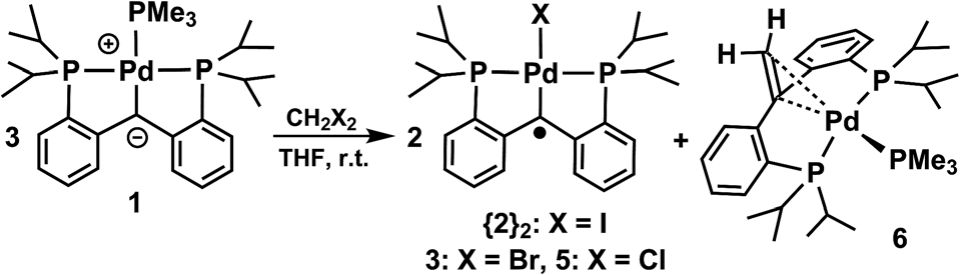
Reactions of **1** with dihalogenomethanes.

The last complex of the series, the chloro derivative, [PC˙(sp^2^)P]PdCl (**5**) was synthesized by halogen atom abstraction from dichloromethane in an analogous manner with the synthesis of [^F^(PNP)PtCl][BAr^F^_4_] (^F^(PNP) = (4-F-2-(^i^Pr_2_P)C_6_H_3_)_2_N; Ar^F^ = 3,5-(CF_3_)_2_C_6_H_3_) to generate a nitrogen based radical on the PNP ligand.^14^ The reaction proceeds slowly at room temperature and, after 2.5 days, the product was obtained in 75% of the theoretical yield (Scheme 2).

Similarly to the bromo derivative, the chloro substituted complex **5** is monomeric in both solution (1.86*μ*_B_) and the solid state. The molecular structure of **5** (Fig. 1) indicates that the carbene carbon is planar and found at 2.005(2) Å from the metal center. Like the bromo and the iodo derivatives, **5** is best described as a Pd(ii) metal center attached to a radical carbon, interpretation supported by DFT calculations (see below and ESI: Fig. S27†).

**Fig. 2:**
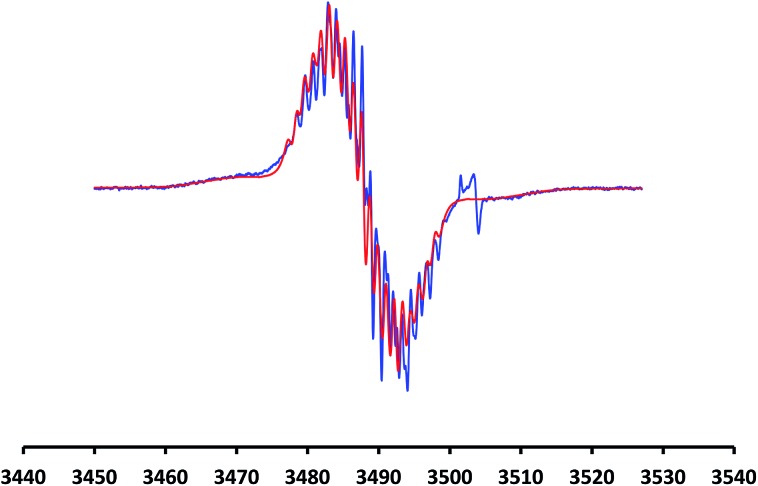
EPR spectrum for **5** (298 K, 100 μM solution in toluene, X-band). The blue line represents the experimental data and the red line the simulated spectrum. The small satellite peak on the right comes from the quartz sample tube.

EPR spectroscopy indicates that all three radical species, [PC˙(sp^2^)P]PdI (**2**), [PC˙(sp^2^)P]PdBr (**3**), and [PC˙(sp^2^)P]PdCl (**5**) display *g*-factors close to 2, supporting the radical state of the backbone.^5b,15^ However, the *g*-factor increases slightly from **5** (*g* = 2.0100) to **3** (*g* = 2.0105), and to **2** (*g* = 2.0110). No hyperfine structure was resolved for [PC˙(sp^2^)P]PdBr and [PC˙(sp^2^)P]PdI, but dilute solutions of [PC˙(sp^2^)P]PdCl display a well-resolved hyperfine splitting (Fig. 2) attributed to 8 phenyl ring protons: *a*_1_(2H) = 4.5 G; *a*_2_(2H) = 2.6 G; *a*_3_(2H) = 2.1 G; *a*_4_(2H) = 1.2 G. The computed hyperfine interactions for **5**, *a*(2H, 4,4′) = 4.54 G; *a*(2H, 6,6′) = 4.18 G; *a*(2H, 5,5′) = 2.00 G; *a*(2H, 3,3′) = 1.90 G, are in agreement with experimental values. Hyperfine coupling to ^105^Pd nucleus (nuclear spin 5/2, natural abundance 22.33%) gives rise to broad satellites on either side of the central multiplet (Fig. 2). In **2** and **3**, hyperfine coupling to ring protons contributes to the inhomogeneous line width (ESI: Fig. S3–S5† for details).

The halogen atom transfer reaction from CH_2_X_2_ to **1** also proved a good way to synthesize {**2**}_2_ (X = I) and **3** (X = Br). In all cases, the identity of the paramagnetic species was confirmed by X-ray crystallography, solution magnetic moment, and subsequent reactivity studies (see below). A new diamagnetic complex was observed in all three crude reaction mixtures (Scheme 2). The corresponding ^1^H NMR spectra show a new resonance as a doublet at 3.81 ppm in the olefinic region, while the ^31^P NMR spectra display an AX_2_ spin system, *δ* (A) is –31.55 ppm (t, ^2^ *J*_PP_ = 21.6 Hz) and *δ* (X_2_) is 33.85 ppm (d, ^2^ *J*_PP_ = 21.7 Hz), consistent with the presence of PMe_3_ in the molecule. The ^13^C NMR spectra show the backbone carbon atom resonating at 112.16 ppm as a doublet of triplets, due to coupling to both types of phosphorus nuclei present in the molecule (*J*_CP_ = 17.6 Hz, *J*_CP_ = 2.3 Hz), and a methylenic carbon is found at 64.4 ppm as a triplet of doublets due to long range phosphorus coupling (*J*_CP_ = 7.5 Hz, *J*_CP_ = 5.1 Hz). X-ray crystallography indicates that the new product, [PC(CH_2_)P]Pd(PMe_3_) (**6**), is the result of coupling of the CH_2_ group of dihalogenomethane with the carbene carbon of **1** to generate a new carbon–carbon double bond. The C=C bond formation in **6** is somewhat reminiscent of the formation of the C=O bond in the iridaepoxide [P′C(O)P′]IrCl (P′C(sp^3^)H_2_P′ = bis[2-(di-iso-propylphosphino)-benzothiophene]methane), isolated from the reaction of the iridium PC_carbene_P complex [P'C_carbene_P′]IrCl and N_2_O.^16^

To the best of our knowledge, a similar “CH_2_” transfer reaction as that described above has not been previously characterized, although examples of nickel^17^ or iron^18^ catalysed cross-coupling reactions of CH_2_Cl_2_ with Grignard reagents are known. It has also been reported that Kharasch addition reactions of perhalogenated reagents to olefins involve Pd(0)/Pd(i) or Pd(ii)/Pd(iii) oxidations and halogen transfer, but the intermediate metal species have not been characterized.^19^ It is important to note that in the reactions of **1** with CH_2_X_2_ (X = Cl, Br, I), palladium is not oxidized and the electron transfer takes place at the carbene ligand.

**Fig. 3:**
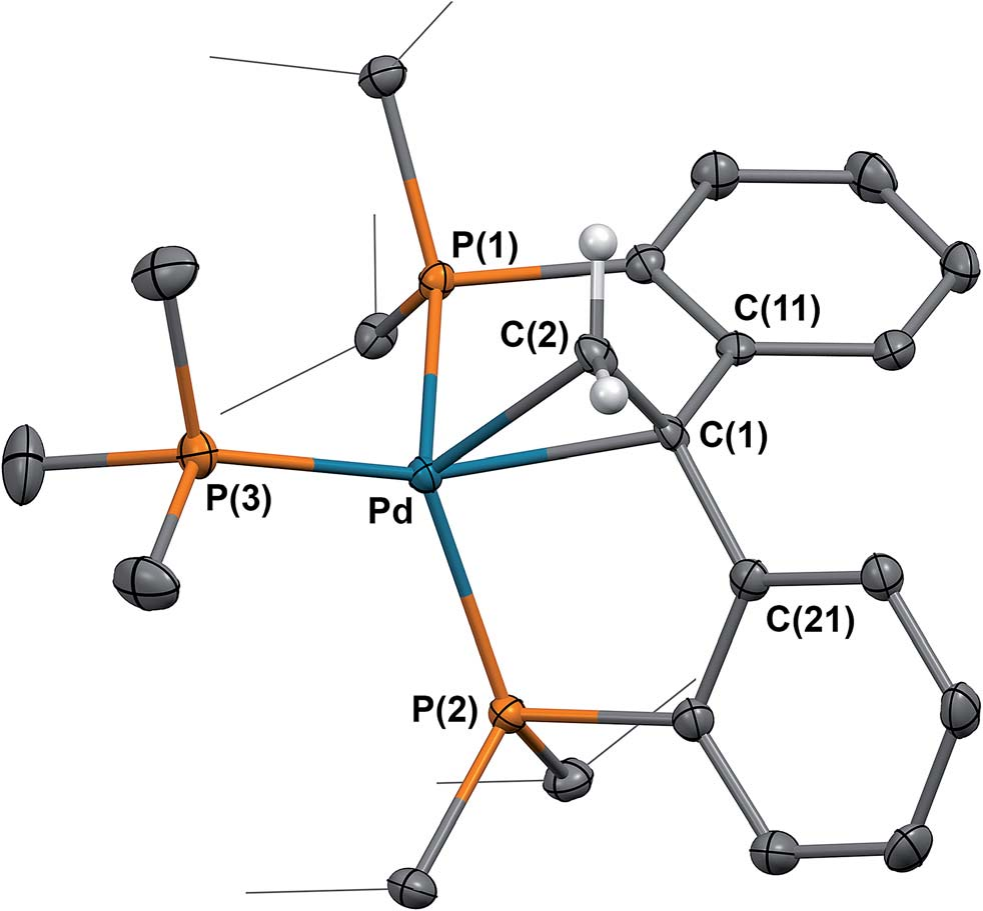
Molecular structure of **6** with thermal ellipsoids at 50% probability. Most hydrogen atoms were omitted for clarity. Selected distances (Å) and angles (°): C(1)–C(2) = 1.398(3), Pd–C(1) = 2.2242(18), Pd–C(2) = 2.2613(19), C(1)–Pd–C(2) = 36.30(7).

In **6** (Fig. 3), the metal centre is coordinated in a side-bound fashion to the new C=C double bond.^20^ The metrical parameters for **6** point to a distorted tetrahedral palladium(0) metal center (P(1)–Pd–P(3) = 109.332(19)°, P(2)–Pd–P(3) = 118.946(19)°, P(1)–Pd–P(2) = 115.868(19)°). The C(1)–C(2) distance of 1.398(3) Å is slightly longer than 1.34 Å (C(sp^2^)–C(sp^2^)), likely due to π-backbonding.

**Scheme 3:**
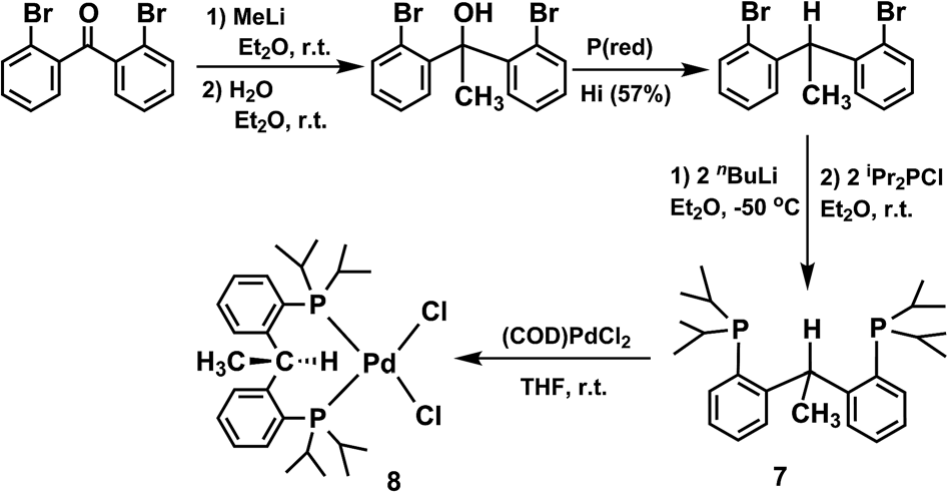
Synthesis of PC(CH_3_)HP (**7**) and [PC(CH_3_)HP]PdCl_2_ (**8**).

The formation of **6** in the above reactions (Scheme 2) occurs in a relatively low yield since two thirds of **1** convert to the radical species, while the rest converts to **6** (75%, 38%, and 64% conversion to **6** observed besides the formation of {**2**}_2_, **3**, and **5**, respectively, isolated yield). Therefore, an independent synthesis of compound **6** was designed. We reasoned that the deprotonation of a methyl group that is a substituent of the carbon atom that connects the two phosphine phenyl rings in [PC(CH_3_)HP] (**7**) would lead to the isolation of **6**. Compound **7** was synthesized in three steps from bis(2-bromophenyl)methanone (Scheme 3). Reaction of bis(2-bromophenyl)methanone with methyl lithium led to the isolation of the 1,1-bis(2-bromophenyl)ethan-1-ol. Reduction of this carbinol in the presence of red phosphorous and hydroiodic acid generated 1,1-bis(2-bromophenyl)ethane, the precursor for **7**. [PC(CH_3_)HP] (**7**) was synthesized from this precursor by double lithiation with ^*n*^BuLi followed by metathesis with ^i^Pr_2_PCl in 75% yield, as a clear oil. From **7**, a palladium(ii) complex could be isolated in 76% yield by mixing (COD)PdCl_2_ and [PC(CH_3_)HP] at room temperature in THF to give [PC(CH_3_)HP]PdCl_2_ (**8**).

**Scheme 4:**
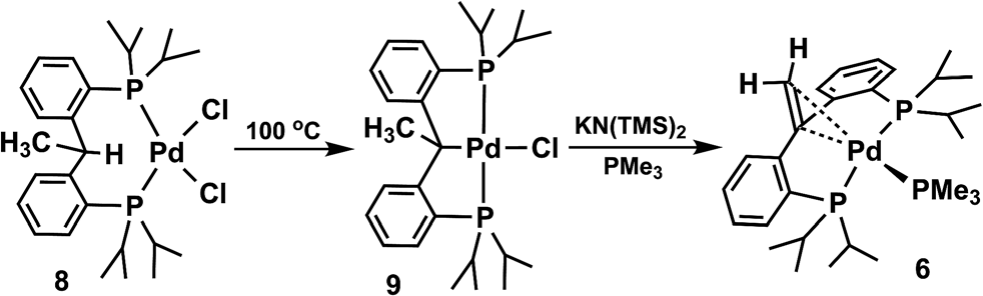
Independent synthesis of **6**.

Heating [PC(CH_3_)HP]PdCl_2_ (**8**) at 100 °C in toluene resulted in the C–H activation of the backbone (Scheme 4); a subsequent dehydrohalogenation generates the square planar complex [PC(CH_3_)P]PdCl (**9**).^10*a*^ A second dehydrohalogenation, using KN(SiMe_3_)_2_ in the presence of PMe_3_, achieved the second C–H activation, now at the methyl group, and led to the isolation of **6** (Scheme 4) in high yield (75%).

### DFT calculations

DFT calculations were carried out using Gaussian09 on model complexes of the three carbene radical species, **2′**, **3′**, and **5′**, in which the iso-propyls on phosphines were replaced by methyl groups. Geometry optimization results indicate a good agreement between the calculated structures of **3′** and **5′** and the corresponding experimental structures (Table 1).

**Table 1:** Selected metrical parameters for the calculated and experimental structures of **3** and **5**

	**3′** (DFT)	**3** (X-ray)	**5′** (DFT)	**5** (X-ray)
Pd–C (Å)	2.047	2.020(3)	2.042	2.005(2)
Pd–X (Å)	2.604	2.5117(4)	2.463	2.3884(6)

**Fig. 4:**
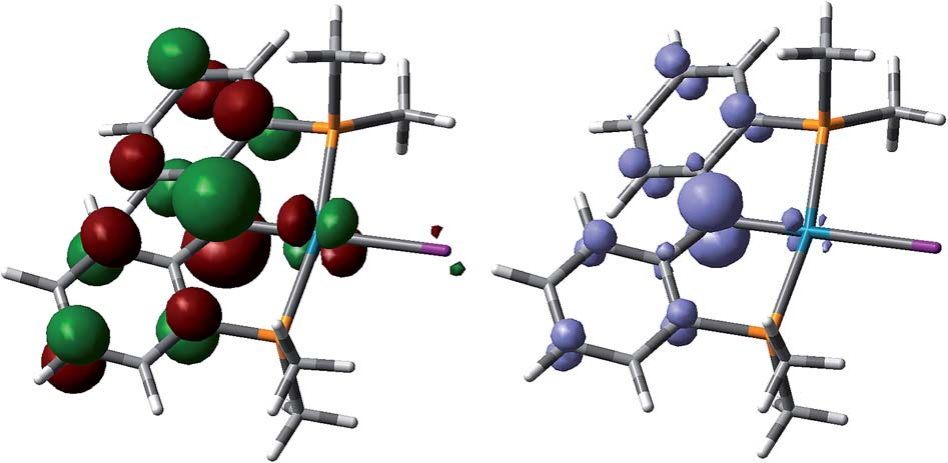
Left: SOMO for **2′**; right: spin density for **2′**.

DFT calculations indicate that the unpaired electron is localized mostly on the carbene carbon atom and slightly delocalized over the two phenyl rings in **2′**, **3′**, and **5′** (Fig. 4). These results together with the fact that *ca.* 64% of the spin density was found on the former carbene carbon atom for all three radical carbene complexes support the interpretation that most of the spin density rests on this atom (Fig. 4). Furthermore, the composition of the SOMO for the three radical species indicates that 63.9%, 63.7%, and 63.4% of the p orbital of the carbon atom and 2.2%, 2.3%, and 2.4% of the palladium d orbital contribute in **2′**, **3′**, and **5′**, respectively. Since the three radical species are formed by the oxidation of **1** and the electron is removed from an antibonding orbital, the order of the Pd–C bond increases for **2′**, **3′**, and **5′**, respectively, as also shown by the decrease of the Pd–C distance in the radical species (see above).

In agreement with experimental results, the corresponding coupling product {**2**}_2_ is less stable than **2** by 4.96 kcal mol^–1^, consistent with the observation of the monomeric species in solution. Consequently, the difference between the respective dimers, {**3**}_2_ and {**5**}_2_, and the radical species increases from the iodo to the bromo (6.45 kcal mol^–1^) and chloro (8.03 kcal mol^–1^) derivative. The fact that **3** and **5** do not dimerize in the solid state is also supported by a slight decrease of the spin density on the *para* carbon involved in the coupling reaction (18.23% in **2**, 18.06% in **3**, and 18.03% in **5**).

### Reactivity studies of palladium radical carbene complexes

**Scheme 5:**
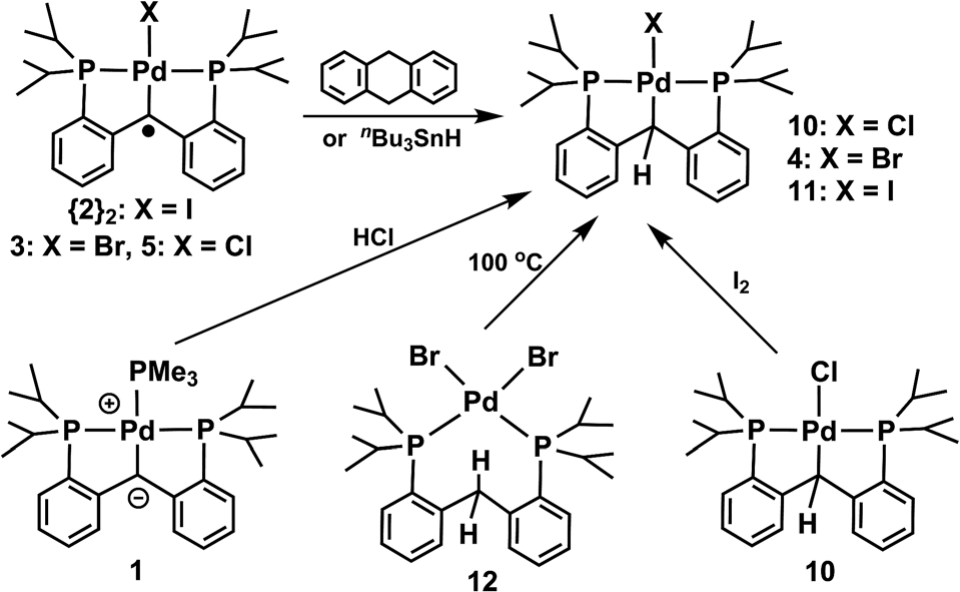
Reactions of the radical carbene palladium complexes with hydrogen donors and alternate synthesis of these products.

The radical nature of all three carbene radical species was probed by their reactions with hydrogen atom donors, 9,10-dihydroanthracene and ^*n*^Bu_3_SnH (Scheme 5). In all cases, the reactions were slow and it took a few hours in order to achieve moderate conversions; consequently, the isolated yields were relatively low: 14% for **10**, 16% for **4**, and 17% for **11** for the reaction with 9,10-dihydroanthracene and 48% for **10**, 24% for **4**, and 78% for **11** for the reaction with ^*n*^Bu_3_SnH. In order to confirm the identity of the respective products of these reactions, [PC(sp^3^)HP]PdCl (**10**),^10*a*^ [PC(sp^3^)HP]PdBr (**4**), and [PC(sp^3^)HP]PdI (**11**) were compared with samples synthesized by independent methods (Scheme 5). Compound [PC(sp^3^)HP]PdCl (**10**) was previously reported.^10*a*^ Compound [PC(sp^3^)HP]PdBr (**4**) was synthesized using a method analogous to that used for the synthesis of [PC(sp^3^)HP]PdCl: [PC(sp^3^)H_2_P]PdBr_2_ (**12**) could be isolated by reacting PC(sp^3^)H_2_P (**13**) with (COD)PdBr_2_ at ambient temperature^21^ in 91% yield. This complex undergoes dehydrohalogenation through ligand C–H activation by heating it at 100 °C in toluene to generate [PC(sp^3^)HP]PdBr (**4**) in high yield (83%). On the other hand, compound [PC(sp^3^)HP]PdI was synthesized from the reaction of [PC(sp^3^)HP]PdCl with one equivalent of I_2_ in THF. A usual workup with Et_2_O allowed the isolation of **11** as light yellow crystals in 68% yield.

**Scheme 6:**
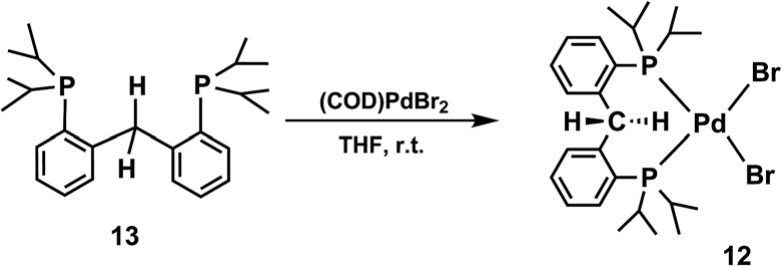
Synthesis of [PC(sp^3^)H_2_P]PdBr_2_ (**12**).

## Conclusions

In conclusion, we described the formation of a series of palladium(ii) radical carbene complexes, [PC˙(sp^2^)P]PdI (**2**), [PC˙(sp^2^)P]PdBr (**3**), and [PC˙(sp^2^)P]PdCl (**5**). These radical species are persistent in solution and, for **3** and **5**, also in the solid state as indicated by X-ray crystallography. Metrical parameters for **3** and **5** indicate that Pd–C distances are slightly shorter than the corresponding values observed for [PC(sp^2^)P]Pd(PMe_3_) (**1**) and [PC(sp^3^)HP]PdX (X = Cl, Br). The carbene carbon is planar and palladium shows a distorted square planar geometry in both metal complexes. Compound **2** dimerizes in the solid state to {**2**}_2_, akin to the formation of Gomberg's dimer.

While **2** and **3** could be obtained from the oxidation of [PC(sp^2^)P]Pd(PMe_3_) by the respective dihalogens, a halogen transfer reaction from CH_2_Cl_2_ was used for the formation of **5**. The halogen transfer from CH_2_Br_2_ and CH_2_I_2_ also led to the isolation of the corresponding radical carbene palladium complexes; in addition, this reaction allowed the isolation of [PC(CH_2_)P]Pd(PMe_3_) (**6**), the result of methylene group transfer. Compound **6** was independently synthesized from [PC(CH_3_)HP]PdCl_2_, which contains a supporting ligand analogous to that of the radical carbene complexes but has one of the hydrogen atoms replaced by a methyl group.

The radical nature of the carbene carbon was also confirmed by the results of DFT calculations and EPR spectroscopy. All three radical species, [PC˙(sp^2^)P]PdI (**2**), [PC˙(sp^2^)P]PdBr (**3**), and [PC˙(sp^2^)P]PdCl (**5**), display *g* factors close to 2, supporting the radical state of the backbone. In addition, although no hyperfine structure was resolved for [PC˙(sp^2^)P]PdBr and [PC˙(sp^2^)P]PdI, dilute solutions of [PC˙(sp^2^)P]PdCl display a well-resolved hyperfine splitting attributed to 8 phenyl ring protons and ^105^Pd nucleus.

Reactivity studies with **2**, **3**, and **5** showed that all three compounds abstract a hydrogen from 9,10-dihydroanthracene or ^*n*^Bu_3_SnH supporting their radical nature. In addition to hydrogen-abstraction reactions, the radical carbene species discussed herein may be involved in redox reactions, conferring the supporting ligand a non-innocent behavior.^22^ We are currently exploring these possibilities.

## Experimental

All experiments are performed under an inert atmosphere of N_2_ using standard glovebox techniques. Solvents, hexane, *n*-pentane, CH_2_Cl_2_, and diethylether, were dried by passing through a column of activated alumina and stored in the glovebox. THF was dried over LiAlH_4_ followed by vacuum transfer and stored in the glovebox. Deuterated solvents, CDCl_3_ and CD_2_Cl_2_, were dried over 4 Å molecular sieves under N_2_, while C_6_D_6_ and C_6_D_5_CD_3_ were dried over CaH_2_ followed by vacuum transfer, and stored in the glovebox. Bis(2-bromophenyl)methanone,^23^ [PC(sp^2^)P]Pd(PMe_3_) (**1**),^10*a*^ [PC(sp^3^)HP]PdCl (**10**),^10*a*^ and PC(sp^3^)H_2_P (**13**)^9*b*^ were prepared according to literature procedures. All other materials were used as received. ^1^H, ^13^C{^1^H} and ^31^P{^1^H} NMR spectra were recorded on a Bruker DRX 400 or 500 spectrometer. All chemical shifts are reported in *δ* units with references to the residual solvent resonance of the deuterated solvents for proton and carbon chemical shifts or to external H_3_PO_4_ for ^31^P. Magnetic moments were determined by the Evans method^24^ using capillaries containing hexamethylsiloxane in C_6_D_6_ as a reference and hexamethylsiloxane in the sample solution. EPR spectra were recorded on a Bruker EMXplus EPR spectrometer with a standard X-band EMXplus resonator and an EMX premiumX microwave bridge. Electrochemical data was collected on a Metrohm Autolab PGSTAT-128N instrument. CHN analyses were performed on a CE-440 Elemental Analyzer or by Midwest Microlab, LLC. Gaussian 03 (revision D.02) was used for all reported calculations.^25^ The B3LYP (DFT) method was used to carry out the geometry optimizations on model compounds specified in text using the LANL2DZ basis set. The validity of the true minima was checked by the absence of negative frequencies in the energy Hessian.

### Synthesis of {[PC(sp^2^)P]PdI}_2_ ({**2**}_2_)

58.1 mg of [PC(sp^2^)P]Pd(PMe_3_) (**1**, 0.1 mmol) was dissolved in THF and cooled at –50 °C. Then, 0.5 mL of a chilled I_2_ solution in THF (0.1 M, 0.05 mmol, –50 °C) was added dropwise and a rapid colour change was observed from dark brown to very dark green-brown. The solution was allowed to react for two additional hours at –50 °C, then warmed up to room temperature and the volatiles were removed under reduced pressure. The residual powder was triturated with *n*-pentane. The residue was dissolved in Et_2_O, filtered through a Celite plug and set to crystallize at –35 °C in the glovebox to yield analytically pure {**2**}_2_ (44.2 mg, 70%). For {**2**}_2_: ^1^H NMR (400 MHz, C_7_D_8_, 250 K) *δ* = 7.77 (d, *J* = 6.1 Hz, 1H, Ar*H*), 7.27 (d, *J* = 8.0 Hz, 1H, Ar*H*), 7.17 (d, *J* = 5.6 Hz, 1H, Ar*H*), 6.92 (m, 9H, Ar*H*), 6.58 (d, *J* = 9.3 Hz, 1H, Ar*H*), 6.36 (d, *J* = 10.7 Hz, 1H, Ar*H*), 5.30 (d, *J* = 9.0 Hz, 1H, Ar*H*), 4.95 (s, 1H, Ar*H*), 2.56 (m, 2H, C*H*(CH_3_)_2_), 2.44 (m, 2H, C*H*(CH_3_)_2_), 2.38 (m, 2H, C*H*(CH_3_)_2_), 2.34 (m, 2H, C*H*(CH_3_)_2_), 1.48 (m, 12H, CH(C*H*_3_)_2_), 1.29 (m, 21H, CH(C*H*_3_)_2_), 0.87 (m, 12H, CH(C*H*_3_)_2_), 0.67 (m, 3H, CH(C*H*_3_)_2_). ^31^P{^1^H} NMR (162 MHz, C_7_D_8_, 250 K) *δ* = 60.60 (d, *J* = 350.4 Hz), 30.68 (d, *J* = 350.5 Hz), 62.20 (d, *J* = 363.3 Hz), 58.11 (d, *J* = 363.1 Hz). ^1^H NMR (400 MHz, C_6_D_6_, 300 K) *δ* = 5.26 (br, *Δ*_1/2_ = 666.67 Hz, 8H, Ar*H*), 2.96 (br, *Δ*_1/2_ = 414.81 Hz, 24H, CH(C*H*_3_)_2_), 1.36 (br, *Δ*_1/2_ = 88.88 Hz, 2H, C*H*(CH_3_)_2_), 1.00 (br, *Δ*_1/2_ = 103.70 Hz, 2H, C*H*(CH_3_)_2_). Magnetic moment (298 K): *μ*_eff_ = 1.76*μ*_B_. EPR: *g* = 2.0110. Anal. calcd for C_50_H_72_I_2_P_4_Pd_2_: C, 47.52; H, 5.74. Found: C, 47.50; H, 5.70.

### Synthesis of [PC˙(sp^2^)P]PdBr (**3**)

A cold solution of 58.1 mg [PC(sp^2^)P]Pd(PMe_3_) (**1**, 0.1 mmol) in 5 mL THF at –35 °C was stirred for 5 min prior to the addition of 0.05 mL solution of Br_2_ (0.1 M in *n*-pentane). The colour changed rapidly to green. In the crude mixture, the presence of [PC(sp^3^)HP]PdBr (**4**) was determined by ^1^H NMR spectroscopy but was not quantified. The volatiles were removed under reduced pressure and the residue extracted in Et_2_O, concentrated, and filtered over Celite. Analytically pure **3** crystallized at –35 °C (36.8 mg, 63%). For **3**: ^1^H NMR, ^31^P{^1^H} NMR and ^13^C{^1^H} NMR spectra show no signals. Magnetic moment (298 K): *μ*_eff_ = 2.19*μ*_B_. EPR: *g* = 2.0105. Anal. calcd for C_25_H_36_BrP_2_Pd: C, 51.34; H, 6.20. Found: C, 50.99; H, 6.15.

### Synthesis of [PC(sp^3^)H_2_P]PdBr_2_ (**12**) (Scheme 6)

A mixture of 420.5 mg of PC(sp^3^)H_2_P (**13**, 1.05 mmol) and 374.4 mg (COD)PdBr_2_ (1 mmol) in 10 mL THF was stirred for 3 hours. Over this period of time, the mixture changed colour from cream to pale orange/light yellow. The volatiles were removed under reduced pressure and the residue was triturated 3 times with 5 mL of *n*-pentane. The resulted orange powder was dried under reduced pressure and was analytically pure by ^1^H and ^31^P{^1^H} NMR spectroscopy. Yield: 340.7 mg, 91%. For **12**: ^1^H NMR (500 MHz, CD_2_Cl_2_, 248 K) *δ* = 7.63 (t, *J* = 7.9 Hz, 1H, Ar*H*), 7.60–7.55 (m, 1H, Ar*H*), 7.51 (t, *J* = 7.2 Hz, 1H, Ar*H*), 7.42 (t, *J* = 7.5 Hz, 1H, Ar*H*), 7.37 (dt, *J* = 14.8, 6.3 Hz, 3H, Ar*H*), 7.28 (t, *J* = 7.0 Hz, 1H, Ar*H*), 6.69 (ddd, *J* = 14.8, 4.8, 4.8 Hz, 1H, –CH_endo_), 4.03 (d, *J* = 14.8 Hz, 1H, –CH_exo_), 3.96 (dt, *J* = 13.3, 6.9 Hz, 1H, C*H*(CH_3_)_2_), 3.67 (m, 1H, C*H*(CH_3_)_2_), 2.64 (m, 1H, C*H*(CH_3_)_2_), 1.77 (dd, *J* = 20.3, 7.4 Hz, 3H, CH(C*H*_3_)_2_), 1.62 (ddd, *J* = 18.2, 13.6, 6.9 Hz, 6H, CH(C*H*_3_)_2_), 1.45 (dd, *J* = 19.7, 6.8 Hz, 3H, CH(C*H*_3_)_2_), 1.34 (dd, *J* = 18.3, 7.0 Hz, 3H, CH(C*H*_3_)_2_), 1.07 (dd, *J* = 15.7, 7.2 Hz, 3H, CH(C*H*_3_)_2_), 0.89 (dd, *J* = 14.2, 6.8 Hz, 3H, CH(C*H*_3_)_2_), 0.69 (m, 1H, C*H*(CH_3_)_2_), 0.04 (dd, *J* = 13.8, 7.3 Hz, 3H, CH(C*H*_3_)_2_). ^31^P{^1^H} NMR (202 MHz, CD_2_Cl_2_, 248 K) *δ* = 44.66 (d, *J* = 15.0 Hz), 30.65 (d, *J* = 15.1 Hz). ^13^C{^1^H} NMR (126 MHz, CD_2_Cl_2_, 248 K) *δ* = 144.99 (d, *J* = 12.2 Hz, Ar*C*), 141.15 (d, *J* = 11.2 Hz, Ar*C*), 133.12 (d, *J* = 7.2 Hz, Ar*C*), 132.82 (s, Ar*C*), 132.49 (d, *J* = 8.8 Hz, Ar*C*), 132.02 (s, Ar*C*), 131.46 (s, Ar*C*), 130.62 (s, Ar*C*), 130.52 (s, Ar*C*), 126.61 (d, *J* = 3.5 Hz, Ar*C*), 126.41 (m, Ar*C*), 43.17 (t, *J* = 10.5 Hz, –*C*H_2_–), 31.30 (d, *J* = 29.4 Hz, *C*H(CH_3_)_2_), 30.36 (d, *J* = 32.3 Hz, *C*H(CH_3_)_2_), 25.34 (dd, *J* = 18.1, 4.9 Hz, *C*H(CH_3_)_2_), 24.61 (d, *J* = 23.9 Hz, *C*H(CH_3_)_2_), 24.38 (d, *J* = 7.1 Hz, CH(*C*H_3_)_2_), 22.47 (d, *J* = 5.0 Hz, CH(*C*H_3_)_2_), 21.95 (d, *J* = 7.6 Hz, CH(*C*H_3_)_2_), 21.21 (d, *J* = 6.9 Hz, CH(*C*H_3_)_2_), 20.50 (s, CH(*C*H_3_)_2_), 19.75 (s, CH(*C*H_3_)_2_), 19.58 (s, CH(*C*H_3_)_2_), 17.38 (d, *J* = 5.8 Hz, CH(*C*H_3_)_2_). Anal. calcd for C_25_H_38_Br_2_P_2_Pd: C, 45.04; H, 5.74. Found: C, 44.92; H, 5.71.

### Synthesis of [PC(sp^3^)HP]PdBr (**4**)

130 mg of [PC(sp^3^)H_2_P]PdBr_2_ (**12**, 0.195 mmol) was stirred in 10 mL toluene and added as a slurry in a Schlenk flask, under nitrogen. The suspension was heated at 100 °C for 2 days, resulting in a light cream solution. Aliquots were taken from the reaction mixture at intermediate times and analysed by ^1^H and ^31^P{^1^H} NMR spectroscopy to monitor the reaction progress. After 48 hours, only the desired product **4** was observed by ^31^P{^1^H} NMR spectroscopy. The volatiles were removed under reduced pressure and the residue was triturated twice with 10 mL of *n*-pentane. The resulted cream powder was dried under reduced pressure and was analytically pure by ^1^H NMR spectroscopy. Yield: 83.4 mg, 73%. For **4**: ^1^H NMR (500 MHz, CDCl_3_) *δ* = 7.43 (dtd, *J* = 5.0, 3.9, 1.1 Hz, 2H, Ar*H*), 7.33–7.28 (m, 2H, Ar*H*), 7.26 (d, *J* = 7.9 Hz, 2H, Ar*H*), 7.17 (t, *J* = 7.2 Hz, 2H, Ar*H*), 6.15 (s, 1H, –C(*H*)Pd), 2.78–2.70 (m, 2H, C*H*(CH_3_)_2_), 2.69–2.62 (m, 2H, C*H*(CH_3_)_2_), 1.43 (td, *J* = 8.4, 7.2 Hz, 6H, CH(C*H*_3_)_2_), 1.34 (td, *J* = 8.4, 7.1 Hz, 6H, CH(C*H*_3_)_2_), 1.28 (q, *J* = 7.3 Hz, 6H, CH(C*H*_3_)_2_), 1.17 (dd, *J* = 14.8, 7.6 Hz, 6H, CH(C*H*_3_)_2_). ^31^P{^1^H} NMR (202 MHz, CDCl_3_) *δ* = 50.33 (s). ^13^C{^1^H} NMR (126 MHz, CDCl_3_) *δ* = 157.75 (t, *J* = 14.7 Hz, Ar*C*), 133.56 (t, *J* = 17.0 Hz, Ar*C*), 131.90 (s, Ar*C*), 130.23 (s, Ar*C*), 127.09 (t, *J* = 9.2 Hz, Ar*C*), 125.45 (t, *J* = 3.2 Hz, Ar*C*), 54.23 (t, *J* = 3.8 Hz, –*C*(H)Pd), 25.90 (t, *J* = 10.2 Hz, *C*H(CH_3_)_2_), 25.54 (t, *J* = 12.0 Hz, *C*H(CH_3_)_2_), 19.47 (t, *J* = 2.8 Hz, CH(*C*H_3_)_2_), 18.80 (t, *J* = 2.1 Hz, CH(*C*H_3_)_2_), 18.78 (s, CH(*C*H_3_)_2_), 18.38 (s, CH(*C*H_3_)_2_). Anal. calcd for C_25_H_37_BrP_2_Pd: C, 51.26; H, 6.37. Found: C, 51.20; H, 5.99.

### Reaction of [PC(sp^2^)P]Pd(PMe_3_) (**1**) with CH_2_Cl_2_

To a solution of 58.1 mg of [PC(sp^2^)P]Pd(PMe_3_) (**1**, 0.1 mmol) in 5 mL of THF, was added 1 mL solution of CH_2_Cl_2_ (0.1 M) in THF and the reaction mixture was stirred at room temperature for 2.5 days. The color gradually changed from dark brown to green. The reaction was monitored by ^1^H NMR spectroscopy. The volatiles were removed under reduced pressure. [PC(CH_2_)P]Pd(PMe_3_) (**6**) was present in the crude mixture by ^1^H NMR spectroscopy. The residue was dissolved in Et_2_O. Analytically pure **5** crystallized from this Et_2_O solution layered with *n*-pentane at –35 °C. Yield for **5**: 29 mg, 80%. The supernatant contained mostly **6**. The volatiles were removed under reduced pressure and the residue was dissolved in *n*-pentane, filtered over Celite. **6** crystallized from this *n*-pentane solution at –35 °C. Yield: 15 mg, 75%. Due to the low theoretical yield and difficulties encountered in separation, an analytically pure sample of **6** was obtained by a different method (*vide infra*). For **5**: ^1^H, ^31^P{^1^H} and ^13^C{^1^H} NMR are silent. Magnetic moment (298 K): *μ*_eff_ = 1.86*μ*_B_. EPR: *g* = 2.0100. Anal. calcd for C_25_H_36_ClP_2_Pd: C, 55.57; H, 6.72. Found: C, 55.71; H, 6.33.

### Reaction of [PC(sp^2^)P]Pd(PMe_3_) (**1**) with CH_2_Br_2_

To a solution of 58.1 mg of [PC(sp^2^)P]Pd(PMe_3_) (**1**, 1 mmol) in 5 mL of THF, was added 1 mL solution of CH_2_Br_2_ (0.1 M) in THF and the reaction mixture was stirred at room temperature. The color changed within 10 minutes from dark brown to greyish-green. The volatiles were removed under reduced pressure. [PC(CH_2_)P]Pd(PMe_3_) (**6**) was present in the crude mixture by ^1^H and ^31^P{^1^H} NMR spectroscopy. The residue was dissolved in Et_2_O and filtered. Analytically pure **3** crystallized from this Et_2_O solution layered with *n*-pentane at –35 °C. Yield for **3**: 21.1 mg, 54%. The identity of the product **3** was confirmed by converting it to [PC(sp^3^)HP]PdBr (**4**) in a subsequent step (*vide infra*), and by X-ray crystallography. The supernatant contained mostly **6**. The volatiles were removed under reduced pressure, the residue was dissolved in *n*-pentane, and the resulting solution filtered over Celite. Compound **6** crystallized from this *n*-pentane solution at –35 °C. Yield: 7.5 mg, 38%. The spectroscopic data for the crystallized sample was identical to the one obtained by a different method (*vide infra*).

### Reaction of [PC(sp^2^)P]Pd(PMe_3_) (**1**) with CH_2_I_2_

To a solution of 58.1 mg of [PC(sp^2^)P]Pd(PMe_3_) (**1**, 0.1 mmol) in 5 mL of THF was added 1 mL solution of CH_2_I_2_ (0.1 M) in THF and the reaction mixture was stirred at room temperature. The color gradually changed from dark brown to greyish-green. The volatiles were removed under reduced pressure. [PC(CH_2_)P]Pd(PMe_3_) (**6**) was present in the crude mixture by ^1^H and ^31^P{^1^H} NMR spectroscopy. The residue was dissolved in Et_2_O. Analytically pure {**2**}_2_ crystallized from this Et_2_O solution layered with *n*-pentane at –35 °C. Yield for {**2**}_2_: 29.6 mg, 64%. The identity of the product {**2**}_2_ was confirmed by converting it to [PC(sp^3^)HP]PdI (**7**) in a subsequent step (*vide infra*), and by X-ray crystallography. The supernatant contained mostly **6**. The volatiles were removed under reduced pressure and the residue was dissolved in *n*-pentane. **6** crystallized from this *n*-pentane solution at –35 °C. Yield: 9.3 mg, 47%. The spectroscopic data for the crystallized sample was identical to data for a sample obtained by a different method (*vide infra*).

### Synthesis of 1,1-bis(2-bromophenyl)ethan-1-ol

To a suspension of 5.4 g of bis(2-bromophenyl)methanone (16 mmol) in 50 mL of Et_2_O, 10 mL of MeLi (1.6 M in Et_2_O) was added dropwise at room temperature with stirring over a period of 10 minutes. The reaction mixture was stirred at room temperature for 12 hours. 50 mL of H_2_O was carefully added to the reaction mixture. The aqueous layer was extracted 3 times with 50 mL of Et_2_O. The combined organic layers were washed with brine (3 times, 24 mL) and dried over anhydrous sodium sulfate. After filtration, the volatiles were removed under reduced pressure. The crude product was isolated as a clear oil. Yield: 5.5 g, 96%. The crude product was used in the next step without further purification. An analytically pure sample of 1,1-bis(2-bromophenyl)ethan-1-ol was obtained through separation by column chromatography (silica gel, hexanes : ethyl acetate = 95 : 5). For 1,1-bis(2-bromophenyl)ethan-1-ol: ^1^H NMR (500 MHz, CDCl_3_) *δ* = 7.92 (dd, *J* = 7.9, 1.2 Hz, 2H, Ar*H*), 7.50 (dd, *J* = 8.0, 0.8 Hz, 2H, Ar*H*), 7.42–7.34 (m, 2H, Ar*H*), 7.20–7.11 (m, 2H, Ar*H*), 3.57 (s, 1H, –C(O*H*)–CH_3_), 2.05 (s, 1H, –C(OH)–C*H*_3_). ^13^C{^1^H} NMR (126 MHz, CDCl_3_) *δ* = 144.94 (s, Ar*C*), 134.79 (s, Ar*C*), 129.70 (s, Ar*C*), 129.09 (s, Ar*C*), 127.34 (s, Ar*C*), 124.86 (s, Ar*C*), 77.94 (s, Ar_2_ *C*(OH)–CH_3_), 28.35 (s, Ar_2_C(OH)–*C*H_3_).

### Synthesis of 1,1-bis(2-bromophenyl)ethane

A mixture of 5 g of 1,1-bis(2-bromophenyl)ethan-1-ol (14 mmol), 15 g of red phosphorous (483 mmol) and 15 mL of HI (57%) was refluxed for 12 hours. The reaction mixture was diluted with 100 mL H_2_O, and extracted multiple times with CH_2_Cl_2_. The combined organic extract was washed with diluted NaOH, water, brine and dried over anhydrous sodium sulfate. After filtration, the volatiles were removed under reduced pressure to generate the crude product as an oil. The product was purified on a silica gel column using hexanes : ethyl acetate 95 : 5. Yield: 4 g, 84%. For 1,1-bis(2-bromophenyl)ethane: ^1^H NMR (500 MHz, CDCl_3_) *δ* = 7.60 (d, *J* = 7.3 Hz, 2H, Ar*H*), 7.29 (t, *J* = 7.5 Hz, 2H, Ar*H*), 7.19–7.09 (m, 4H, Ar*H*), 4.86 (q, *J* = 7 Hz, 1H, Ar_2_C(*H*)–CH_3_), 1.62 (d, *J* = 7 Hz, 3H, Ar_2_C(H)–C*H*_3_). ^13^C{^1^H} NMR (126 MHz, CDCl_3_) *δ* = 144.15 (s, Ar*C*), 133.19 (s, Ar*C*), 128.57 (s, Ar*C*), 127.95 (s, Ar*C*), 127.54 (s, Ar*C*), 125.60 (s, Ar*C*), 77.16 (s, Ar_2_ *C*(H)–CH_3_), 20.35 (s, Ar_2_C(H)–*C*H_3_).

### Synthesis of PC(CH_3_)HP (**7**)

To a solution of 1,1-bis(2-bromophenyl)ethane (4 g, 11.8 mmol) in 50 mL of Et_2_O, 15 mL of a ^*n*^BuLi solution (1.6 M in hexanes, 24 mmol) was added dropwise at –50 °C in a nitrogen-filled glovebox. The reaction mixture was warmed up to room temperature and stirred for an additional hour. To this mixture, a solution of 3.66 g of ^i^Pr_2_PCl (24 mmol) in Et_2_O was added dropwise over a period of 30 minutes and stirred overnight at room temperature. The reaction mixture was quenched with 5 mL of a degassed, saturated NH_4_Cl solution in water. The volatiles were removed under reduced pressure and the residue was dissolved in *n*-pentane. The pentane solution was dried over anhydrous sodium sulfate, filtered over Celite and concentrated under reduced pressure. The product, **7**, crystallized as a white solid from this solution at –35 °C. Yield: 3.8 g, 78%. For **7**: ^1^H NMR (500 MHz, C_6_D_6_) *δ* = 7.39–7.33 (m, 2H, Ar*H*), 7.19 (ddd, *J* = 7.8, 3.8, 1.3 Hz, 2H, Ar*H*), 7.08 (td, *J* = 7.5, 1.4 Hz, 2H, Ar*H*), 7.03 (td, *J* = 7.3, 1.4 Hz, 2H, Ar*H*), 6.25 (m, *J* = 6.9 Hz, 1H, –C(*H*)CH_3_), 2.05 (m, 2H, C*H*(CH_3_)_2_), 1.90 (m, 2H, C*H*(CH_3_)_2_), 1.76 (d, *J* = 7.1 Hz, 3H, –C(H)C*H*_3_), 1.17 (dd, *J* = 14.2, 6.9 Hz, 6H, CH(C*H*_3_)_2_), 1.09 (dd, *J* = 13.6, 6.9 Hz, 6H, CH(C*H*_3_)_2_), 0.91 (dd, *J* = 11.3, 7.0 Hz, 6H, CH(C*H*_3_)_2_), 0.86 (dd, *J* = 13.5, 7.1 Hz, 6H, CH(C*H*_3_)_2_). ^31^P{^1^H} NMR (202 MHz, C_6_D_6_) *δ* = –8.28. ^13^C{^1^H} NMR (126 MHz, C_6_D_6_) *δ* = 153.55 (d, *J* = 25.8 Hz, Ar*C*), 136.71 (d, *J* = 19.9 Hz, Ar*C*), 132.97 (s, Ar*C*), 128.81 (s, Ar*C*), 128.57 (t, *J* = 3.5 Hz, Ar*C*), 125.72 (s, Ar*C*), 40.71 (t, *J* = 23.2 Hz, –*C*(H)CH_3_), 26.34 (d, *J* = 14.8 Hz, *C*H(CH_3_)_2_), 25.39 (d, *J* = 11.7 Hz, *C*H(CH_3_)_2_), 24.33 (t, *J* = 3.6 Hz, –C(H)*C*H_3_), 22.02–21.50 (m, CH(*C*H_3_)_2_), 21.32–20.82 (m, CH(*C*H_3_)_2_), 20.37 (m, CH(*C*H_3_)_2_).

### Synthesis of [PC(CH_3_)HP]PdCl_2_ (**8**)

A mixture of 85 mg of PC(CH_3_)HP (**7**) (0.206 mmol) and 57 mg of (COD)PdCl_2_ (0.2 mmol) was stirred in 5 mL of THF for 3 hours at room temperature. The solution remained cloudy yellow throughout the reaction. The volatiles were removed under reduced pressure and the residue was triturated 3 times with 5 mL of *n*-pentane. The resulting yellow powder (91 mg, 76%) was analytically pure based on ^1^H and ^31^P{^1^H} NMR spectroscopy. Because the room temperature ^1^H NMR spectrum was broad, additional NMR data was recorded at 300, 310, and 320 K. For **8**: ^1^H NMR (400 MHz, CDCl_3_, 320 K) *δ* = 7.60–7.52 (m, 4H, Ar*H*), 7.44 (t, *J* = 7.6 Hz, 2H, Ar*H*), 7.27 (t, *J* = 7.6 Hz, 2H, Ar*H*), 7.00–6.91 (m, 1H, –C(*H*)CH_3_), 3.76–3.50 (m, 2H, C*H*(CH_3_)_2_), 1.80 (d, *J* = 6.7 Hz, 3H, –C(H)C*H*_3_), 1.71 (br s, 2H, C*H*(CH_3_)_2_), 1.66 (dd, *J* = 15.9, 7.1 Hz, 6H, CH(C*H*_3_)_2_), 1.46 (ddd, *J* = 17.4, 15.1, 7.2 Hz, 12H, CH(C*H*_3_)_2_), 0.94 (dd, *J* = 13.5, 6.8 Hz, 6H, CH(C*H*_3_)_2_). ^31^P{^1^H} NMR (162 MHz, CDCl_3_, 320 K) *δ* = 39.96 (s). ^13^C{^1^H} NMR (101 MHz, CDCl_3_, 290 K) *δ* = 148.92 (s, Ar*C*), 131.82 (s, Ar*C*), 131.31 (d, *J* = 2.0 Hz, Ar*C*), 129.67 (s, Ar*C*), 129.42 (d, *J* = 7.9 Hz, Ar*C*), 126.14 (d, *J* = 6.7 Hz, Ar*C*), 40.69 (t, *J* = 10.4 Hz, –*C*(H)CH_3_), 29.02 (d, *J* = 30.1 Hz, *C*H(CH_3_)_2_), 26.80 (d, *J* = 23.9 Hz, *C*H(CH_3_)_2_), 25.67 (s, –C(H)*C*H_3_), 22.26 (s, CH(*C*H_3_)_2_), 22.11 (s, CH(*C*H_3_)_2_), 21.85 (d, *J* = 2.0 Hz, CH(*C*H_3_)_2_), 20.96 (d, *J* = 3.7 Hz, CH(*C*H_3_)_2_). Anal. calcd for C_26_H_40_Cl_2_P_2_Pd: C, 52.76; H, 6.81. Found: C, 53.01; H, 6.79.

### Synthesis of [PC(CH_3_)P]PdCl (**9**)

A solution of 100 mg of [PC(CH_3_)HP]PdCl_2_ (8, 0.169 mmol) in 10 mL of dioxane was heated at 100 °C for 36 hours in a Schlenk tube. The volatiles were removed under reduced pressure and the residue was triturated 3 times with 10 mL of *n*-pentane. After recrystallization from a concentrated Et_2_O solution at –35 °C, analytically pure **9** was isolated in high yield (92%, 86.3 mg). For **9**: ^1^H NMR (400 MHz, C_6_D_6_) *δ* = 7.45 (dd, *J* = 8.0, 0.8 Hz, 2H, Ar*H*), 7.14 (ddd, *J* = 7.5, 3.7, 1.2 Hz, 2H, Ar*H*), 7.09–7.04 (m, 2H, Ar*H*), 6.94 (t, *J* = 7.3 Hz, 2H, Ar*H*), 2.58–2.38 (m, 4H, C*H*(CH_3_)_2_), 2.05 (t, *J* = 3.9 Hz, 3H, Pd–C(C*H*_3_)), 1.44 (dd, *J* = 13.9, 5.1 Hz, 6H, CH(C*H*_3_)_2_), 1.39 (dd, *J* = 13.6, 5.3 Hz, 6H, CH(C*H*_3_)_2_), 1.14 (dd, *J* = 15.1, 7.4 Hz, 6H, CH(C*H*_3_)_2_), 0.98 (dd, *J* = 15.2, 7.2 Hz, 6H, CH(C*H*_3_)_2_). ^31^P{^1^H} NMR (162 MHz, C_6_D_6_) *δ* = 46.67 (s). ^13^C{^1^H} NMR (101 MHz, C_6_D_6_) *δ* = 164.21 (t, *J* = 14.7 Hz, Ar*C*), 135.49 (t, *J* = 15.6 Hz, Ar*C*), 132.72 (s, Ar*C*), 129.94 (s, Ar*C*), 127.27 (t, *J* = 9.3 Hz, Ar*C*), 126.06 (t, *J* = 3.0 Hz, Ar*C*), 65.94 (t, *J* = 4.7 Hz, Pd–*C*(CH_3_)), 40.74 (t, *J* = 2.2 Hz, Pd–C(*C*H_3_)), 26.96 (t, *J* = 11.7 Hz, *C*H(CH_3_)_2_), 26.40 (t, *J* = 9.9 Hz, *C*H(CH_3_)_2_), 19.56 (t, *J* = 1.7 Hz, CH(*C*H_3_)_2_), 19.30 (t, *J* = 2.5 Hz, CH(*C*H_3_)_2_), 19.13 (t, *J* = 2.2 Hz, CH(*C*H_3_)_2_), 19.01 (t, *J* = 1.4 Hz, CH(*C*H_3_)_2_). Anal. calcd for C_26_H_39_ClP_2_Pd: C, 56.23; H, 7.08. Found: C, 56.31; H, 6.72.

### Synthesis of [PC(CH_2_)P]Pd(PMe_3_) (**6**)

To a solution of 50 mg of [PC(CH_3_)P]PdCl (**9**, 0.090 mmol) in 5 mL of THF, one equivalent of PMe_3_ (0.9 mL of a 0.1 M solution in THF) was added and the mixture was cooled to –35 °C. To this cold mixture, 1.36 mL KN(TMS)_2_ (0.066 M in toluene) was added. The solution rapidly changed color to orange, and the reaction was warmed up to room temperature, and stirred for one additional hour. After removal of volatiles under reduced pressure, the residue was extracted with *n*-pentane and the solution filtered over Celite. Analytically pure product was isolated by crystallization at –35 °C from *n*-pentane. Yield: 40 mg (75%). For **6**: ^1^H NMR (400 MHz, C_6_D_6_) *δ* = 7.80 (dd, *J* = 7.7, 1.4 Hz, 2H, Ar*H*), 7.30 (ddd, *J* = 7.6, 3.6, 1.4 Hz, 2H, Ar*H*), 7.07 (t, *J* = 7.4 Hz, 2H, Ar*H*), 7.00 (td, *J* = 7.3, 1.4 Hz, 2H, Ar*H*), 3.81 (d, *J* = 4.4 Hz, 2H, –Pd(C=CH_2_)), 2.24 (m, 2H, C*H*(CH_3_)_2_), 1.99 (m, 2H, C*H*(CH_3_)_2_), 1.36 (d, *J* = 4.6 Hz, 9H, –P(C*H*_3_)_3_), 1.21 (dd, *J* = 15.3, 6.9 Hz, 6H, CH(C*H*_3_)_2_), 1.04 (ddd, *J* = 13.0, 10.2, 7.1 Hz, 12H, CH(C*H*_3_)_2_), 0.95 (dd, *J* = 12.4, 7.0 Hz, 6H, CH(C*H*_3_)_2_). ^31^P{^1^H} NMR (162 MHz, C_6_D_6_) *δ* = 33.85 (d, *J* = 21.7 Hz, *P* ^i^Pr_2_), –31.55 (t, *J* = 21.6 Hz, *P*Me_3_). ^13^C{^1^H} NMR (101 MHz, C_6_D_6_) *δ* 155.08–154.28 (m, Ar*C*), 143.19 (dt, *J* = 10.5, 8.0 Hz, Ar*C*), 131.38 (d, *J* = 1.3 Hz, Ar*C*), 129.80 (dd, *J* = 9.9, 5.1 Hz, Ar*C*), 127.58 (s, Ar*C*), 125.40 (s, Ar*C*), 112.16 (dt, *J* = 17.6, 2.3 Hz, –Pd(*C* =CH_2_)), 64.40 (td, *J* = 7.5, 5.1 Hz, –Pd(C= *C*H_2_)), 28.38–28.15 (m, *C*H(CH_3_)_2_), 26.49 (t, *J* = 4.9 Hz, *C*H(CH_3_)_2_), 25.02 (t, *J* = 5.7 Hz, P(*C*H_3_)_3_), 24.90 (t, *J* = 5.8 Hz, CH(*C*H_3_)_2_), 21.07 (t, *J* = 6.0 Hz, CH(*C*H_3_)_2_), 20.77 (t, *J* = 8.2 Hz, CH(*C*H_3_)_2_), 19.96–19.66 (m, CH(*C*H_3_)_2_). Anal. calcd for C_29_H_47_P_3_Pd: C, 58.54; H, 7.96. Found: C, 58.50; H, 8.01.

### Reaction of **5**, **3** or {**2**}_2_ with 9,10-dihydroanthracene

In a typical experiment a 20 mL scintillation vial, the radical (**5**, 54 mg, 0.1 mmol; **3**, 59 mg, 0.1 mmol; {**2**}_2_, 64 mg, 0.05 mmol) were mixed with 36 mg of 9,10-dihydroantracene (0.4 mmol) in 5 mL of THF and stirred at room temperature. After about 12 hours, the color changed to a lighter shade of green. The volatiles were then removed under reduced pressure and the residue was extracted in Et_2_O. The reaction was monitored by ^1^H and ^31^P{^1^H} NMR. The product was isolated by crystallization from this concentrated Et_2_O solution at –35 °C. The ^1^H and ^31^P{^1^H} NMR spectra matched the spectra previously obtained for the products. Isolated yield: 14% for **10**, 16% for **4** and 17% for **11**.

### Reaction of **5**, **3** or {**2**}_2_ with ^*n*^Bu_3_SnH

In a typical experiment a 20 mL scintillation vial, the radical (**5**, 54 mg, 0.1 mmol; **3**, 59 mg, 0.1 mmol; {**2**}_2_, 64 mg, 0.05 mmol) were mixed with 4 mL solution of ^*n*^Bu_3_SnH (0.05 M in THF, 0.2 mmol) and stirred at room temperature. After about 12 hours the color changed to light green. The reaction was monitored by ^1^H and ^31^P{^1^H} NMR. The volatiles were then removed under reduced pressure and the residue was extracted in Et_2_O. The products were isolated by crystallization from this concentrated Et_2_O solution at –35 °C. The ^1^H and ^31^P{^1^H} NMR spectra matched the spectra previously obtained for these compounds. Yield 48% for **10**, 24% for **4** and 78% for **11**.

### Synthesis of [PC(sp^3^)HP]PdI (**11**)

54.1 mg of [PC(sp^3^)HP]PdCl (**10**, 0.1 mmol) were stirred in 5 mL of THF in a 20 mL scintillation vial, at –50 °C for 30 minutes. To this mixture, 1 mL solution of I_2_ (0.1 M in THF) was added dropwise. The color gradually changed from cream to bright yellow. After warming up to room temperature, the reaction mixture was stirred for an additional 1 hour. The volatiles were removed under reduced pressure, and the yellow residue was extracted with Et_2_O and filtered over Celite. This Et_2_O solution was concentrated under reduced pressure and set to crystallize at –35 °C. After 2 days, analytically pure **11** (43 mg, 68%) was isolated as light yellow crystals. For **11**: ^1^H NMR (400 MHz, C_6_D_6_) *δ* = 7.29 (ddd, *J* = 7.8, 1.4, 1.0 Hz, 2H, Ar*H*), 7.14 (ddd, *J* = 7.5, 3.9, 1.5 Hz, 2H, Ar*H*), 7.09–7.04 (m, 2H, Ar*H*), 6.90 (t, *J* = 7.4 Hz, 2H, Ar*H*), 6.37 (s, 1H, Pd–C*H*), 2.66–2.55 (m, 2H, C*H*(CH_3_)_2_), 2.55–2.44 (m, 2H, C*H*(CH_3_)_2_), 1.38 (qd, *J* = 8.3, 7.1 Hz, 12H, CH(C*H*_3_)_2_), 1.06 (dd, *J* = 14.7, 7.4 Hz, 6H, CH(C*H*_3_)_2_), 1.02 (q, *J* = 7.5 Hz, 6H, CH(C*H*_3_)_2_). ^31^P{^1^H} NMR (162 MHz, C_6_D_6_) *δ* = 51.43 (s). ^13^C{^1^H} NMR (101 MHz, C_6_D_6_) *δ* = 158.14 (t, *J* = 14.6 Hz, Ar*C*), 135.04 (t, *J* = 16.7 Hz, Ar*C*), 132.27 (s, Ar*C*), 130.40 (s, Ar*C*), 127.40 (t, *J* = 9.2 Hz, Ar*C*), 125.47 (t, *J* = 3.2 Hz, Ar*C*), 59.31 (t, *J* = 4.4 Hz Pd–*C*H), 26.77 (t, *J* = 10.4 Hz, *C*H(CH_3_)_2_), 26.74 (t, *J* = 12.6 Hz, *C*H(CH_3_)_2_), 19.88 (t, *J* = 2.6 Hz, CH(*C*H_3_)_2_), 19.26 (t, *J* = 1.9 Hz, CH(*C*H_3_)_2_), 18.47 (s, CH(*C*H_3_)_2_). Anal. calcd for C_25_H_37_IP_2_Pd: C, 47.45; H, 5.89. Found: C, 47.48; H, 5.75.
