# Supplementary material for: Palladium carbene complexes as persistent radicals
Source: Chem Sci. 2015 May 18;6(8):4570–9. doi: 10.1039/c5sc01441g (PMC5500858; doi:10.1039/c5sc01441g)
Supplement: Supplementary file 2 [file SC-006-C5SC01441G-s002.pdf]

Electronic Supplementary Material (ESI) for Chemical Science.
This journal is © The Royal Society of Chemistry 2015

Electronic Supporting information for:

Palladium carbene complexes as persistent radicals

Cezar C. Comanescu,[†] *Mariya Vyushkova,*[‡] *and Vlad M. Iluc*[†]*

[†]Department of Chemistry and Biochemistry, University of Notre Dame, Notre Dame, IN 46556
[‡]Notre Dame Radiation Laboratory, Notre Dame, Indiana 46556

*Corresponding author: viluc@nd.edu

1 X-ray data for compounds {2}$_2$, 3-6, 8, 9, 11, 12 **S6**

2 Magnetic moment for {[PC(sp^2)P]PdI}$_2$ ({2}$_2$) **S8**
Figure S1. Magnetic moment for **{2}$_2$** at different temperatures . . . S8

3 Cyclic Voltammetry **S8**
Figure S2. Cyclic voltammogram of [PC(sp^2)P]Pd(PMe$_3$) (**1**) . . . S8

4 EPR Spectra **S9**
Figure S3. EPR spectrum of [PC$^•$(sp^2)P]PdI (**2**) . . . S9
Figure S4. EPR spectrum of [PC$^•$(sp^2)P]PdBr (**3**) . . . S9
Figure S5. EPR spectrum of [PC$^•$(sp^2)P]PdCl (**5**) . . . S10

5 Complete Reference 24 **S10**

6 DFT Results **S11**
Figure S6. Computed molecules . . . S11
6.1 {[PC(sp^2)P]PdI}$_2$ (**{2}$_2$**) . . . S12
Table S1. Optimized coordinates for {[PC(sp^2)P]PdI}$_2$ (**{2}$_2$**) . . . S12
Figure S7. Overlaid structures for {[PC(sp^2)P]PdI}$_2$ (**{2}$_2$**) (red: X-ray, blue: optimized) . . . S15
Table S2. Selected distances (Å) and angles (°) for the optimized geometry and the crystal structure of {[PC(sp^2)P]PdI}$_2$ (**{2}$_2$**) . . . S16
Figure S8. Optimized geometry for {[PC(sp^2)P]PdI}$_2$ (**{2}$_2$**) . . . S16
6.2 {*p,p'*-[PC(sp^2)P]PdI}$_2$ (**{*p,p'*-2}$_2$**) . . . S17
Table S3. Optimized coordinates for {*p,p'*-[PC(sp^2)P]PdI}$_2$ (**{*p,p'*-2}$_2$**) . . . S17
Figure S9. Optimized geometry for {*p,p'*-[PC(sp^2)P]PdI}$_2$ (**{*p,p'*-2}$_2$**) . . . S20
6.3 [PC$^•$(sp^2)P]PdI (**2**) . . . S21
Table S4. Optimized coordinates for [PC$^•$(sp^2)P]PdI (**2**) . . . S21
Figure S10. Optimized geometry for [PC$^•$(sp^2)P]PdI (**2**) . . . S23
6.4 Calculated energies for [PC$^•$(sp^2)P]PdI (**2**) and corresponding dimers . . . S23

6.5 [$Me_2PC^\bullet(sp^2)PMe_2$]PdI (**2’**) S24
Table S5. Optimized coordinates for [$Me_2PC^\bullet(sp^2)PMe_2$]PdI (**2’**) S24
Figure S11. Optimized geometry for [$Me_2PC^\bullet(sp^2)PMe_2$]PdI (**2’**) S25
Figure S12. Spin density for [$Me_2PC^\bullet(sp^2)PMe_2$]PdI (**2’**) S25
Figure S13. Frontier molecular orbitals for [$Me_2PC^\bullet(sp^2)PMe_2$]PdI (**2’**) S26
6.6 [$PC^\bullet(sp^2)P$]PdBr (**3**) S27
Table S6. Optimized coordinates for [$PC^\bullet(sp^2)P$]PdBr (**3**) S27
Table S7. Selected distances (Å) and angles (°) for the optimized geometry and the crystal structure of [$PC^\bullet(sp^2)P$]PdBr (**3**) S28
Figure S14. Optimized geometry for [$PC^\bullet(sp^2)P$]PdBr (**3**) S29
Figure S15. Overlaid structures for [$PC^\bullet(sp^2)P$]PdBr (**3**) (red: X-ray, blue: optimized) S29
6.7 {[$PC(sp^2)P$]PdBr}$_2$ (**{3}$_2$**) S30
Table S8. Optimized coordinates for {[$PC(sp^2)P$]PdBr}$_2$ (**{3}$_2$**) S30
Figure S16. Optimized geometry for {[$PC(sp^2)P$]PdBr}$_2$ (**{3}$_2$**) S33
6.8 {*p,p’*-[$PC(sp^2)P$]PdBr}$_2$ (**{*p,p’*-3}$_2$**) S34
Table S9. Optimized coordinates for {*p,p’*-[$PC(sp^2)P$]PdBr}$_2$ (**{*p,p’*-3}$_2$**) S34
Figure S17. Optimized geometry for {*p,p’*-[$PC(sp^2)P$]PdBr}$_2$ (**{*p,p’*-3}$_2$**) S37
6.9 Calculated energies for [$PC^\bullet(sp^2)P$]PdBr (**3**) and corresponding dimers S38
6.10 [$Me_2PC^\bullet(sp^2)PMe_2$]PdBr (**3’**) S39
Table S10. Optimized coordinates for [$Me_2PC^\bullet(sp^2)PMe_2$]PdBr (**3’**) S39
Figure S18. Optimized geometry for [$Me_2PC^\bullet(sp^2)PMe_2$]PdBr (**3’**) S40
Figure S19. Spin density for [$Me_2PC^\bullet(sp^2)PMe_2$]PdBr (**3’**) S40
Figure S20. Frontier molecular orbitals for [$Me_2PC^\bullet(sp^2)PMe_2$]PdBr (**3’**) S41
6.11 [$PC^\bullet(sp^2)P$]PdCl (**5**) S42
Table S11. Optimized coordinates for [$PC^\bullet(sp^2)P$]PdCl (**5**) S42
Table S12. Selected distances (Å) and angles (°) for the optimized geometry and the crystal structure of [$PC^\bullet(sp^2)P$]PdCl (**5**) S43
Figure S21. Optimized geometry for [$PC^\bullet(sp^2)P$]PdCl (**5**) S44
Figure S22. Overlaid structures for [$PC^\bullet(sp^2)P$]PdCl (**5**) (red: Xray, blue: optimized) S44
6.12 {[$PC(sp^2)P$]PdCl}$_2$ (**{5}$_2$**) S45
Table S13. Optimized coordinates for {[$PC(sp^2)P$]PdCl}$_2$ (**{5}$_2$**) S45
Figure S23. Optimized geometry for {[$PC(sp^2)P$]PdCl}$_2$ (**{5}$_2$**) S48
6.13 {*p,p’*-[$PC(sp^2)P$]PdCl}$_2$ (**{*p,p’*-5}$_2$**) S49
Table S14. Optimized coordinates for {*p,p’*-[$PC(sp^2)P$]PdCl}$_2$ (**{*p,p’*-5}$_2$**) S49
Figure S24. Optimized geometry for {*p,p’*-[$PC(sp^2)P$]PdCl}$_2$ (**{*p,p’*-5}$_2$**) S52
6.14 Calculated energies for [$PC^\bullet(sp^2)P$]PdCl (**5**) and corresponding dimers S53
6.15 [$Me_2PC^\bullet(sp^2)PMe_2$]PdCl (**5’**) S54
Table S15. Optimized coordinates for [$Me_2PC^\bullet(sp^2)PMe_2$]PdCl (**5’**) S54
Figure S25. Optimized geometry for [$Me_2PC^\bullet(sp^2)PMe_2$]PdCl (**5’**) S55
Figure S26. Spin density for [$Me_2PC^\bullet(sp^2)PMe_2$]PdCl (**5’**) S55
Figure S27. Frontier molecular orbitals for [$Me_2PC^\bullet(sp^2)PMe_2$]PdCl (**5’**) S56

7 NMR Spectra **S57**
7.1 NMR Spectra for [PC•(sp^2)P]PdI (**2**)and {[PC(sp^2)P]PdI}$_2$ (**{2}$_2$**) S57
Figure S28. ^{1}H NMR spectrum (293 K) for [PC•(sp^2)P]PdI (**2**) S57
7.2 NMR Spectra for [PC(sp^3)HP]PdBr (**4**) S58
Figure S29. ^{1}H NMR spectrum for [PC(sp^3)HP]PdBr (**4**) S58
Figure S30. ^{31}P{^{1}H} NMR spectrum for [PC(sp^3)HP]PdBr (**4**) S59
Figure S31. ^{13}C{^{1}H} NMR spectrum for [PC(sp^3)HP]PdBr (**4**) S60
Figure S32. ^{1}H-^{13}C HSQC NMR spectrum for [PC(sp^3)HP]PdBr (**4**) S61
Figure S33. ^{1}H-^{1}H COSY NMR spectrum for [PC(sp^3)HP]PdBr (**4**) S62
7.3 NMR Spectra for [PC(CH_2)P]Pd(PMe_3) (**6**) S63
Figure S34. ^{1}H NMR spectrum for [PC(CH_2)P]Pd(PMe_3) (**6**) S63
Figure S35. ^{31}P{^{1}H} NMR spectrum for [PC(CH_2)P]Pd(PMe_3) (**6**) S64
Figure S36. ^{13}C{^{1}H} NMR spectrum for [PC(CH_2)P]Pd(PMe_3) (**6**) S65
Figure S37. ^{1}H-^{13}C HSQC NMR spectrum for [PC(CH_2)P]Pd(PMe_3) (**6**) S66
7.4 NMR Spectra for 1,1-bis(2-bromophenyl)ethan-1-ol S67
Figure S38. ^{1}H NMR spectrum for 1,1-bis(2-bromophenyl)ethan-1-ol S67
Figure S39. ^{13}C{^{1}H} NMR spectrum for 1,1-bis(2-bromophenyl)ethan-1-ol S68
7.5 NMR Spectra for 1,1-bis(2-bromophenyl)ethane S69
Figure S40. ^{1}H NMR spectrum for 1,1-bis(2-bromophenyl)ethane S69
Figure S41. ^{13}C{^{1}H} NMR spectrum for 1,1-bis(2-bromophenyl)ethane S70
7.6 NMR Spectra for PC(CH_3)HP (**7**) S71
Figure S42. ^{1}H NMR spectrum for PC(CH_3)HP (**7**) S71
Figure S43. ^{31}P{^{1}H} NMR spectrum for PC(CH_3)HP (**7**) S72
Figure S44. ^{13}C{^{1}H} NMR spectrum for PC(CH_3)HP (**7**) S73
Figure S45. ^{1}H-^{13}C HSQC NMR spectrum for PC(CH_3)HP (**7**) S74
Figure S46. ^{1}H-^{1}H COSY NMR spectrum for PC(CH_3)HP (**7**) S75
7.7 NMR Spectra for [PC(CH_3)HP]$PdCl_2$ (**8**) S76
Figure S47. ^{1}H NMR spectrum (290 K) for [PC(CH_3)HP]$PdCl_2$ (**8**) S76
Figure S48. ^{1}H NMR spectrum (320 K) for [PC(CH_3)HP]$PdCl_2$ (**8**) S77
Figure S49. ^{31}P{^{1}H} NMR spectrum (290 K) for [PC(CH_3)HP]$PdCl_2$ (**8**) S78
Figure S50. ^{31}P{^{1}H} NMR spectrum (320 K) for [PC(CH_3)HP]$PdCl_2$ (**8**) S79
Figure S51. ^{13}C{^{1}H} NMR spectrum (290 K) for [PC(CH_3)HP]$PdCl_2$ (**8**) S80
Figure S52. ^{1}H-^{13}C HSQC NMR spectrum (290 K) for [PC(CH_3)HP]$PdCl_2$ (**8**) . . . S81
Figure S53. Variable temperature ^{1}H NMR spectra for [PC(CH_3)HP]$PdCl_2$ (**8**) . . . S82
7.8 NMR Spectra for [PC(CH_3)P]PdCl (**9**) S83
Figure S54. ^{1}H NMR spectrum for [PC(CH_3)P]PdCl (**9**) S83
Figure S55. ^{31}P{^{1}H} NMR spectrum for [PC(CH_3)P]PdCl (**9**) S84
Figure S56. ^{13}C{^{1}H} NMR spectrum for [PC(CH_3)P]PdCl (**9**) S85
7.9 NMR Spectra for [PC(sp^3)HP]PdI (**11**) S86
Figure S57. ^{1}H NMR spectrum for [PC(sp^3)HP]PdI (**11**) S86
Figure S58. ^{31}P{^{1}H} NMR spectrum for [PC(sp^3)HP]PdI (**11**) S87
Figure S59. ^{13}C{^{1}H} NMR spectrum for [PC(sp^3)HP]PdI (**11**) S88
Figure S60. ^{1}H-^{1}H COSY NMR spectrum for [PC(sp^3)HP]PdI (**11**) S89
Figure S61. ^{1}H-^{13}C HSQC NMR spectrum for [PC(sp^3)HP]PdI (**11**) S90
Figure S62. ^{1}H-^{13}C HMBC NMR spectrum for [PC(sp^3)HP]PdI (**11**) S91

7.10 NMR Spectra for $[PC(sp^3)H_2P]PdBr_2$ (**12**) S92
Figure S63. ^{1}H NMR spectrum (298 K) for $[PC(sp^3)H_2P]PdBr_2$ (**12**) S92
Figure S64. $^{31}P\{^1H\}$ NMR spectrum (298 K) for $[PC(sp^3)H_2P]PdBr_2$ (**12**) S93
Figure S65. $^{13}C\{^1H\}$ NMR (298 K) spectrum for $[PC(sp^3)H_2P]PdBr_2$ (**12**) S94
Figure S66. ^{1}H NMR (248 K) spectrum for $[PC(sp^3)H_2P]PdBr_2$ (**12**) S95
Figure S67. $^{31}P\{^1H\}$ NMR spectrum (248 K) for $[PC(sp^3)H_2P]PdBr_2$ (**12**) S96
Figure S68. $^{13}C\{^1H\}$ NMR spectrum (248 K) for $[PC(sp^3)H_2P]PdBr_2$ (**12**) S97
Figure S69. ^1H-^1H COSY NMR spectrum (248 K) for $[PC(sp^3)H_2P]PdBr_2$ (**12**) . . . S98
Figure S70. ^1H-^{13}C HSQC NMR (248 K) spectrum for $[PC(sp^3)H_2P]PdBr_2$ (**12**) . . . S99
Figure S71. Variable temperature ^{1}H NMR spectra for $[PC(sp^3)H_2P]PdBr_2$ (**12**) . . . S100
Figure S72. Variable temperature $^{31}P\{^1H\}$ NMR spectra for $[PC(sp^3)H_2P]PdBr_2$ (**12**) S101

8 Crystallographic tables **S102**
8.1 Crystal data for $\{[PC(sp^2)P]PdI\}_2 \cdot ½Et_2O$ (**$\{2\}_2 \cdot ½Et_2O$**) S102
Figure S73. Thermal-ellipsoid representation of $\{[PC(sp^2)P]PdI\}_2$ (**$\{2\}_2$**) S102
Table S16. Crystal data and structure refinement for **$\{2\}_2$** S103
Table S17. Atomic coordinates and equivalent isotropic displacement parameters (Å^2) for **$\{2\}_2 \cdot ½Et_2O$** S104
Table S18. Anisotropic displacement parameters (Å^2) for **$\{2\}_2 \cdot ½Et_2O$** S108
Table S19. Distances [Å] for **$\{2\}_2 \cdot ½Et_2O$** S110
Table S20. Angles [°] for **$\{2\}_2 \cdot ½Et_2O$** S112
8.2 Crystal data for $[PC^{\bullet}(sp^2)P]PdBr$ (**3**) S116
Figure S74. Thermal-ellipsoid representation of $[PC^{\bullet}(sp^2)P]PdBr$ (**3**) S116
Table S21. Crystal data and structure refinement for **3** S117
Table S22. Atomic coordinates and equivalent isotropic displacement parameters (Å^2) for **3** S118
Table S23. Anisotropic displacement parameters (Å^2) for **3** S120
Table S24. Distances [Å] for **3** S121
Table S25. Angles [°] for **3** S122
8.3 Crystal data for $[PC(sp^3)HP]PdBr$ (**4**) S124
Figure S75. Thermal-ellipsoid representation of $[PC(sp^3)HP]PdBr$ (**4**) S124
Table S26. Crystal data and structure refinement for **4** S125
Table S27. Atomic coordinates and equivalent isotropic displacement parameters (Å^2) for **4** S126
Table S28. Anisotropic displacement parameters (Å^2) for **4** S128
Table S29. Distances [Å] for **4** S129
Table S30. Angles [°] for **4** S130
8.4 Crystal data for $[PC^{\bullet}(sp^2)P]PdCl$ (**5**) S132
Figure S76. Thermal-ellipsoid representation of $[PC^{\bullet}(sp^2)P]PdCl$ (**5**) S132
Table S31. Crystal data and structure refinement for **5** S133
Table S32. Atomic coordinates and equivalent isotropic displacement parameters (Å^2) for **5** S134
Table S33. Anisotropic displacement parameters (Å^2) for **5** S136
Table S34. Distances [Å] for **5** S137
Table S35. Angles [°] for **5** S138

8.5 Crystal data for [PC(CH_2)P]Pd(PMe_3) (**6**) S140
Figure S77. Thermal-ellipsoid representation of [PC(CH_2)P]Pd(PMe_3) (**6**) S140
Table S36. Crystal data and structure refinement for **6** S141
Table S37. Atomic coordinates and equivalent isotropic displacement parameters ($Å^2$) for **6** S142
Table S38. Anisotropic displacement parameters ($Å^2$) for **6** S144
Table S39. Distances [Å] for **6** S145
Table S40. Angles [°] for **6** S146
8.6 Crystal data for [PC(CH_3)HP]$PdCl_2$ (**8**) S148
Figure S78. Thermal-ellipsoid representation of [PC(CH_3)HP]$PdCl_2$ (**8**) S148
Table S41. Crystal data and structure refinement for **8** S149
Table S42. Atomic coordinates and equivalent isotropic displacement parameters ($Å^2$) for **8** S150
Table S43. Anisotropic displacement parameters ($Å^2$) for **8** S152
Table S44. Distances [Å] for **8** S153
Table S45. Angles [°] for **8** S154
8.7 Crystal data for [PC(CH_3)P]PdCl (**9**) S156
Figure S79. Thermal-ellipsoid representation of [PC(CH_3)P]PdCl (**9**) S156
Table S46. Crystal data and structure refinement for **9** S157
Table S47. Atomic coordinates and equivalent isotropic displacement parameters ($Å^2$) for **9** S158
Table S48. Anisotropic displacement parameters ($Å^2$) for **9** S162
Table S49. Distances [Å] for **9** S164
Table S50. Angles [°] for **9** S166
8.8 Crystal data for [PC(sp^3)HP]PdI (**11**) S170
Figure S80. Thermal-ellipsoid representation of [PC(sp^3)HP]PdI (**11**) S170
Table S51. Crystal data and structure refinement for **11** S171
Table S52. Atomic coordinates and equivalent isotropic displacement parameters ($Å^2$) for **11** S172
Table S53. Anisotropic displacement parameters ($Å^2$) for **11** S175
Table S54. Distances [Å] for **11** S177
Table S55. Angles [°] for **11** S179
8.9 Crystal data for [PC(sp^3)H_2P]$PdBr_2$·CH_2Cl_2 (**12·CH_2Cl_2**) S182
Figure S81. Thermal-ellipsoid representation of **12·CH_2Cl_2** S182
Table S56. Crystal data and structure refinement for **12·CH_2Cl_2** S183
Table S57. Atomic coordinates and equivalent isotropic displacement parameters ($Å^2$) for **12·CH_2Cl_2** S184
Table S58. Anisotropic displacement parameters ($Å^2$) for **12·CH_2Cl_2** S186
Table S59. Distances [Å] for **12·CH_2Cl_2** S187
Table S60. Angles [°] for **12·CH_2Cl_2** S188

1 X-ray data for compounds $\{2\}_2$, 3-6, 8, 9, 11, 12

X-Ray crystal structure of $\{[PC(sp^2)P]PdI\}_2\cdot½Et_2O$ ($\{2\}_2\cdot½Et_2O$). Single crystals were obtained as green blocks from a concentrated diethyl ether solution at −34 °C in the glovebox. Crystal and refinement data for **$\{2\}_2\cdot½Et_2O$**: $C_{104}H_{154}I_4OP_8Pd_4$; M_r =2601.23; Monoclinic; space group $P2_1/c$; a = 16.2343(6) Å; b = 15.9423(6) Å; c = 20.8753(8) Å; α = 90°; β = 94.7040(14)°; γ = 90°; V = 5384.6(4) $Å^3$; Z = 2; T = 120(2) K; λ = 0.71073 Å; μ = 1.967 mm^{-1}; d_{calc} = 1.604 $g\cdot cm^{-3}$; 131164 reflections collected; 9479 unique (R_{int} = 0.0363); giving R_1 = 0.0257, wR_2 = 0.0662 for 8389 data with [I>2σ(I)] and R_1 = 0.0314, wR_2 = 0.0733 for all 9479 data. Residual electron density ($e^-\cdot Å^{-3}$) max/min: 0.964/−1.540.

X-Ray crystal structure of $[PC^{\bullet}(sp^2)P]PdBr$ (3). Single crystals were obtained as dichroic orange-green rhomboids from a concentrated diethyl ether solution at −35 °C in the glovebox. Crystal and refinement data for **3**: $C_{25}H_{36}BrP_2Pd$; M_r =584.79; Monoclinic; space group $P2_1/n$; a = 12.5686(12) Å; b = 13.9781(13) Å; c = 14.9939(14) Å; α = 90°; β = 97.3300(18)°; γ = 90°; V = 2612.7(4) $Å^3$; Z = 4; T = 120(2) K; λ = 0.71073 Å; μ = 2.372 mm^{-1}; d_{calc} = 1.487 $g\cdot cm^{-3}$; 33653 reflections collected; 4601 unique (R_{int} = 0.0528); giving R_1 = 0.0258, wR_2 = 0.0542 for 3834 data with [I>2σ(I)] and R_1 = 0.0375, wR_2 = 0.0569 for all 4601 data. Residual electron density ($e^-\cdot Å^{-3}$) max/min: 0.617/−0.548.

X-Ray crystal structure of $[PC(sp^3)HP]PdBr$ (4). Single crystals were obtained as cream rhomboids from a concentrated diethyl ether solution at −35 °C in the glovebox. Crystal and refinement data for **4**: $C_{25}H_{37}BrP_2Pd$; M_r =585.80; Monoclinic; space group $P2_1/n$; a = 11.2438(8) Å; b = 13.7677(10) Å; c = 17.4246(12) Å; α = 90°; β = 106.9775(12)°; γ = 90°; V = 2579.8(3) $Å^3$; Z = 4; T = 120(2) K; λ = 0.71073 Å; μ = 2.402 mm^{-1}; d_{calc} = 1.508 $g\cdot cm^{-3}$; 33493 reflections collected; 4546 unique (R_{int} = 0.0524); giving R_1 = 0.0348, wR_2 = 0.0742 for 3801 data with [I>2σ(I)] and R_1 = 0.0463, wR_2 = 0.0776 for all 4546 data. Residual electron density ($e^-\cdot Å^{-3}$) max/min: 1.970/−1.106.

X-Ray crystal structure of $[PC^{\bullet}(sp^2)P]PdCl$ (5). Single crystals were obtained as green rhomboids from a concentrated diethyl ether solution at −35 °C in the glovebox. Crystal and refinement data for **5**: $C_{25}H_{36}ClP_2Pd$; M_r =540.33; Monoclinic; space group $P2_1/n$; a = 11.6639(11) Å; b = 13.5934(13) Å; c = 16.5680(16) Å; α = 90°; β = 105.1159(14)°; γ = 90°; V = 2536.0(4) $Å^3$; Z = 4; T = 120(2) K; λ = 0.71073 Å; μ = 0.973 mm^{-1}; d_{calc} = 1.415 $g\cdot cm^{-3}$; 36052 reflections collected; 4436 unique (R_{int} = 0.0430); giving R_1 = 0.0240, wR_2 = 0.0523 for 3928 data with [I>2σ(I)] and R_1 = 0.0298, wR_2 = 0.0541 for all 4436 data. Residual electron density ($e^-\cdot Å^{-3}$) max/min: 0.664/−0.356.

X-Ray crystal structure of $[PC(CH_2)P]Pd(PMe_3)$ (6). Single crystals were obtained as orange blocks from a concentrated *n*-pentane at −35 °C in the glovebox. Crystal and refinement data for **6**: $C_{29}H_{47}P_3Pd$; M_r =594.98; Monoclinic; space group $P2_1/n$; a = 10.3086(7) Å; b = 24.0617(16) Å; c = 12.5709(8) Å; α = 90°; β = 107.5771(18)°; γ = 90°; V = 2972.5(3) $Å^3$; Z = 4; T = 120(2) K; λ = 0.71073 Å; μ = 0.801 mm^{-1}; d_{calc} = 1.329 $g\cdot cm^{-3}$; 71407 reflections collected; 5233 unique (R_{int} = 0.0510); giving R_1 = 0.0220, wR_2 = 0.0495 for 4714 data with [I>2σ(I)] and R_1 = 0.0257, wR_2 = 0.0512 for all 5233 data. Residual electron density ($e^-\cdot Å^{-3}$) max/min: 0.407/−0.292.

X-Ray crystal structure of [PC(CH_3)HP]$PdCl_2$ (8). Single crystals were obtained as yellow needles from a concentrated dichloromethane solution at −35 °C in the glovebox. Crystal and refinement data for **8**: $C_{26}H_{40}Cl_2P_2Pd$; M_r =591.82; Monoclinic; space group $C2/c$; a = 13.0569(14) Å; b = 16.8080(14) Å; c = 31.261(3) Å; α = 90°; β = 93.209(2)°; γ = 90°; V = 6849.8(11) $Å^3$; Z = 8; T = 120(2) K; λ = 0.71073 Å; μ = 0.801 mm^{-1}; d_{calc} = 1.148 $g{\cdot}cm^{-3}$; 38696 reflections collected; 6002 unique (R_{int} = 0.0536); giving R_1 = 0.0372, wR_2 = 0.0751 for 5140 data with [I>2σ(I)] and R_1 = 0.0457, wR_2 = 0.0779 for all 6002 data. Residual electron density ($e^-{\cdot}Å^{-3}$) max/min: 0.596/−0.503.

X-Ray crystal structure of [PC(CH_3)P]PdCl (9). Single crystals were obtained as yellow cubes from a concentrated diethyl ether solution at −35 °C in the glovebox. Crystal and refinement data for **9**: $C_{26}H_{39}ClP_2Pd$; M_r =555.36; Monoclinic; space group $P2_1/c$; a = 11.2685(8) Å; b = 20.7303(14) Å; c = 23.1605(16) Å; α = 90°; β = 99.2340(13)°; γ = 90°; V = 5340.2(6) $Å^3$; Z = 8; T = 120(2) K; λ = 0.71073 Å; μ = 0.926 mm^{-1}; d_{calc} = 1.382 $g{\cdot}cm^{-3}$; 62725 reflections collected; 9411 unique (R_{int} = 0.0607); giving R_1 = 0.0419, wR_2 = 0.0827 for 7826 data with [I>2σ(I)] and R_1 = 0.0557, wR_2 = 0.0867 for all 9411 data. Residual electron density ($e^-{\cdot}Å^{-3}$) max/min: 0.851/−0.547.

X-Ray crystal structure of [PC(sp^3)HP]PdI (11). Single crystals were obtained as light yellow plates from a concentrated diethyl ether solution at −35 °C in the glovebox. Crystal and refinement data for **11**: $C_{25}H_{37}IP_2Pd$; M_r =632.79; Monoclinic; space group $C2/c$; a = 29.176(6) Å; b = 12.515(2) Å; c = 22.247(4) Å; α = 90°; β = 105.315(4)°; γ = 90°; V = 7835(2) $Å^3$; Z = 12; T = 120(2) K; λ = 0.71073 Å; μ = 2.025 mm^{-1}; d_{calc} = 1.609 $g{\cdot}cm^{-3}$; 55501 reflections collected; 6893 unique (R_{int} = 0.0219); giving R_1 = 0.0235, wR_2 = 0.0511 for 6564 data with [I>2σ(I)] and R_1 = 0.0252, wR_2 = 0.0519 for all 6893 data. Residual electron density ($e^-{\cdot}Å^{-3}$) max/min: 1.412/−1.244.

X-Ray crystal structure of [PC(sp^3)H_2P]$PdBr_2{\cdot}CH_2Cl_2$ (12·CH_2Cl_2). Single crystals were obtained as orange cubes from a concentrated dichloromethane solution at −35 °C in the glovebox. Crystal and refinement data for **12·CH_2Cl_2**: $C_{51}H_{78}Br_4Cl_2P_4Pd_2$; M_r =1418.35; Monoclinic; space group $P2_1/n$; a = 10.3876(9) Å; b = 16.2993(14) Å; c = 17.0895(15) Å; α = 90°; β = 103.4770(14)°; γ = 90°; V = 2813.8(4) $Å^3$; Z = 2; T = 120(2) K; λ = 0.71073 Å; μ = 3.721 mm^{-1}; d_{calc} = 1.674 $g{\cdot}cm^{-3}$; 50587 reflections collected; 4956 unique (R_{int} = 0.0399); giving R_1 = 0.0185, wR_2 = 0.0423 for 4411 data with [I>2σ(I)] and R_1 = 0.0239, wR_2 = 0.0435 for all 4956 data. Residual electron density ($e^-{\cdot}Å^{-3}$) max/min: 0.371/−0.325.

2 Magnetic moment for {[PC(sp^2)P]PdI}$_2$ ({2}$_2$)

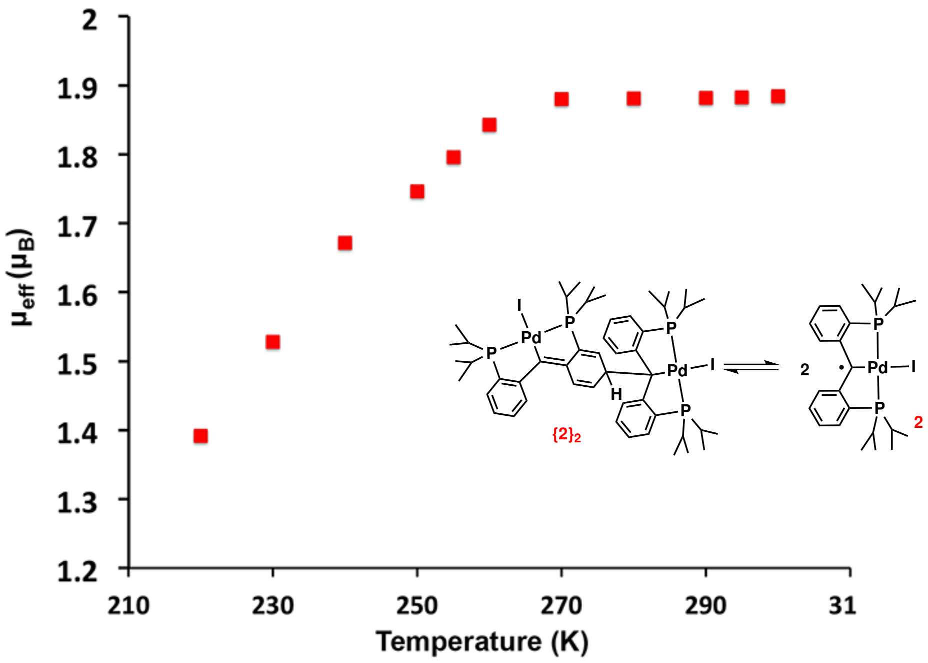

Figure S1. Magnetic moment for **{2}$_2$** as a function of temperature (Evans method, C_6D_6).

3 Cyclic Voltammetry

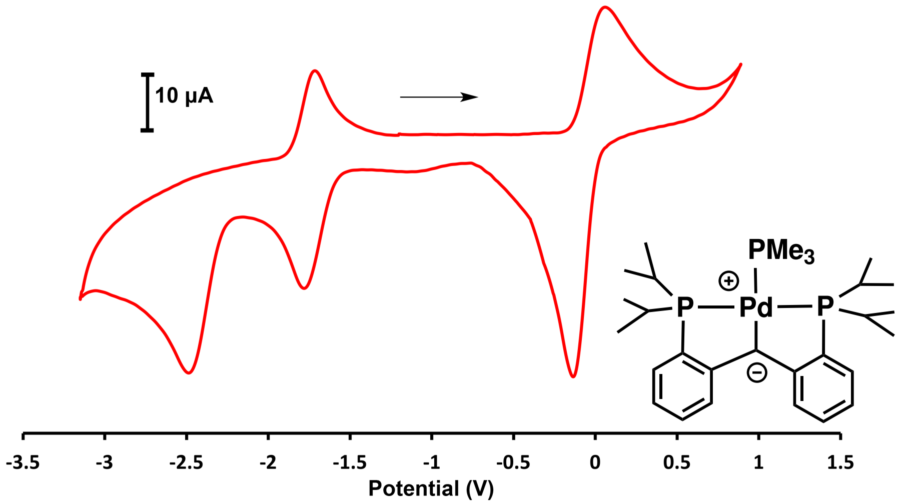

Figure S2. Cyclic voltammogram of [PC(sp^2)P]Pd(PMe_3) (**1**) at 100 mV/s, 1 mM in 0.1 M [^{n}Bu$_4$N][PF_6] in THF, Cp_2Fe/Cp_2Fe^+ corrected.

4 EPR Spectra

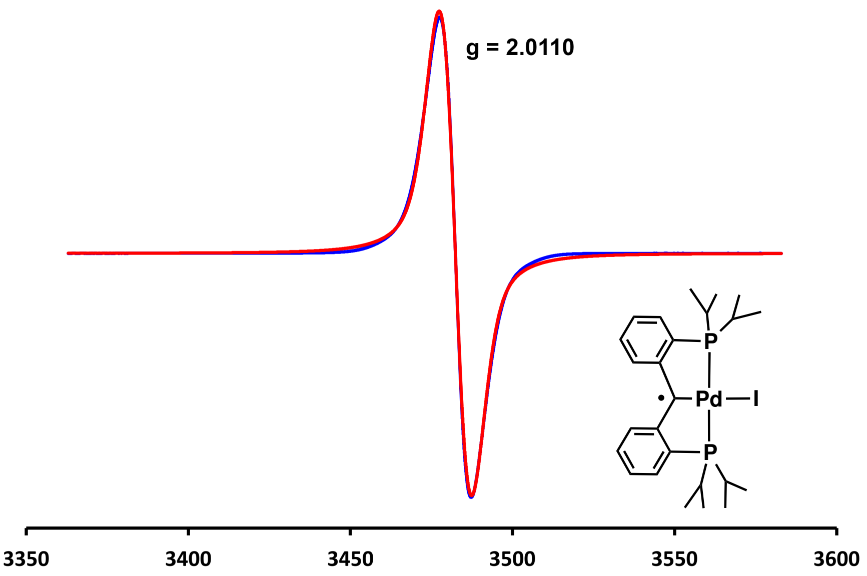

Figure S3. EPR spectrum of [PC$^{\bullet}$(sp^2)P]PdI (**2**) (1 mM solution in toluene, 298 K), ΔH_{pp} = 10.0 G; the blue line represents the experimental data and the red line represents the simulated spectrum.

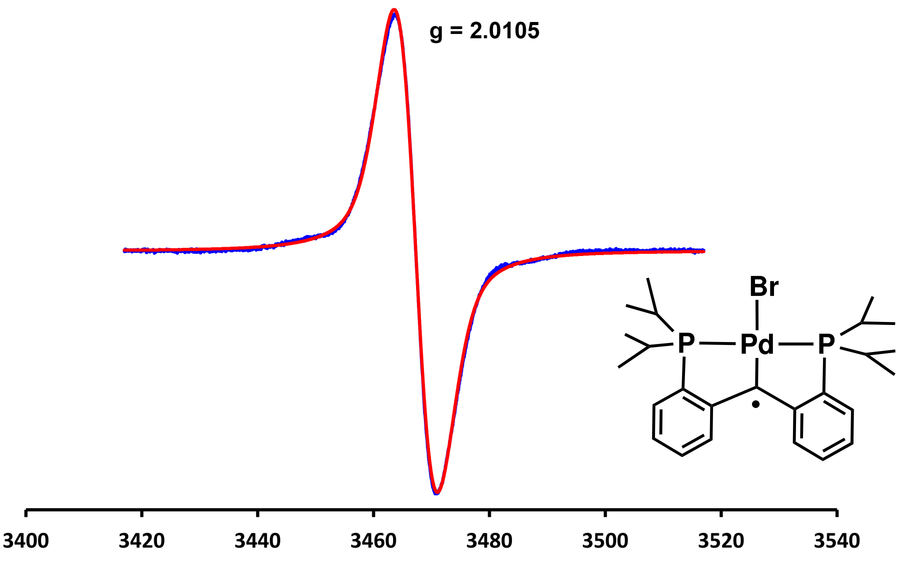

Figure S4. EPR spectrum of [PC$^{\bullet}$(sp^2)P]PdBr (**3**) (1 mM solution in toluene, 298 K), ΔH_{pp} = 7.5 G; the blue line represents the experimental data and the red line represents the simulated spectrum.

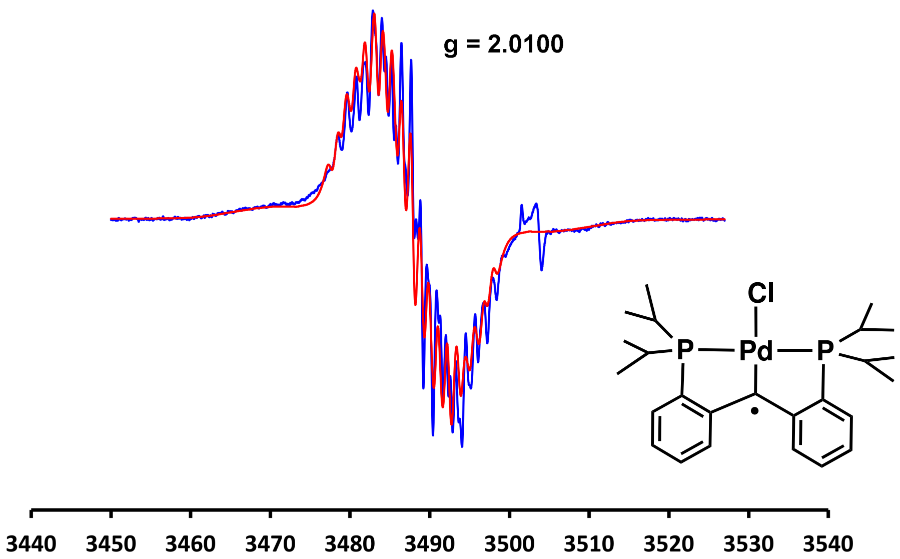

Figure S5. EPR spectrum of [PC•(sp^2)P]PdCl (**5**) at 298 K(100 μM solution in toluene); the blue line represents the experimental data and the red line represents the simulation with the following parameters: a_1(2H) = 4.5 G; a_2(2H) = 2.6 G; a_3(2H) = 2.1 G; a_4(2H) = 1.2 G; g = 2.0100, individual linewidth 0.8 G (lorentzian lineshape), relative intensity 77.67% for the nonmagnetic Pd isotope species; a_1(2H) = 4.5 G, a_2(2H) = 2.6 G, a_3(2H) = 2.1 G, a_4(2H) = 1.2 G, a(^{105}Pd) = 6.0 G, individual linewidth 1.7 G, relative intensity 22.33% for the ^{105}Pd species. The small satellite peak on the right comes from the quartz sample tube.

5 Complete Reference 24

24. M. J. Frisch, G. W. Trucks, H. B. Schlegel, G. E. Scuseria, M. A. Robb, J. R. Cheeseman, J. A. Montgomery, T. Vreven, K. N. Kudin, J. C. Burant, J. M. Millam, S. S. Iyengar, J. Tomasi, V. Barone, B. Mennucci, M. Cossi, G. Scalmani, N. Rega, G. A. Petersson, H. Nakatsuji, M. Hada, M. Ehara, K. Toyota, R. Fukuda, J. Hasegawa, M. Ishida, T. Nakajima, Y. Honda, O. Kitao, H. Nakai, M. Klene, X. Li, J. E. Knox, H. P. Hratchian, J. B. Cross, V. Bakken, C. Adamo, J. Jaramillo, R. Gomperts, R. E. Stratmann, O. Yazyev, A. J. Austin, R. Cammi, C. Pomelli, J. W. Ochterski, P. Y. Ayala, K. Morokuma, G. A. Voth, P. Salvador, J. J. Dannenberg, V. G. Zakrzewski, S. Dapprich, A. D. Daniels, M. C. Strain, O. Farkas, D. K. Malick, A. D. Rabuck, K. Raghavachari, J. B. Foresman, J. V. Ortiz, Q. Cui, A. G. Baboul, S. Clifford, J. Cioslowski, B. B. Stefanov, G. Liu, A. Liashenko, P. Piskorz, I. Komaromi, R. L. Martin, D. J. Fox, T. Keith, M. A. Al-Laham, C. Y. Peng, A. Nanayakkara, M. Challacombe, P. M. W. Gill, B. Johnson, W. Chen, M. W. Wong, C. Gonzalez, and J. A. Pople, *Gaussian, Inc.*, Wallingford CT, 2004.

6 DFT Results

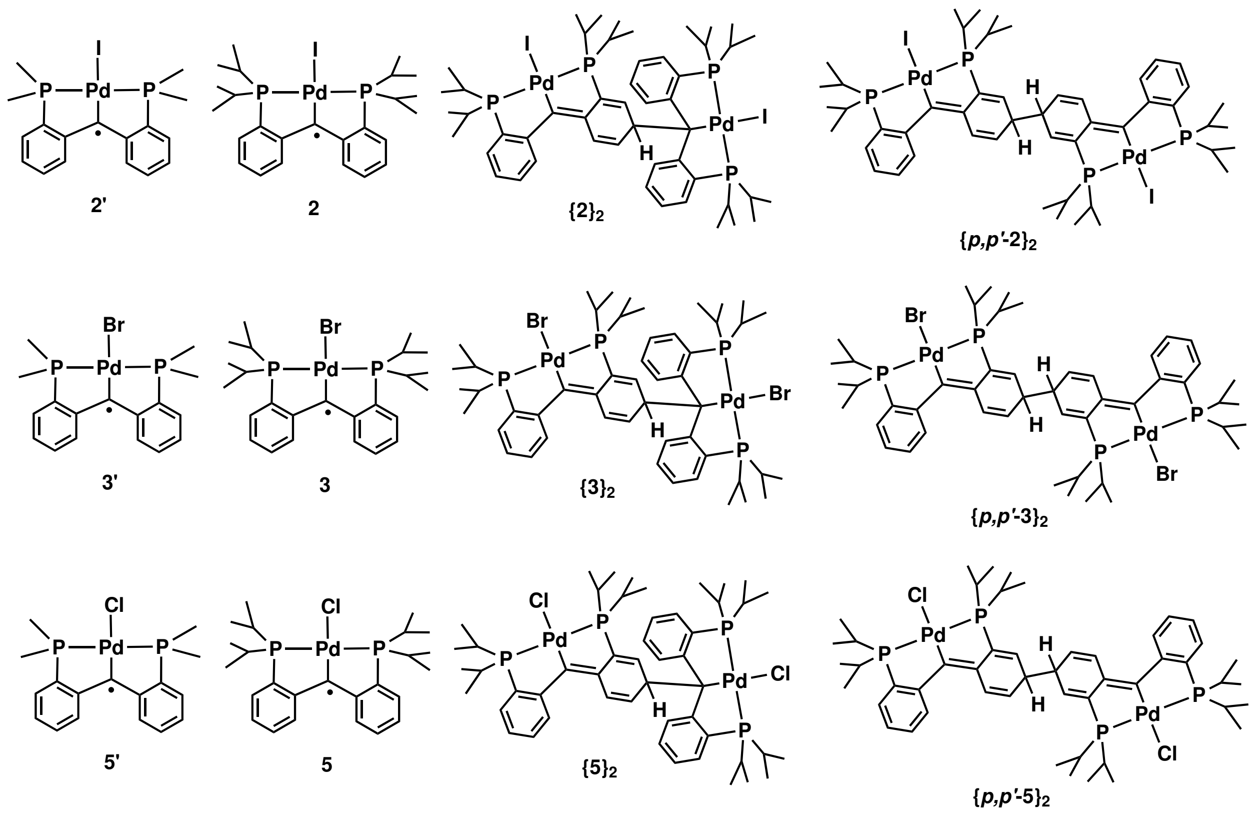

Figure S6. Computed molecules.

6.1 {[PC(sp^2)P]PdI}$_2$ ({**2**}$_2$)

Table S1. Optimized coordinates for {[PC(sp^2)P]PdI}$_2$ ({**2**}$_2$).

atom	x	y	z
Pd	3.782800	−0.200187	−0.256172
P	4.026958	1.407714	−2.076560
P	4.090199	−1.009851	1.941575
C	2.559016	1.337554	0.638793
I	4.962089	−2.438263	−1.442177
C	5.811178	−0.681159	2.733384
H	5.671573	−0.873930	3.807701
C	6.216618	0.796881	2.550489
H	5.461461	1.484439	2.948748
H	6.369166	1.033918	1.491427
H	7.160582	0.984778	3.080369
C	6.888091	−1.637611	2.176198
H	7.856604	−1.404763	2.639792
H	6.990430	−1.528434	1.090616
H	6.663411	−2.691010	2.381565
C	3.702958	−2.852073	2.328847
H	4.382629	−3.372420	1.641145
C	2.257122	−3.178261	1.908239
H	2.094186	−2.943585	0.850759
C	2.684636	−0.051193	4.277140
H	1.534846	−2.618426	2.515763
H	2.063453	−4.250070	2.050888
C	3.988351	−3.323268	3.772677
H	3.250192	−2.929142	4.481093
H	3.917277	−4.419259	3.807117
H	4.990639	−3.051618	4.124174
C	5.820961	1.635020	−2.752956
H	5.825726	2.634763	−3.214598
C	6.165718	0.584616	−3.832362
H	5.478167	0.609397	−4.687074
H	6.153587	−0.426403	−3.412092
H	7.175641	0.783969	−4.216965
C	6.848500	1.603411	−1.602743
H	6.640815	2.365765	−0.842958
H	6.851737	0.618288	−1.121712
H	7.854379	1.787914	−2.004608
C	2.968265	1.641183	−3.694576

Continued on next page

Table S1. – continued from previous page

atom	x	y	x
H	3.718309	1.832994	−4.473787
C	1.994385	2.838490	−3.663845
H	1.489276	2.912572	−4.637253
H	2.506288	3.788955	−3.477264
H	1.218168	2.720511	−2.899452
C	2.241830	0.318001	−4.032969
H	1.772090	0.394241	−5.023461
H	1.451405	0.102463	−3.302746
H	2.926101	−0.538195	−4.038317
C	2.922099	0.096344	2.893011
C	2.267483	1.102617	2.137631
C	1.318196	1.904805	2.825823
H	0.772387	2.665260	2.279954
C	1.055275	1.736495	4.194531
H	0.315403	2.368367	4.680838
C	1.751985	0.764700	4.936347
H	1.567070	0.640004	6.000370
H	3.224181	−0.803798	4.844258
C	1.192833	1.310565	−0.219442
H	1.552957	1.294799	−1.259680
C	3.280604	2.667711	0.341065
C	3.816368	2.914223	−0.961055
C	4.317411	4.182960	−1.305095
C	4.369929	5.223691	−0.358986
C	3.925531	4.970665	0.949544
C	3.382765	3.717011	1.286766
H	3.033830	3.560874	2.300772
H	3.990730	5.747310	1.708478
H	4.775733	6.194484	−0.631772
H	4.710759	4.359579	−2.303869
C	0.365078	0.049880	−0.053054
C	0.322633	2.551157	−0.115809
H	0.817456	3.507358	−0.276287
H	0.922813	−0.883171	−0.094499
C	−0.989985	0.042507	0.062781
C	−1.793245	1.275150	0.214399
C	−1.020529	2.523346	0.079688
H	−1.569534	3.460306	0.062453
C	−3.167787	1.213781	0.427758
C	−3.967079	2.375233	0.905183
C	−5.355020	2.474820	0.589982
C	−6.118173	3.576390	1.016269

Continued on next page

Table S1. – continued from previous page

atom	x	y	x
C	−5.543881	4.583395	1.814591
C	−4.197086	4.460157	2.206975
C	−3.425342	3.374111	1.765108
H	−2.404086	3.273174	2.116522
H	−3.753031	5.201199	2.868184
H	−6.144124	5.424390	2.152375
H	−7.172435	3.643612	0.759785
P	−2.055023	−1.509555	0.074036
Pd	−4.201216	−0.504744	−0.046412
P	−6.073383	0.973072	−0.273740
I	−5.525091	−2.819056	−0.847462
C	−7.735101	0.663962	0.638474
H	−8.223248	1.646341	0.717173
C	−8.656712	−0.297359	−0.143684
H	−8.178547	−1.272870	−0.283962
H	−9.584581	−0.449029	0.425161
H	−8.933056	0.094666	−1.130324
C	−6.528418	1.493449	−2.065205
C	−5.237817	1.808139	−2.853079
H	−4.537596	0.965593	−2.824630
H	−4.727924	2.689390	−2.442164
H	−5.485768	2.017655	−3.902552
C	−7.535707	2.661255	−2.142258
H	−7.102619	3.583210	−1.735103
H	−7.792212	2.853105	−3.193310
H	−8.469942	2.449108	−1.608058
C	−7.444349	0.134102	2.060570
H	−6.954757	−0.845939	2.012320
H	−6.800744	0.815825	2.630236
H	−8.388002	0.018260	2.611160
C	−1.568723	−2.503766	1.635648
H	−0.484225	−2.655614	1.565908
C	−1.875780	−1.675464	2.901722
H	−1.561270	−2.235847	3.793139
H	−1.347897	−0.714317	2.900402
H	−2.951494	−1.477097	2.985307
C	−2.280018	−3.875091	1.664057
H	−1.996690	−4.413259	2.579338
H	−3.369600	−3.756817	1.658384
H	−6.983426	0.587560	−2.488316
C	−1.591557	−2.590987	−1.444179
H	−2.359693	−3.375438	−1.409016

Continued on next page

Table S1. – continued from previous page

atom	x	y	x
C	−1.801231	−1.780503	−2.741701
H	−1.107568	−0.931323	−2.799930
H	−2.827502	−1.402773	−2.808948
H	−1.619524	−2.427639	−3.610286
C	−0.193075	−3.244942	−1.400166
H	−0.009104	−3.793526	−0.468856
H	0.610359	−2.511732	−1.535929
H	−0.111758	−3.966356	−2.224917
H	−2.010096	−4.505164	0.807950

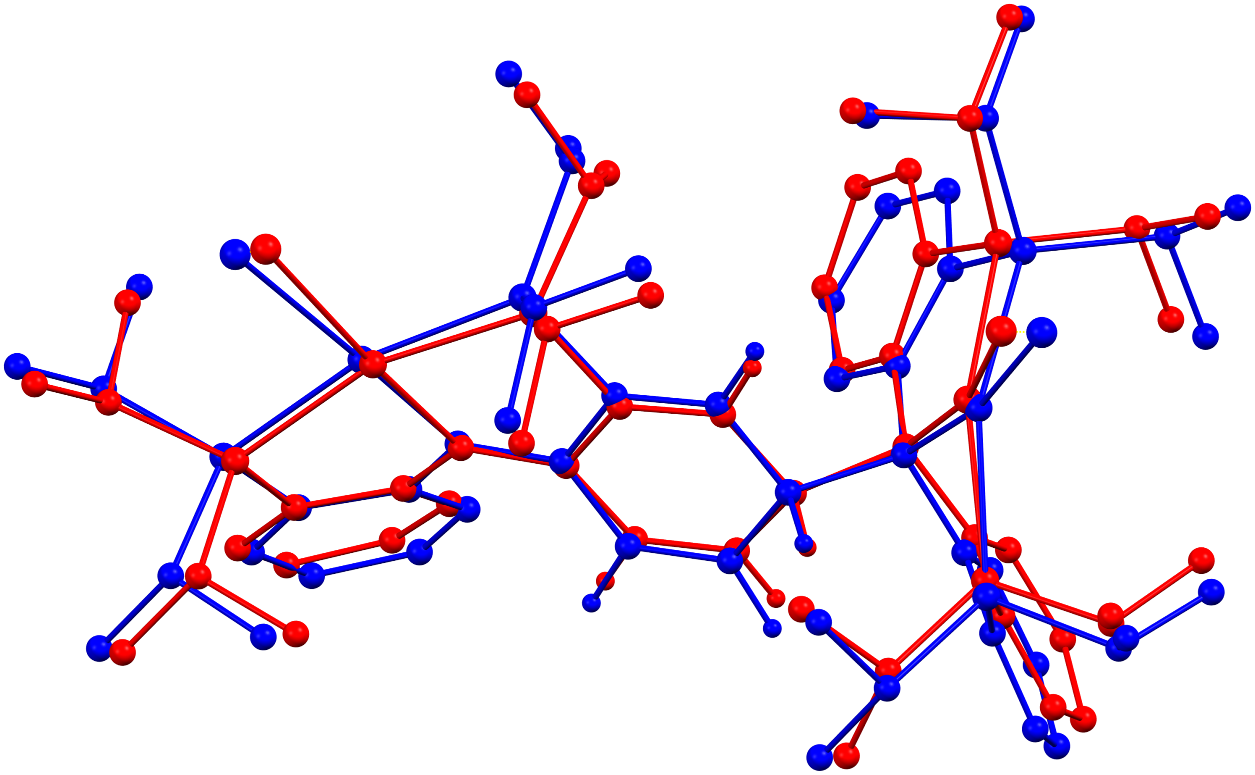

Figure S7. Overlaid structures for {[PC(sp^2)P]PdI}$_2$ (**{2}$_2$**) (red: X-ray, blue: optimized).

Table S2. Selected distances (Å) and angles (°) for the optimized geometry and the crystal structure of {[PC(sp^2)P]PdI}$_2$ (**{2}$_2$**).

Distance	Calcd.	X-Ray	Angle	Calcd.	X-Ray
Pd(1) – I(1)	2.794	2.6851(3)	I(1) – Pd(1) – C(1)	170.24	167.42(8)
Pd(2) – I(2)	2.784	2.6868(3)	I(2) – Pd(2) – C(2)	176.37	177.19(9)
Pd(1) – C(1)	2.159	2.127(3)	I(1) – Pd(1) – P(11)	99.72	100.04(2)
Pd(2) – C(2)	2.061	2.043(3)	I(1) – Pd(1) – P(12)	93.75	95.99(2)
Pd(1) – P(11)	2.441	2.3359(9)	I(2) – Pd(2) – P(21)	95.32	94.63(2)
Pd(1) – P(12)	2.362	2.2686(9)	I(2) – Pd(2) – P(22)	96.54	98.75(2)
Pd(2) – P(21)	2.373	2.2728(8)	P(11) – Pd(1) – P(12)	155.03	155.64(3)
Pd(2) – P(22)	2.396	2.3104(9)	P(11) – Pd(1) – C(1)	84.07	83.20(9)
C(1) – C(54)	1.614	1.588(4)	P(21) – Pd(2) – P(22)	166.60	163.58(3)
C(1) – C(11)	1.542	1.529(4)	P(21) – Pd(2) – C(2)	83.56	82.65(9)
C(1) – C(21)	1.545	1.530(4)	P(12) – Pd(1) – C(1)	86.11	85.13(9)
C(54) – C(53)	1.517	1.496(4)	P(22) – Pd(2) – C(2)	84.22	84.06(9)
C(53) – C(52)	1.360	1.345(4)	C(51) – C(2) – C(61)	123.01	123.6(3)
C(52) – C(51)	1.479	1.465(4)	C(21) – C(1) – C(54)	110.71	109.1(3)
C(51) – C(56)	1.474	1.465(4)	C(11) – C(1) – C(54)	107.93	107.8(2)
C(56) – C(55)	1.358	1.339(4)	C(11) – C(1) – C(21)	114.00	115.5(3)
C(55) – C(54)	1.519	1.508(4)	Pd(1) – C(1) – C(54)	104.33	103.74(19)
C(2) – C(51)	1.392	1.380(4)	C(51) – C(2) – Pd(2)	119.78	120.8(2)
C(2) – C(61)	1.489	1.481(4)	C(61) – C(2) – Pd(2)	117.08	115.6(2)

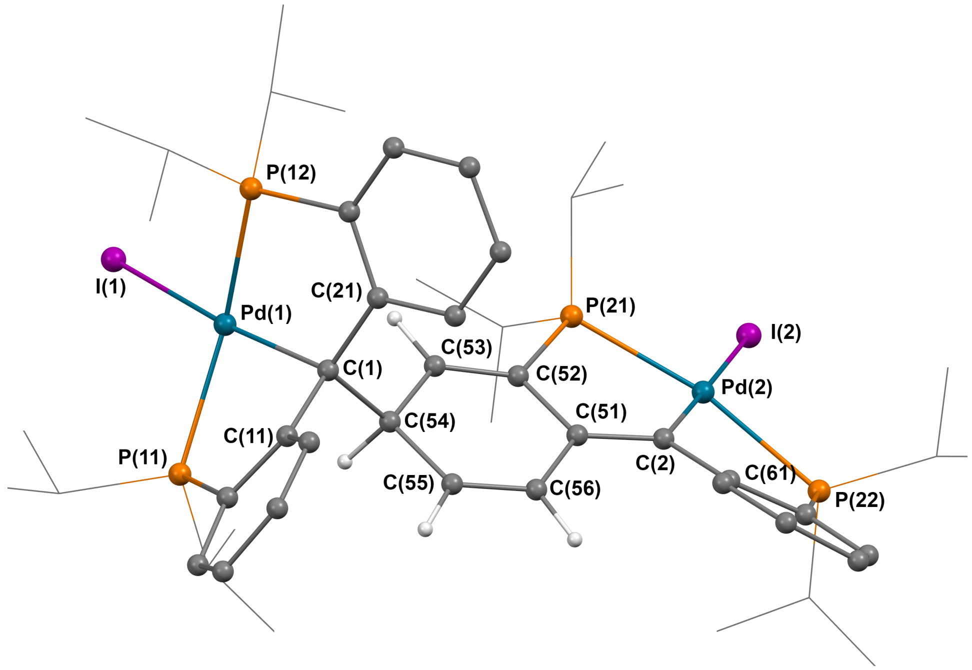

Figure S8. Optimized geometry for {[PC(sp^2)P]PdI}$_2$ (**{2}$_2$**).

6.2 {*p,p'*-[PC(sp^2)P]PdI}$_2$ ({*p,p'*-2}$_2$)

Table S3. Optimized coordinates for {*p,p'*-[PC(sp^2)P]PdI}$_2$ (**{*p,p'*-2}$_2$**).

atom	x	y	z
C	−6.504565	2.938936	−0.215188
C	−5.144848	2.543000	−0.373098
C	−4.243832	3.516065	−0.896617
C	−4.653831	4.834257	−1.148796
C	−5.982300	5.228211	−0.894610
C	−6.905054	4.267314	−0.440585
C	−4.731525	1.143955	−0.080733
C	−3.426699	0.800089	0.264217
C	−2.979018	−0.606621	0.223070
C	−1.680873	−0.970150	0.397014
C	−0.561663	0.010605	0.682607
C	−1.113675	1.383522	0.989959
C	−2.401866	1.744303	0.750285
P	−4.369513	−1.805310	−0.174151
C	−3.861938	−2.642745	−1.820844
C	−4.797039	−3.822047	−2.168257
P	−7.699124	1.550877	0.189606
C	−9.118164	1.851080	−1.077246
C	−8.613055	1.487867	−2.491746
Pd	−6.204573	−0.302361	−0.104061
I	−8.130067	−2.314697	−0.082570
C	−4.402762	−3.173161	1.171112
C	−3.178795	−4.116471	1.190870
C	−8.418772	1.966759	1.929043
C	−7.308772	2.497050	2.863948
C	−9.115908	0.737554	2.557261
C	−10.416447	1.091825	−0.731032
C	−4.643035	−2.528586	2.554218
C	−3.839052	−1.587961	−2.949091
H	−7.941708	4.560661	−0.287722
H	−6.300935	6.250967	−1.079404
H	−3.941748	5.550424	−1.552796
H	−3.227043	3.224205	−1.135805
H	−6.516930	1.748304	2.996641
H	−6.848980	3.416152	2.485123
H	−7.738144	2.710450	3.852294
H	−9.159438	2.764130	1.761300

Continued on next page

Table S3. – continued from previous page

atom	x	y	x
H	−9.544596	1.021441	3.528573
H	−9.918766	0.334345	1.933802
H	−8.398164	−0.074133	2.721317
H	−9.316613	2.932491	−1.035073
H	−10.857376	1.431417	0.213721
H	−11.155874	1.270165	−1.524026
H	−10.239085	0.012437	−0.669043
H	−9.388337	1.728166	−3.232030
H	−8.397605	0.414826	−2.558269
H	−7.704982	2.041784	−2.759201
H	−5.298090	−3.749394	0.900940
H	−3.801895	−1.886811	2.847269
H	−4.751487	−3.315628	3.312656
H	−5.558438	−1.926639	2.556236
H	−2.280387	−3.602715	1.552884
H	−3.380328	−4.946659	1.882053
H	−2.960530	−4.554015	0.208949
H	−2.839028	−3.016306	−1.663326
H	−3.164644	−0.754375	−2.718861
H	−4.842520	−1.177521	−3.116195
H	−3.499527	−2.054789	−3.883707
H	−4.758793	−4.621506	−1.418635
H	−4.497119	−4.252154	−3.133643
H	−5.838463	−3.489995	−2.248086
H	−2.723795	2.748702	1.010580
H	−0.435569	2.104909	1.442607
H	−1.389849	−2.013791	0.312909
H	0.024864	−0.352333	1.542992
C	0.498783	0.042453	−0.537846
C	1.045446	−1.333837	−0.840886
H	−0.086691	0.404751	−1.398958
C	1.622769	1.017853	−0.252320
C	2.333173	−1.699775	−0.607015
C	2.918068	0.647882	−0.077124
C	3.366483	−0.756454	−0.138484
H	0.362800	−2.054275	−1.287995
H	1.337813	2.065861	−0.173869
H	2.647985	−2.706245	−0.867338
C	4.688925	−1.093682	0.140287
P	4.307167	1.858541	0.307908
C	5.127236	−2.494173	0.381314
C	6.472927	−2.886365	0.114307

Continued on next page

Table S3. – continued from previous page

atom	x	y	x
C	6.873646	−4.229079	0.238917
C	5.978358	−5.202797	0.720305
C	4.679217	−4.811748	1.099891
C	4.261127	−3.482592	0.933436
P	7.624913	−1.474522	−0.312923
H	7.886441	−4.523980	−0.021342
H	6.298481	−6.235979	0.828475
H	3.994355	−5.541009	1.526905
H	3.265465	−3.195884	1.254483
Pd	6.147696	0.363220	0.053007
C	3.902076	2.591340	2.043004
C	4.010631	3.264258	−0.973613
C	4.761446	4.571443	−0.644126
C	4.374567	2.744062	−2.382582
H	2.927894	3.455813	−0.933702
C	5.134057	3.309728	2.642530
H	3.092002	3.319032	1.878663
C	3.405602	1.488152	3.003621
C	8.296092	−1.874314	−2.064593
C	9.123641	−1.538804	0.885706
H	9.663279	−0.618204	0.625010
C	8.629410	−1.406133	2.342846
C	10.067406	−2.749286	0.713153
C	9.421307	−0.901448	−2.480349
H	8.698771	−2.896723	−2.001595
C	7.132522	−1.860895	−3.080021
I	8.169295	2.271460	−0.100392
H	7.503824	−2.160269	−4.069471
H	6.330155	−2.551572	−2.794021
H	6.704242	−0.854837	−3.167032
H	9.759565	−1.148891	−3.495978
H	9.066278	0.135370	−2.477581
H	10.291591	−0.956232	−1.815323
H	4.124394	3.507676	−3.131808
H	5.449130	2.537953	−2.450051
H	3.827173	1.827862	−2.635239
H	4.580303	5.298570	−1.447870
H	4.417072	5.023540	0.293586
H	5.841758	4.401348	−0.578544
H	10.977502	−2.579772	1.304899
H	9.607745	−3.673604	1.082440
H	10.377039	−2.903303	−0.327452

Continued on next page

Table S3. – continued from previous page

**atom**	**x**	**y**	**x**
H	9.492763	−1.344437	3.019076
H	8.030145	−0.498515	2.475127
H	8.024548	−2.271936	2.642270
H	4.863106	3.743123	3.615441
H	5.956213	2.601743	2.798220
H	5.516720	4.112263	2.005959
H	3.193470	1.930916	3.986321
H	2.492770	1.002741	2.642297
H	4.172837	0.715470	3.141101

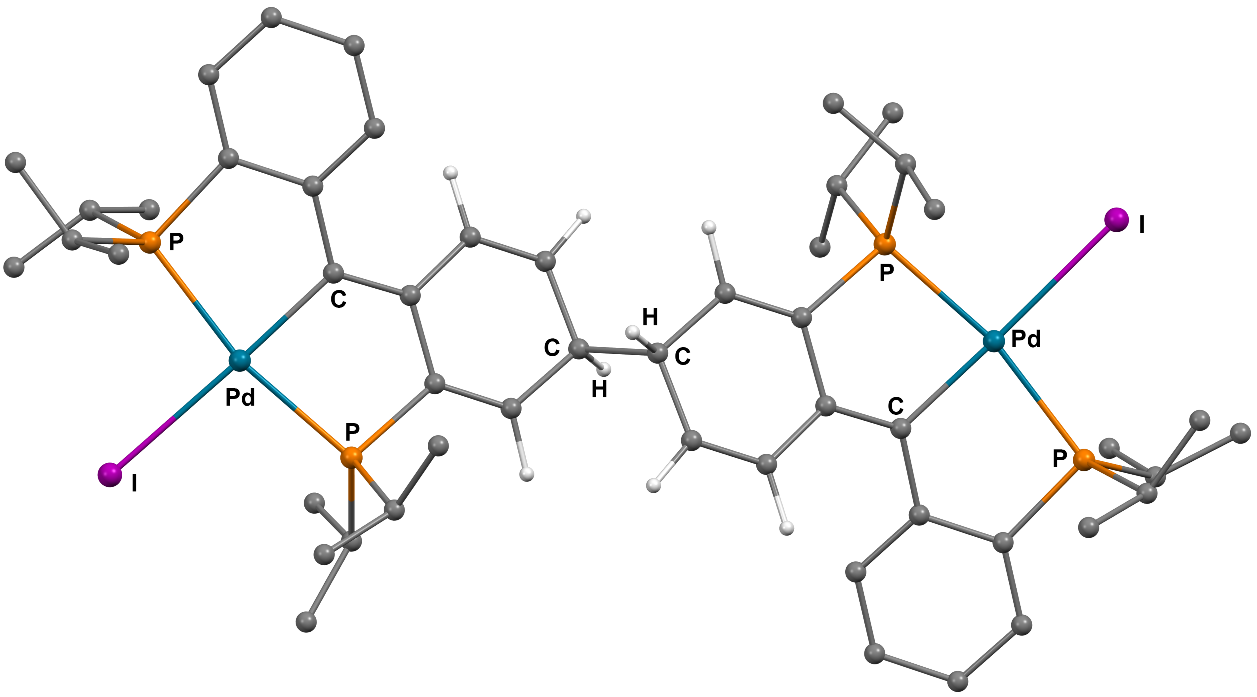

Figure S9. Optimized geometry for {*p,p'*-[PC(sp^2)P]PdI}$_2$ (**{*p,p'*-2}$_2$**).

6.3 $[PC^{\bullet}(sp^2)P]PdI$ (2)

Table S4. Optimized coordinates for $[PC^{\bullet}(sp^2)P]PdI$ (**2**).

atom	x	y	z
Pd	−0.047428	−0.336933	−0.021538
P	2.314178	−0.111216	−0.242191
P	−2.408665	−0.031872	0.252013
C	−0.015497	1.711373	−0.025248
I	−0.012672	−3.123864	−0.015889
C	1.234436	2.397589	0.305717
C	2.485282	1.702763	0.183669
C	3.706161	2.359810	0.402858
C	3.735091	3.703391	0.828783
C	2.515566	4.377781	1.050342
C	1.293030	3.745105	0.790881
H	0.369476	4.277140	0.993148
H	2.521079	5.398067	1.427987
H	4.684022	4.202265	1.007824
H	4.644528	1.833033	0.253272
C	−1.240699	2.425462	−0.389895
C	−1.255048	3.752748	−0.930795
C	−2.454871	4.395813	−1.261877
C	−3.694353	3.750346	−1.067033
C	−3.706489	2.427687	−0.579017
C	−2.508874	1.765863	−0.271768
H	−4.660794	1.919098	−0.457630
H	−4.625503	4.254142	−1.313199
H	−2.426501	5.398750	−1.682504
H	−0.314929	4.257147	−1.126741
C	−3.181593	−0.127689	2.015115
C	−2.764100	−1.436203	2.726011
C	−2.774311	1.101886	2.856756
H	−1.683070	1.157021	2.963408
H	−3.121474	2.041954	2.414576
H	−3.207327	1.014285	3.862547
H	−4.272451	−0.119097	1.866410
H	−3.252454	−1.488497	3.709008
H	−3.030742	−2.334556	2.161946
H	−1.678893	−1.462049	2.878093
C	−3.652360	−0.892521	−0.937133
C	−4.042252	−2.316526	−0.486624

Continued on next page

Table S4. – continued from previous page

atom	x	y	x
C	−3.067761	−0.893992	−2.367384
H	−4.551028	−0.257457	−0.917949
H	−4.562875	−2.319806	0.478529
H	−4.723385	−2.751704	−1.231064
H	−3.161141	−2.963906	−0.413881
H	−3.809627	−1.301689	−3.067644
H	−2.171573	−1.523839	−2.414154
H	−2.801718	0.115486	−2.703764
C	3.326479	−1.135174	1.027466
C	3.168314	−0.337316	−1.944102
H	3.069481	−2.166093	0.749269
C	2.785358	−0.874738	2.450487
H	2.948157	0.166805	2.757184
H	3.305694	−1.525528	3.166217
H	1.713506	−1.094085	2.510691
C	4.860185	−0.958781	0.960112
H	5.170772	0.013813	1.359208
H	5.335054	−1.731222	1.580692
H	5.258220	−1.064300	−0.056503
C	2.415096	0.480102	−3.016215
H	4.180786	0.078520	−1.829423
C	3.262448	−1.830179	−2.329213
H	2.356231	1.543471	−2.754478
H	1.393504	0.102557	−3.147309
H	2.937381	0.394056	−3.978750
H	3.871136	−2.407599	−1.622967
H	3.722952	−1.922379	−3.322394
H	2.268731	−2.291364	−2.365452

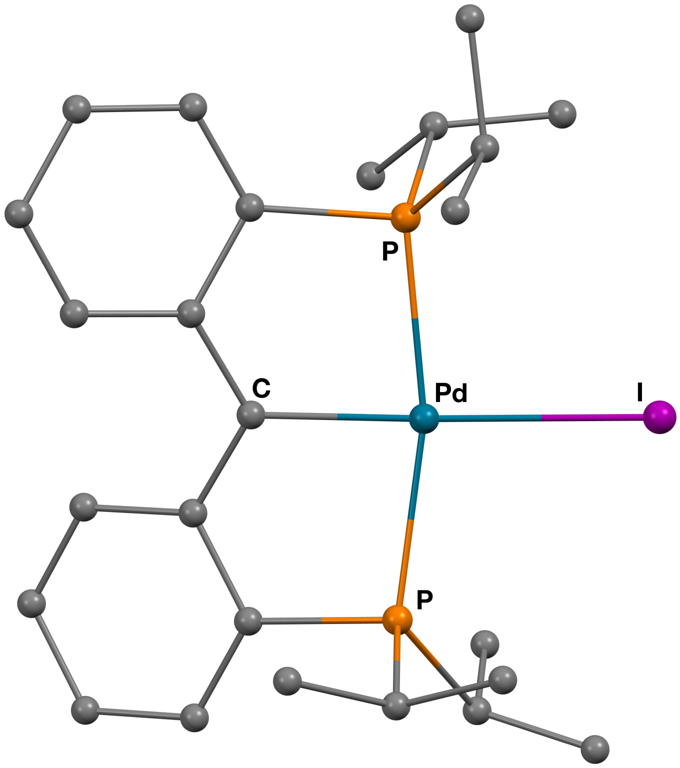

Figure S10. Optimized geometry for [PC•(sp^2)P]PdI (**2**).

6.4 Calculated energies for [PC•(sp^2)P]PdI (2) and corresponding dimers

		Energy (hartree)	Energy loss (kcal/mol)
[PC•(sp^2)P]PdI (**2**)	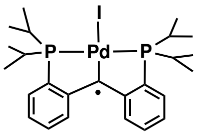	−11125.396903	—
{[PC(sp^2)P]PdI}$_2$ (**{2}$_2$**)	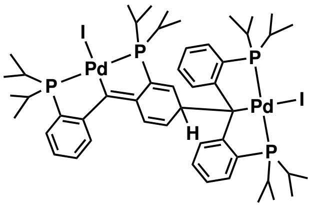	−2250.785894	4.96
{*p,p'*-[PC(sp^2)P]PdI}$_2$ (**{*p,p'*-2}$_2$**)	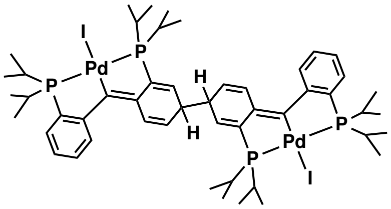	−2250.76912	14.49

6.5 [$Me_2PC^{\bullet}(sp^2)PMe_2$]PdI (2')

Table S5. Optimized coordinates for [$Me_2PC^{\bullet}(sp^2)PMe_2$]PdI (**2'**).

atom	x	y	z
C	−0.031108	−0.016835	0.015152
C	−0.027816	0.001792	1.416416
C	1.193959	0.065679	2.163228
C	2.408273	0.042061	1.396324
C	2.397381	0.044988	−0.005134
C	1.177432	0.027524	−0.711706
P	3.964051	−0.079648	2.424639
C	4.930817	1.514206	2.167430
C	1.259091	0.084204	3.627302
C	0.148662	0.520412	4.478642
C	0.111832	0.125723	5.859262
C	−0.939406	0.500212	6.707124
C	−1.982886	1.321845	6.233961
C	−1.938880	1.779904	4.900127
C	−0.901979	1.391728	4.041030
P	1.610572	−0.837920	6.423076
C	2.133249	−0.090356	8.065134
Pd	2.997657	−0.533349	4.533483
I	5.335609	−1.363243	5.751769
C	1.024366	−2.566868	6.879448
C	5.033808	−1.392731	1.612419
H	−0.971207	−0.080655	1.945104
H	−0.978725	−0.079179	−0.515589
H	1.170507	0.019796	−1.798454
H	3.334278	0.038643	−0.559384
H	−0.872176	1.798719	3.036159
H	−2.711155	2.453270	4.534449
H	−2.792334	1.619656	6.895195
H	−0.945927	0.176032	7.746295
H	5.907428	−1.564354	2.248248
H	4.463046	−2.323047	1.544357
H	5.353530	−1.082419	0.611087
H	3.071358	−0.561668	8.372965
H	2.308773	0.980234	7.927581
H	1.366927	−0.242412	8.833785
H	5.844394	1.471503	2.768654
H	5.187903	1.646393	1.110276

Continued on next page

Table S5. – continued from previous page

atom	x	y	x
H	4.322718	2.358235	2.504424
H	1.887394	−3.155526	7.205925
H	0.278664	−2.524993	7.681516
H	0.585607	−3.039203	5.996204

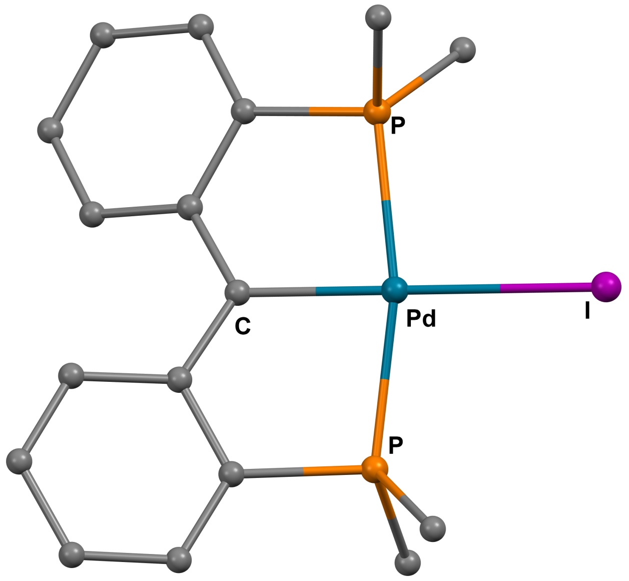

Figure S11. Optimized geometry for [$Me_2PC^{\bullet}(sp^2)PMe_2$]PdI (**2'**).

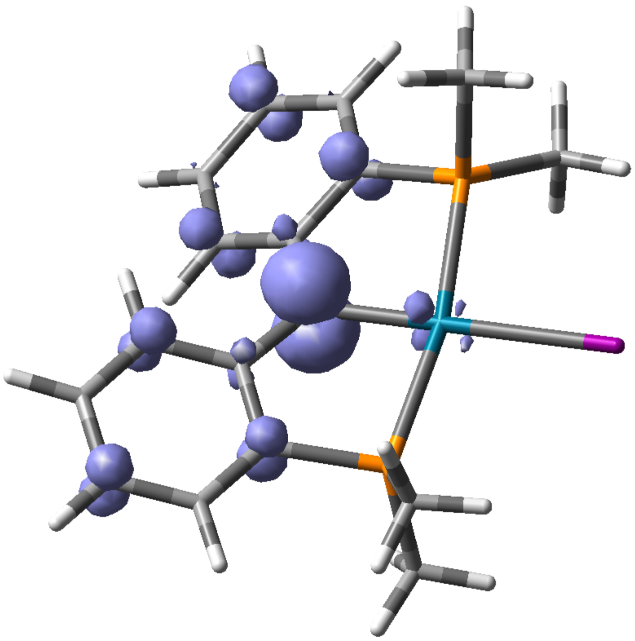

Figure S12. Spin density for [$Me_2PC^{\bullet}(sp^2)PMe_2$]PdI (**2'**).

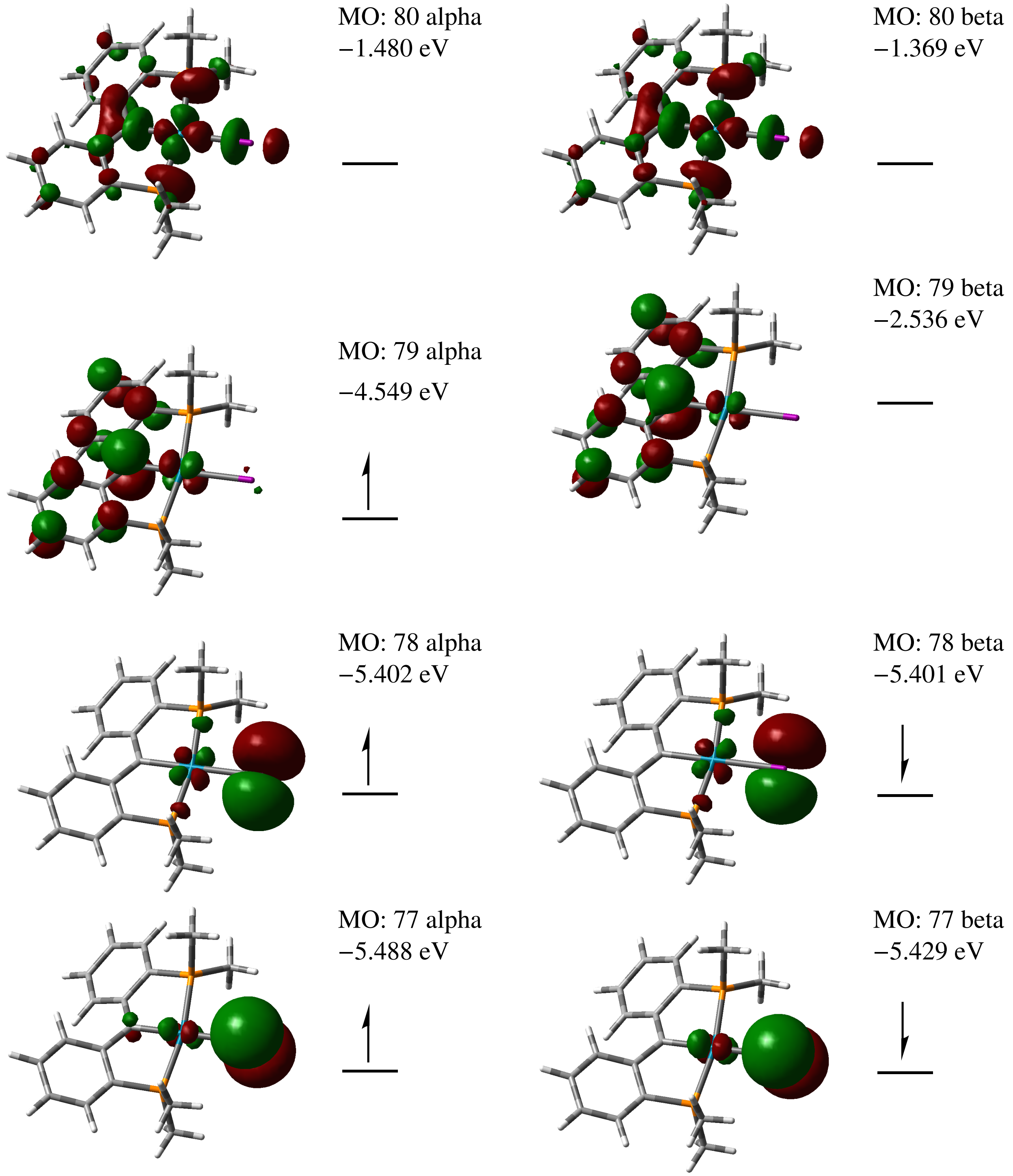

Figure S13. Frontier molecular orbitals for $[Me_2PC^{\bullet}(sp^2)PMe_2]PdI$ (**2’**).

6.6 [PC$^{\bullet}$(sp^2)P]PdBr (3)

Table S6. Optimized coordinates for [PC$^{\bullet}$(sp^2)P]PdBr (**3**).

atom	x	y	z
C	−2.523749	1.541582	−0.271242
C	−1.261254	2.213428	−0.391645
C	−1.289297	3.538012	−0.938071
C	−2.495517	4.166221	−1.275021
C	−3.728523	3.508164	−1.081302
C	−3.727625	2.188064	−0.586119
C	−0.028459	1.510623	−0.027427
C	1.219502	2.204678	0.297147
C	2.474169	1.513746	0.180991
C	3.692135	2.175566	0.401519
C	3.715486	3.521930	0.819041
C	2.492984	4.193933	1.031315
C	1.273045	3.555579	0.773115
P	2.308982	−0.301219	−0.239085
C	3.166509	−0.550215	−1.934379
C	3.227713	−2.049391	−2.303265
P	−2.403755	−0.251356	0.260331
C	−3.619073	−1.152046	−0.924006
C	−3.034300	−1.136957	−2.354268
Pd	−0.046341	−0.529920	−0.023534
Br	0.008132	−3.153402	−0.044946
C	3.296727	−1.332721	1.041652
C	4.832011	−1.167396	0.989924
C	−3.166599	−0.362507	2.026094
C	−2.801067	0.885640	2.859436
C	−2.692951	−1.651406	2.738379
C	−3.943000	−2.590503	−0.465363
C	2.741511	−1.067812	2.458457
C	2.430745	0.272359	−3.014701
H	0.347493	4.085940	0.970445
H	2.494078	5.216912	1.401677
H	4.662124	4.025003	0.998493
H	4.632366	1.649724	0.259227
H	−4.676136	1.668348	−0.465633
H	−4.664233	4.000378	−1.333551
H	−2.476941	5.167419	−1.700342
H	−0.354569	4.051791	−1.135462

Continued on next page

Table S6. – continued from previous page

atom	x	y	x
H	−1.712031	0.981955	2.960103
H	−3.184929	1.810010	2.414391
H	−3.225274	0.787271	3.868010
H	−4.257911	−0.396647	1.884825
H	−3.171338	−1.719665	3.725267
H	−2.928271	−2.560786	2.178205
H	−1.606297	−1.633423	2.881758
H	−4.543252	−0.554541	−0.906133
H	−4.472649	−2.611582	0.494774
H	−4.593519	−3.066087	−1.212320
H	−3.029757	−3.189828	−0.378127
H	−3.760286	−1.574672	−3.053077
H	−2.115610	−1.733829	−2.396726
H	−2.805274	−0.119699	−2.694494
H	3.032055	−2.361022	0.760585
H	2.908479	−0.027596	2.767559
H	3.248453	−1.722809	3.179994
H	1.667473	−1.279133	2.505219
H	5.144275	−0.190756	1.378311
H	5.295756	−1.933752	1.626299
H	5.240204	−1.289560	−0.020852
H	4.187249	−0.154289	−1.822663
H	2.393732	1.339245	−2.763272
H	1.401592	−0.085201	−3.142793
H	2.951517	0.166368	−3.976060
H	3.837270	−2.628528	−1.599084
H	3.671009	−2.162137	−3.302062
H	2.225065	−2.492266	−2.317080

Table S7. Selected distances (Å) and angles (°) for the optimized geometry and the crystal structure of [PC$^{\bullet}$(sp^2)P]PdBr (**3**).

Distance	Calcd.	X-Ray	Angle	Calcd.	X-Ray
Pd – Br	2.624	2.5117(4)	P(1) – Pd – P(2)	167.68	166.96(3)
Pd – P(1)	2.376	2.2891(7)	C – Pd – Br	178.21	175.31(15)
Pd – P(2)	2.391	2.2783(7)	P(2) – Pd – Br	94.30	94.93(2)
Pd – C	2.041	2.020(3)	C(11) – C – C(21)	123.01	123.0(3)
C – C(11)	1.464	1.467(4)	C(11) – C – Pd	118.75	118.1(2)
C – C(21)	1.465	1.463(4)	C(21) – C – Pd	118.22	118.7(2)

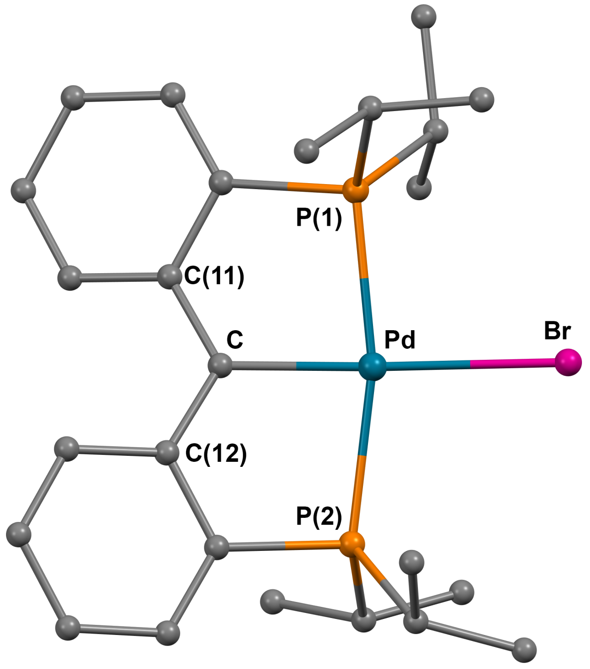

Figure S14. Optimized geometry for [PC$^{\bullet}$(sp^{2})P]PdBr (**3**).

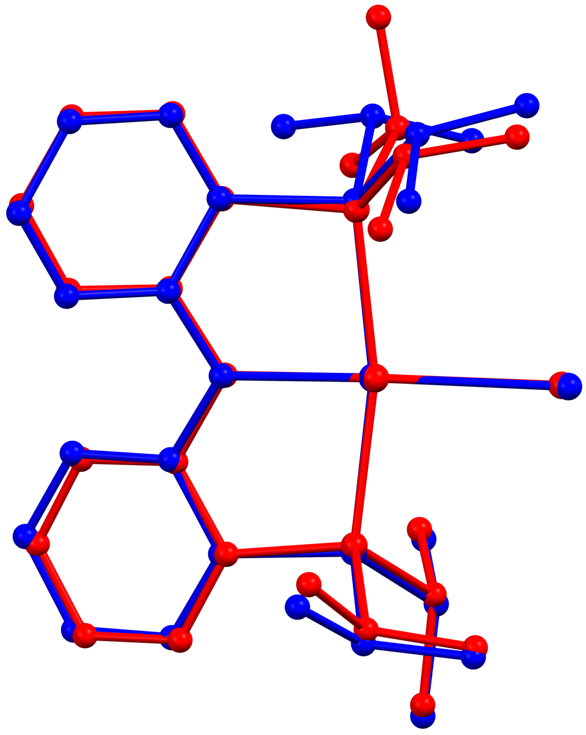

Figure S15. Overlaid structures for [PC$^{\bullet}$(sp^{2})P]PdBr (**3**) (red: X-ray, blue: optimized).

6.7 {[PC(sp^2)P]PdBr}$_2$ ({**3**}$_2$)

Table S8. Optimized coordinates for {[PC(sp^2)P]PdBr}$_2$ ({**3**}$_2$).

atom	x	y	z
C	−3.431432	2.782678	2.270227
C	−3.989782	1.971788	1.239762
C	−5.386126	2.123303	0.984168
C	−6.142484	3.108925	1.643392
C	−5.551616	3.941078	2.612366
C	−4.194673	3.752875	2.938406
C	−3.199677	0.931763	0.522721
Pd	−4.241630	−0.646222	−0.278539
Br	−5.509009	−2.627472	−1.437927
P	−6.123117	0.818805	−0.139754
C	−6.682432	1.675145	−1.763587
C	−7.727230	2.793458	−1.558236
C	−1.825991	1.030398	0.312579
C	−1.029957	−0.147084	−0.100506
C	0.325810	−0.124565	−0.210948
C	1.162947	1.135268	−0.093767
C	0.296949	2.334375	0.247984
C	−1.046725	2.275750	0.431637
C	2.504065	0.967159	0.793735
C	3.190639	2.347233	0.892750
C	3.764978	2.946103	−0.271916
C	4.234630	4.271901	−0.244143
C	4.216617	5.023020	0.946109
C	3.733211	4.418927	2.118968
C	3.222082	3.108209	2.086453
P	4.066886	1.798440	−1.735643
C	3.086635	2.438888	−3.290261
C	2.560581	1.225323	−4.092354
P	−2.106266	−1.655671	−0.420447
C	−1.701657	−2.357531	−2.158858
C	−0.311028	−3.012815	−2.302456
Pd	3.824367	−0.230337	−0.405522
Br	5.216833	−1.882483	−1.913905
P	4.057044	−1.607859	1.490614
C	2.808426	−0.835159	2.645689
C	2.512176	−1.358081	3.923632
C	1.532266	−0.762064	4.733001

Continued on next page

Table S8. – continued from previous page

atom	x	y	x
C	0.846814	0.368614	4.251055
C	1.164935	0.905947	2.993825
C	2.163312	0.332287	2.161914
C	5.882143	2.209222	−2.244929
C	6.863183	1.883748	−1.099904
C	5.727474	−1.476251	2.430409
C	6.858217	−2.220484	1.686543
C	3.732561	−3.489931	1.298626
C	3.920548	−4.339947	2.575175
C	6.080338	0.007490	2.670706
C	2.348009	−3.723923	0.663902
C	6.281736	1.470593	−3.542596
C	1.963812	3.452245	−2.983314
C	−1.614369	−2.991035	0.856627
C	−2.357814	−4.315992	0.573143
C	−7.724386	0.263767	0.757648
C	−7.347854	−0.509266	2.041498
C	−8.624359	−0.585772	−0.167744
C	−5.443327	2.190055	−2.528225
C	−1.892523	−2.473589	2.284678
C	−1.931153	−1.264211	−3.225244
H	5.551648	−1.957334	3.404502
H	5.299346	0.528835	3.236470
H	6.221073	0.535701	1.720475
H	7.016177	0.074118	3.242118
H	7.805280	−2.071936	2.223232
H	6.975343	−1.844862	0.663946
H	6.676787	−3.300128	1.622454
H	4.495381	−3.771065	0.560240
H	2.262135	−3.199916	−0.294130
H	1.545586	−3.385580	1.331890
H	2.203948	−4.796737	0.477008
H	3.111572	−4.170391	3.295796
H	3.894654	−5.403894	2.301806
H	4.878582	−4.154980	3.075392
H	5.889717	3.294636	−2.432297
H	5.643286	1.734749	−4.394758
H	6.240285	0.385333	−3.399446
H	7.312930	1.742776	−3.807405
H	6.601706	2.404671	−0.171295
H	6.877746	0.804599	−0.908802
H	7.877845	2.191559	−1.388561

Continued on next page

Table S8. – continued from previous page

atom	x	y	x
H	3.850469	2.951678	−3.891642
H	1.538997	3.811257	−3.931393
H	2.329211	4.322227	−2.426534
H	1.146442	3.003589	−2.408399
H	2.117287	1.566388	−5.038179
H	1.785787	0.688172	−3.530146
H	3.354847	0.506108	−4.322571
H	0.625101	1.778729	2.645535
H	0.071237	0.835371	4.854201
H	1.303050	−1.174179	5.712568
H	3.044162	−2.230718	4.290999
H	1.555916	1.328607	−1.103740
H	2.841653	2.678897	3.005830
H	3.743532	4.965971	3.059206
H	4.597684	6.040953	0.959916
H	4.656645	4.717119	−1.142792
H	0.795337	3.300919	0.280517
H	0.877617	−1.032051	−0.447840
H	−1.590110	3.200862	0.599579
H	−2.402448	2.621584	2.572657
H	−3.735702	4.349992	3.723430
H	−6.145846	4.692039	3.126750
H	−7.204164	3.213589	1.433879
H	−8.257036	1.184350	1.038674
H	−8.090925	−1.474825	−0.522932
H	−9.510133	−0.915453	0.392658
H	−8.975462	−0.023037	−1.042013
H	−4.721227	1.385110	−2.705890
H	−4.935043	2.987381	−1.969836
H	−5.750551	2.599463	−3.500277
H	−7.300574	3.636384	−1.000643
H	−8.050835	3.174990	−2.536456
H	−8.621471	2.441828	−1.028826
H	−6.824575	−1.439142	1.788988
H	−6.703874	0.081640	2.704409
H	−8.260073	−0.769570	2.595629
H	−0.533276	−3.142567	0.738256
H	−1.568785	−3.225347	3.017800
H	−1.355370	−1.540991	2.494222
H	−2.964846	−2.293344	2.431145
H	−2.103409	−5.050283	1.349875
H	−3.444166	−4.169029	0.578479

Continued on next page

Table S8. – continued from previous page

atom	x	y	x
H	−7.133531	0.856267	−2.340713
H	−2.481365	−3.120613	−2.285912
H	−1.224855	−0.432234	−3.105089
H	−2.951811	−0.870216	−3.169278
H	−1.785432	−1.692672	−4.225989
H	−0.115227	−3.767619	−1.531441
H	0.493658	−2.268686	−2.272971
H	−0.251025	−3.516548	−3.277135
H	−2.085458	−4.747039	−0.397843

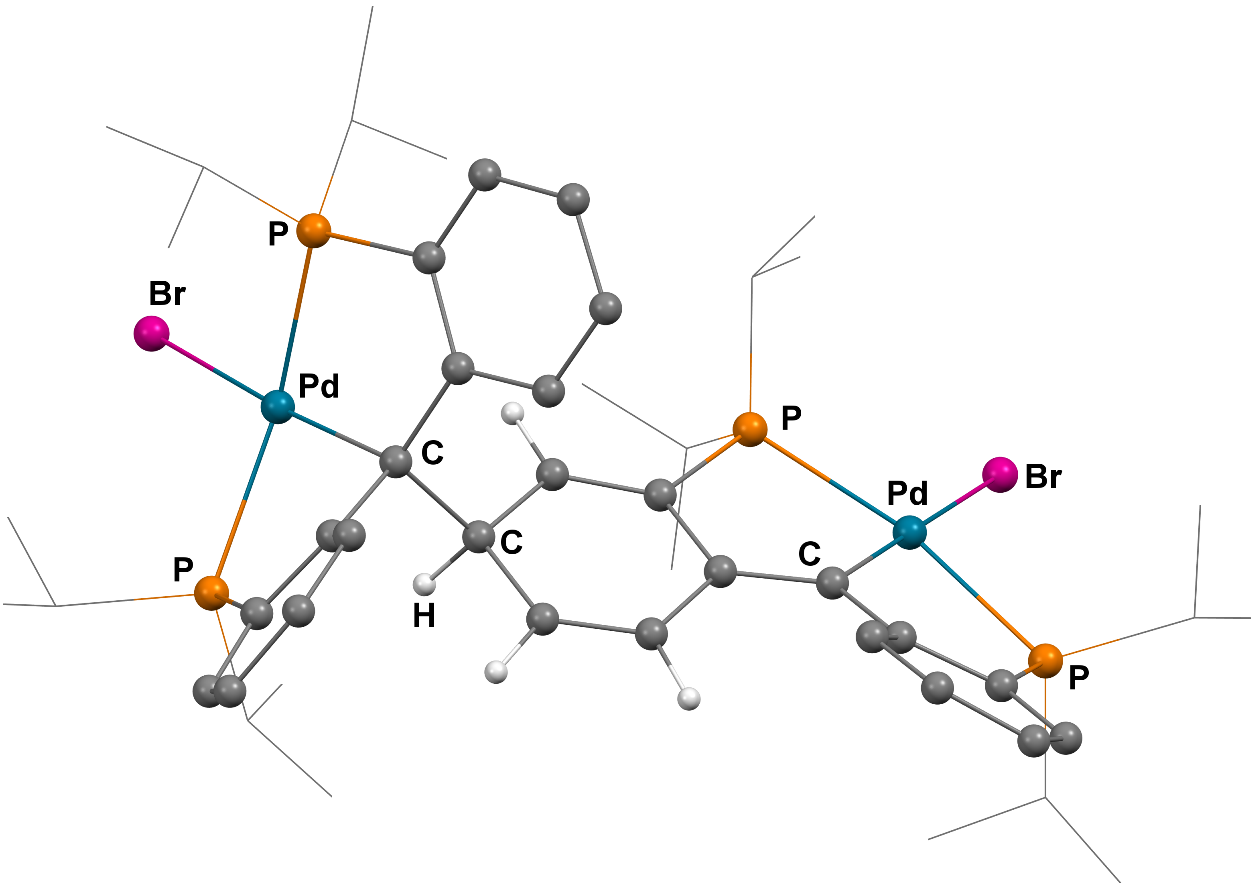

Figure S16. Optimized geometry for {[PC(sp^2)P]PdBr}$_2$ (**{3}$_2$**).

6.8 {*p,p'*-[PC(sp^2)P]PdBr}$_2$ ({*p,p'*-3}$_2$)

Table S9. Optimized coordinates for {*p,p'*-[PC(sp^2)P]PdBr}$_2$ (**{*p,p'*-3}$_2$**).

atom	x	y	z
C	4.425733	−3.350583	0.929137
C	5.237183	−2.318765	0.373617
C	6.597136	−2.646500	0.088081
C	7.062208	−3.969405	0.199609
C	6.219128	−4.987464	0.683536
C	4.907769	−4.659882	1.080595
C	4.730695	−0.937510	0.148535
Pd	6.117148	0.577009	0.044784
Br	7.940470	2.453070	−0.159100
P	7.676352	−1.178357	−0.335792
C	9.172361	−1.154068	0.864840
C	10.176051	−2.317302	0.705189
C	3.390177	−0.656824	−0.109501
C	2.391062	−1.641561	−0.566267
C	1.087391	−1.328006	−0.788411
C	0.487926	0.026057	−0.485373
C	1.573424	1.045915	−0.206589
C	2.883840	0.728317	−0.041791
C	−0.567863	−0.045596	0.737476
C	−1.655681	−1.059923	0.445908
C	−2.961425	−0.733073	0.255992
C	−3.449664	0.661437	0.294283
C	−2.458787	1.632586	0.796616
C	−1.163423	1.307975	1.049225
C	−4.760356	0.969470	−0.064135
Pd	−6.182342	−0.514077	−0.118789
Br	−7.934872	−2.467739	−0.142885
P	−4.314171	−1.966422	−0.161915
C	−4.360977	−3.330771	1.183977
C	−4.644842	−2.688859	2.559907
P	4.226745	1.993521	0.324179
C	3.892003	3.381223	−0.963728
C	4.243755	2.850468	−2.371980
C	−5.213805	2.357319	−0.356475
C	−6.588913	2.708440	−0.219725
C	−7.031084	4.021480	−0.455877
C	−6.133683	5.012617	−0.895884

Continued on next page

Table S9. – continued from previous page

atom	x	y	x
C	−4.788000	4.664069	−1.124338
C	−4.337519	3.360641	−0.863783
P	−7.737730	1.279571	0.167574
C	−8.489608	1.639476	1.904400
C	−9.101160	0.358241	2.518860
C	−3.776406	−2.804835	−1.796481
C	−3.732150	−1.752710	−2.926501
C	−4.716790	−3.978328	−2.152355
C	−9.158878	1.509396	−1.106327
C	−10.393142	0.639309	−0.782952
C	−8.617670	1.211586	−2.522814
C	−3.123656	−4.254896	1.231571
C	−7.420900	2.237125	2.846766
C	3.828665	2.729037	2.058271
C	3.356501	1.625813	3.030845
C	8.376566	−1.514262	−2.086804
C	7.218588	−1.559705	−3.107625
C	5.063548	3.461533	2.635751
C	4.647174	4.691521	−0.654443
C	9.434481	−0.456983	−2.475096
C	8.669137	−1.030552	2.319791
H	−8.080364	4.277813	−0.323712
H	−6.483626	6.023465	−1.089405
H	−4.093588	5.403944	−1.516227
H	−3.307467	3.103790	−1.085677
H	−6.577271	1.545350	2.970118
H	−7.028079	3.191358	2.479271
H	−7.864044	2.406827	3.837506
H	−9.283520	2.384438	1.740393
H	−9.566489	0.605284	3.483335
H	−9.858986	−0.103148	1.879744
H	−8.325676	−0.396394	2.693052
H	−9.442225	2.571159	−1.045160
H	−10.871169	0.932353	0.159466
H	−11.136181	0.761068	−1.583125
H	−10.123305	−0.421494	−0.725974
H	−9.402536	1.409472	−3.265552
H	−8.325342	0.158144	−2.606660
H	−7.749487	1.833719	−2.771778
H	−5.243049	−3.916917	0.892242
H	−3.823696	−2.030290	2.871904
H	−4.756286	−3.476777	3.317047

Continued on next page

Table S9. – continued from previous page

atom	x	y	x
H	−5.571764	−2.105508	2.537611
H	−2.238253	−3.722512	1.599360
H	−3.320820	−5.081140	1.928717
H	−2.885559	−4.698729	0.257024
H	−2.758200	−3.184667	−1.624196
H	−3.060872	−0.919372	−2.686210
H	−4.732018	−1.341636	−3.112692
H	−3.376556	−2.222163	−3.853786
H	−4.678779	−4.783935	−1.409214
H	−4.420742	−4.401612	−3.121860
H	−5.757383	−3.641386	−2.225219
H	−2.812943	2.625588	1.058875
H	−0.511408	2.045854	1.513498
H	−1.334475	−2.095343	0.366332
H	0.030990	−0.393006	1.595454
H	−0.114596	0.363408	−1.344986
H	0.430238	−2.076748	−1.227189
H	1.248299	2.082241	−0.126782
H	2.742808	−2.635187	−0.828399
H	8.085512	−4.212921	−0.072681
H	6.588286	−6.005257	0.780807
H	4.262787	−5.423216	1.510182
H	3.421973	−3.113524	1.265103
H	2.808768	3.568143	−0.910841
H	3.010048	3.448672	1.901641
H	9.664091	−0.209827	0.594574
H	8.847150	−2.507975	−2.040355
H	7.613099	−1.813460	−4.100835
H	6.463854	−2.308869	−2.839270
H	6.723279	−0.583466	−3.176776
H	9.773722	−0.641483	−3.503584
H	9.016179	0.554799	−2.423262
H	10.315371	−0.486127	−1.822189
H	3.978135	3.604222	−3.125855
H	5.319766	2.654585	−2.447539
H	3.703000	1.926340	−2.609590
H	4.460998	5.410622	−1.464191
H	4.311074	5.154042	0.281262
H	5.727523	4.517363	−0.595684
H	11.070714	−2.103546	1.306078
H	9.757116	−3.262409	1.070668
H	10.503169	−2.457212	−0.332294

Continued on next page

Table S9. – continued from previous page

atom	x	y	x
H	9.526915	−0.917211	2.996497
H	8.023767	−0.153280	2.439655
H	8.108467	−1.922540	2.628555
H	4.804383	3.895935	3.611364
H	5.894767	2.761587	2.779647
H	5.428035	4.264662	1.989493
H	3.153047	2.071041	4.014271
H	2.444086	1.128914	2.684210
H	4.133670	0.861774	3.161654

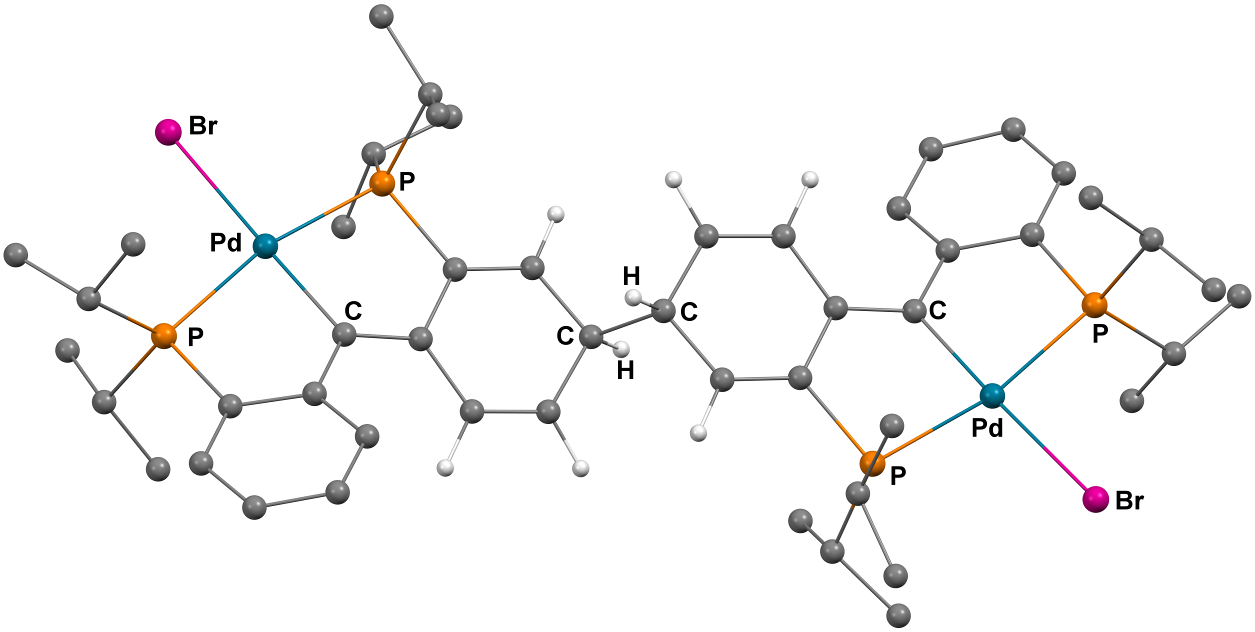

Figure S17. Optimized geometry for {*p,p'*-[PC(sp^2)P]PdBr}$_2$ (**{*p,p'*-3}$_2$**).

6.9 Calculated energies for [PC•(sp^2)P]PdBr (3) and corresponding dimers

		Energy (hartree)	Energy loss (kcal/mol)
[PC•(sp^2)P]PdBr (**3**)	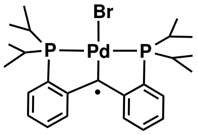	−1127.178084	—
{[PC(sp^2)P]PdBr}$_2$ (**{3}$_2$**)	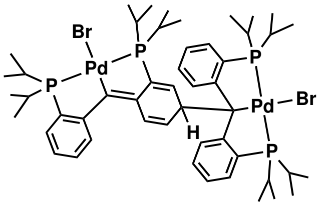	−2254.345893	6.45
{*p,p'*-[PC(sp^2)P]PdBr}$_2$ (**{*p,p'*-3}$_2$**)	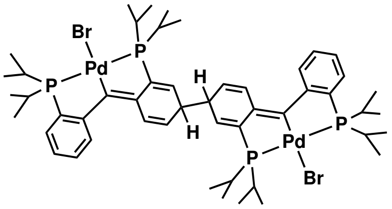	−2254.328477	17.38

6.10 [$Me_2PC^\bullet(sp^2)PMe_2$]PdBr (3’)

Table S10. Optimized coordinates for [$Me_2PC^\bullet(sp^2)PMe_2$]PdBr (**3’**).

atom	x	y	z
C	0.001362	0.001603	−0.006068
C	0.000391	0.002264	1.427140
C	1.289194	0.001383	2.062617
C	2.477383	0.081278	1.323694
C	2.443074	0.123262	−0.085181
C	1.194343	0.060512	−0.739119
C	−1.214015	−0.064724	2.245777
Pd	−1.052732	−0.760324	4.164700
Br	−0.847494	−1.645464	6.605170
P	1.227598	−0.198378	3.919299
C	1.890658	1.391457	4.676455
C	−2.537804	0.340116	1.762952
C	−2.750335	1.253315	0.678985
C	−4.039896	1.611019	0.262912
C	−5.179939	1.079482	0.902188
C	−5.003560	0.212707	2.000122
C	−3.716739	−0.132846	2.434713
P	−3.371079	−1.158327	3.957828
C	−4.507945	−0.522265	5.310329
C	−3.999245	−2.898788	3.616191
C	2.516010	−1.488072	4.369837
H	−1.895723	1.716005	0.197422
H	−4.159793	2.317846	−0.555374
H	−6.178305	1.354497	0.572368
H	−5.878205	−0.170325	2.522988
H	−0.938508	−0.093345	−0.538960
H	1.154104	0.044411	−1.826177
H	3.365633	0.175246	−0.657310
H	3.438874	0.089738	1.834154
H	−4.238382	−1.024743	6.244133
H	−4.346209	0.552303	5.433201
H	−5.560578	−0.711864	5.071137
H	2.414546	−1.710734	5.436263
H	2.316924	−2.401454	3.802342
H	3.530413	−1.133562	4.154183
H	−3.792799	−3.524399	4.490248
H	−5.075916	−2.891020	3.411535

Continued on next page

Table S10. – continued from previous page

**atom**	**x**	**y**	**x**
H	−3.465821	−3.306945	2.753251
H	1.849303	1.304926	5.766771
H	2.923334	1.573472	4.357684
H	1.258275	2.226385	4.362150

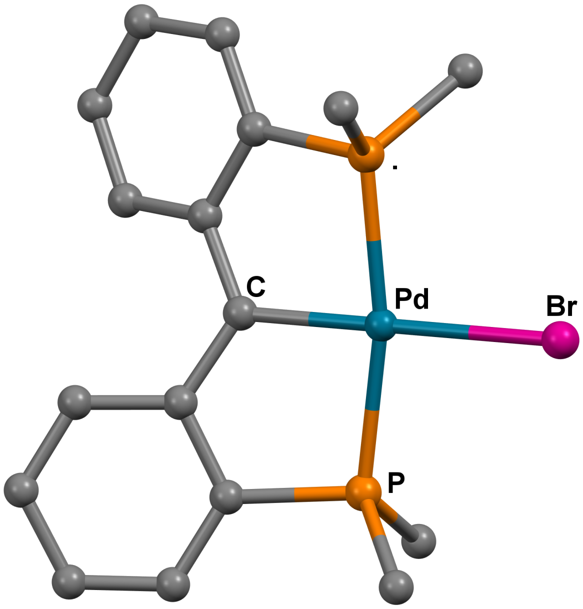

Figure S18. Optimized geometry for $[Me_2PC^{\bullet}(sp^2)PMe_2]PdBr$ (**3’**).

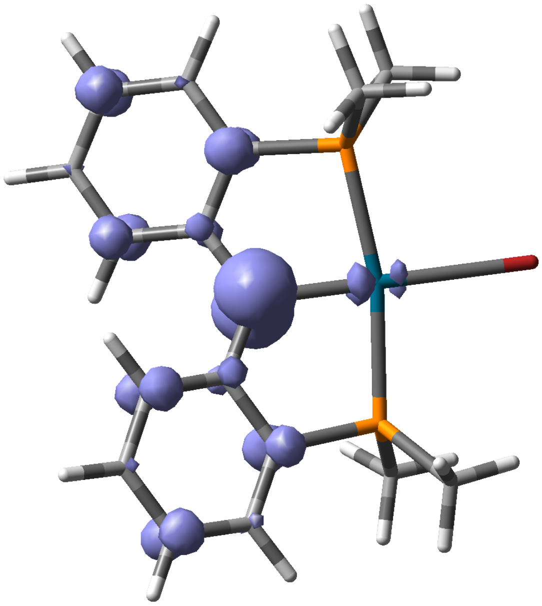

Figure S19. Spin density for $[Me_2PC^{\bullet}(sp^2)PMe_2]PdBr$ (**3’**).

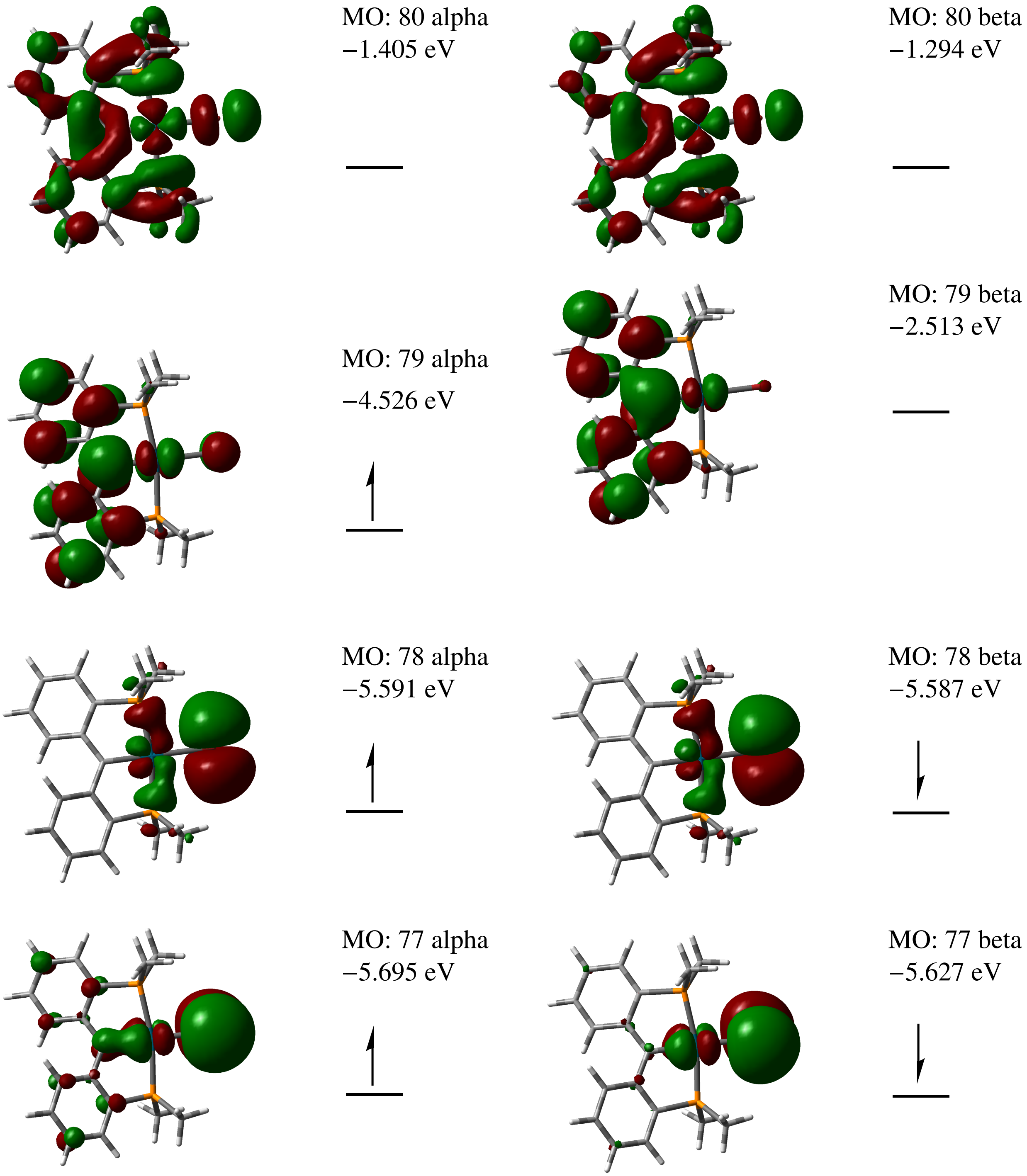

Figure S20. Frontier molecular orbitals for $[Me_2PC^{\bullet}(sp^2)PMe_2]PdBr$ (**3’**).

6.11 [PC•(sp²)P]PdCl (5)

Table S11. Optimized coordinates for [PC•(sp²)P]PdCl (5).

atom	x	y	z
C	−2.533971	1.317523	−0.266066
C	−1.276613	2.002126	−0.381027
C	−1.317489	3.328138	−0.922988
C	−2.529122	3.943827	−1.264003
C	−3.756008	3.271553	−1.080027
C	−3.743198	1.950522	−0.587457
C	−0.036888	1.307492	−0.021397
C	1.210155	2.006726	0.299085
C	2.468125	1.319926	0.180852
C	3.684039	1.984012	0.405396
C	3.703519	3.329690	0.825414
C	2.478912	3.998264	1.036228
C	1.260641	3.357128	0.776359
P	2.306607	−0.493531	−0.243779
C	3.153001	−0.758939	−1.940420
C	3.168282	−2.261472	−2.303566
P	−2.395244	−0.473786	0.261194
C	−3.570697	−1.414321	−0.928161
C	−3.012741	−1.331691	−2.366903
Pd	−0.041496	−0.726136	−0.025537
Cl	0.029648	−3.206109	−0.070665
C	3.278905	−1.540088	1.034736
C	4.815410	−1.388433	0.987768
C	−3.165882	−0.623611	2.020721
C	−2.907256	0.652684	2.850766
C	−2.600510	−1.869723	2.743184
C	−3.775911	−2.882811	−0.492563
C	2.719872	−1.278391	2.450610
C	2.432125	0.080275	−3.017954
H	0.333850	3.884866	0.974920
H	2.476975	5.020841	1.407749
H	4.648527	3.834812	1.007704
H	4.625508	1.459658	0.264579
H	−4.685744	1.418015	−0.474936
H	−4.695424	3.753915	−1.337503
H	−2.519346	4.946571	−1.686032
H	−0.387943	3.852978	−1.115633

Continued on next page

Table S11. – continued from previous page

atom	x	y	x
H	−1.830201	0.836558	2.958500
H	−3.360276	1.542000	2.399371
H	−3.328785	0.524418	3.857174
H	−4.249553	−0.744180	1.869213
H	−3.103586	−1.989938	3.712713
H	−2.729951	−2.791851	2.169417
H	−1.525746	−1.751187	2.924667
H	−4.534487	−0.884190	−0.882183
H	−4.283707	−2.962136	0.476519
H	−4.402154	−3.393820	−1.236816
H	−2.815521	−3.408143	−0.432165
H	−3.718952	−1.805774	−3.062262
H	−2.056689	−1.863940	−2.435628
H	−2.859468	−0.295266	−2.691025
H	3.003600	−2.563860	0.747224
H	2.892293	−0.241101	2.766443
H	3.218853	−1.941066	3.170762
H	1.644255	−1.482737	2.490256
H	5.134809	−0.411553	1.370286
H	5.271462	−2.154424	1.630145
H	5.224961	−1.520403	−0.021317
H	4.185007	−0.391183	−1.836821
H	2.427221	1.148688	−2.770274
H	1.392196	−0.248506	−3.136198
H	2.941822	−0.043757	−3.983062
H	3.774768	−2.852566	−1.606568
H	3.591498	−2.390384	−3.309010
H	2.154095	−2.677911	−2.296934

Table S12. Selected distances (Å) and angles (°) for the optimized geometry and the crystal structure of [PC$^{\bullet}$(sp^2)P]PdCl (**5**).

Distance	Calcd.	X-Ray	Angle	Calcd.	X-Ray
Pd – Cl	2.481	2.3884(6)	P(1) – Pd – P(2)	168.18	165.30(2)
Pd – P(1)	2.370	2.2751(6)	C – Pd – Cl	178.00	175.61(8)
Pd – P(2)	2.385	2.2977(6)	P(1) – Pd – Cl	93.90	94.32(2)
Pd – C	2.034	2.005(2)	C(11) – C – C(21)	123.18	122.2(2)
C – C(11)	1.465	1.461(3)	C(11) – C – Pd	118.66	118.78(17)
C – C(21)	1.466	1.446(3)	C(21) – C – Pd	118.13	118.87(17)

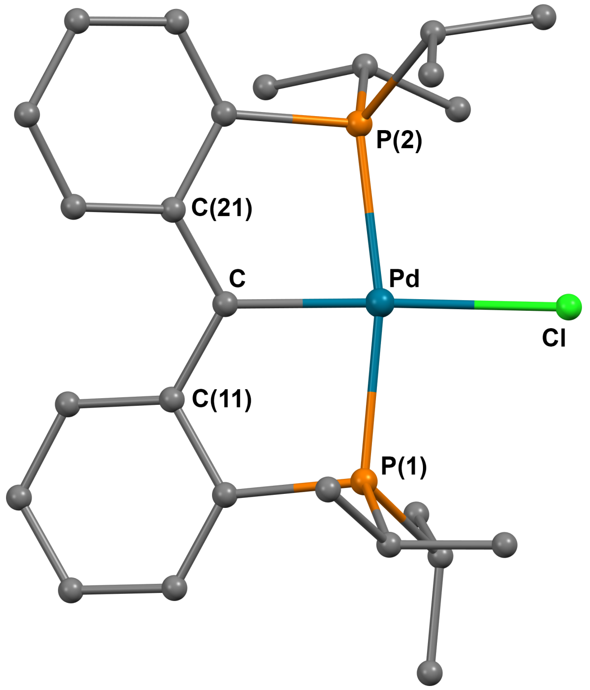

Figure S21. Optimized geometry for [PC•(sp^2)P]PdCl (**5**).

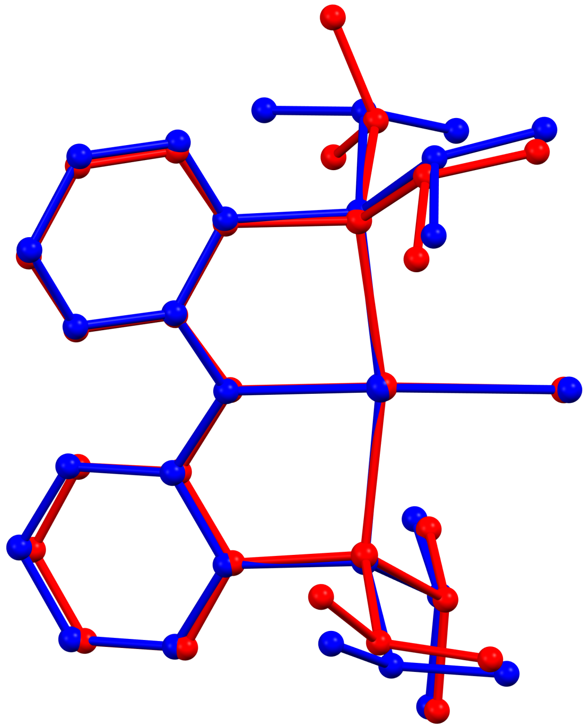

Figure S22. Overlaid structures for [PC•(sp^2)P]PdCl (**5**) (red: Xray, blue: optimized).

6.12 {[PC(sp^2)P]PdCl}$_2$ ({**5**}$_2$)

Table S13. Optimized coordinates for {[PC(sp^2)P]PdCl}$_2$ ({**5**}$_2$).

atom	x	y	z
C	3.526384	−2.022297	2.738419
C	4.048486	−1.467370	1.534512
C	5.440754	−1.663742	1.282779
C	6.228282	−2.459321	2.133642
C	5.673382	−3.045702	3.286724
C	4.321119	−2.801223	3.594380
C	3.230291	−0.622338	0.618639
Pd	4.237998	0.752416	−0.512651
Cl	5.388394	2.411704	−1.957049
P	6.125119	−0.661553	−0.142840
C	6.607234	−1.880863	−1.544084
C	7.679294	−2.914385	−1.135847
C	1.854280	−0.773441	0.460540
C	1.042092	0.274098	−0.200373
C	−0.310985	0.201241	−0.319007
C	−1.129534	−1.015821	0.068358
C	−0.253746	−2.080791	0.704555
C	1.088066	−1.962217	0.874243
C	−2.498545	−0.669427	0.852853
C	−3.174567	−1.999505	1.253282
C	−3.709082	−2.862566	0.245482
C	−4.179428	−4.147628	0.571670
C	−4.196995	−4.596800	1.905497
C	−3.749580	−3.729627	2.916421
C	−3.240244	−2.458763	2.591016
P	−3.966925	−2.095991	−1.455342
C	−2.919354	−3.065416	−2.778788
C	−2.333555	−2.061025	−3.799419
P	2.099583	1.701476	−0.818418
C	1.709868	2.054235	−2.660854
C	0.316013	2.654446	−2.943108
Pd	−3.803432	0.182788	−0.612205
Cl	−5.203693	1.318773	−2.337194
P	−4.112151	1.959200	0.893008
C	−2.880106	1.511530	2.222896
C	−2.624690	2.327152	3.347488
C	−1.660261	1.956440	4.297855

Continued on next page

Table S13. – continued from previous page

atom	x	y	x
C	−0.949298	0.755824	4.113584
C	−1.225569	−0.068249	3.011289
C	−2.206796	0.274788	2.043315
C	−5.761667	−2.617317	−1.923291
C	−6.779441	−2.053202	−0.910180
C	−5.808005	2.038827	1.792515
C	−6.916651	2.583001	0.863607
C	−3.810095	3.732596	0.228969
C	−3.954264	4.871417	1.262510
C	−6.173886	0.649542	2.357415
C	−2.455782	3.797627	−0.503258
C	−6.105815	−2.164213	−3.361486
C	−1.826506	−3.985666	−2.194161
C	1.624294	3.263899	0.176797
C	2.398878	4.491414	−0.356022
C	7.760638	0.096037	0.503131
C	7.439633	1.142687	1.593851
C	8.565889	0.724279	−0.657925
C	5.336122	−2.567510	−2.090035
C	1.893087	3.025361	1.678395
C	1.967275	0.782231	−3.498135
H	−5.661815	2.732892	2.634438
H	−5.394843	0.253182	3.019166
H	−6.330942	−0.069730	1.545692
H	−7.105658	0.722531	2.934683
H	−7.878159	2.558289	1.394631
H	−7.005234	1.977357	−0.045423
H	−6.732928	3.619402	0.555598
H	−4.603711	3.820129	−0.524978
H	−2.412295	3.057027	−1.308642
H	−1.621495	3.624324	0.188953
H	−2.324380	4.792707	−0.949932
H	−3.112832	4.883489	1.965550
H	−3.953170	5.835863	0.736149
H	−4.887822	4.812450	1.835108
H	−5.772227	−3.717868	−1.875686
H	−5.430492	−2.590715	−4.114000
H	−6.070520	−1.071818	−3.438616
H	−7.124230	−2.494731	−3.608737
H	−6.566758	−2.379643	0.114723
H	−6.776009	−0.957708	−0.942440
H	−7.788385	−2.398990	−1.175007

Continued on next page

Table S13. – continued from previous page

atom	x	y	x
H	−3.657484	−3.697245	−3.292118
H	−1.367022	−4.559037	−3.011665
H	−2.228032	−4.698517	−1.465415
H	−1.028107	−3.417128	−1.704410
H	−1.855354	−2.603719	−4.626605
H	−1.573264	−1.423972	−3.329503
H	−3.102716	−1.401551	−4.217588
H	−0.665504	−0.988724	2.892819
H	−0.186267	0.457693	4.829168
H	−1.462959	2.591838	5.157665
H	−3.176730	3.252483	3.485070
H	−1.487899	−1.451266	−0.877450
H	−2.889914	−1.821002	3.394126
H	−3.787774	−4.040070	3.958362
H	−4.577737	−5.585678	2.147963
H	−4.574393	−4.794705	−0.209046
H	−0.741126	−3.016037	0.972686
H	−0.874149	1.022972	−0.756464
H	1.639775	−2.812150	1.264664
H	2.502081	−1.805661	3.021860
H	3.890279	−3.203094	4.508938
H	6.291207	−3.648231	3.947756
H	7.286083	−2.599992	1.924681
H	8.339347	−0.724512	0.952407
H	7.967396	1.480290	−1.179787
H	9.463646	1.212284	−0.254297
H	8.895554	−0.023531	−1.390491
H	4.587235	−1.830310	−2.400886
H	4.879553	−3.217130	−1.331725
H	5.593385	−3.188948	−2.958548
H	7.299763	−3.592632	−0.361309
H	7.947442	−3.527228	−2.007730
H	8.599851	−2.444574	−0.767720
H	6.863992	1.973135	1.167891
H	6.865693	0.711313	2.423311
H	8.375621	1.548091	2.002124
H	0.546072	3.414084	0.028189
H	1.597527	3.915468	2.250747
H	1.326721	2.169408	2.063834
H	2.959949	2.841811	1.857079
H	2.170553	5.365885	0.268608
H	3.481040	4.317860	−0.328539

Continued on next page

Table S13. – continued from previous page

atom	x	y	x
H	7.012462	−1.223957	−2.325876
H	2.485135	2.788327	−2.918753
H	1.268900	−0.022192	−3.231827
H	2.991898	0.421191	−3.356404
H	1.831498	1.011825	−4.563670
H	0.103928	3.538145	−2.329926
H	−0.482718	1.920715	−2.781657
H	0.264887	2.964095	−3.996115
H	2.128783	4.737466	−1.390168

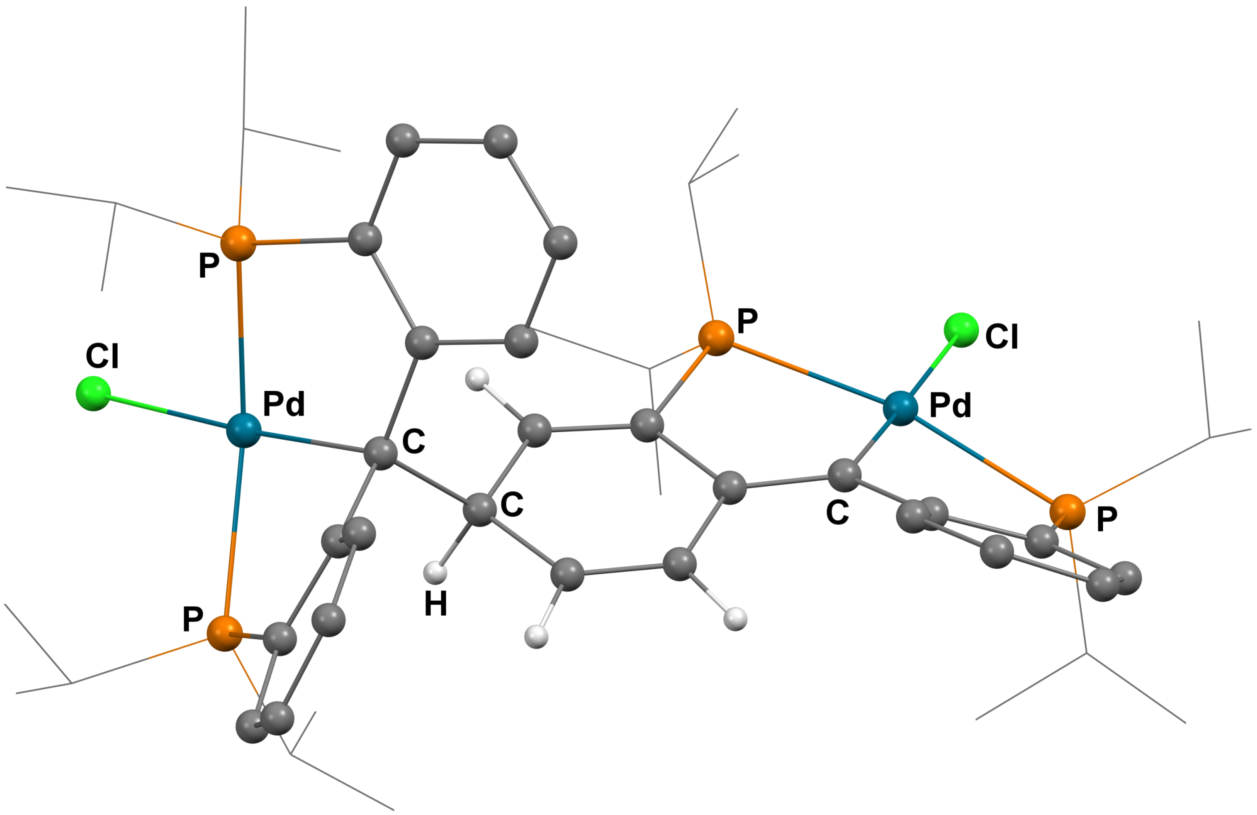

Figure S23. Optimized geometry for {[PC(sp^2)P]PdCl}$_2$ (**{5}$_2$**).

6.13 {*p,p'*-[PC(sp^2)P]PdCl}$_2$ ({*p,p'*-**5**}$_2$)

Table S14. Optimized coordinates for {*p,p'*-[PC(sp^2)P]PdCl}$_2$ (**{*p,p'*-5}$_2$**).

atom	x	y	z
C	4.593542	−3.184905	0.941779
C	5.348943	−2.115349	0.378777
C	6.723379	−2.375377	0.089140
C	7.256072	−3.671954	0.206790
C	6.467653	−4.729372	0.698426
C	5.142421	−4.467221	1.097674
C	4.772702	−0.760629	0.152686
Pd	6.079267	0.814059	0.026890
Cl	7.703751	2.670767	−0.237068
P	7.722757	−0.855245	−0.342850
C	9.211229	−0.731674	0.859994
C	10.280781	−1.836087	0.711359
C	3.417777	−0.546072	−0.097480
C	2.463013	−1.578487	−0.543022
C	1.145995	−1.326281	−0.765763
C	0.485753	0.002634	−0.477138
C	1.523860	1.072122	−0.203963
C	2.847018	0.815071	−0.036555
C	−0.571523	−0.105081	0.741135
C	−1.625337	−1.152495	0.439930
C	−2.939099	−0.864927	0.241211
C	−3.472474	0.513973	0.284738
C	−2.514813	1.513537	0.794633
C	−1.211375	1.228172	1.053268
C	−4.792925	0.781079	−0.072687
Pd	−6.157744	−0.744275	−0.149400
Cl	−7.748969	−2.648272	−0.201044
P	−4.250397	−2.134928	−0.196093
C	−4.291665	−3.507499	1.139612
C	−4.617026	−2.882095	2.513953
P	4.132399	2.140650	0.314835
C	3.753727	3.509636	−0.977210
C	4.086429	2.971225	−2.387355
C	−5.296063	2.155375	−0.353747
C	−6.683705	2.455234	−0.213900
C	−7.176725	3.749560	−0.451193
C	−6.317843	4.774326	−0.891451

Continued on next page

Table S14. – continued from previous page

atom	x	y	x
C	−4.959179	4.478110	−1.116913
C	−4.458559	3.192663	−0.857208
P	−7.771008	0.981562	0.175682
C	−8.512107	1.264094	1.931768
C	−8.954198	−0.081898	2.554399
C	−3.690976	−2.957521	−1.829699
C	−3.631648	−1.897366	−2.951369
C	−4.637135	−4.122389	−2.201382
C	−9.229240	1.140629	−1.061417
C	−10.375260	0.158090	−0.730456
C	−8.701945	0.920324	−2.497080
C	−3.035214	−4.403935	1.203035
C	−7.497393	1.980078	2.850554
C	3.733639	2.876312	2.047661
C	3.277868	1.773113	3.027812
C	8.445779	−1.139523	−2.091900
C	7.293639	−1.254806	−3.113876
C	4.969827	3.618925	2.610505
C	4.511512	4.824047	−0.688682
C	9.429206	−0.006911	−2.466611
C	8.696631	−0.626047	2.312481
H	−8.235701	3.963324	−0.320211
H	−6.706140	5.770776	−1.086544
H	−4.293316	5.244681	−1.507091
H	−3.419234	2.976100	−1.079058
H	−6.575806	1.391269	2.949272
H	−7.227178	2.974385	2.478770
H	−7.932047	2.094189	3.852994
H	−9.391814	1.910681	1.790346
H	−9.437886	0.107639	3.522788
H	−9.651519	−0.638486	1.922124
H	−8.086642	−0.730359	2.722421
H	−9.598317	2.173396	−0.964626
H	−10.844529	0.377477	0.236396
H	−11.152644	0.241146	−1.502376
H	−10.011861	−0.876244	−0.717942
H	−9.518025	1.072704	−3.216487
H	−8.331199	−0.105056	−2.613210
H	−7.890930	1.613684	−2.750242
H	−5.156320	−4.108594	0.827109
H	−3.822232	−2.200152	2.843504
H	−4.716820	−3.677508	3.264947

Continued on next page

Table S14. – continued from previous page

atom	x	y	x
H	−5.561323	−2.328155	2.477846
H	−2.166165	−3.850801	1.579625
H	−3.221120	−5.233978	1.898759
H	−2.776324	−4.842862	0.231432
H	−2.676522	−3.344609	−1.651696
H	−2.960631	−1.067766	−2.697733
H	−4.628311	−1.482324	−3.145969
H	−3.267130	−2.361235	−3.877985
H	−4.601078	−4.938602	−1.469686
H	−4.344551	−4.533269	−3.177156
H	−5.676534	−3.779618	−2.266511
H	−2.900287	2.494578	1.057833
H	−0.584715	1.984659	1.522568
H	−1.272837	−2.177770	0.357397
H	0.034462	−0.435156	1.600874
H	−0.127217	0.305763	−1.342201
H	0.522871	−2.108908	−1.195168
H	1.153914	2.093866	−0.132469
H	2.858198	−2.557782	−0.796938
H	8.290199	−3.863939	−0.066145
H	6.888717	−5.726407	0.799908
H	4.538562	−5.260345	1.533053
H	3.580308	−2.998120	1.280964
H	2.670330	3.691024	−0.906649
H	2.909300	3.590261	1.895911
H	9.647713	0.237210	0.582003
H	8.983362	−2.098921	−2.053064
H	7.704136	−1.469947	−4.109683
H	6.592822	−2.057481	−2.853513
H	6.731153	−0.314877	−3.171996
H	9.774392	−0.150866	−3.499514
H	8.945858	0.974340	−2.393807
H	10.313793	0.009422	−1.817920
H	3.799841	3.716268	−3.142239
H	5.163214	2.786760	−2.479655
H	3.551901	2.039443	−2.608485
H	4.314926	5.536524	−1.501761
H	4.186439	5.294042	0.247340
H	5.592470	4.648589	−0.641186
H	11.153707	−1.576052	1.325957
H	9.909710	−2.804992	1.066453
H	10.629747	−1.952386	−0.322072

Continued on next page

Table S14. – continued from previous page

atom	x	y	x
H	9.543138	−0.452852	2.990722
H	7.995950	0.209282	2.421667
H	8.191766	−1.548496	2.628050
H	4.719149	4.051828	3.588920
H	5.807693	2.924678	2.743977
H	5.320087	4.423972	1.958792
H	3.078170	2.220119	4.011216
H	2.367068	1.267240	2.689846
H	4.062394	1.015931	3.155421

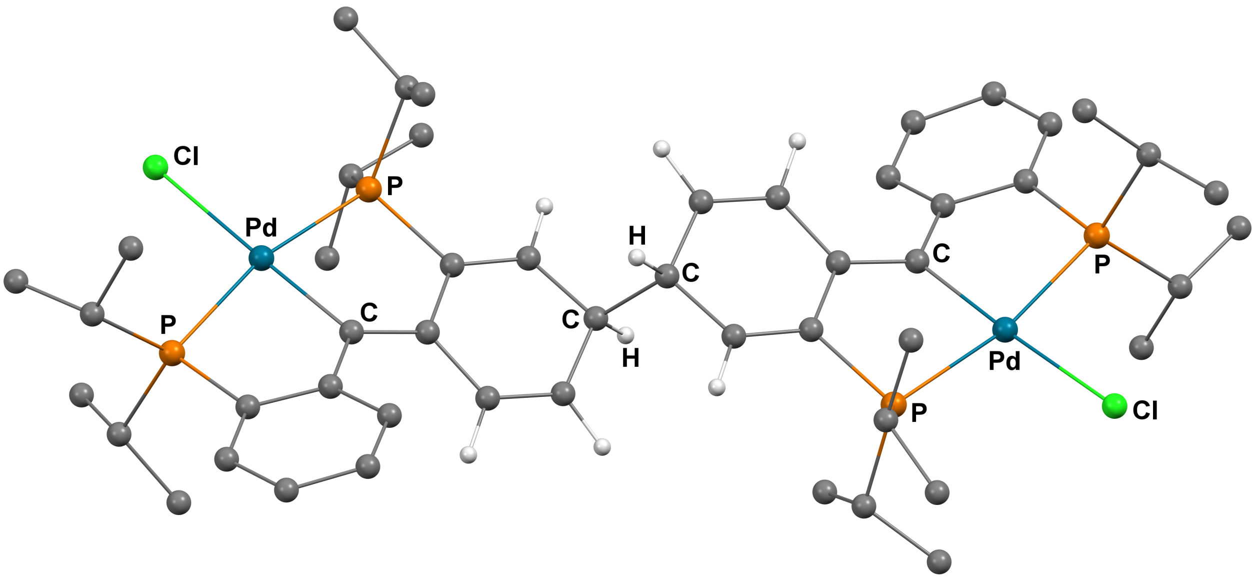

Figure S24. Optimized geometry for {*p,p’*-[PC(sp^2)P]PdCl}$_2$ (**{*p,p’*-5}$_2$**).

6.14 Calculated energies for $[PC^{\bullet}(sp^2)P]PdCl$ (5) and corresponding dimers

		Energy (hartree)	Energy loss (kcal/mol)
$[PC^{\bullet}(sp^2)P]PdCl$ (**5**)	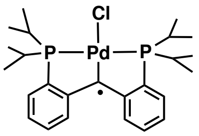	−1128.9587254	—
$\{[PC(sp^2)P]PdCl\}_2$ (**$\{5\}_2$**)	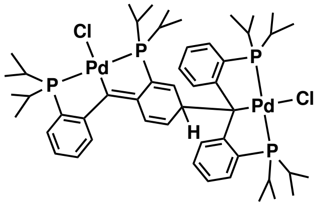	−2257.904652	8.03
$\{p,p'\text{-}[PC(sp^2)P]PdCl\}_2$ (**$\{p,p'\text{-}5\}_2$**)	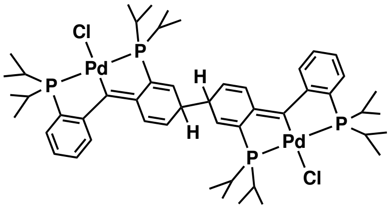	−2257.886631	19.34

6.15 [$Me_2PC^{\bullet}(sp^2)PMe_2$]PdCl (5')

Table S15. Optimized coordinates for [$Me_2PC^{\bullet}(sp^2)PMe_2$]PdCl (**5'**).

atom	x	y	z
C	0.001736	0.003468	−0.011651
C	0.000206	0.005071	1.421448
C	1.289249	0.004683	2.057319
C	2.477685	0.082664	1.318574
C	2.443738	0.122861	−0.090350
C	1.194994	0.060804	−0.744413
C	−1.214469	−0.062727	2.240369
Pd	−1.053654	−0.756342	4.153711
Cl	−0.859618	−1.593209	6.462299
P	1.224649	−0.189176	3.913897
C	1.893347	1.399639	4.668084
C	−2.538615	0.341633	1.756846
C	−2.751642	1.255451	0.673641
C	−4.041355	1.614138	0.258820
C	−5.181349	1.083023	0.898619
C	−5.004691	0.214852	1.995389
C	−3.717751	−0.132022	2.428599
P	−3.369447	−1.161820	3.947428
C	−4.490153	−0.528575	5.314183
C	−4.003516	−2.899923	3.605023
C	2.499338	−1.486668	4.379390
H	−1.897157	1.717991	0.191652
H	−4.161473	2.321532	−0.558965
H	−6.179749	1.359122	0.569779
H	−5.879066	−0.168686	2.518417
H	−0.938071	−0.091065	−0.544782
H	1.155008	0.043962	−1.831485
H	3.366414	0.173367	−0.662440
H	3.438966	0.091366	1.829510
H	−4.205663	−1.031733	6.243126
H	−4.327330	0.545975	5.436211
H	−5.546021	−0.718871	5.090529
H	2.382478	−1.706487	5.444782
H	2.299014	−2.399565	3.811456
H	3.519268	−1.141374	4.175421
H	−3.793144	−3.528521	4.476069
H	−5.081423	−2.890000	3.406980

Continued on next page

Table S15. – continued from previous page

atom	x	y	x
H	−3.476114	−3.306826	2.737758
H	1.847824	1.317132	5.758604
H	2.928113	1.575807	4.352764
H	1.266238	2.236697	4.348802

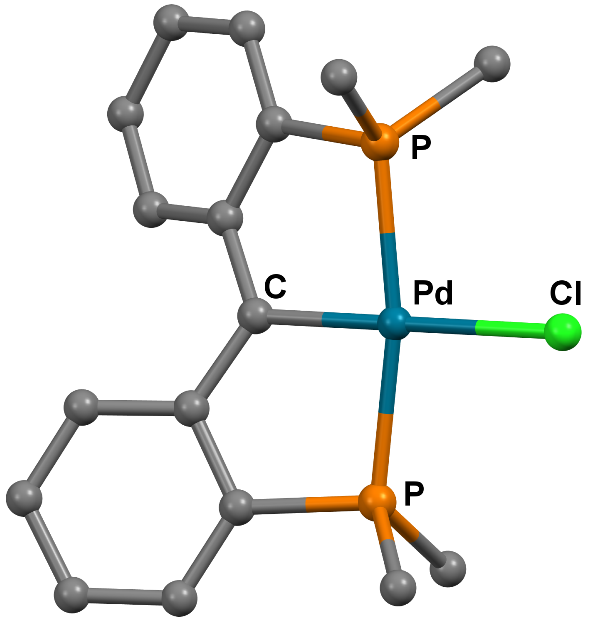

Figure S25. Optimized geometry for [$Me_2PC^{\bullet}(sp^2)PMe_2$]PdCl (**5'**).

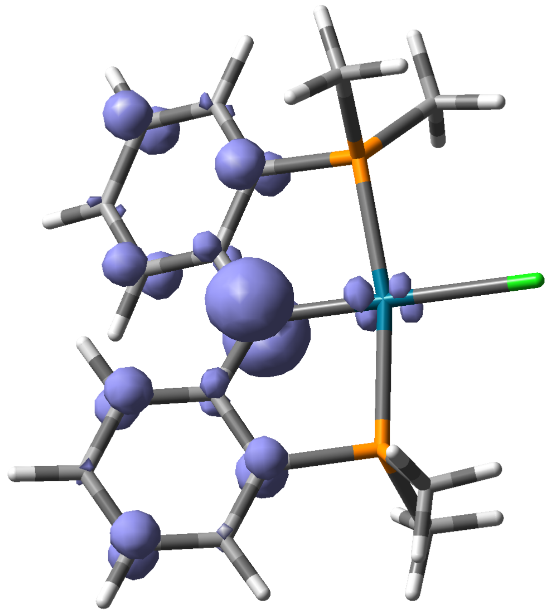

Figure S26. Spin density for [$Me_2PC^{\bullet}(sp^2)PMe_2$]PdCl (**5'**).

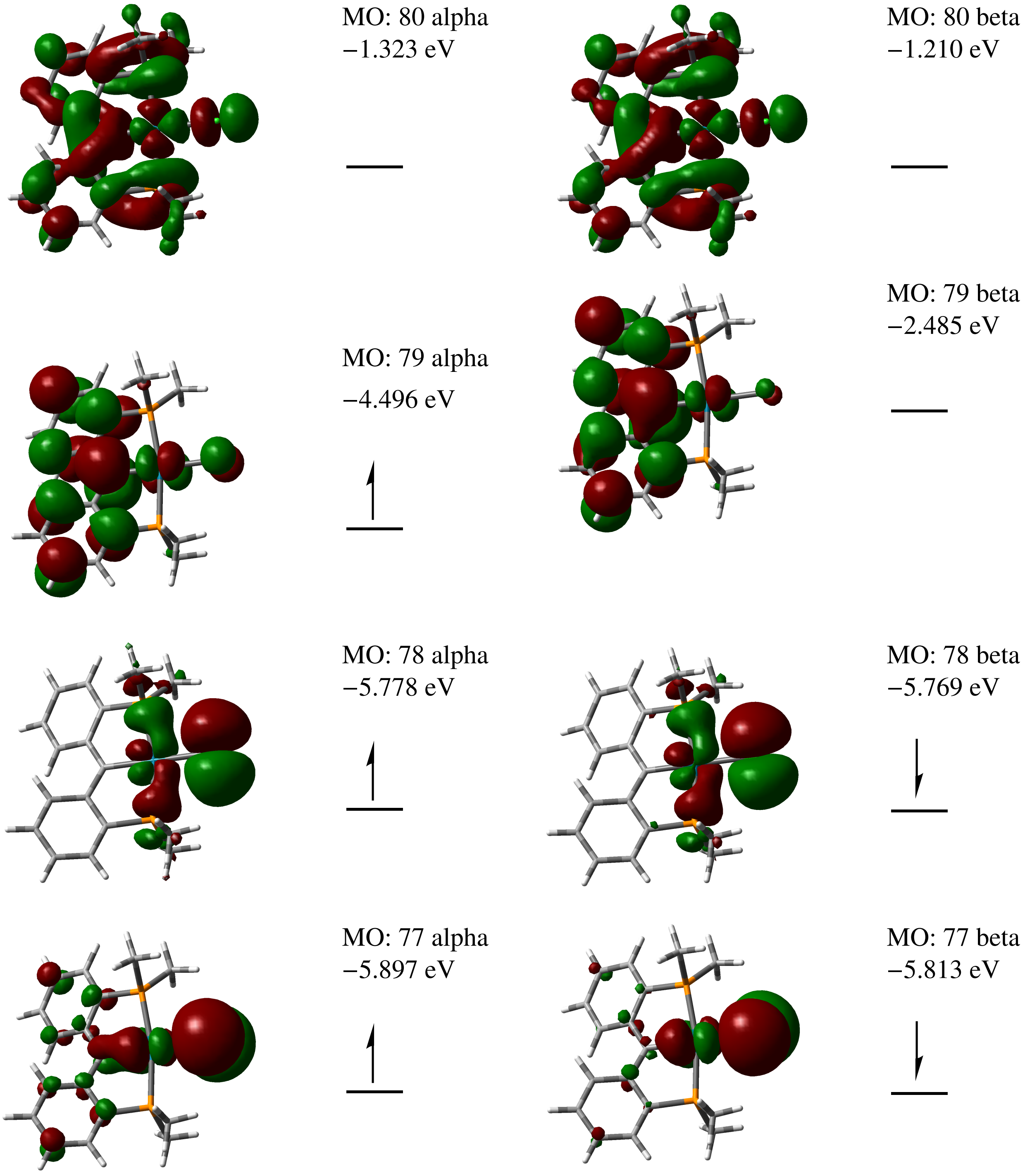

Figure S27. Frontier molecular orbitals for $[Me_2PC^{\bullet}(sp^2)PMe_2]PdCl$ (**5’**).

7 NMR Spectra

7.1 NMR Spectra for [PC•(sp^2)P]PdI (2)and {[PC(sp^2)P]PdI}$_2$ ({2}$_2$)

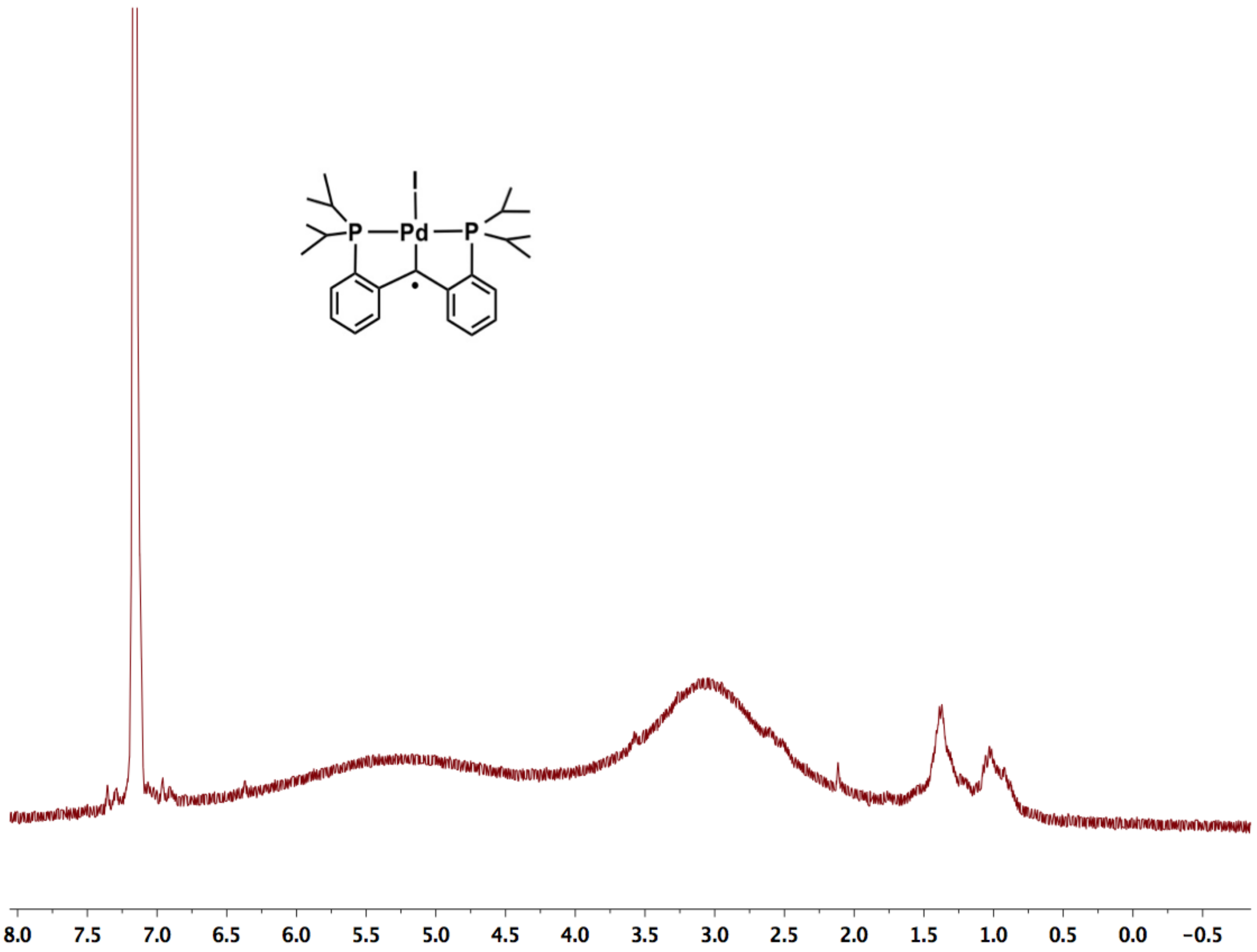

Figure S28. ^{1}H NMR spectrum (293 K) for [PC•(sp^2)P]PdI (**2**).

7.2 NMR Spectra for [PC(sp^3)HP]PdBr (4)

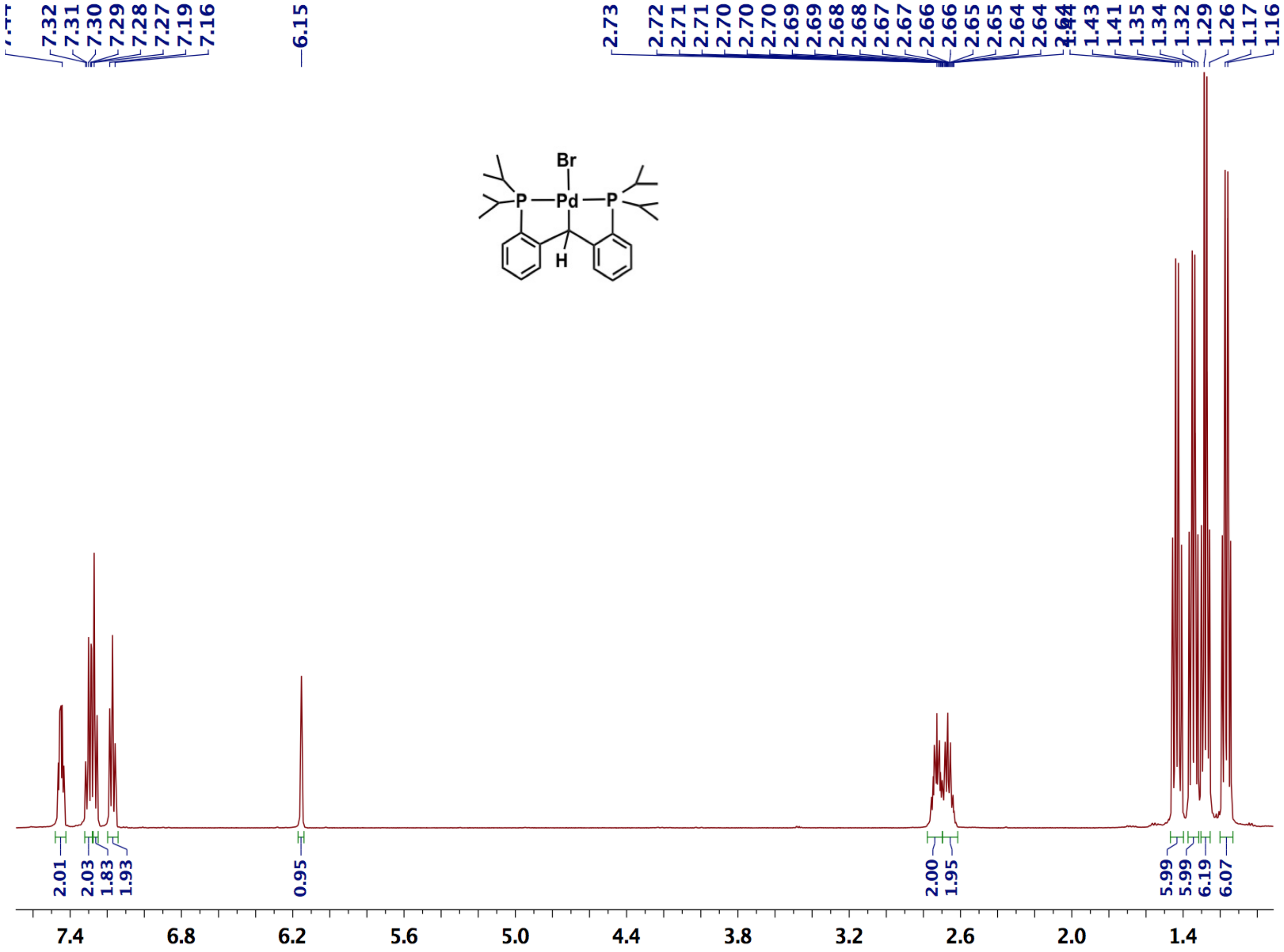

Figure S29. ^{1}H NMR spectrum for [PC(sp^3)HP]PdBr (**4**).

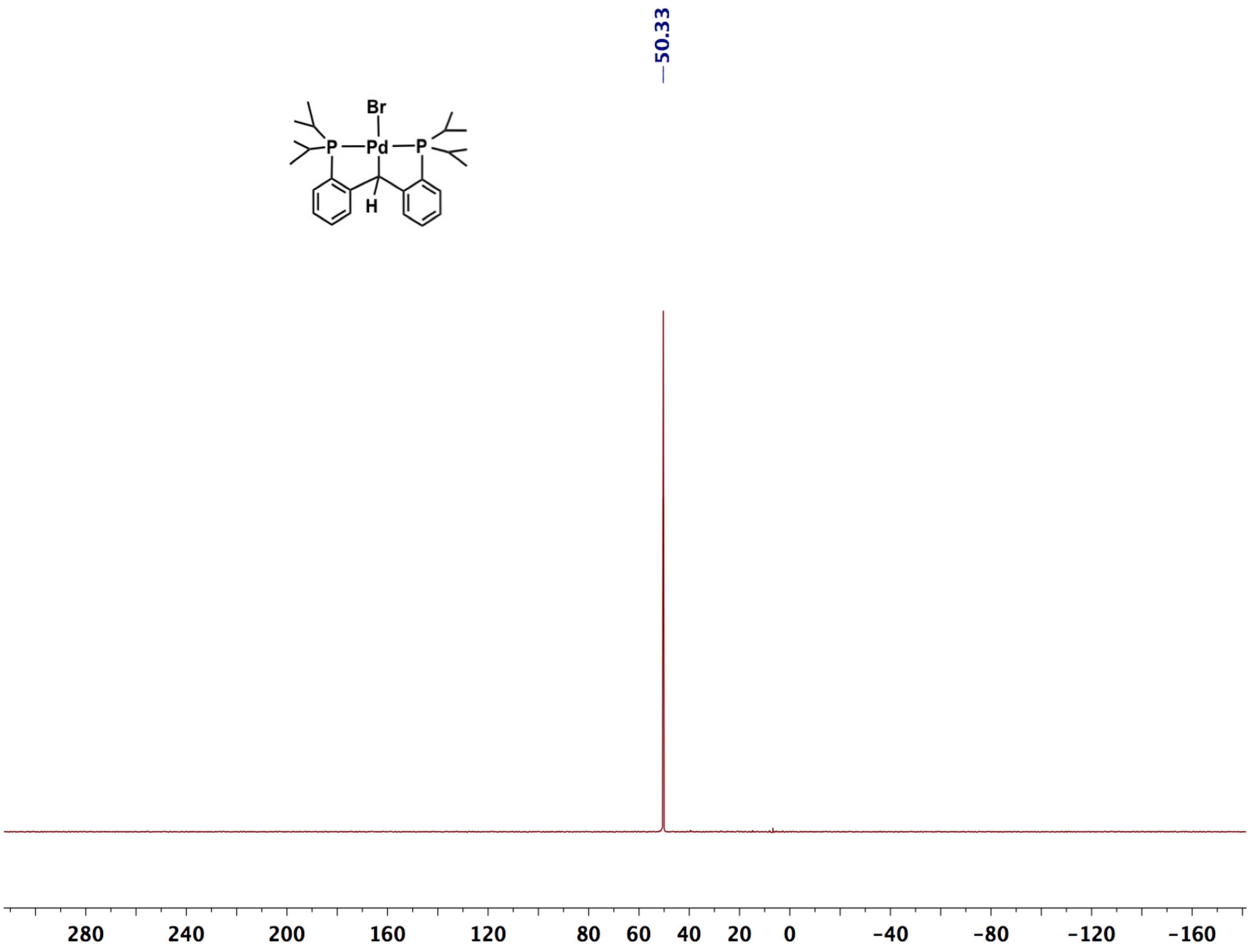

Figure S30. $^{31}P\{^{1}H\}$ NMR spectrum for [PC(sp^{3})HP]PdBr (**4**).

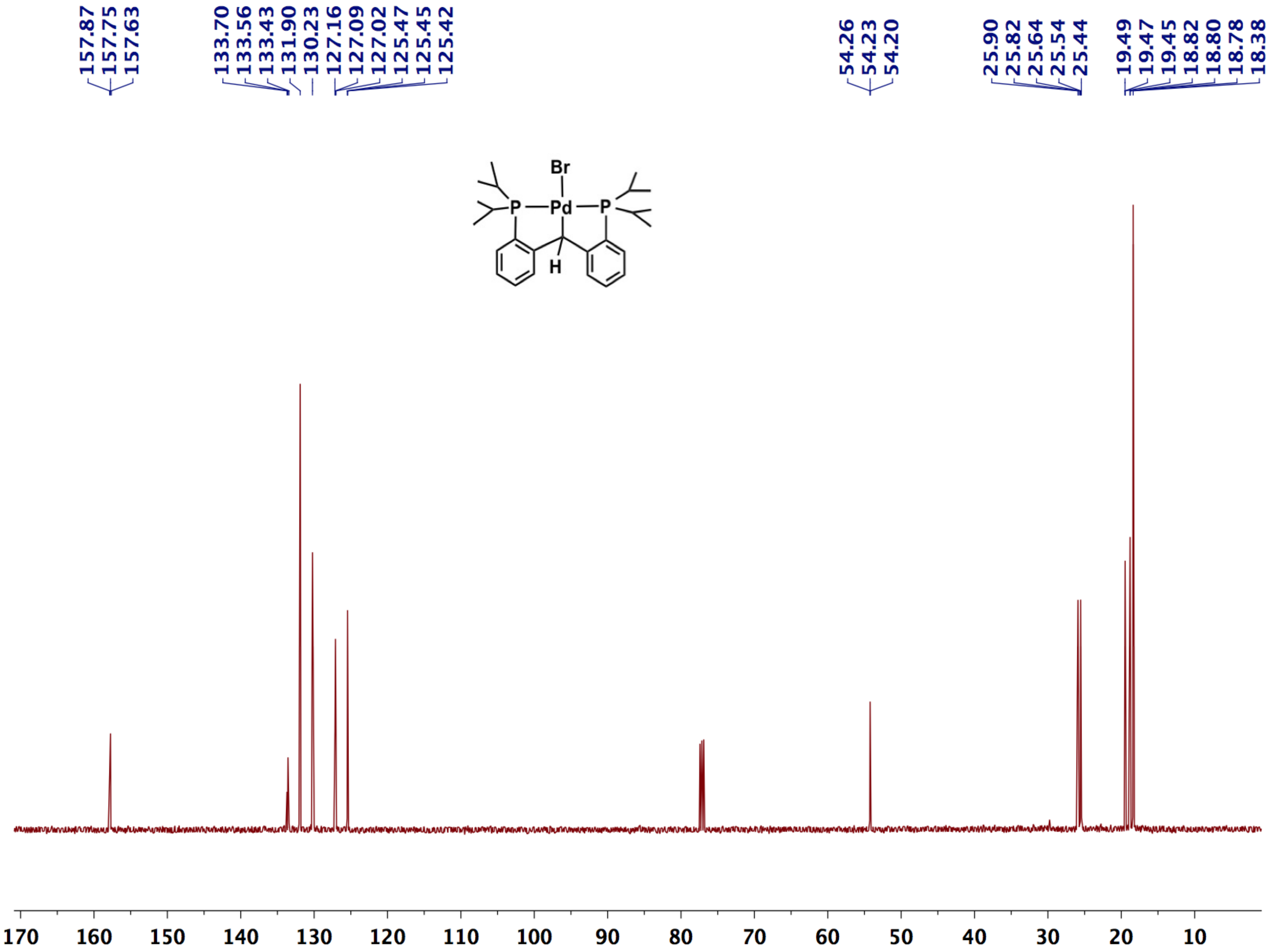

Figure S31. $^{13}C\{^{1}H\}$ NMR spectrum for [PC(sp^3)HP]PdBr (**4**).

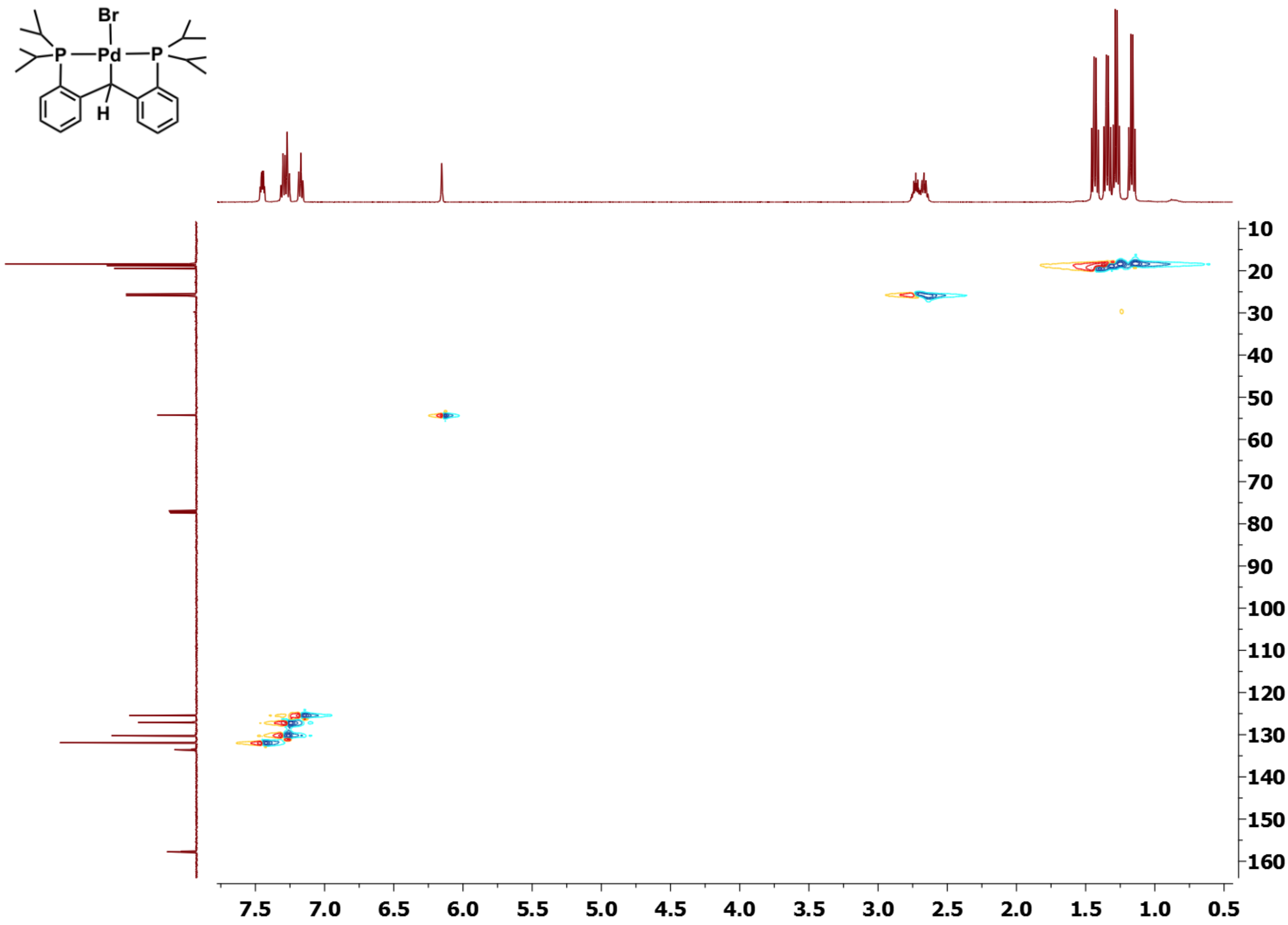

Figure S32. ^{1}H-^{13}C HSQC NMR spectrum for [PC(sp^{3})HP]PdBr (**4**).

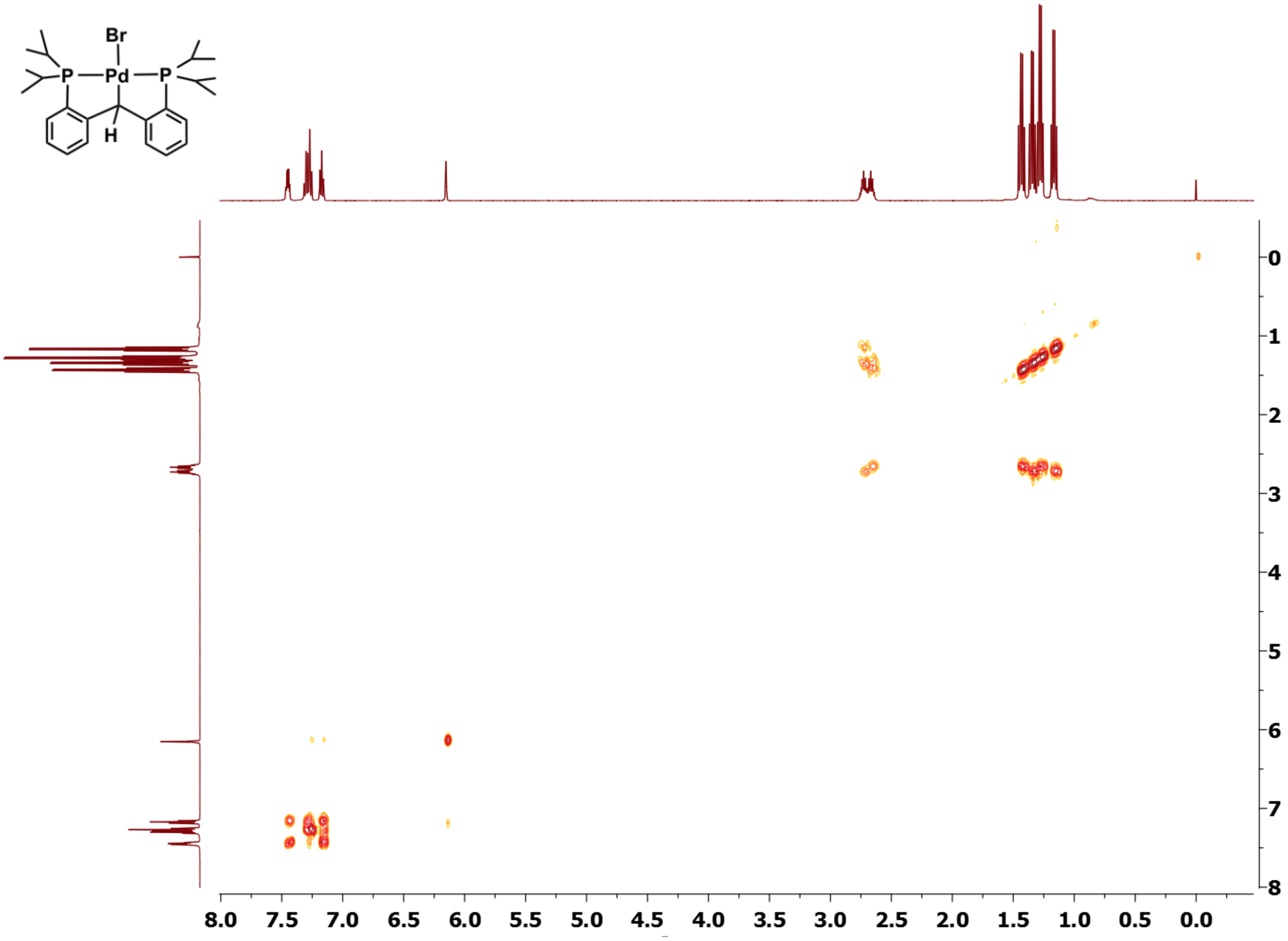

Figure S33. ^1H-^1H COSY NMR spectrum for [PC(sp^3)HP]PdBr (**4**).

7.3 NMR Spectra for $[PC(CH_2)P]Pd(PMe_3)$ (6)

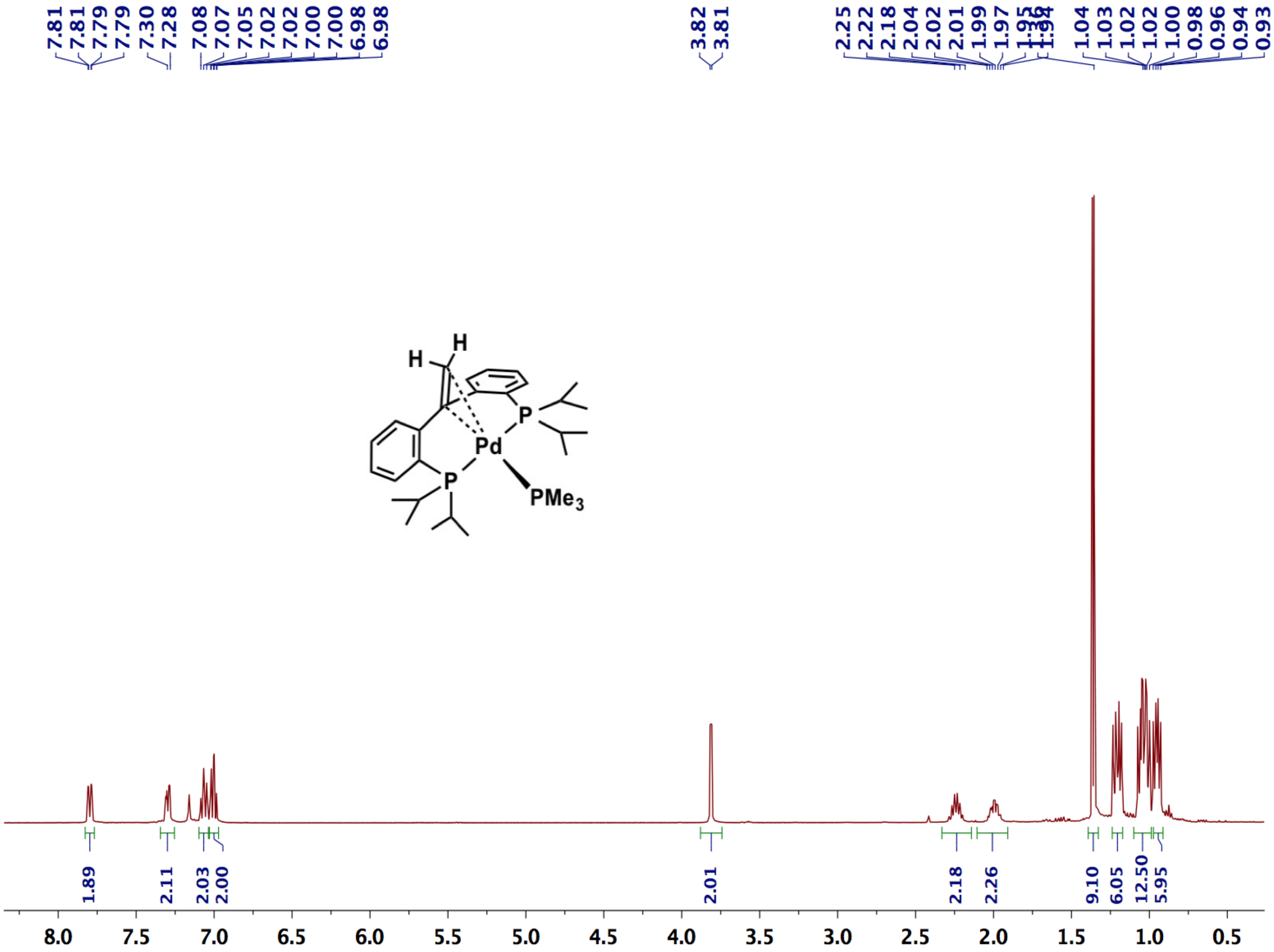

Figure S34. ^1H NMR spectrum for $[PC(CH_2)P]Pd(PMe_3)$ (**6**).

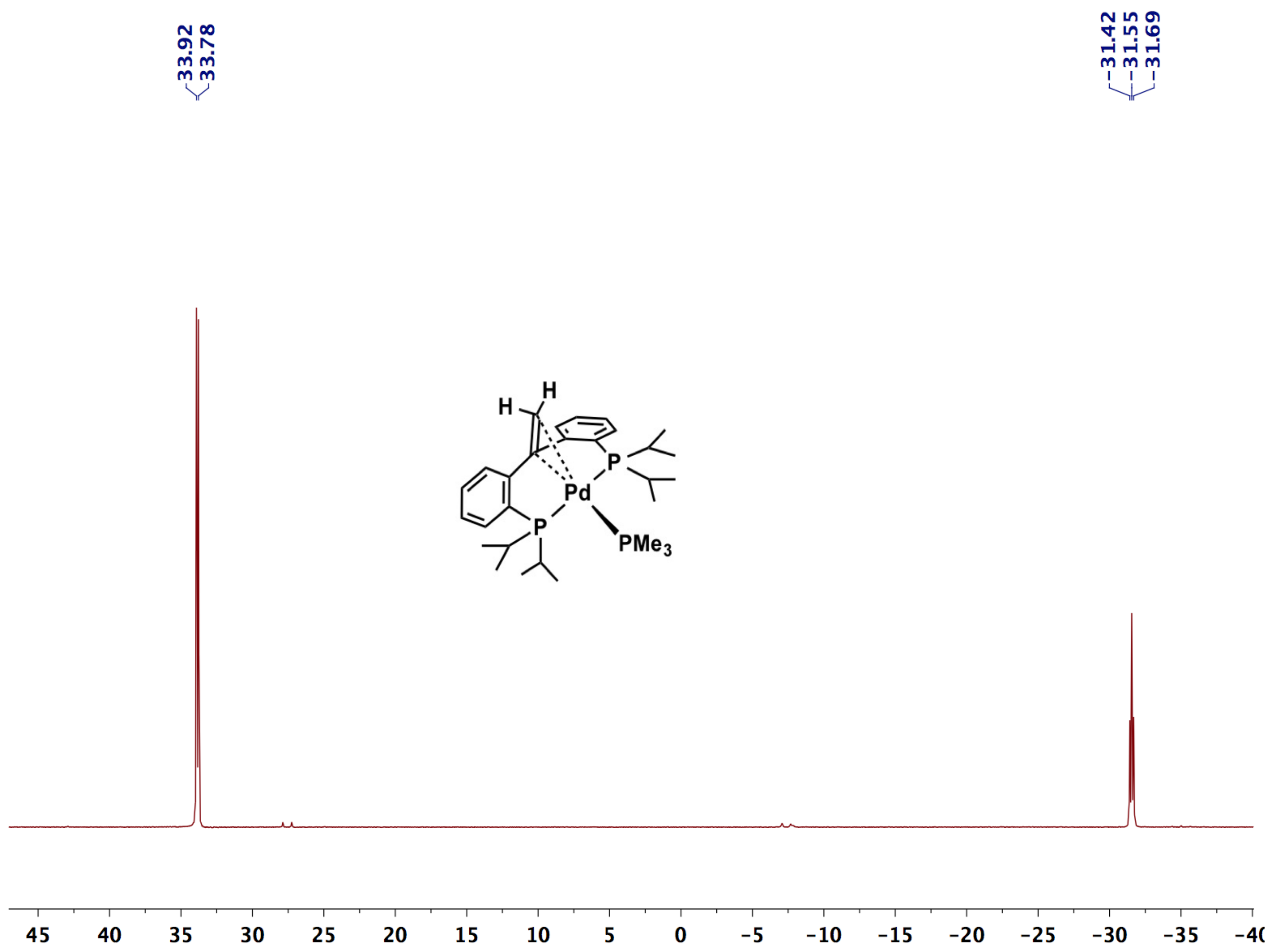

Figure S35. $^{31}P\{^{1}H\}$ NMR spectrum for $[PC(CH_2)P]Pd(PMe_3)$ (**6**).

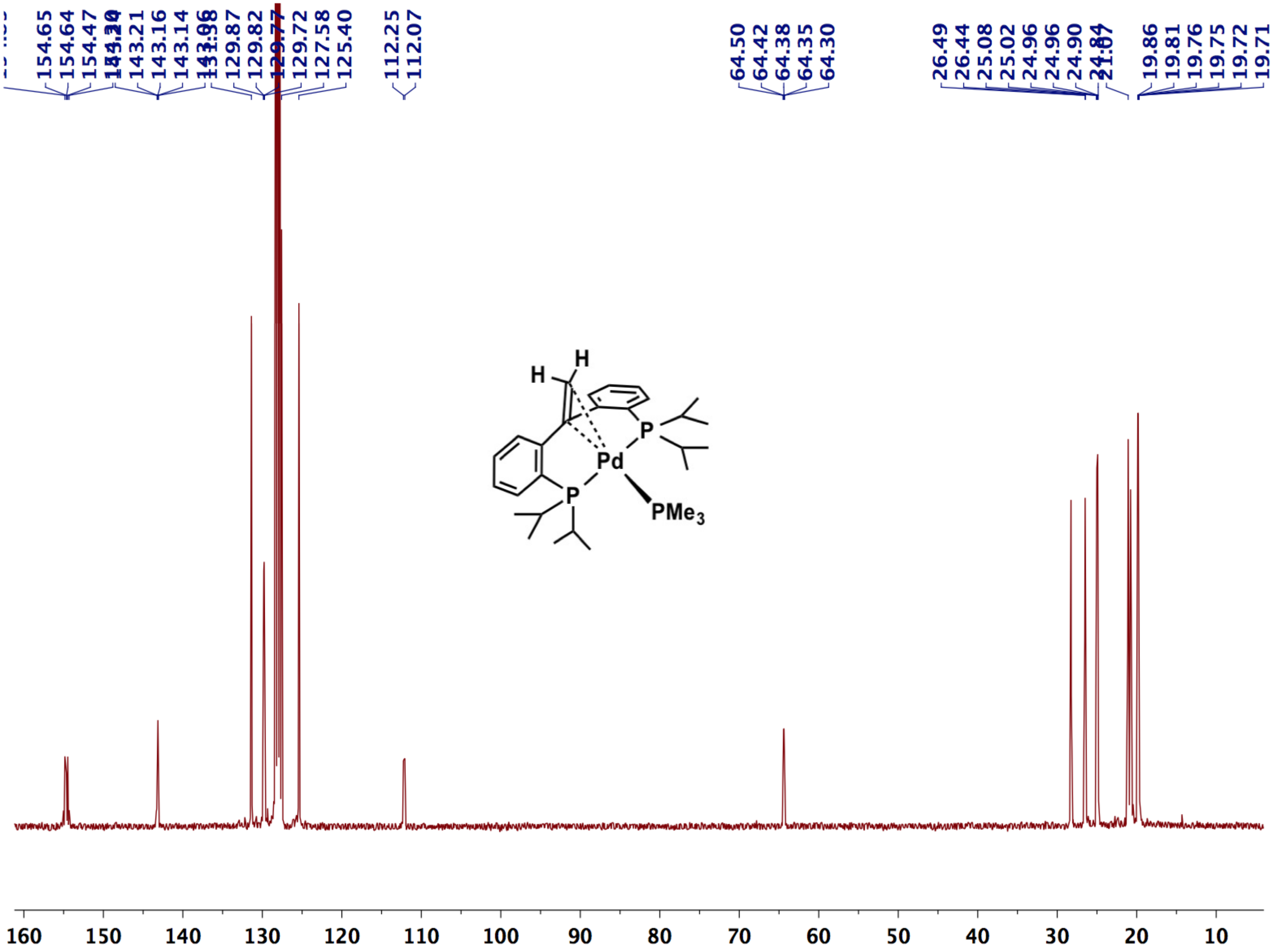

Figure S36. $^{13}C\{^1H\}$ NMR spectrum for $[PC(CH_2)P]Pd(PMe_3)$ (**6**).

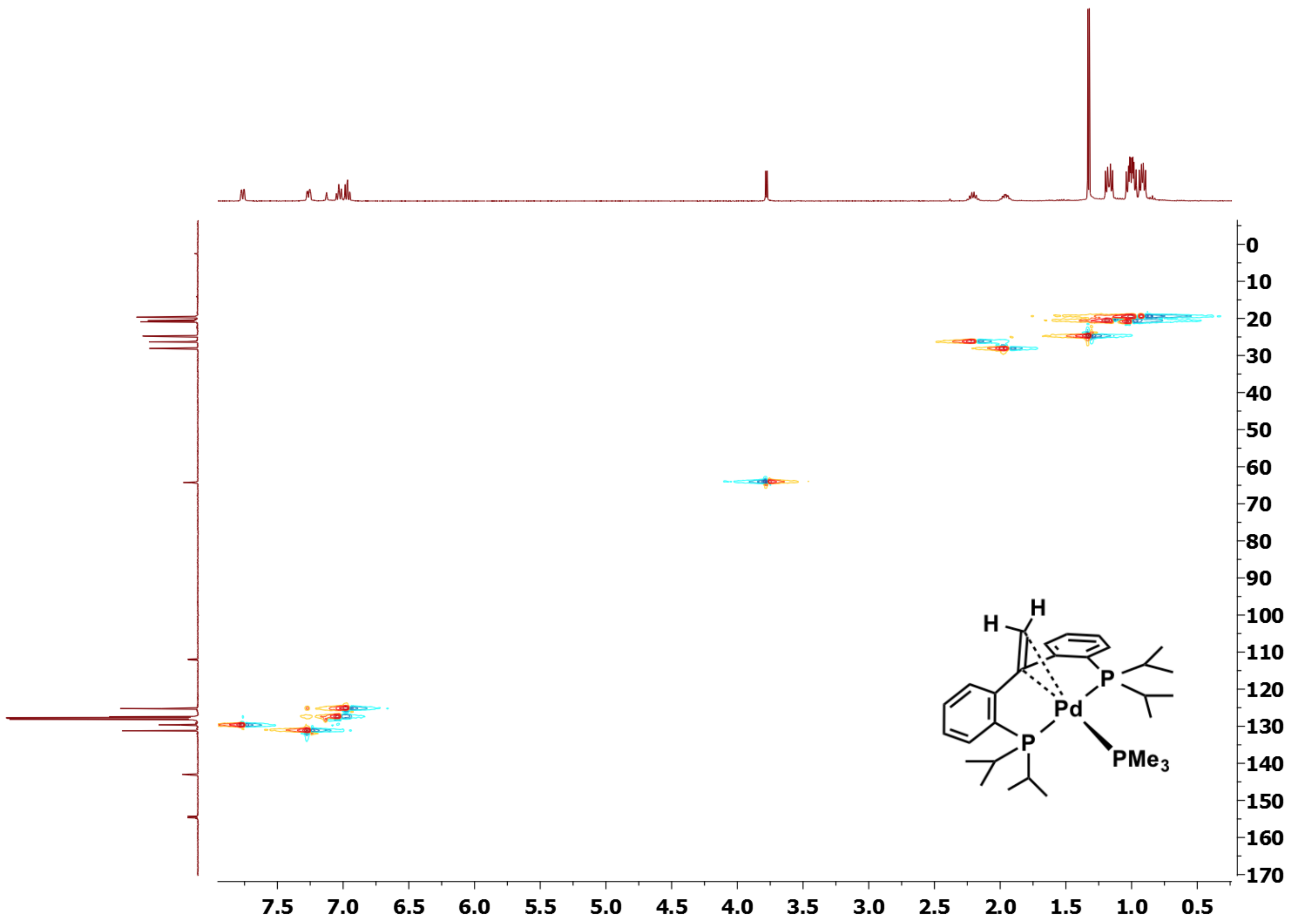

Figure S37. ^{1}H-^{13}C HSQC NMR spectrum for $[PC(CH_2)P]Pd(PMe_3)$ (**6**).

7.4 NMR Spectra for 1,1-bis(2-bromophenyl)ethan-1-ol

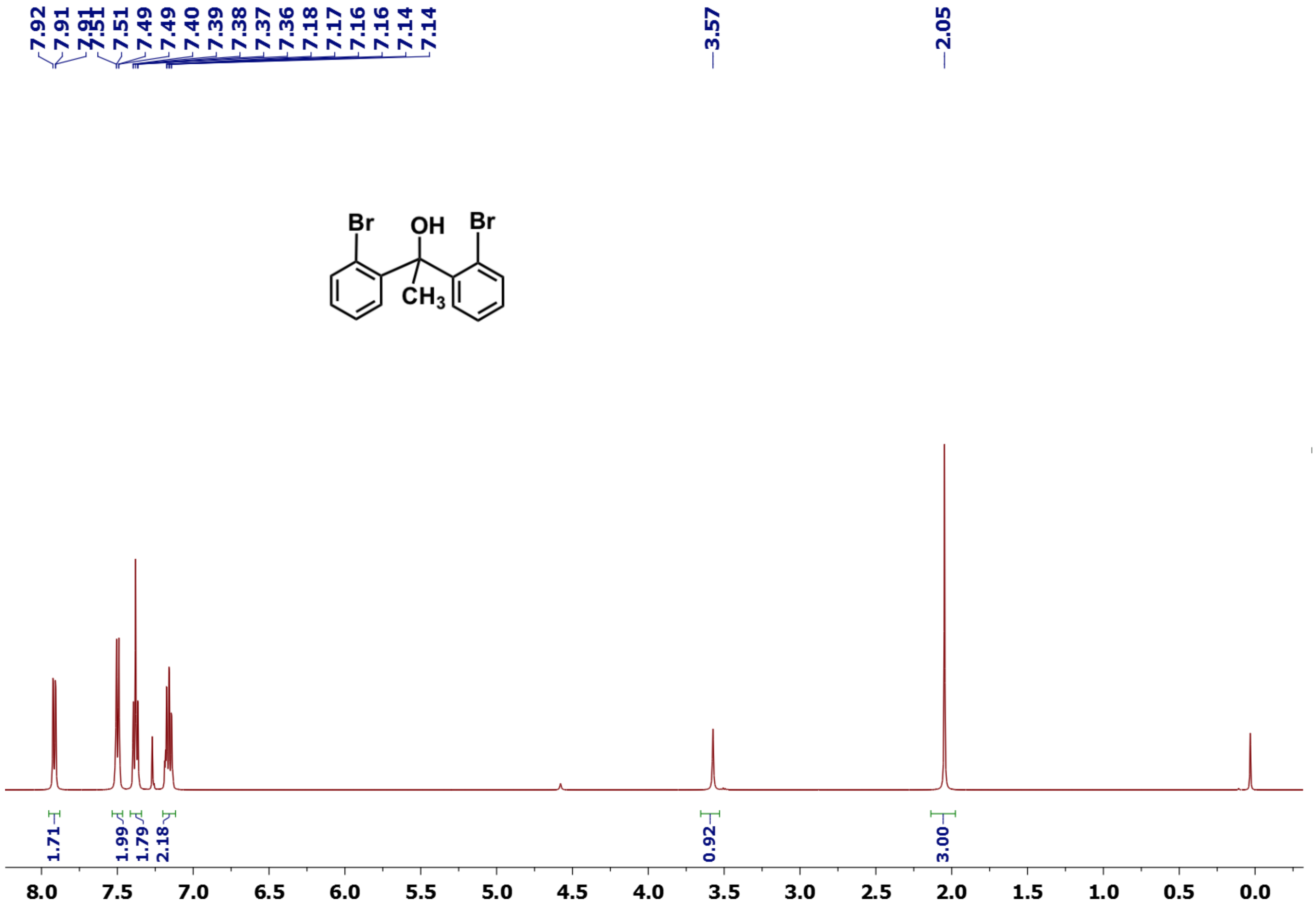

Figure S38. ^{1}H NMR spectrum for 1,1-bis(2-bromophenyl)ethan-1-ol.

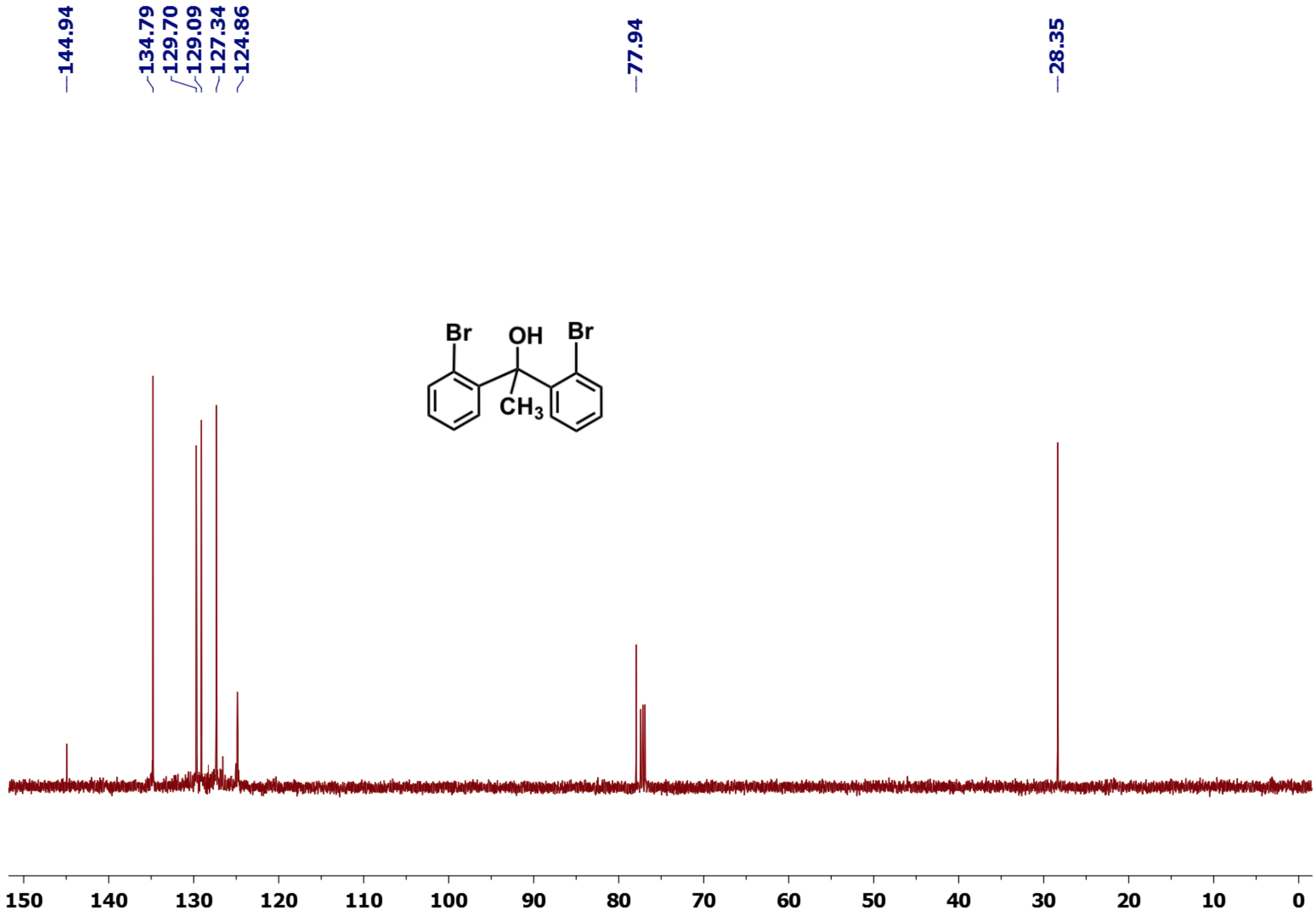

Figure S39. $^{13}C\{^{1}H\}$ NMR spectrum for 1,1-bis(2-bromophenyl)ethan-1-ol.

7.5 NMR Spectra for 1,1-bis(2-bromophenyl)ethane

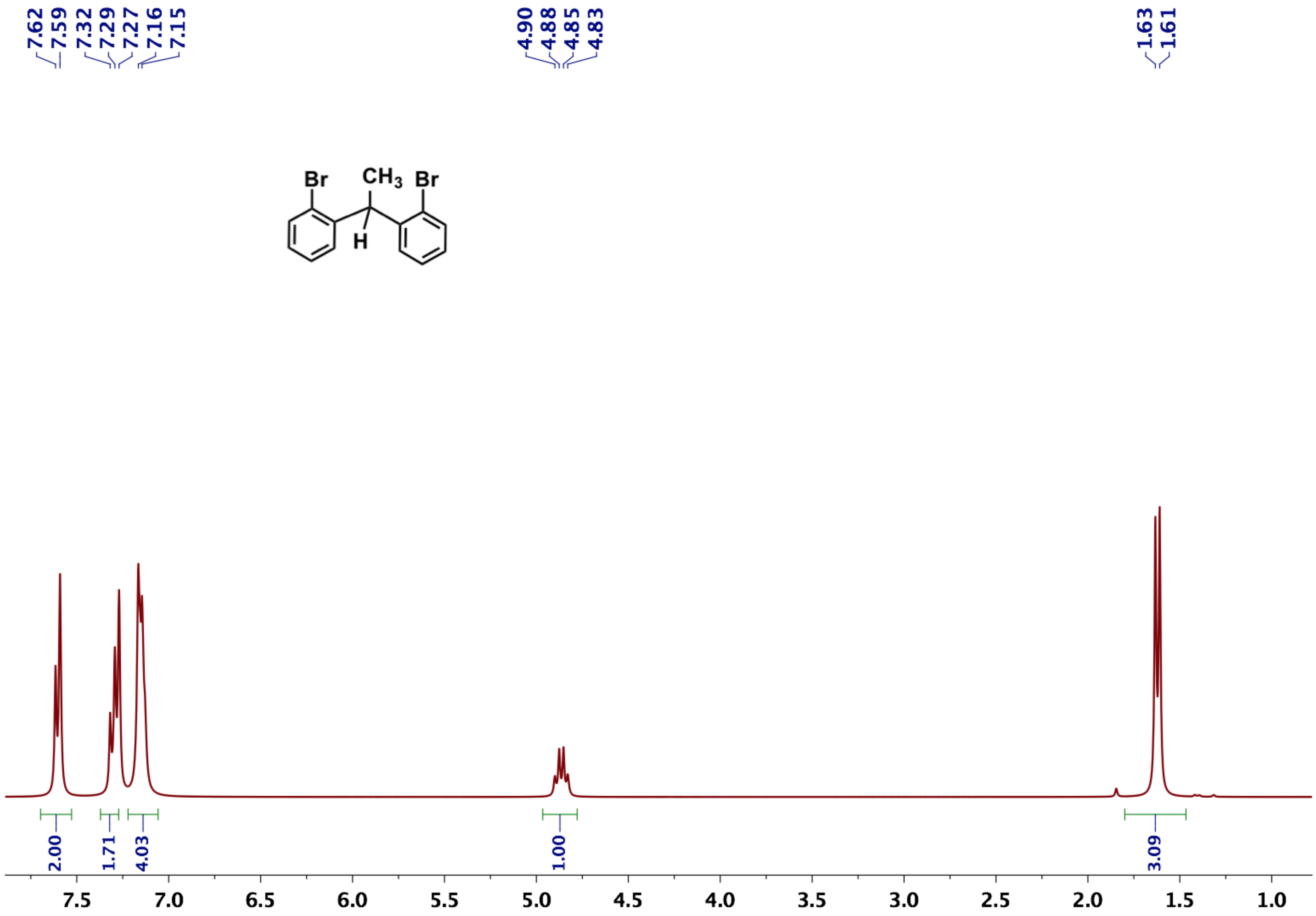

Figure S40. ^{1}H NMR spectrum for 1,1-bis(2-bromophenyl)ethane.

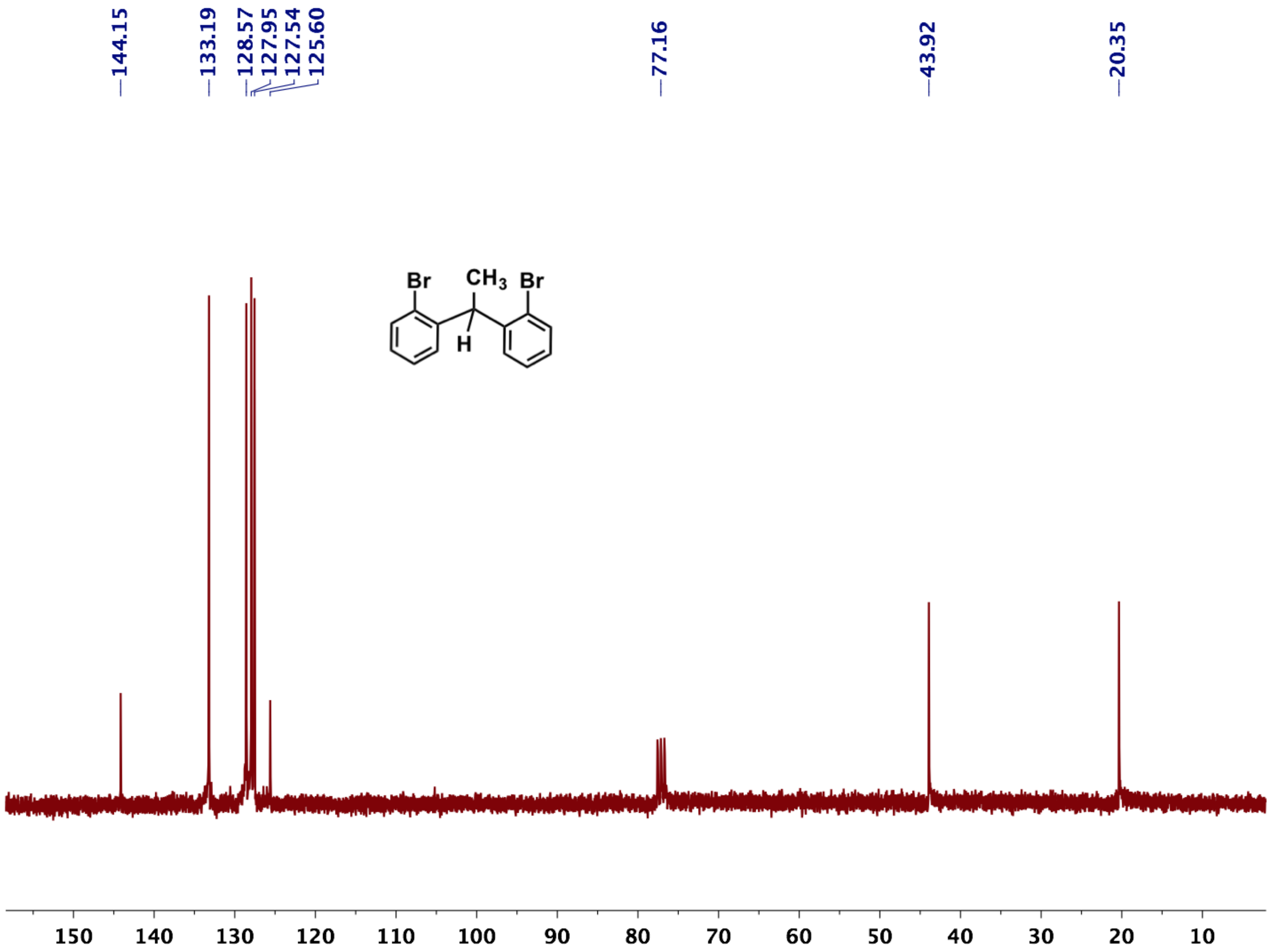

Figure S41. $^{13}C\{^1H\}$ NMR spectrum for 1,1-bis(2-bromophenyl)ethane.

7.6 NMR Spectra for PC(CH_3)HP (7)

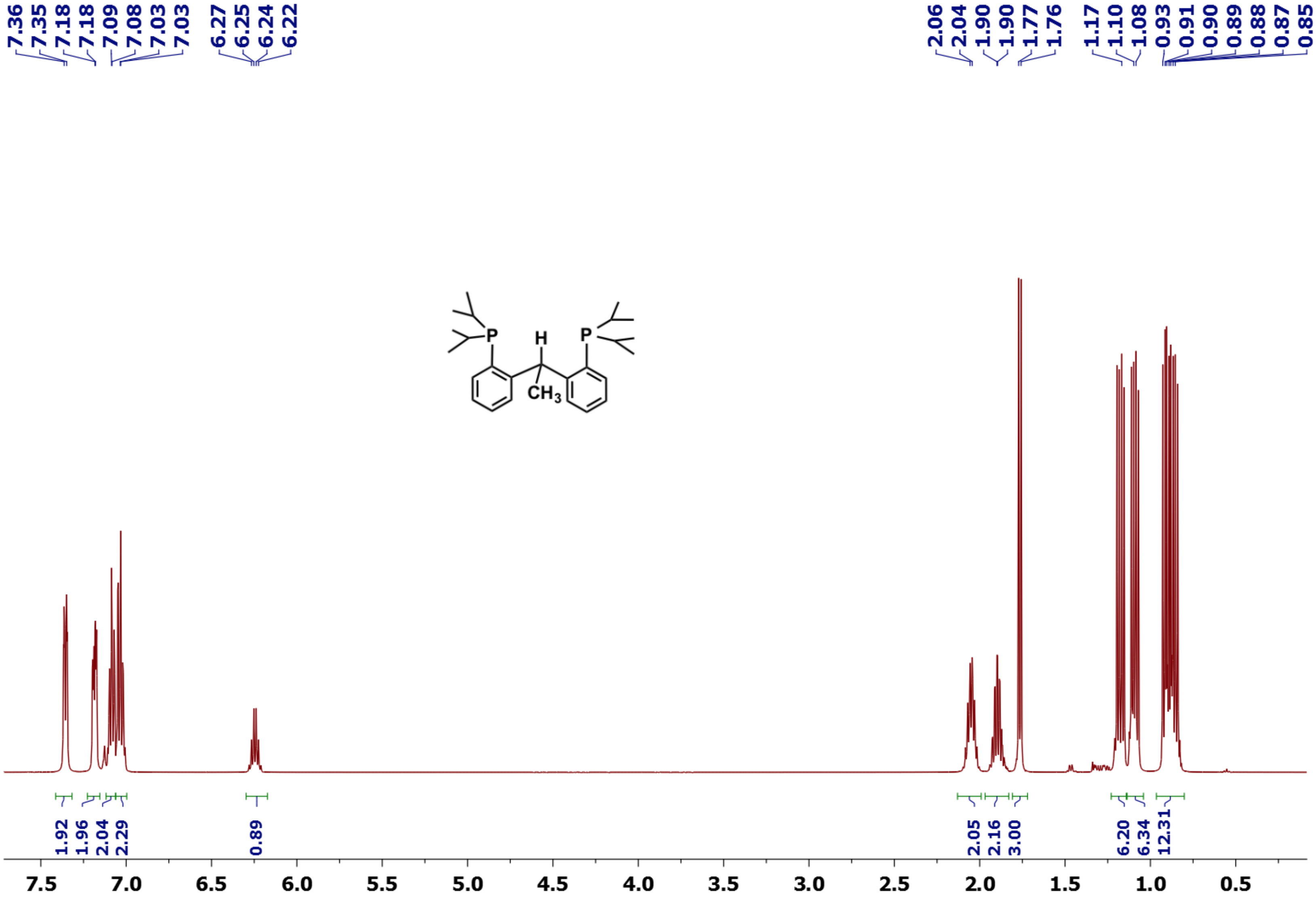

Figure S42. ^{1}H NMR spectrum for PC(CH_3)HP (**7**).

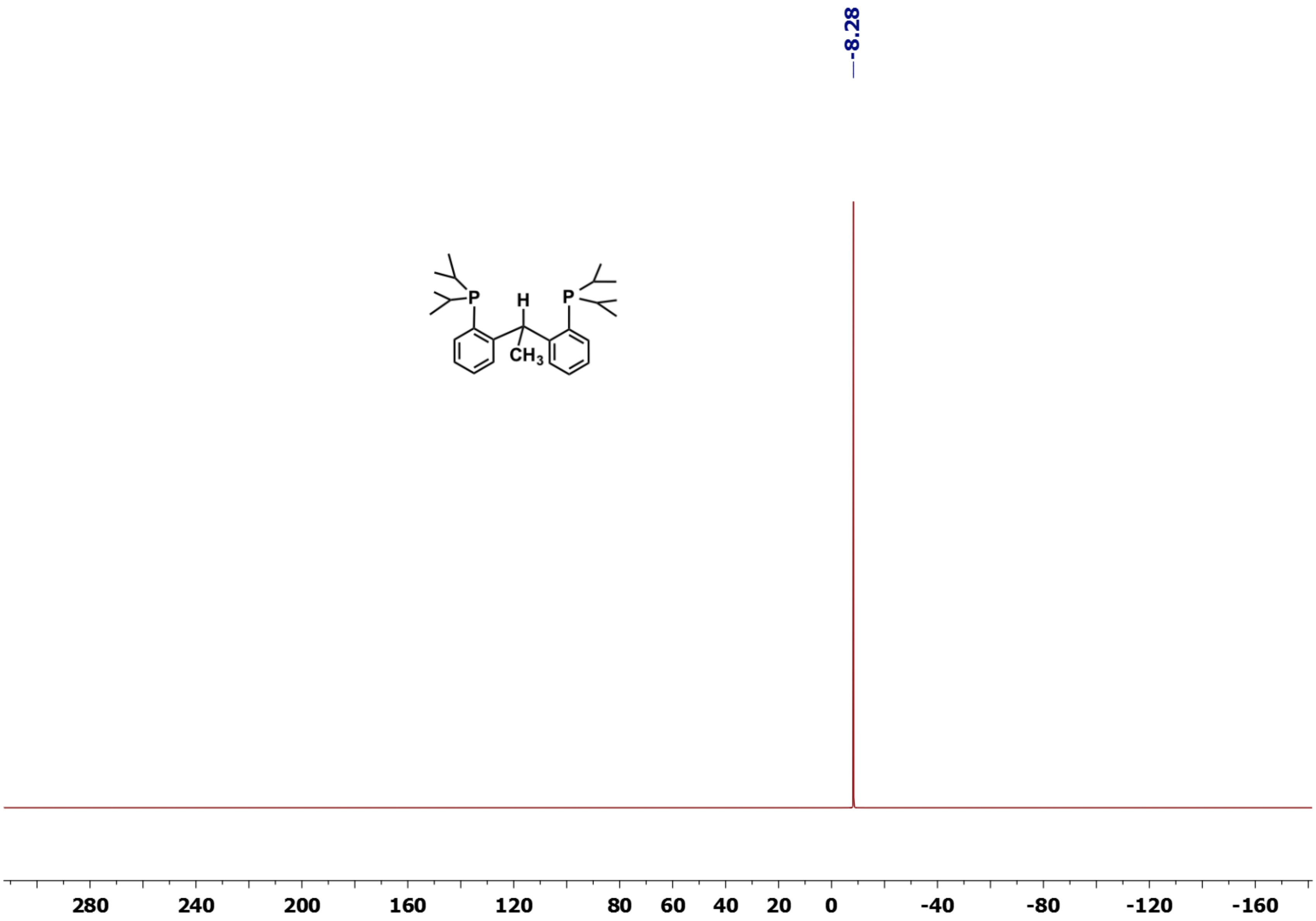

Figure S43. $^{31}P\{^1H\}$ NMR spectrum for $PC(CH_3)HP$ (**7**).

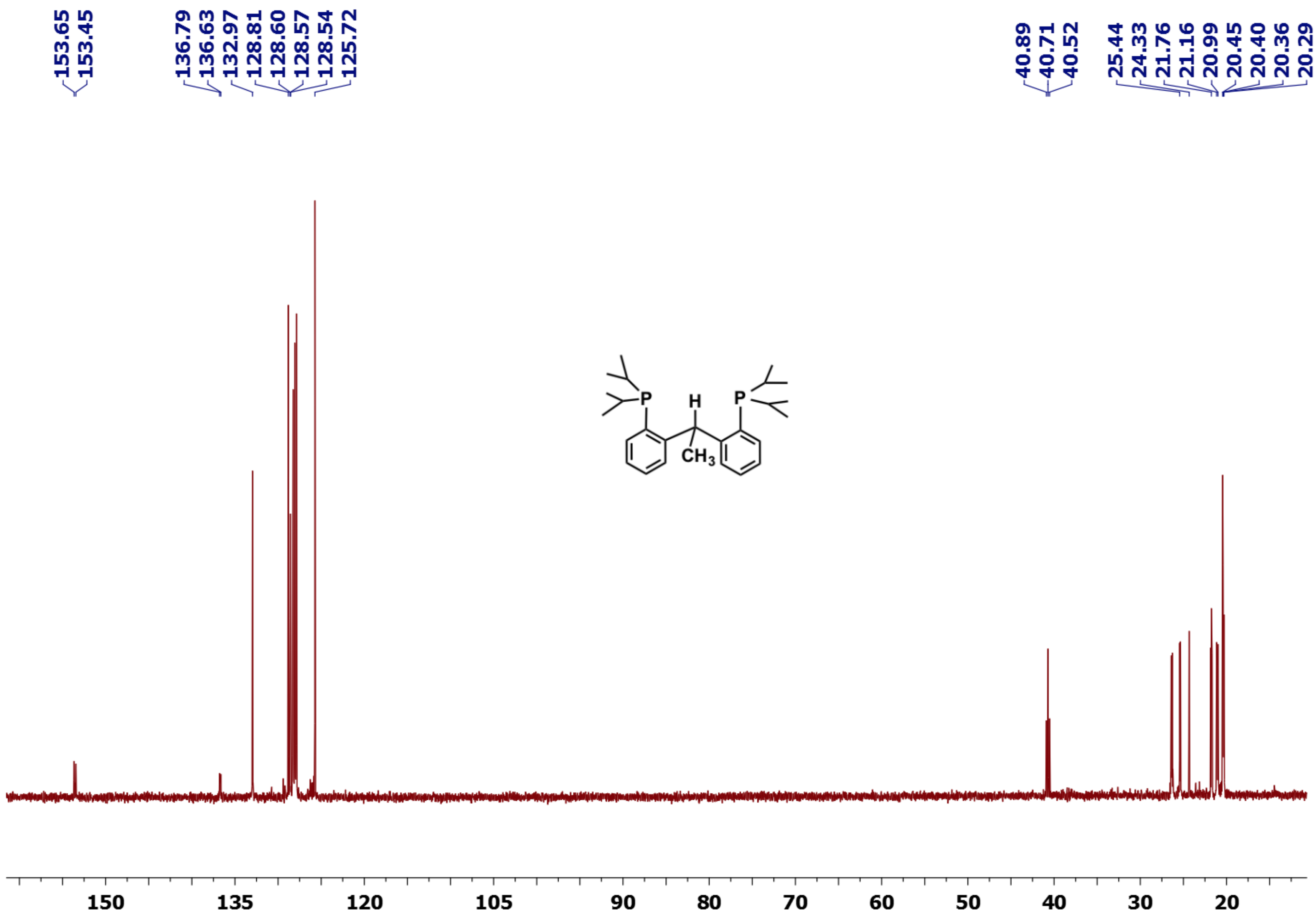

Figure S44. $^{13}C\{^{1}H\}$ NMR spectrum for $PC(CH_3)HP$ (**7**).

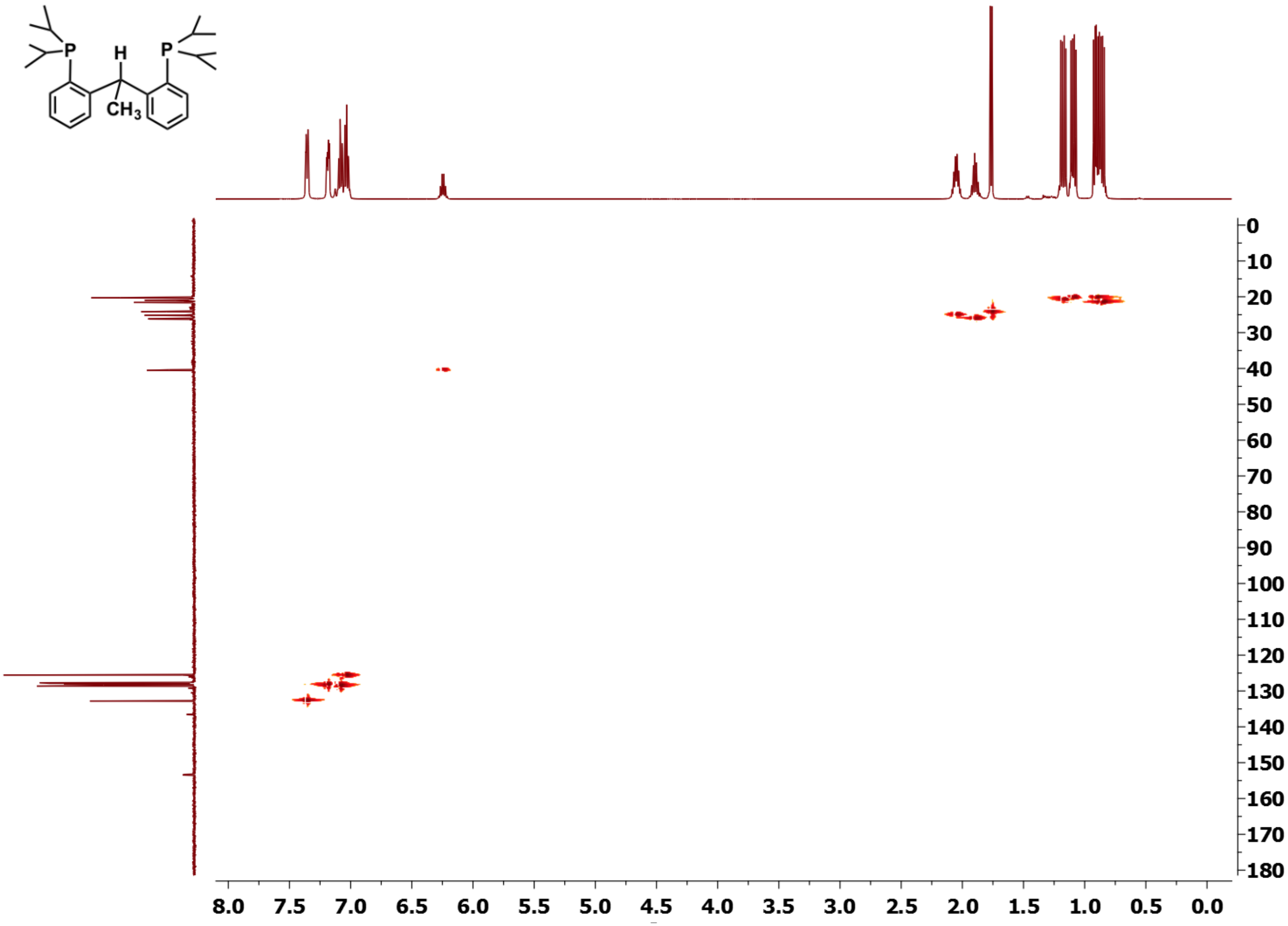

Figure S45. ^{1}H-^{13}C HSQC NMR spectrum for PC(CH_3)HP (**7**).

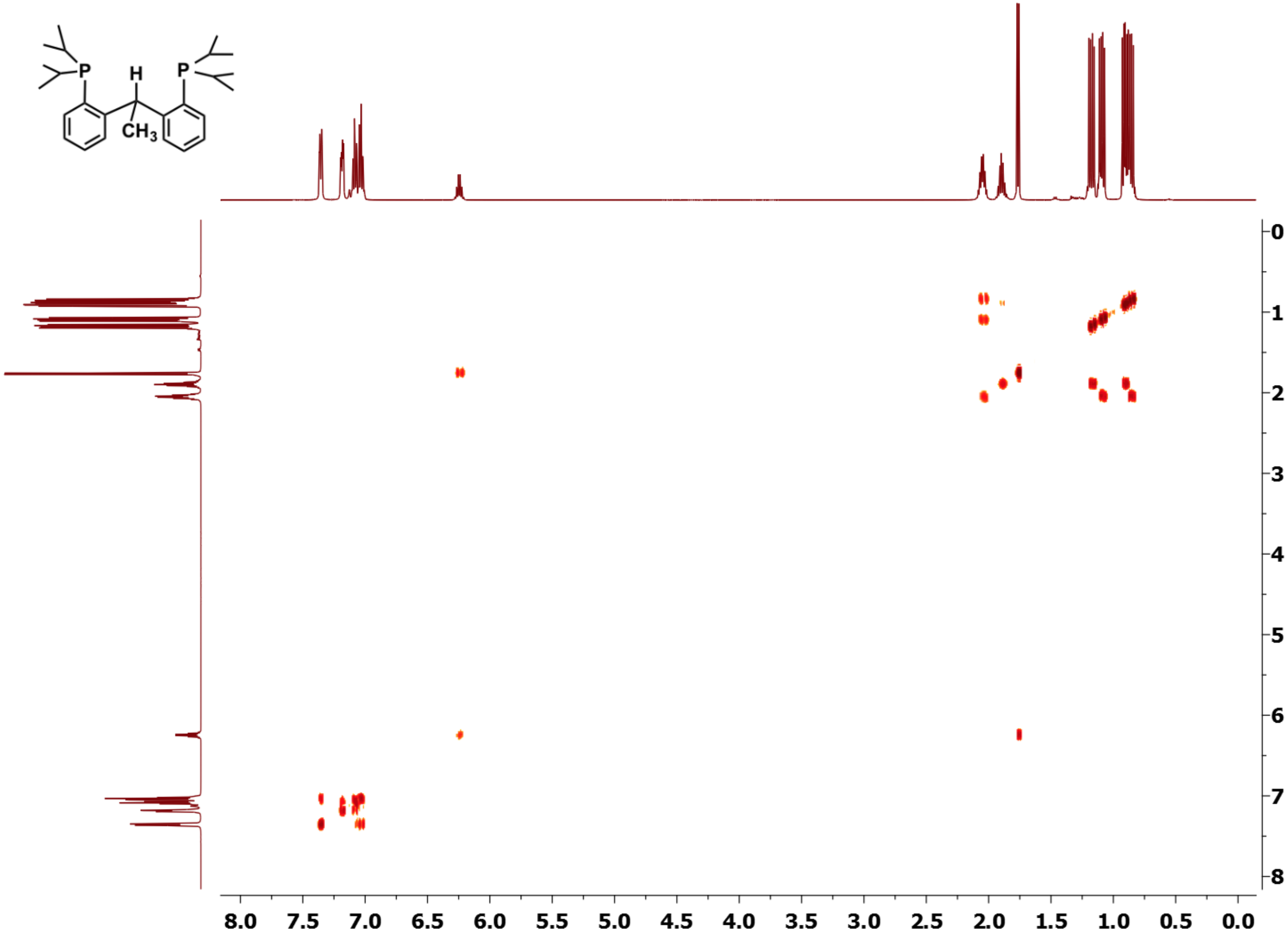

Figure S46. ^{1}H-^{1}H COSY NMR spectrum for PC(CH_3)HP (**7**).

7.7 NMR Spectra for $[PC(CH_3)HP]PdCl_2$ (8)

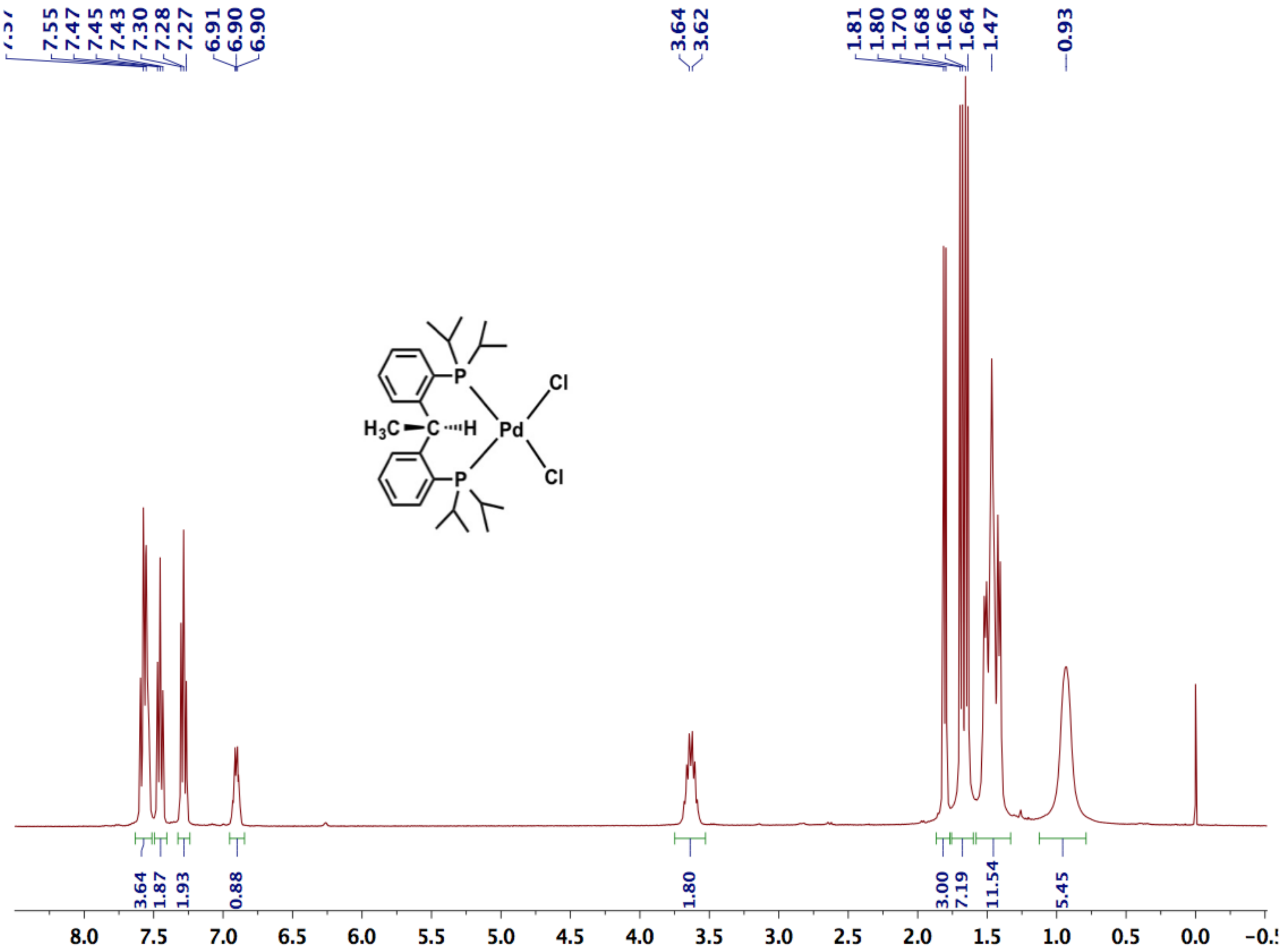

Figure S47. ^{1}H NMR spectrum (290 K) for $[PC(CH_3)HP]PdCl_2$ (**8**).

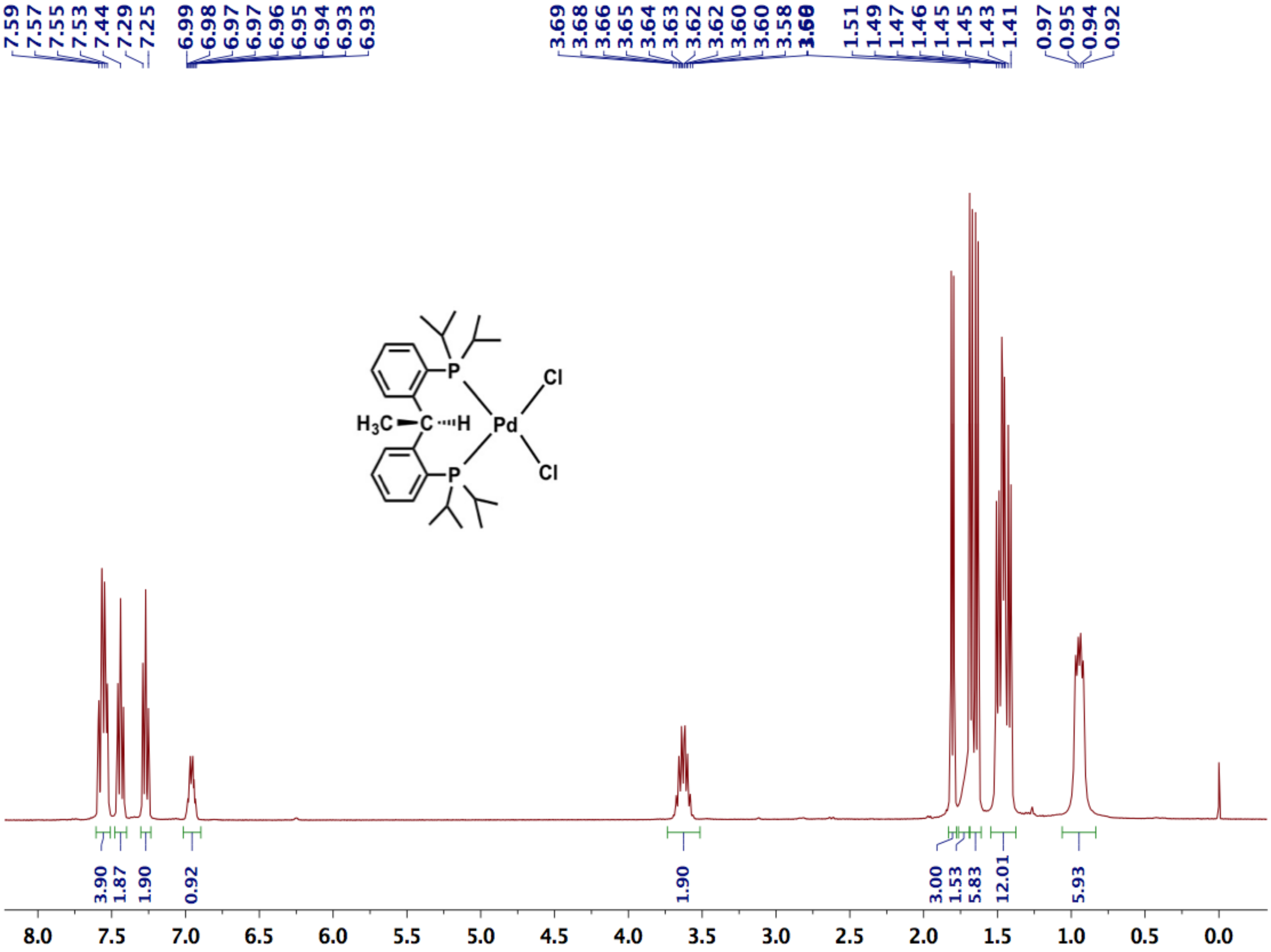

Figure S48. ^{1}H NMR spectrum (320 K) for $[PC(CH_3)HP]PdCl_2$ (**8**).

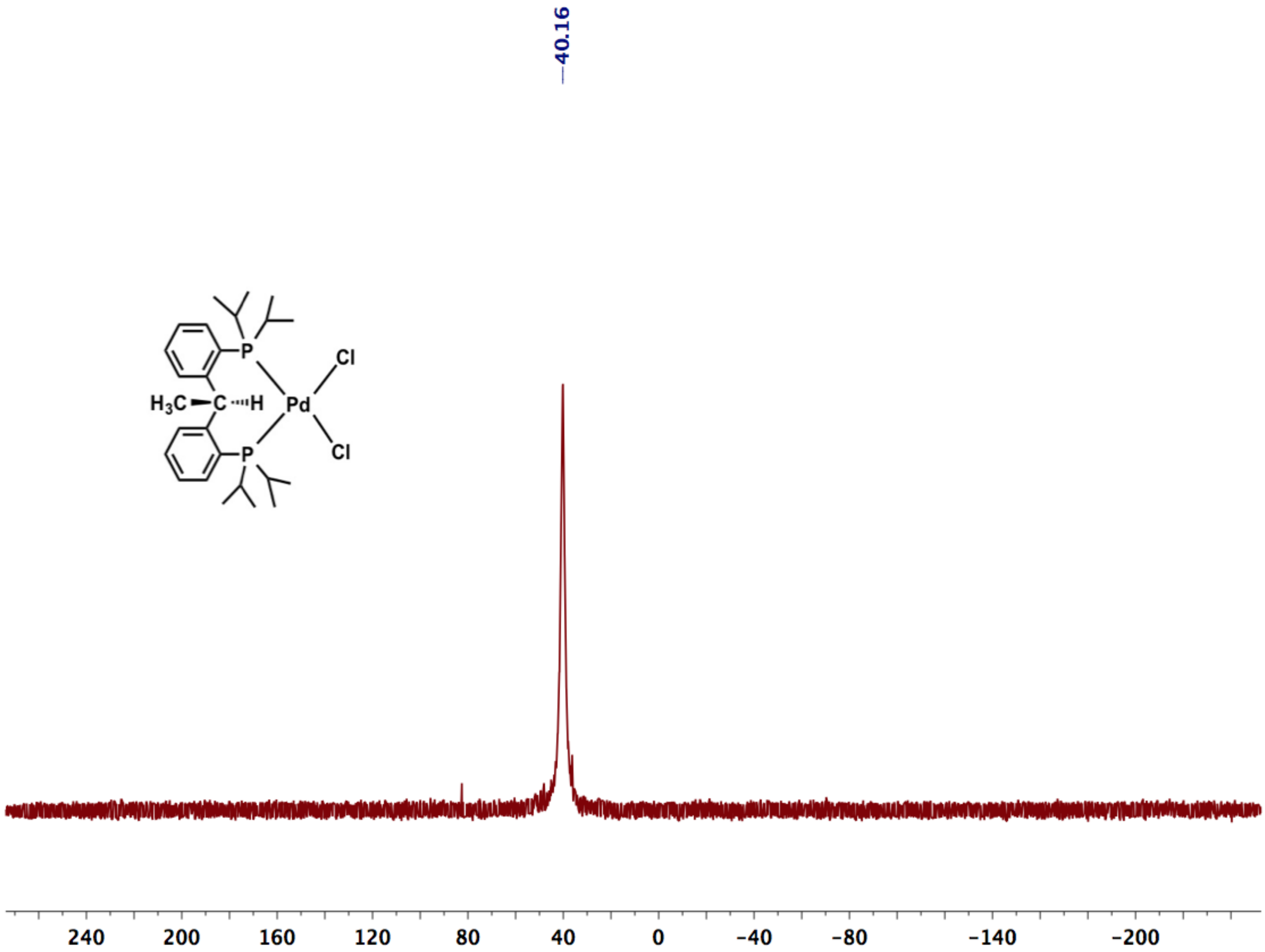

Figure S49. $^{31}P\{^{1}H\}$ NMR spectrum (290 K) for $[PC(CH_3)HP]PdCl_2$ (**8**).

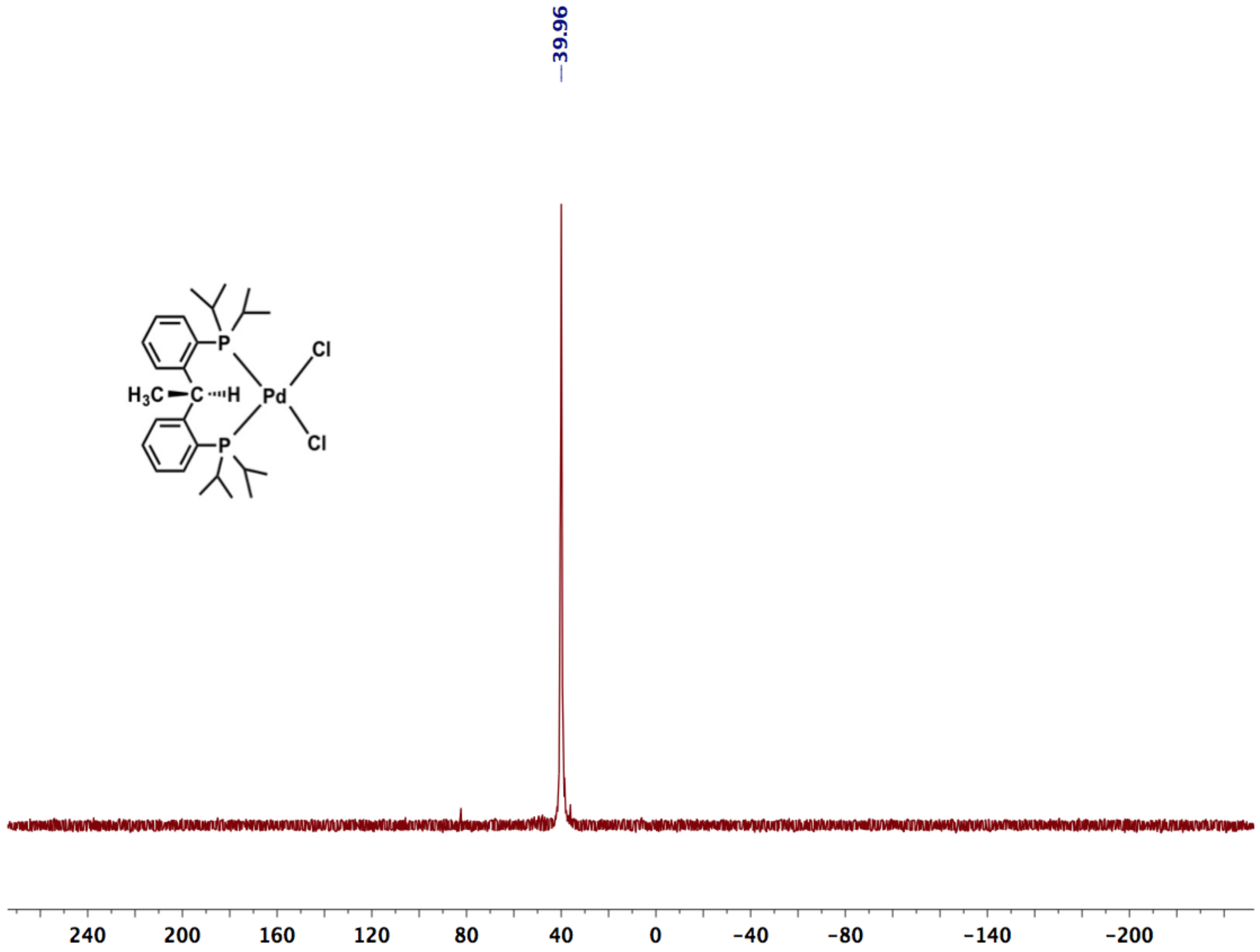

Figure S50. $^{31}P\{^{1}H\}$ NMR spectrum (320 K) for $[PC(CH_3)HP]PdCl_2$ (**8**).

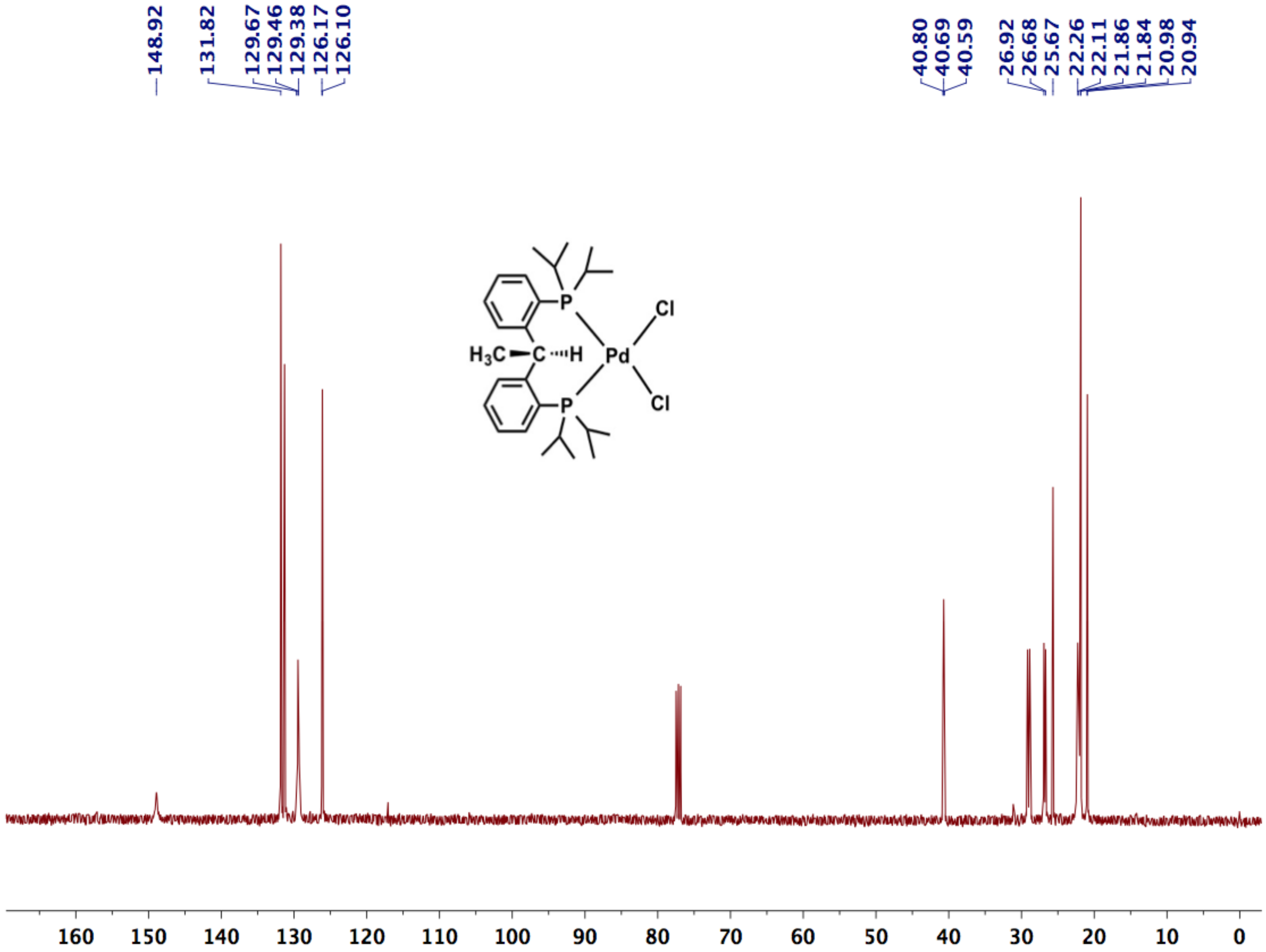

Figure S51. $^{13}C\{^{1}H\}$ NMR spectrum (290 K) for $[PC(CH_3)HP]PdCl_2$ (**8**).

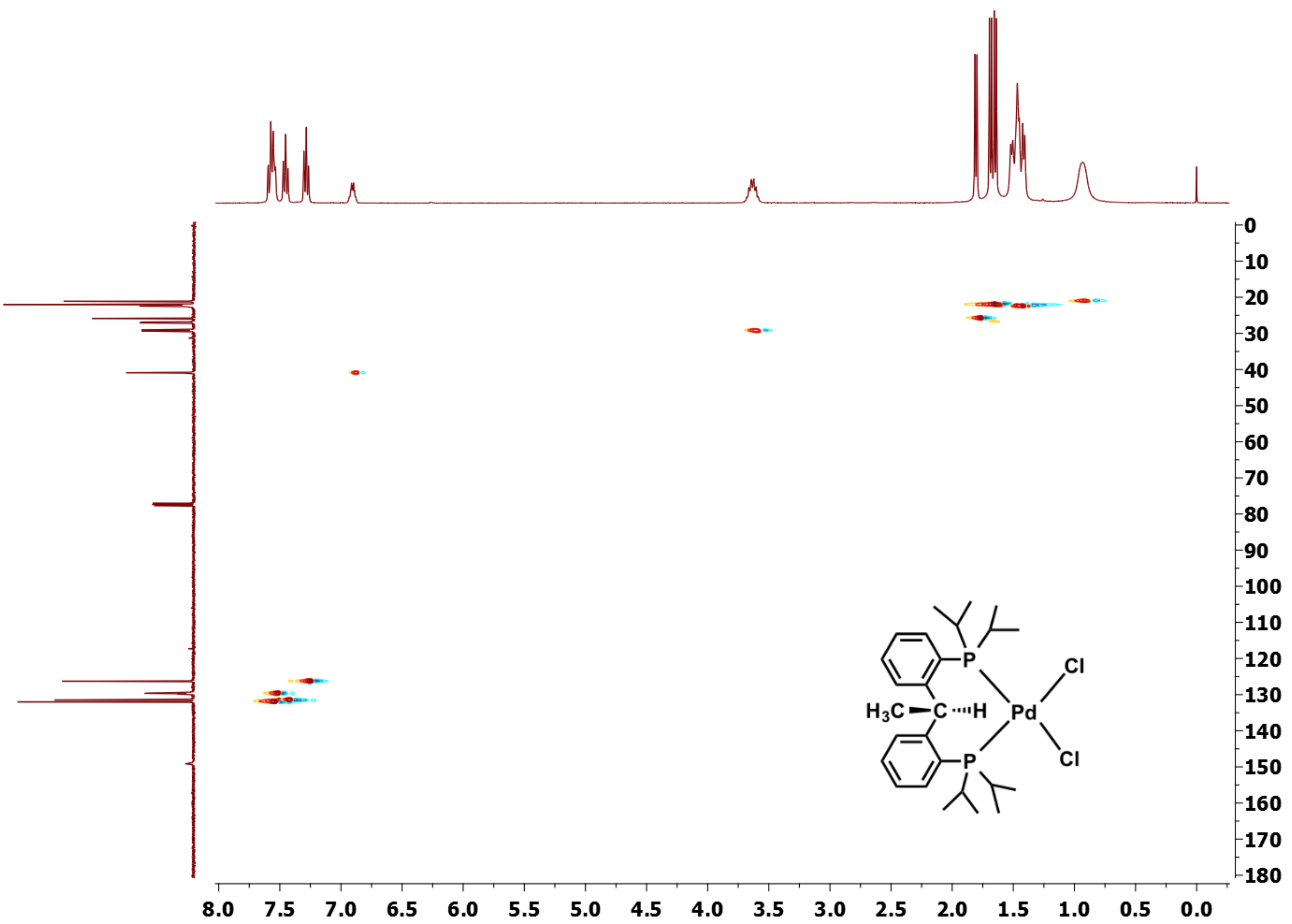

Figure S52. ^{1}H-^{13}C HSQC NMR spectrum (290 K) for $[PC(CH_3)HP]PdCl_2$ (**8**).

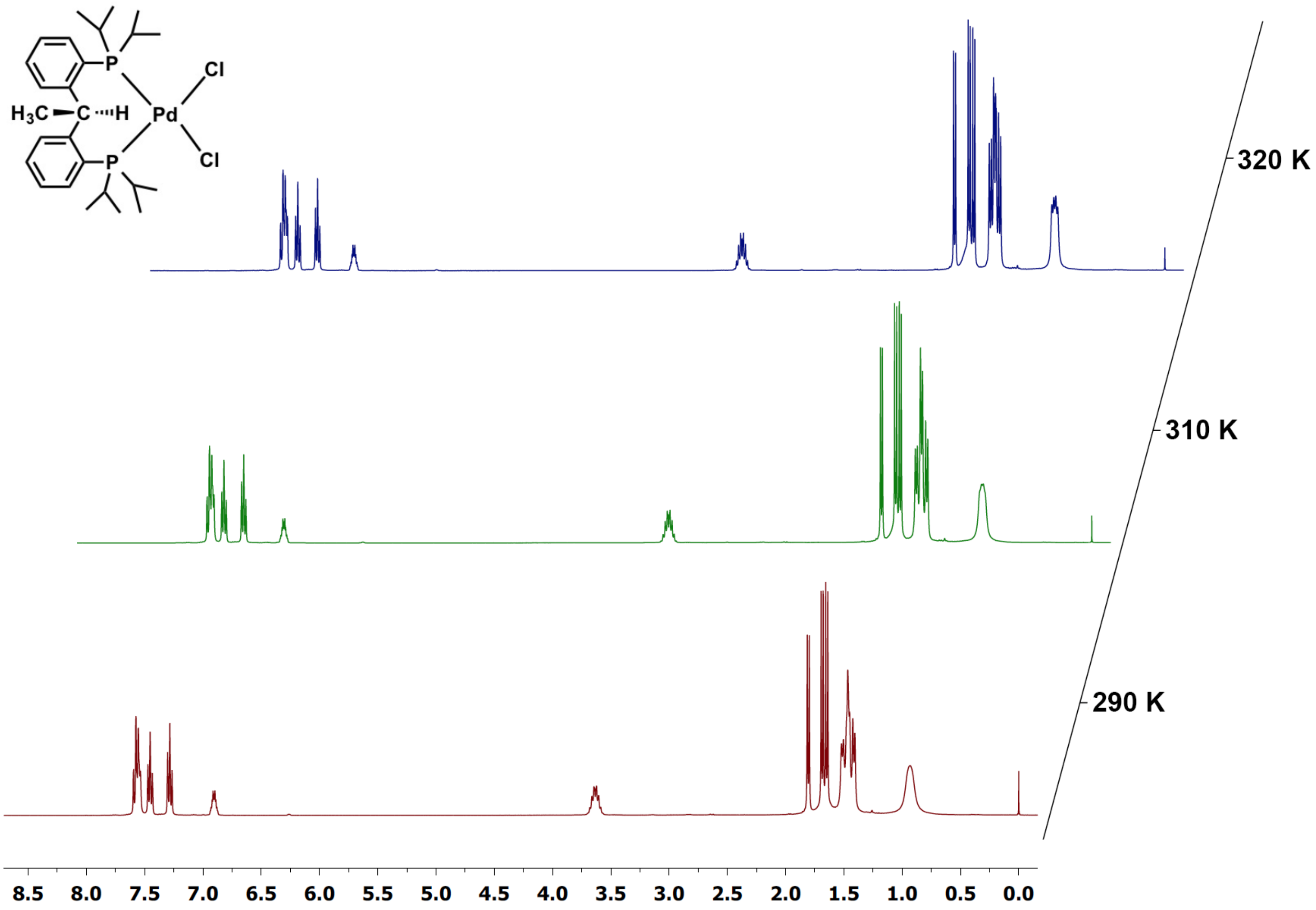

Figure S53. Variable temperature ^{1}H NMR spectra for [PC(CH_3)HP]PdCl_2 (**8**).

7.8 NMR Spectra for [PC(CH_3)P]PdCl (9)

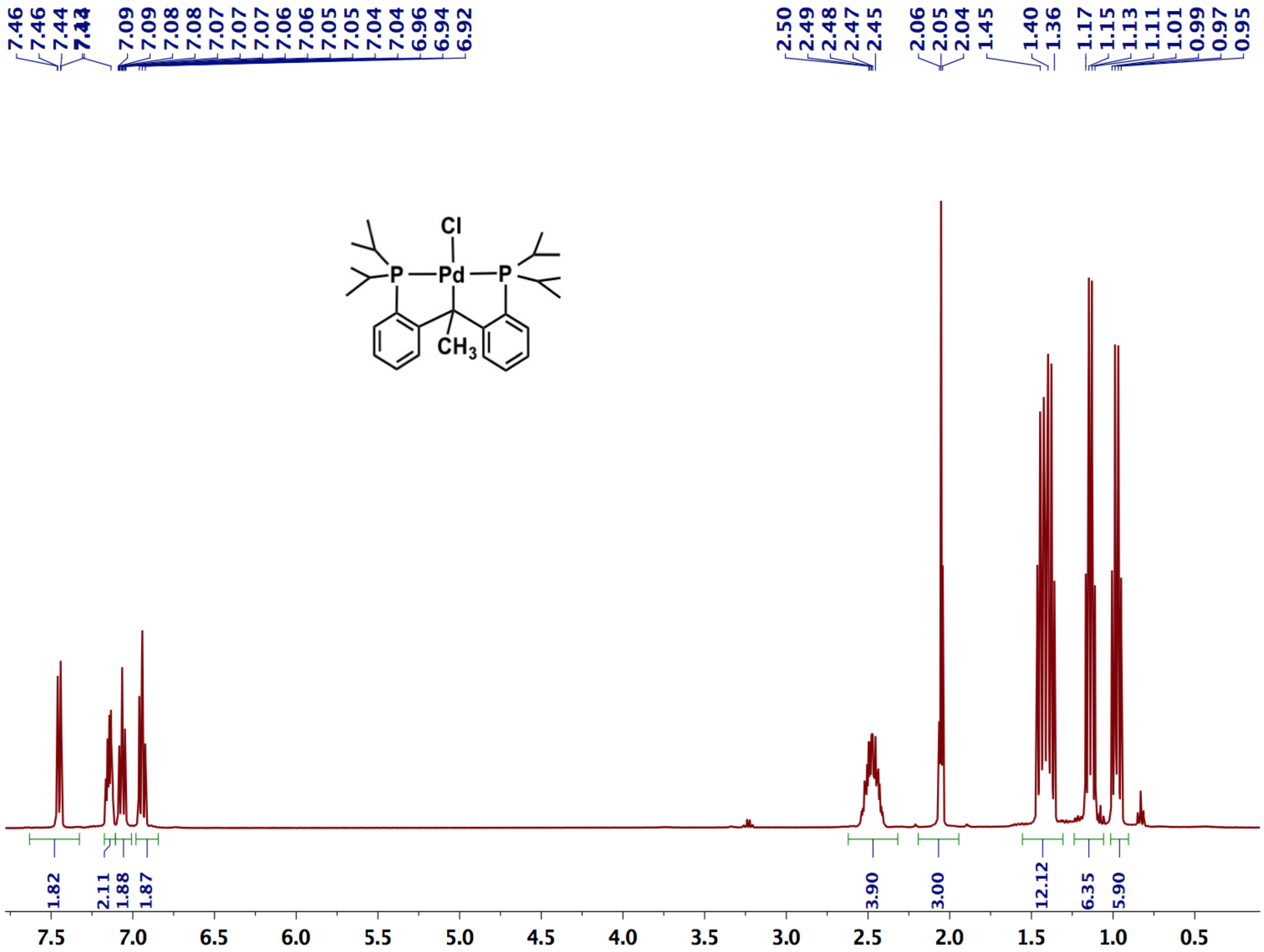

Figure S54. ^{1}H NMR spectrum for [PC(CH_3)P]PdCl (**9**).

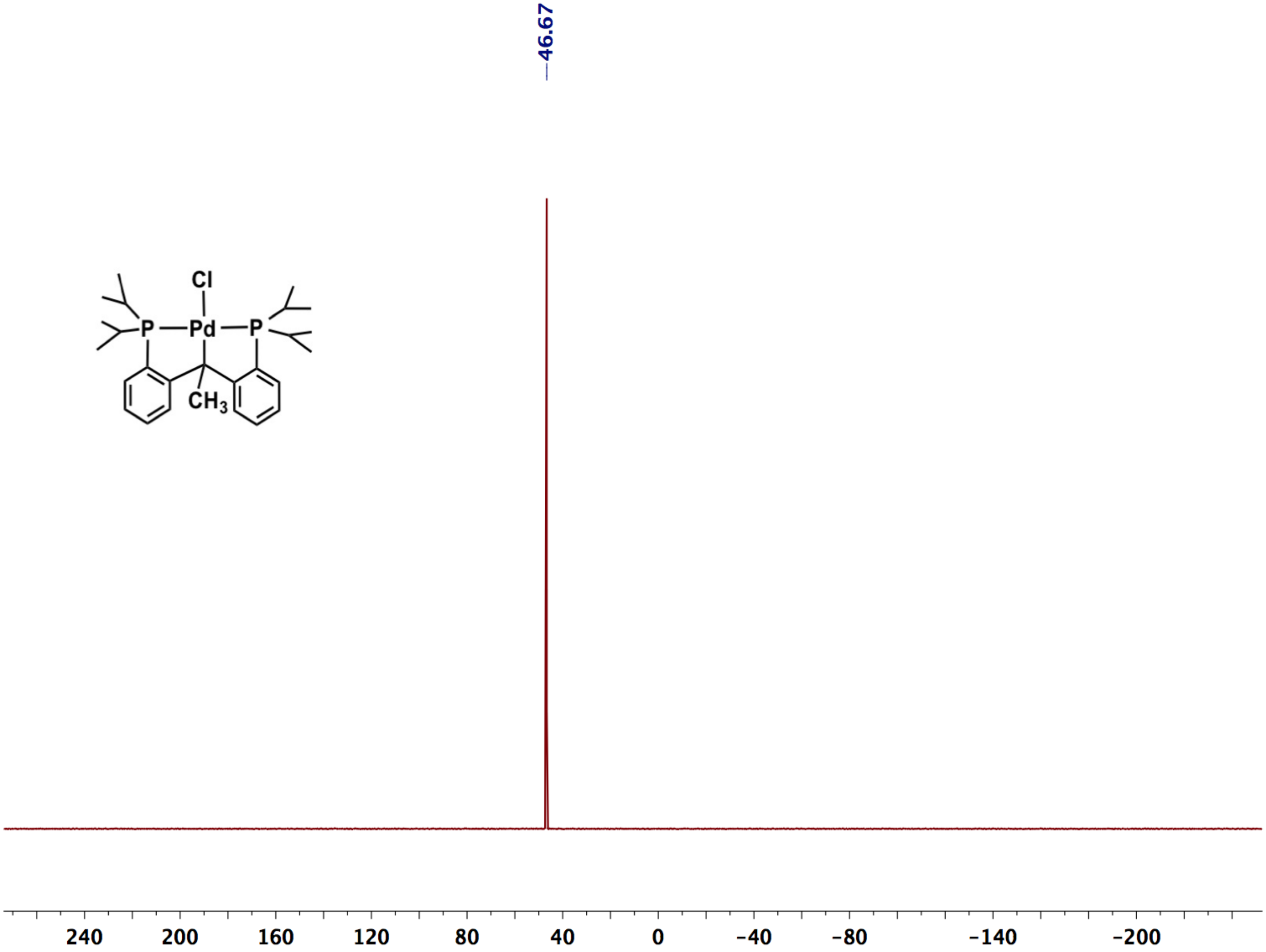

Figure S55. $^{31}P\{^1H\}$ NMR spectrum for $[PC(CH_3)P]PdCl$ (**9**).

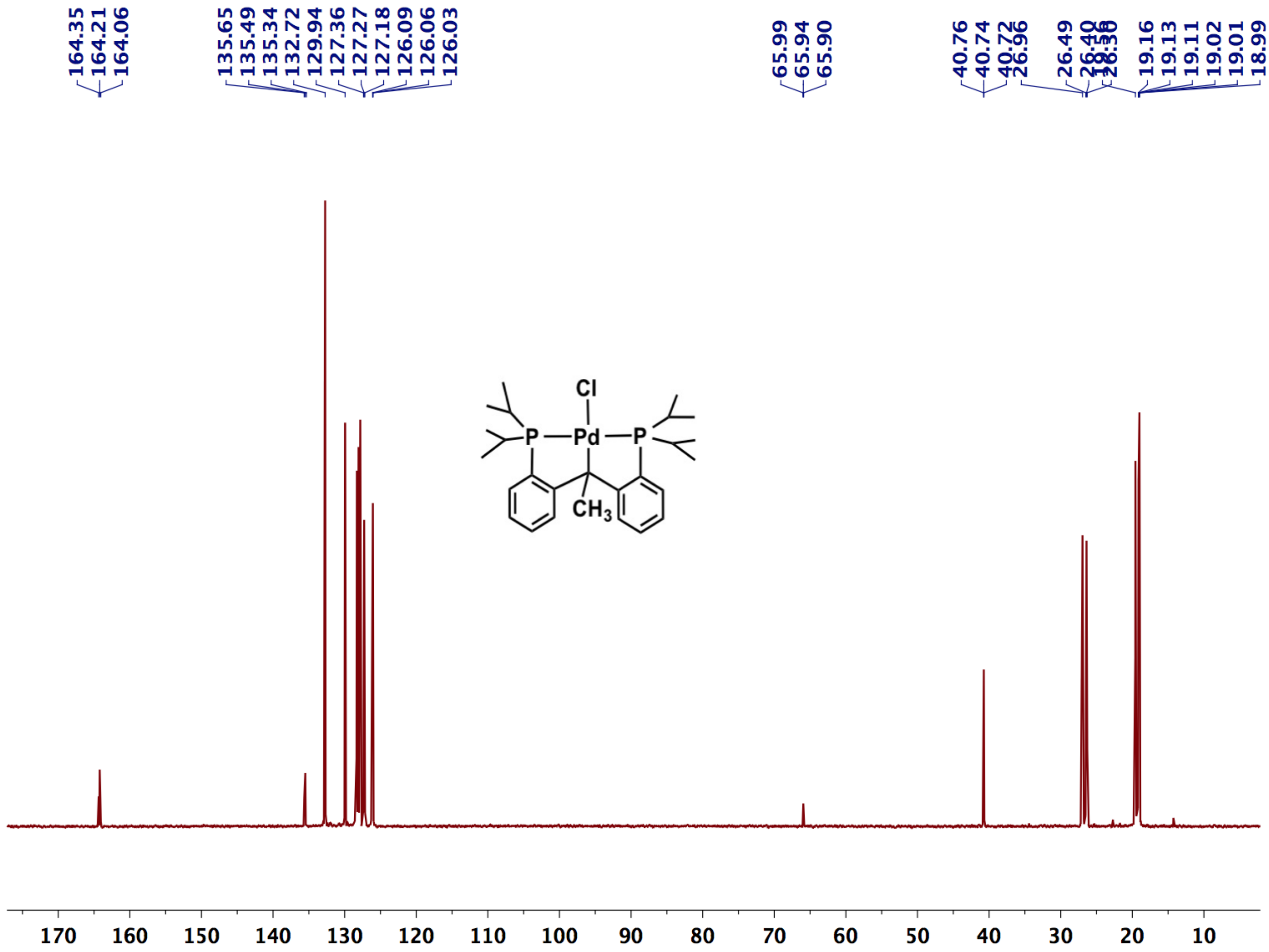

Figure S56. $^{13}C\{^{1}H\}$ NMR spectrum for $[PC(CH_3)P]PdCl$ (**9**).

7.9 NMR Spectra for [PC(sp^3)HP]PdI (11)

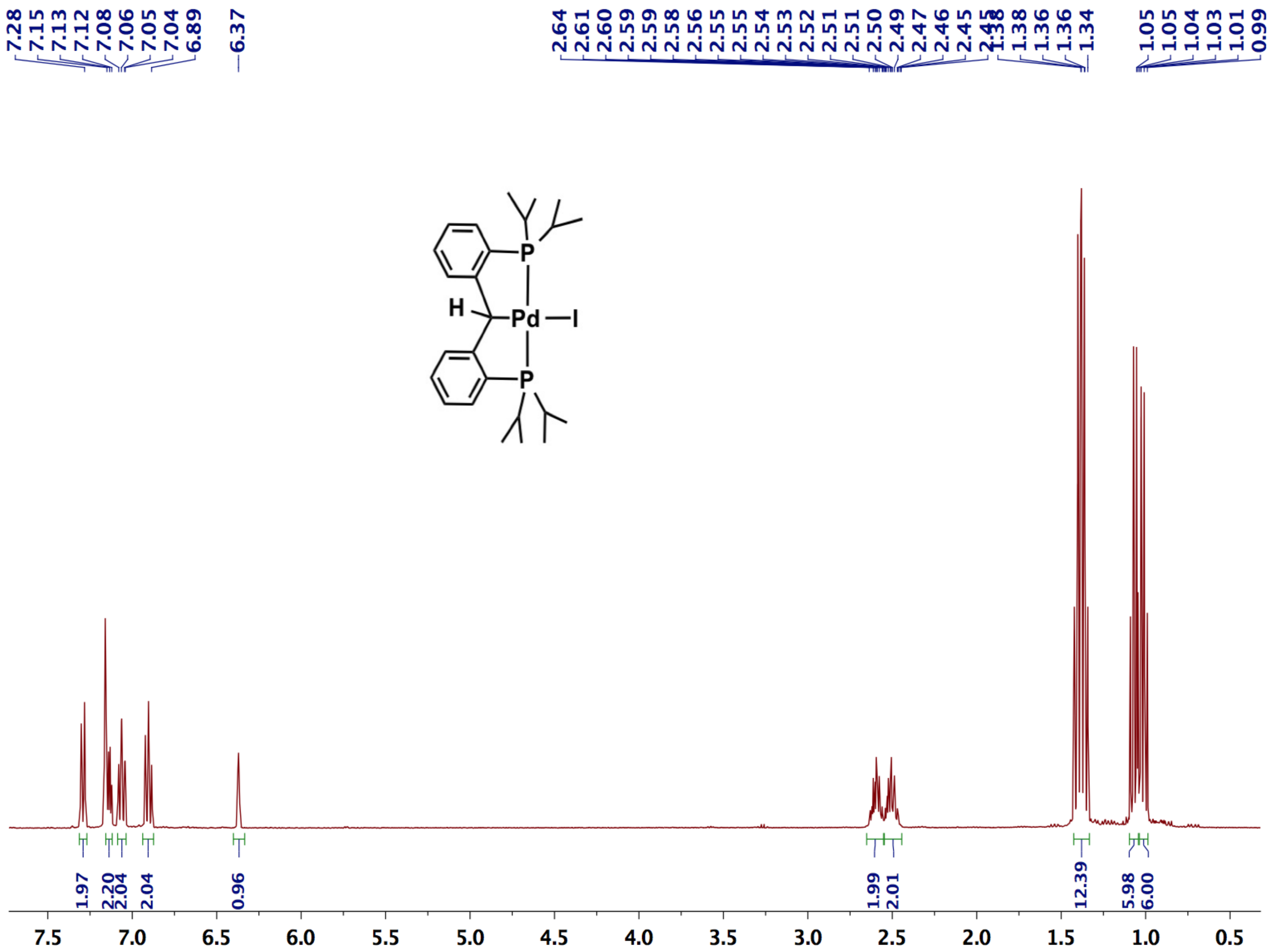

Figure S57. ^{1}H NMR spectrum for [PC(sp^3)HP]PdI (**11**).

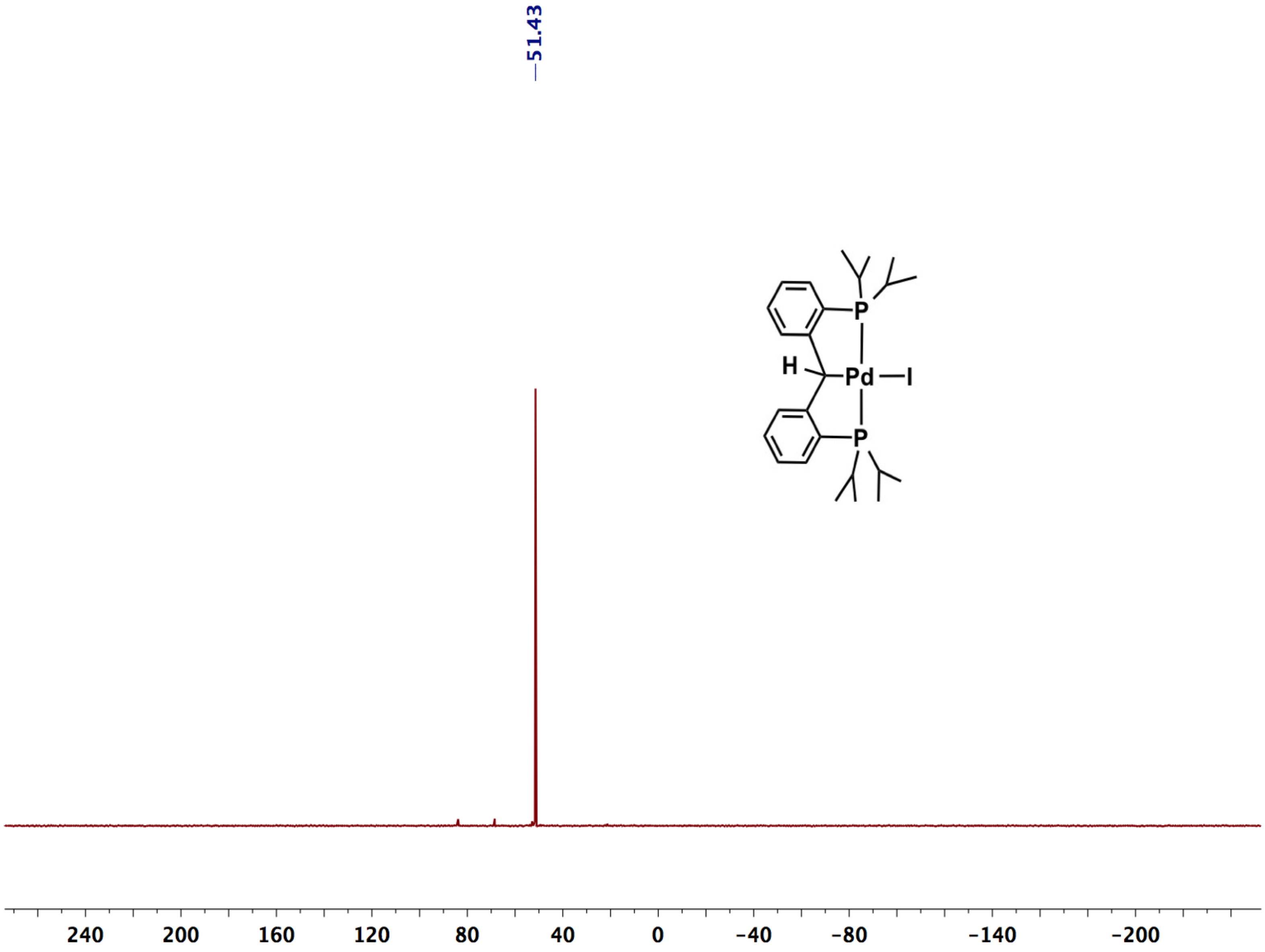

Figure S58. $^{31}P\{^{1}H\}$ NMR spectrum for [PC(sp^3)HP]PdI (**11**).

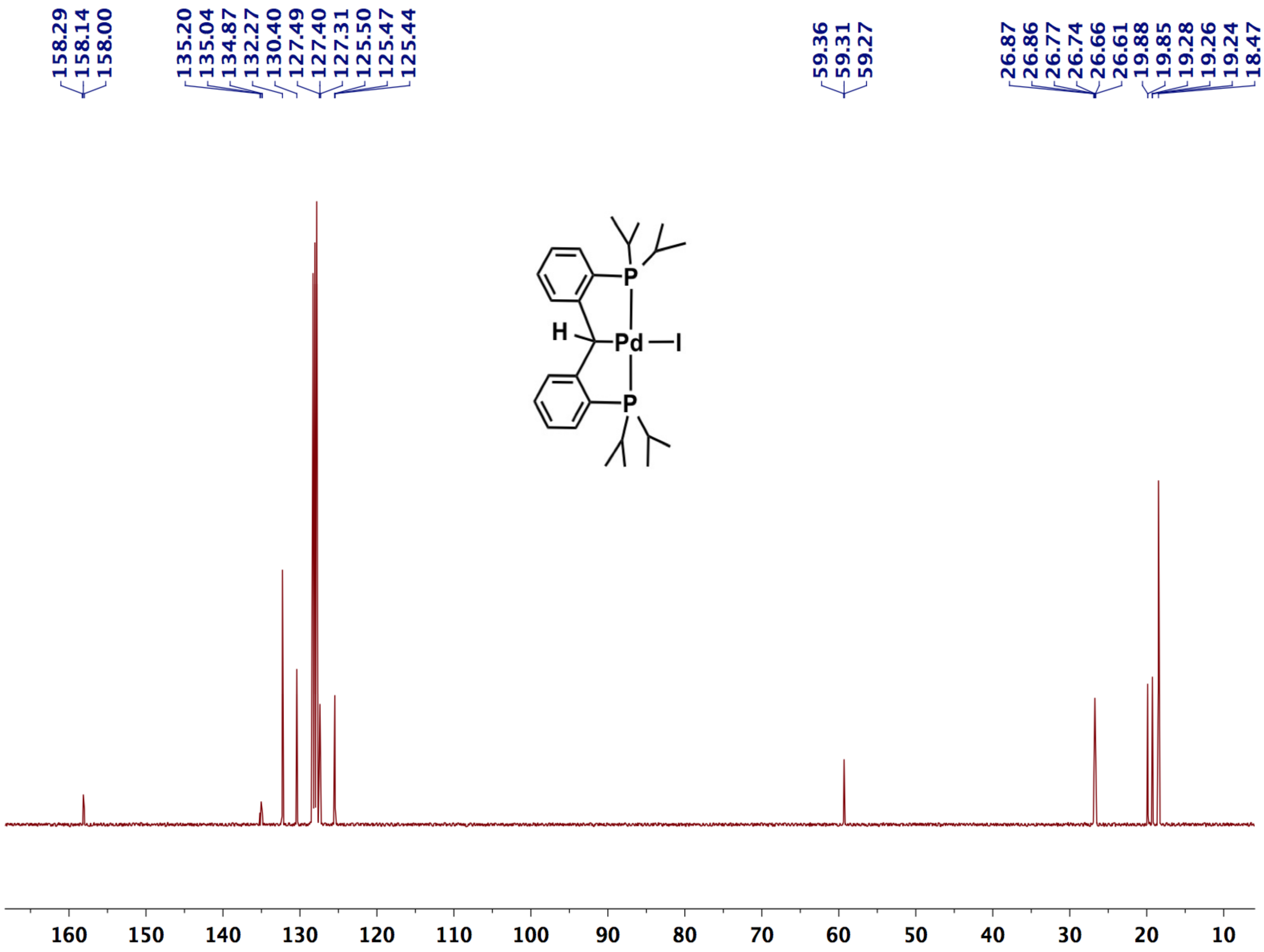

Figure S59. $^{13}C\{^1H\}$ NMR spectrum for [PC(sp^3)HP]PdI (**11**).

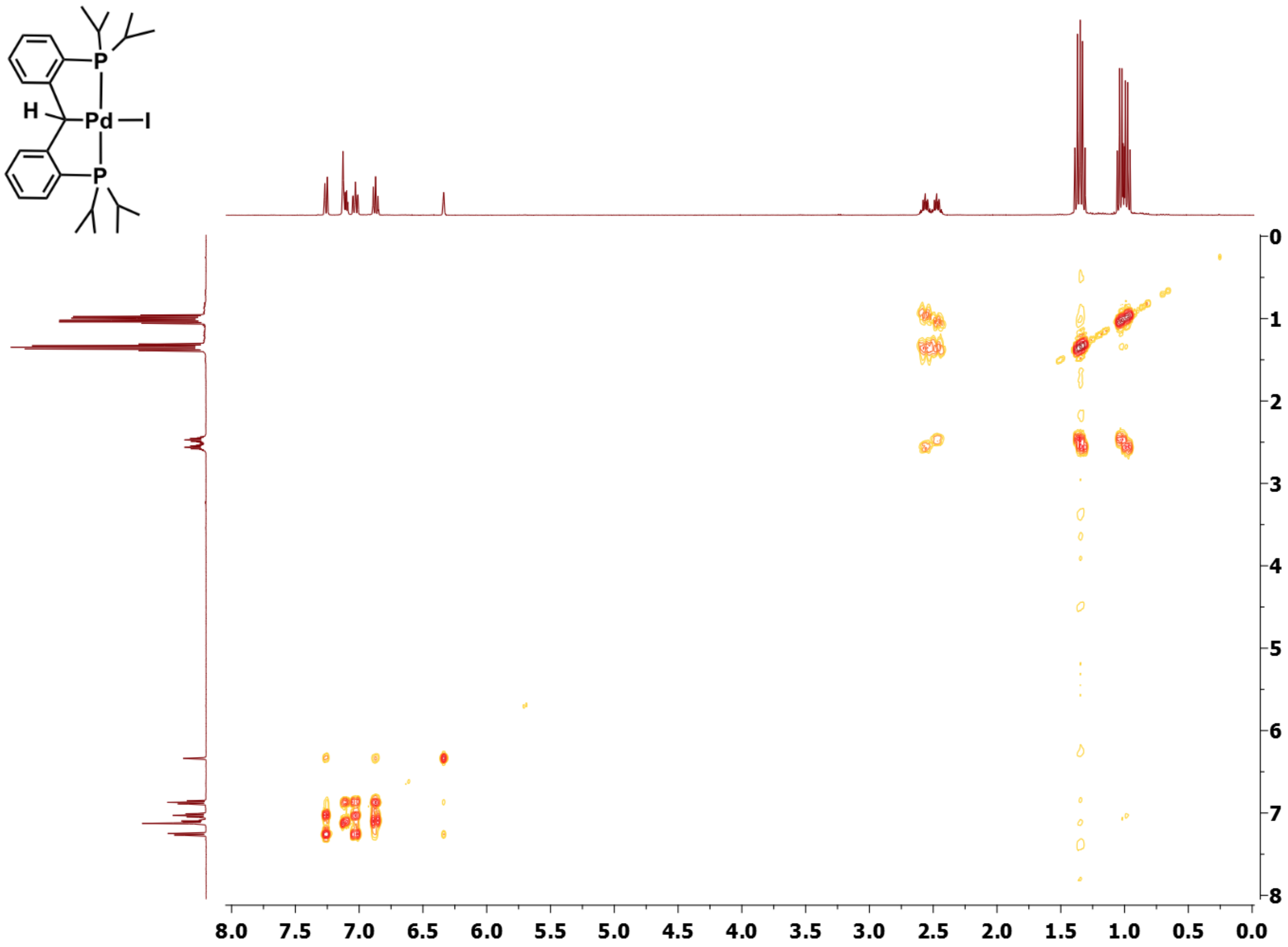

Figure S60. ^{1}H-^{1}H COSY NMR spectrum for [PC(sp^3)HP]PdI (**11**).

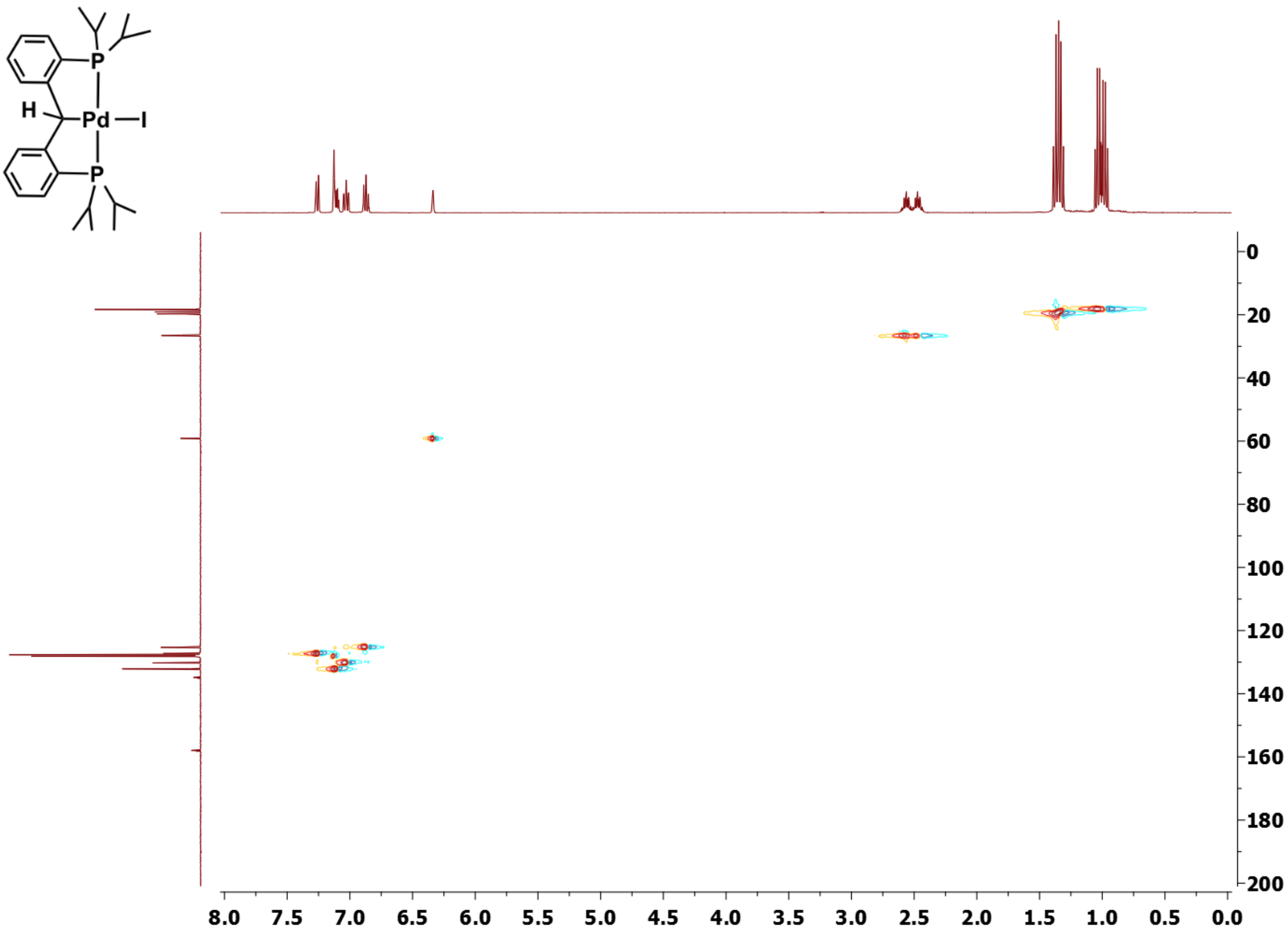

Figure S61. ^{1}H-^{13}C HSQC NMR spectrum for [PC(sp^{3})HP]PdI (**11**).

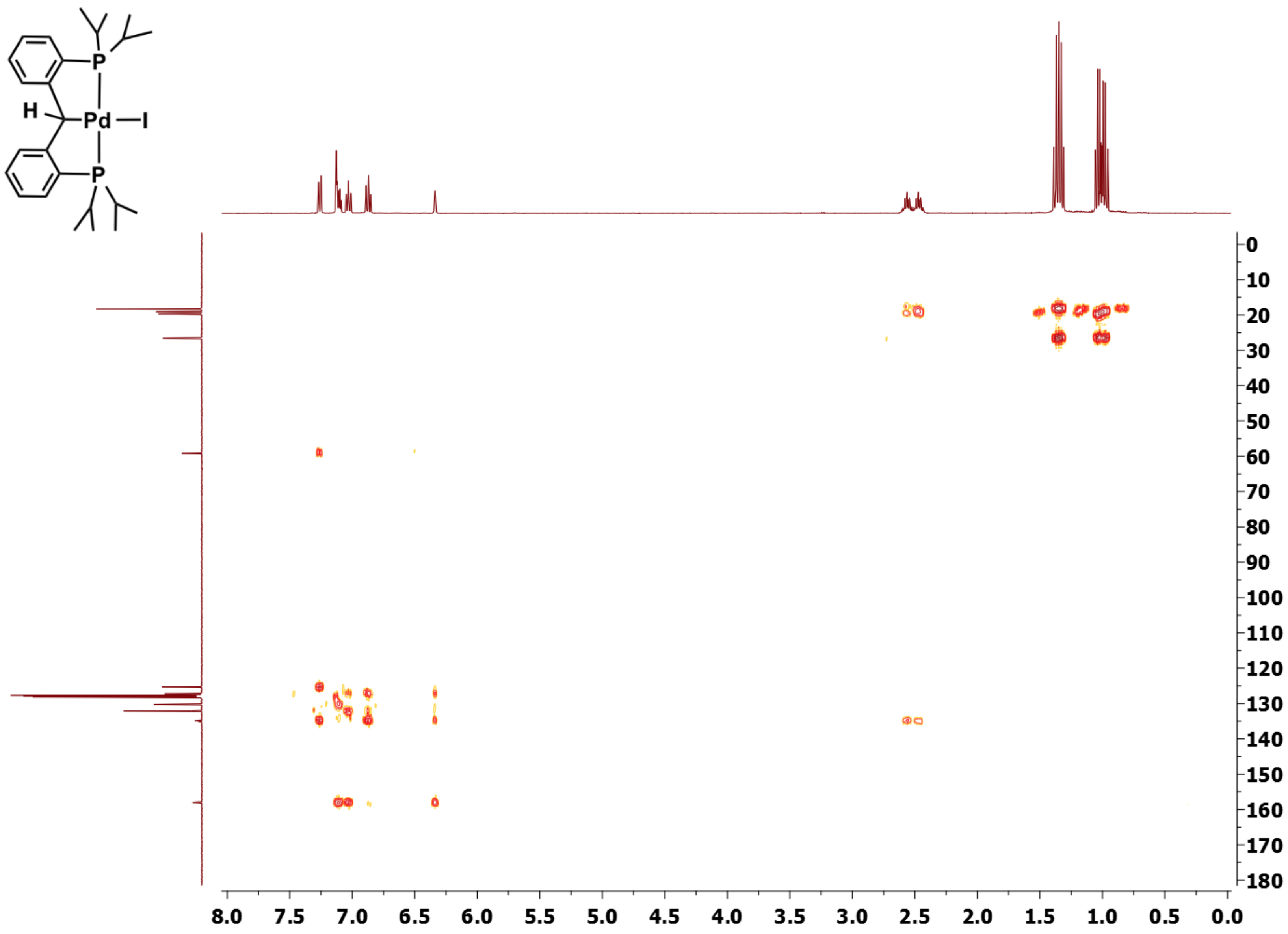

Figure S62. ^{1}H-^{13}C HMBC NMR spectrum for [PC(sp^{3})HP]PdI (**11**).

7.10 NMR Spectra for $[PC(sp^3)H_2P]PdBr_2$ (12)

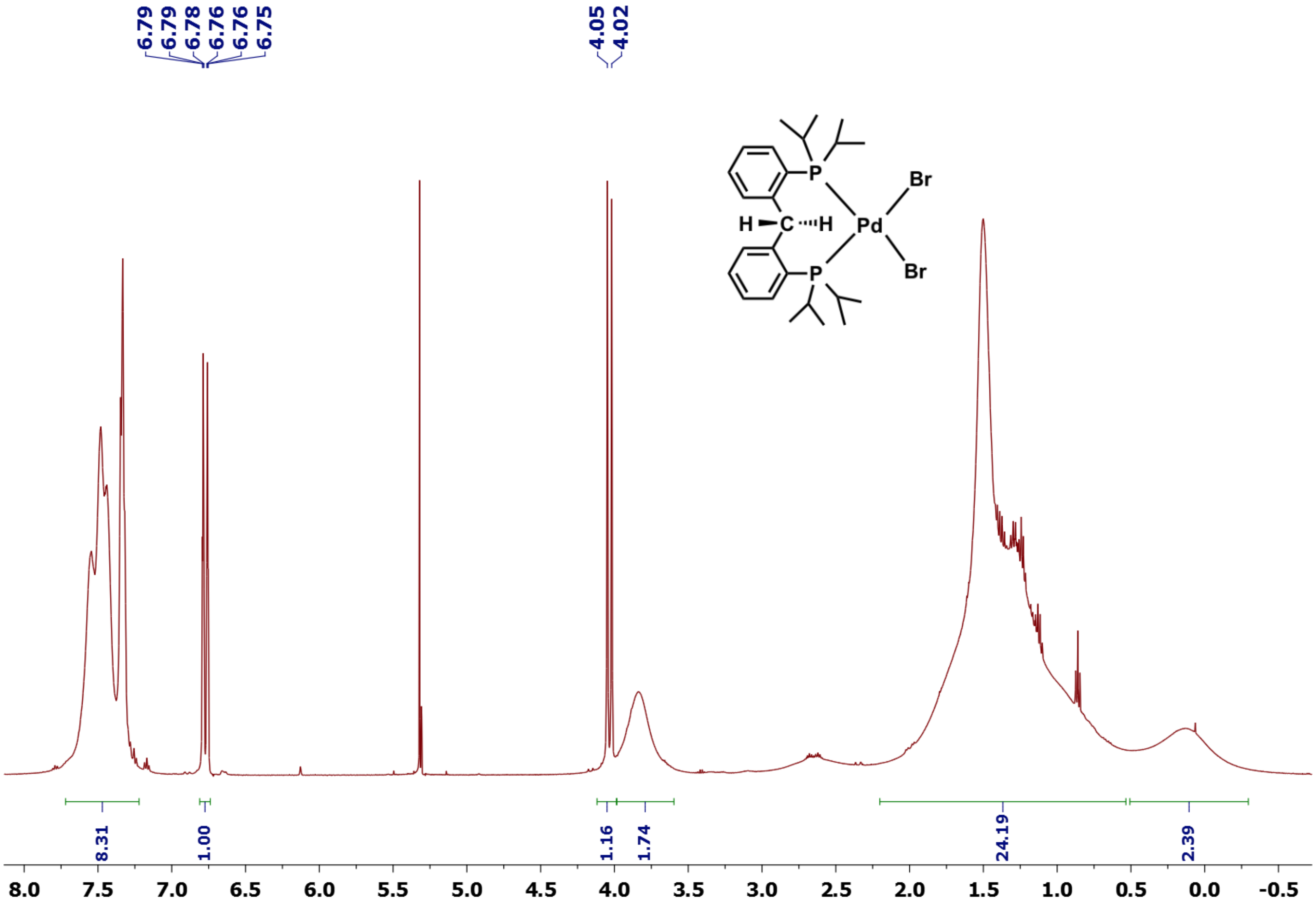

Figure S63. ^1H NMR spectrum (298 K) for $[PC(sp^3)H_2P]PdBr_2$ (**12**).

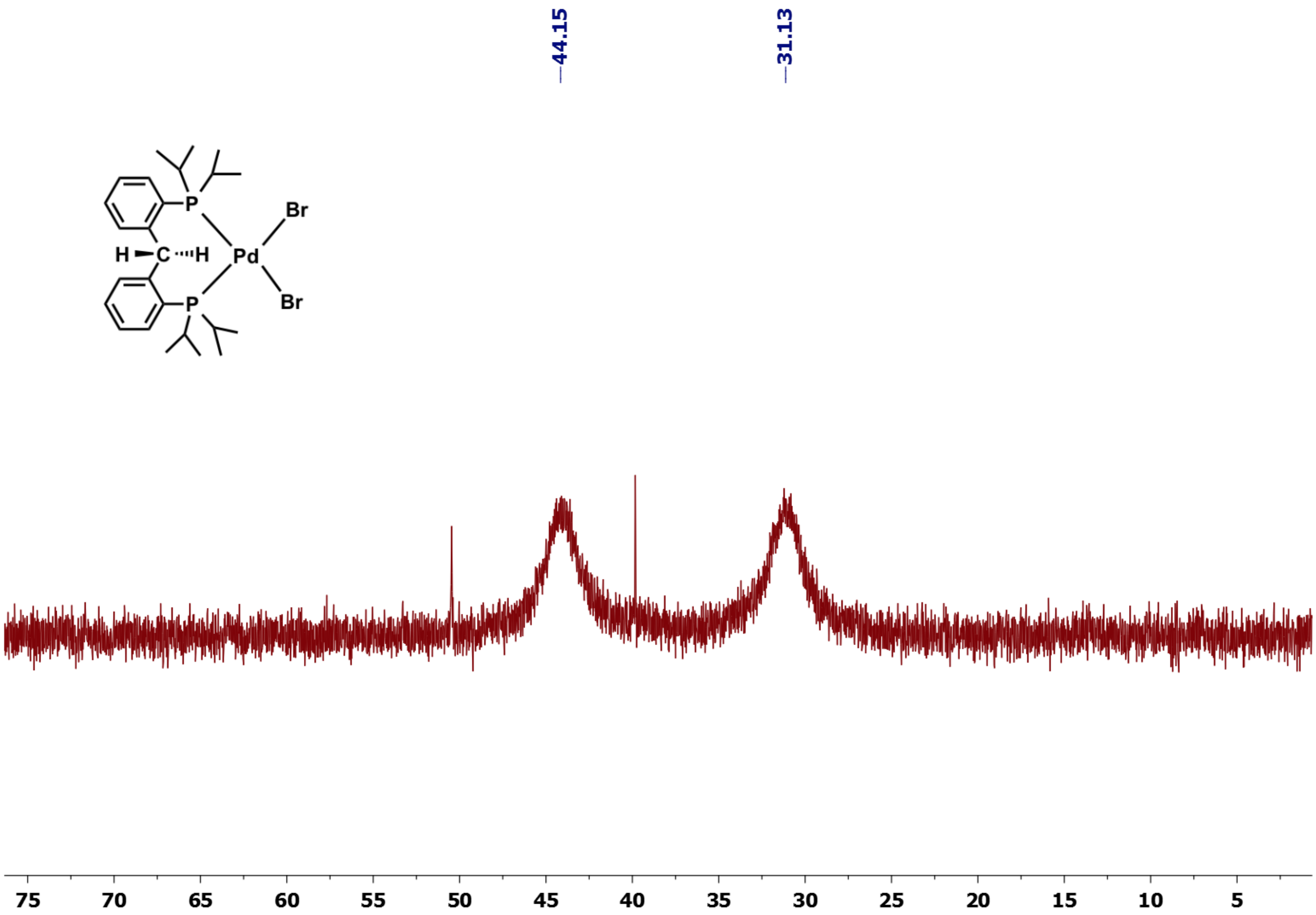

Figure S64. $^{31}P\{^{1}H\}$ NMR spectrum (298 K) for $[PC(sp^3)H_2P]PdBr_2$ (**12**).

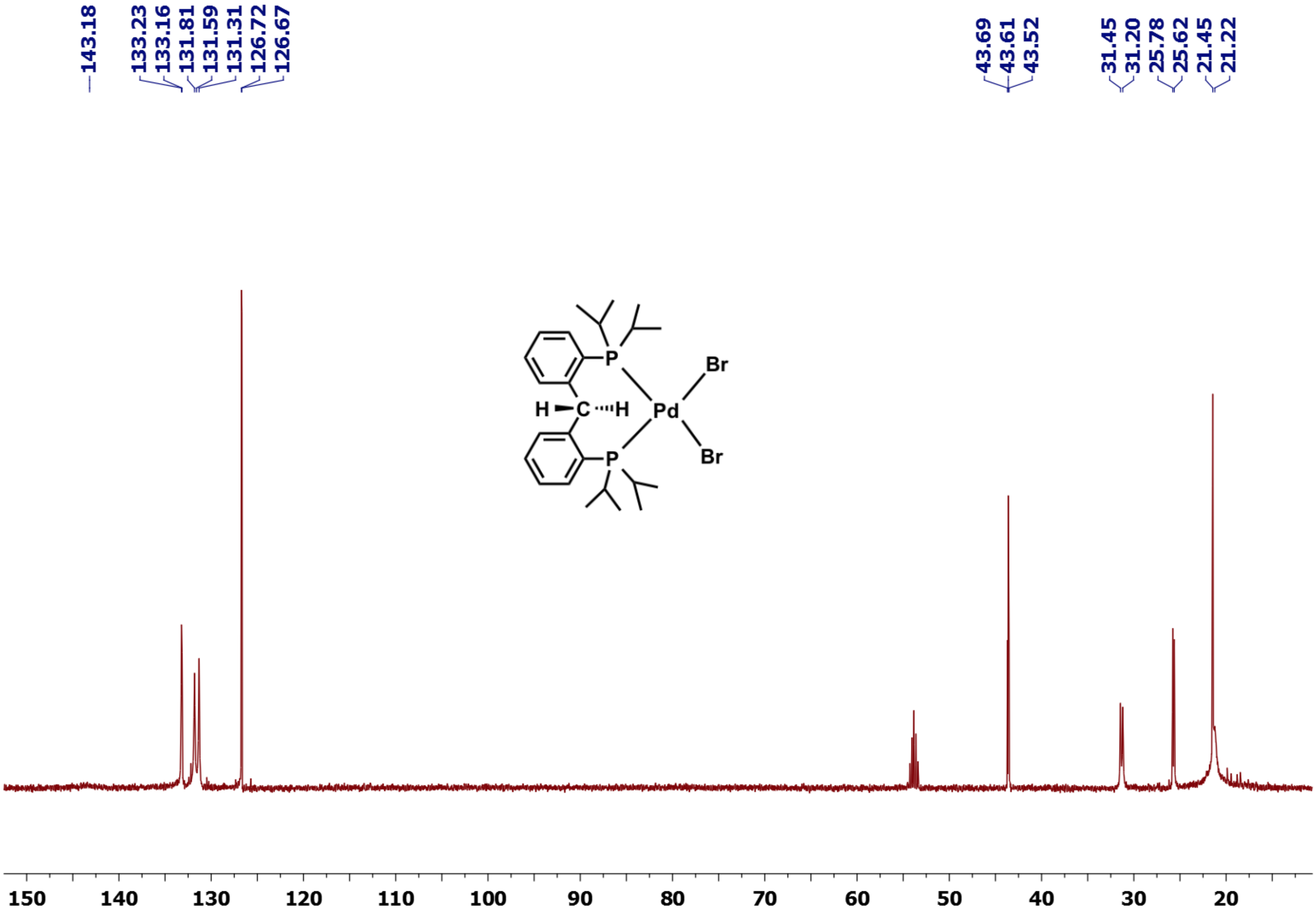

Figure S65. $^{13}C\{^{1}H\}$ NMR spectrum (298 K) for $[PC(sp^3)H_2P]PdBr_2$ (**12**).

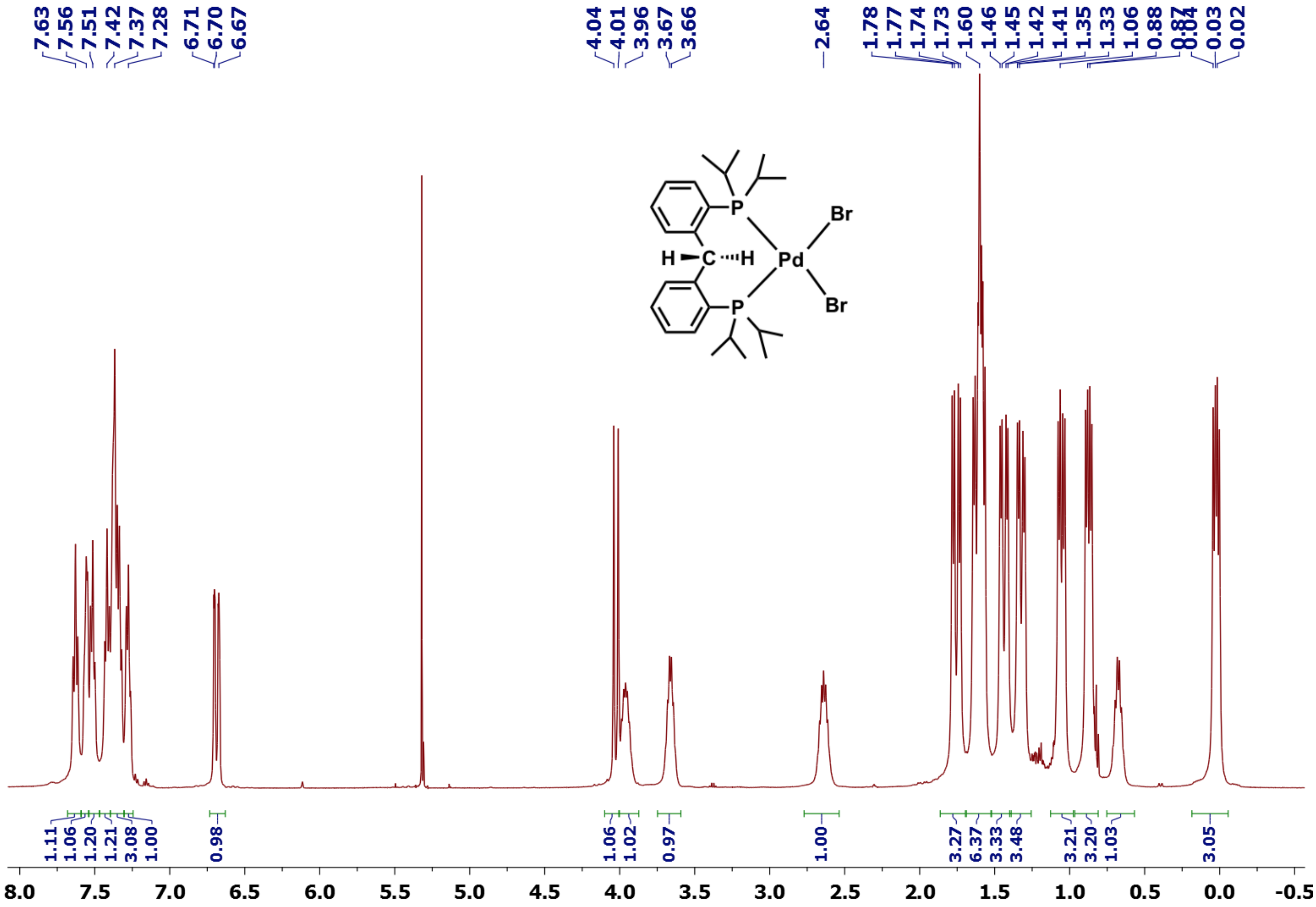

Figure S66. ^{1}H NMR spectrum (248 K) for [PC(sp^3)H$_2$P]PdBr$_2$ (**12**).

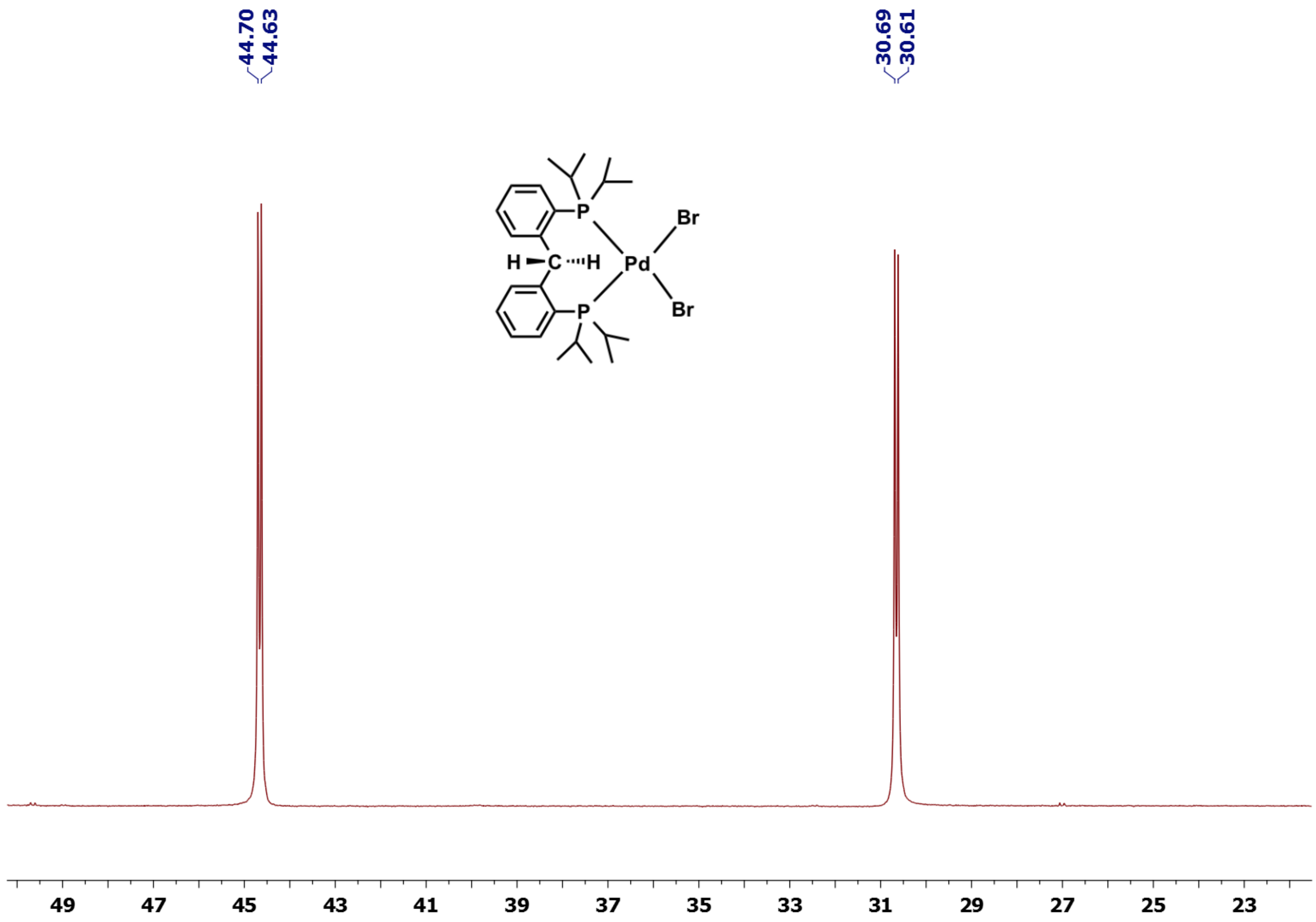

Figure S67. $^{31}P\{^{1}H\}$ NMR spectrum (248 K) for $[PC(sp^3)H_2P]PdBr_2$ (**12**).

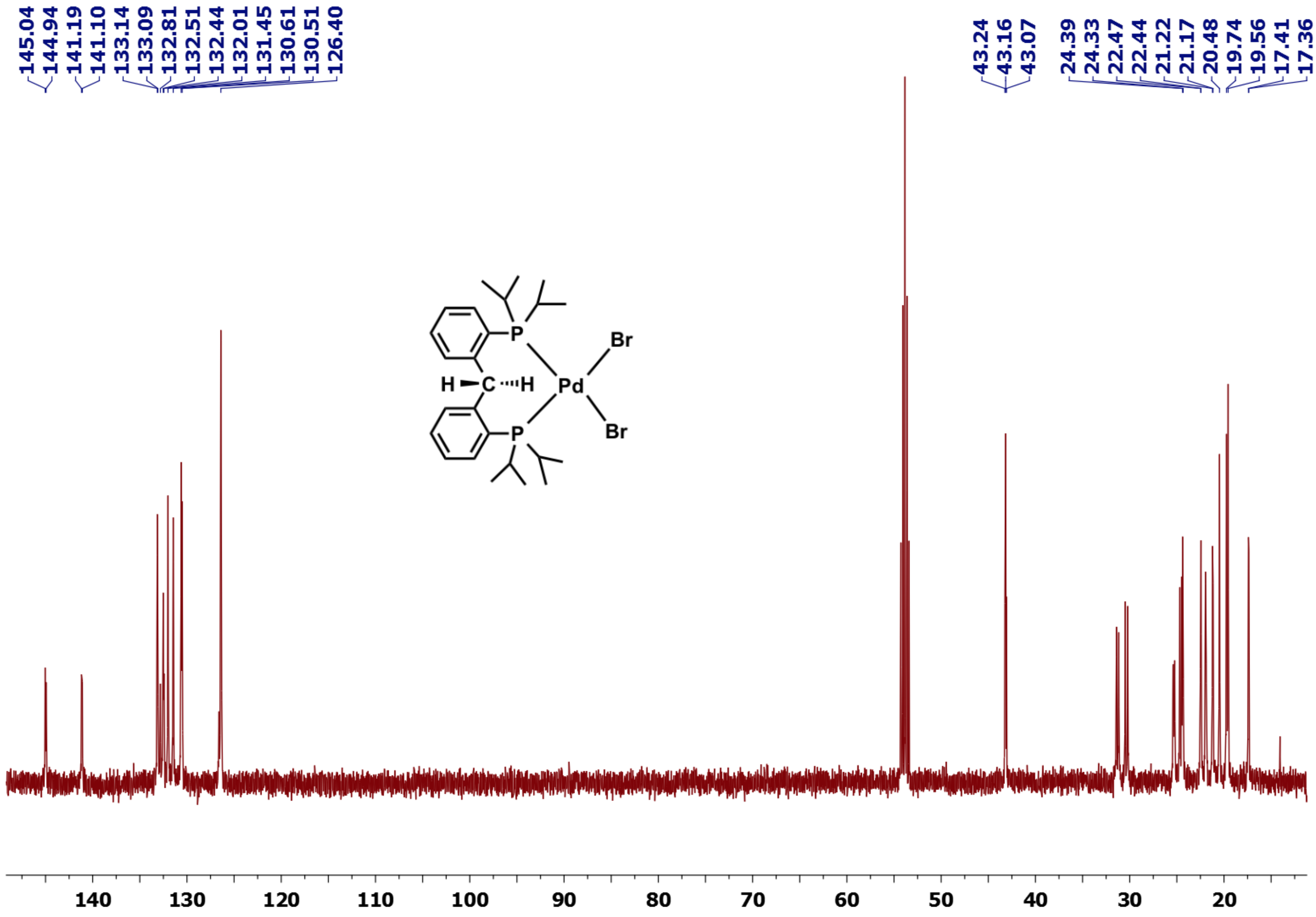

Figure S68. $^{13}C\{^1H\}$ NMR spectrum (248 K) for $[PC(sp^3)H_2P]PdBr_2$ (**12**).

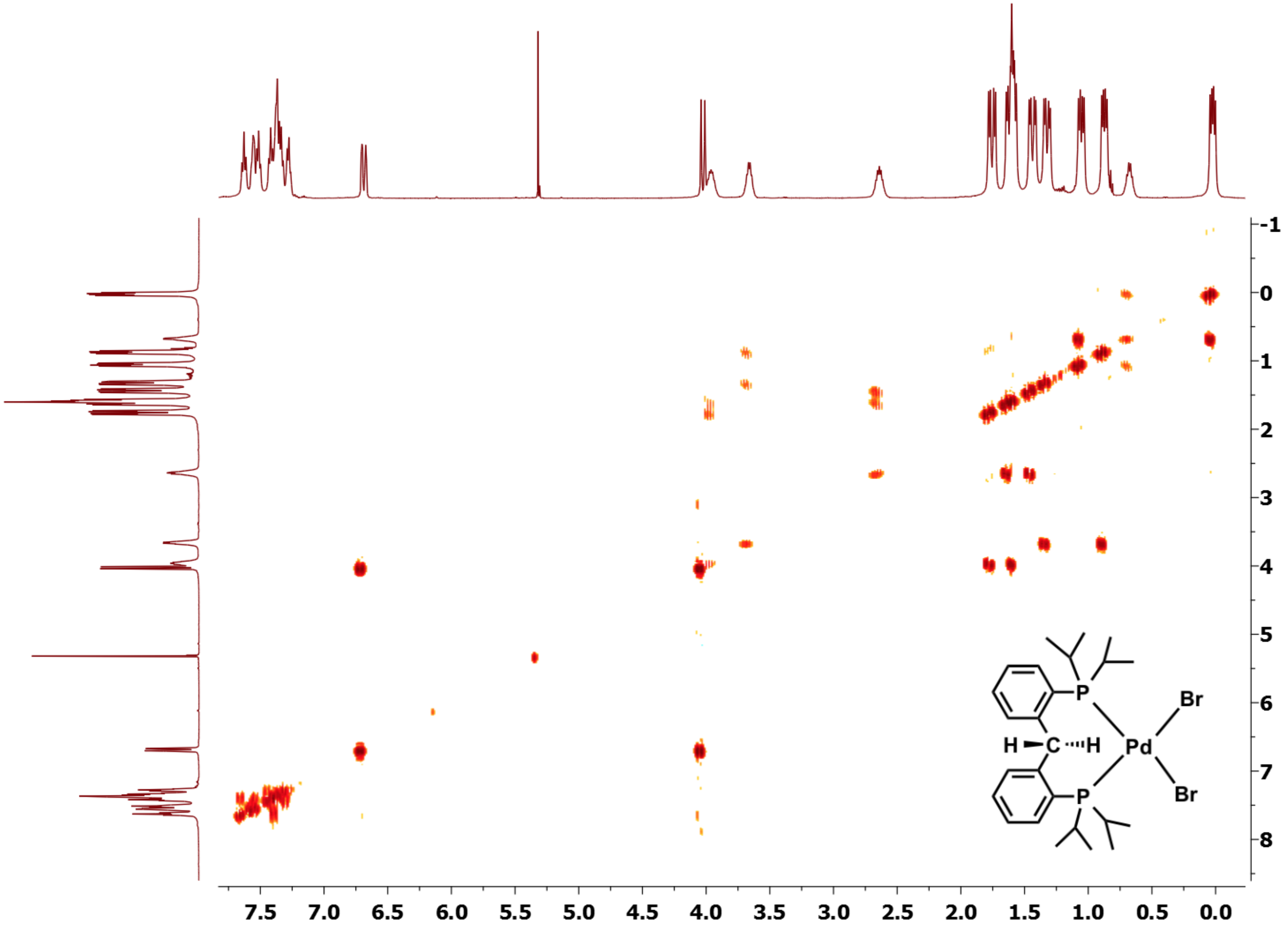

Figure S69. ^{1}H-^{1}H COSY NMR spectrum (248 K) for [PC(sp^3)H_2P]PdBr_2 (**12**).

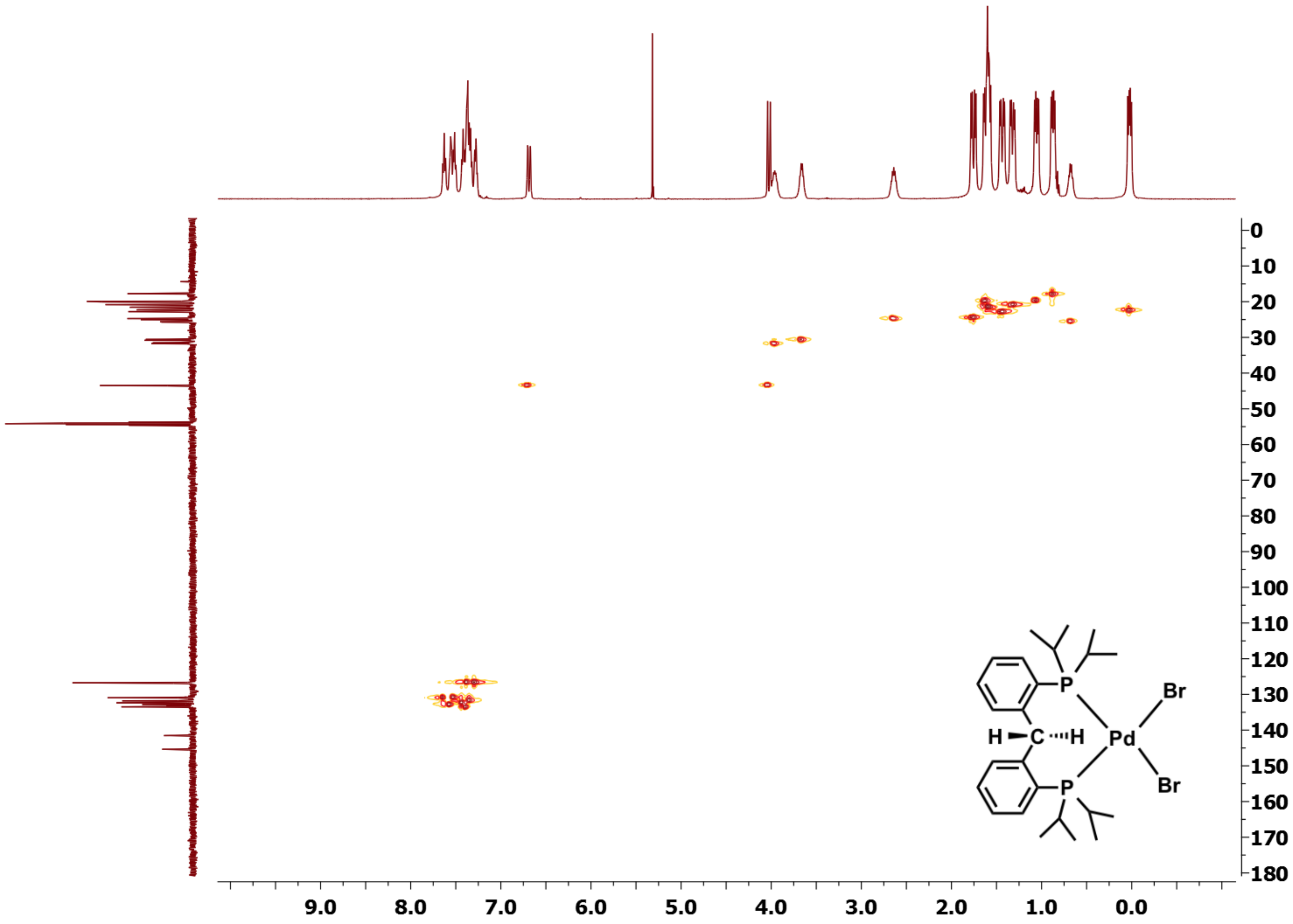

Figure S70. ^{1}H-^{13}C HSQC NMR spectrum (248 K) for [PC(sp^3)H$_2$P]PdBr$_2$ (**12**).

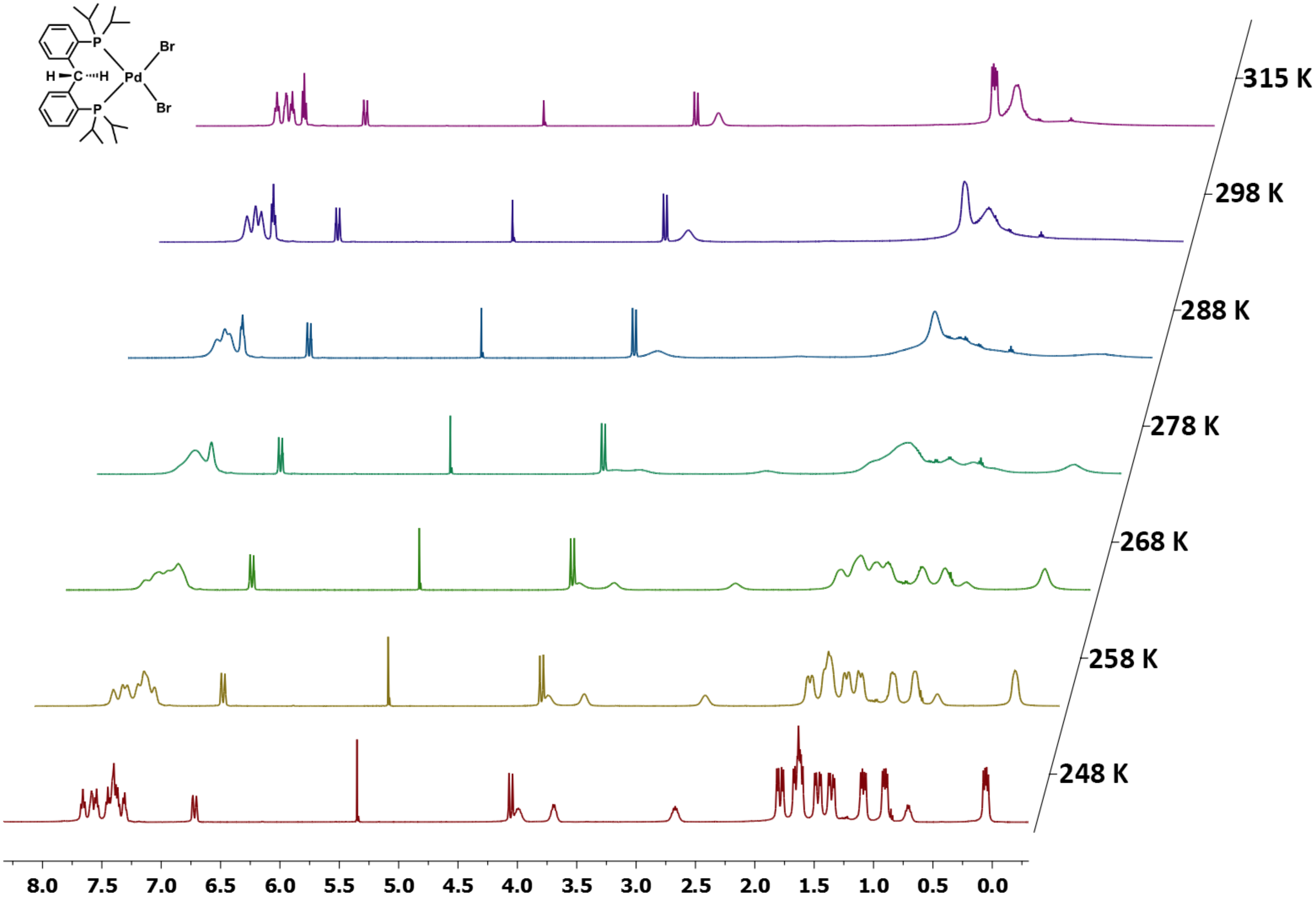

Figure S71. Variable temperature ^{1}H NMR spectra for [PC(sp^{3})H$_{2}$P]PdBr$_{2}$ (**12**).

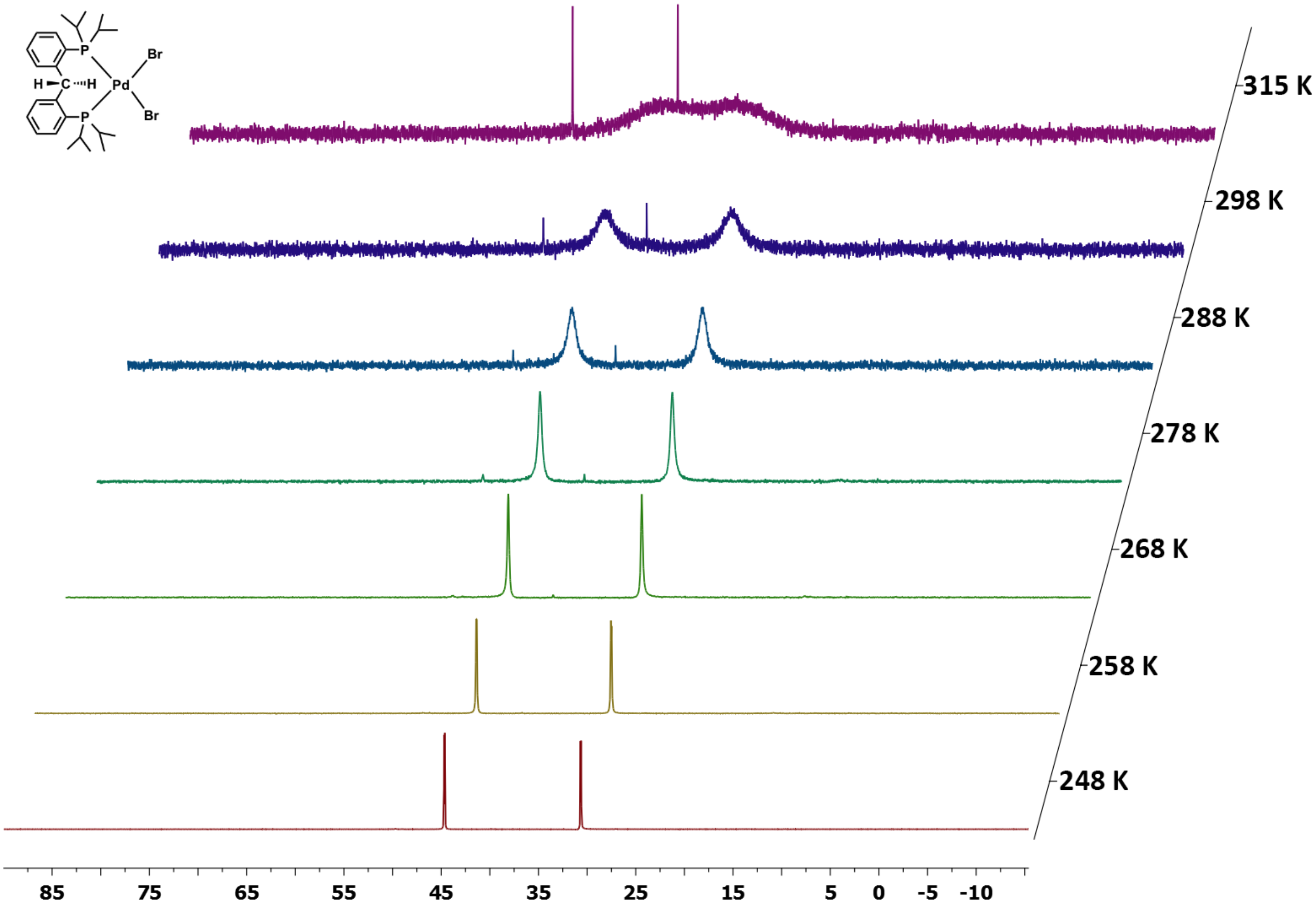

Figure S72. Variable temperature $^{31}P\{^{1}H\}$ NMR spectra for $[PC(sp^3)H_2P]PdBr_2$ (**12**).

8 Crystallographic tables

8.1 Crystal data for {[PC(sp^2)P]PdI}$_2$·½Et$_2$O ({2}$_2$·½Et$_2$O)

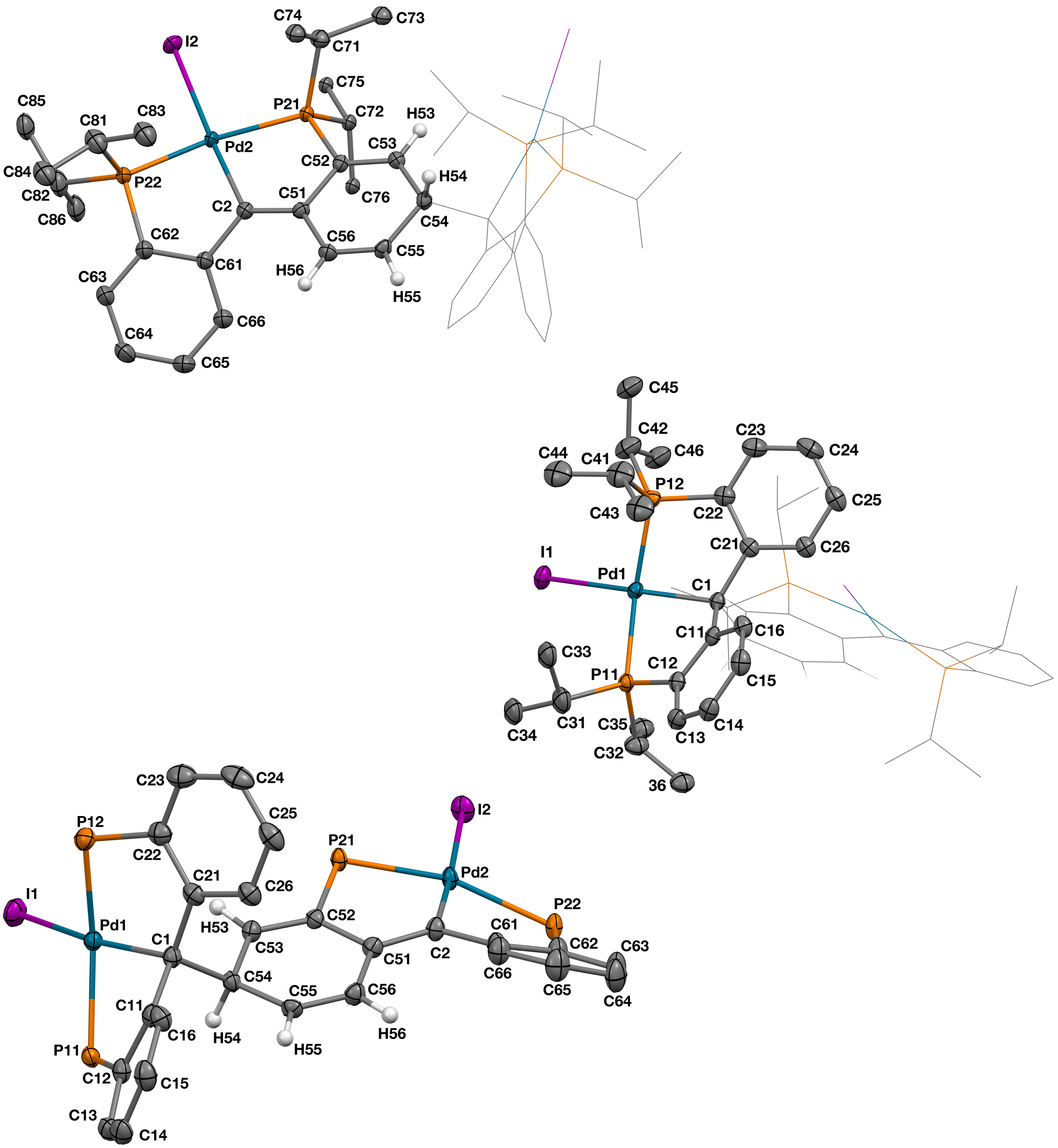

Figure S73. Thermal-ellipsoid representation of {[PC(sp^2)P]PdI}$_2$·½Et$_2$O (**{2}$_2$·½Et$_2$O**) at 50% probability. Most hydrogen atoms and the solvent were omitted for clarity.

Table S16. Crystal data and structure refinement for {[PC(sp^2)P]PdI}$_2$·½ Et$_2$O (**{2}$_2$·½ Et$_2$O**).

Identification code:	cc218c	
Empirical formula:	$C_{104}H_{154}I_4OP_8Pd_4$	
Formula weight:	2601.23	
Temperature:	120(2) K	
Wavelength:	0.71073 Å	
Crystal system:	Monoclinic	
Space group:	$P2_1/c$	
Unit cell dimensions:	*a* = 16.2343(6) Å	α = 90°
	b = 15.9423(6) Å	β = 94.7040(14)°
	c = 20.8753(8) Å	γ = 90°
Volume:	5384.6(4) Å^3	
Z:	2	
Density (calculated):	1.604 g·cm^{-3}	
Absorption coefficient (μ):	1.967 mm^{-1}	
F(000):	2604	
Crystal size:	0.11 × 0.09 × 0.08 mm^3	
θ range for data collection:	1.26 to 25.00°	
Index ranges:	$-19 \le h \le 19$, $-18 \le k \le 18$, $-24 \le l \le 24$	
Reflections collected:	131164	
Independent reflections:	9479 [R_{int} = 0.0363]	
Completeness to θ = 25.00°:	100.0 %	
Absorption correction:	Semi-empirical from equivalents	
Max. and min. transmission:	0.7457 and 0.6774	
Refinement method:	Full-matrix least-squares on F^2	
Data / restraints / parameters:	9479 / 0 / 477	
Goodness-of-fit on F^2:	1.113	
Final R indices [I>2σ(I)]:	R_1 = 0.0257, wR_2 = 0.0662	
R indices (all data):	R_1 = 0.0314, wR_2 = 0.0733	
Largest diff. peak and hole:	0.964 and −1.540 e$^-$·Å^{-3}	

Table S17. Atomic coordinates and equivalent isotropic displacement parameters (Å^2) for {[PC(sp^2)P]PdI}$_2$·½ Et$_2$O (**{2}$_2$·½ Et$_2$O**). U(eq) is defined as one third of the trace of the orthogonalized U_{ij} tensor

atom	x	y	z	U(eq)
C(1)	0.33153(19)	0.1629(2)	0.26023(15)	0.018(1)
I(1)	0.12423(1)	0.16406(2)	0.08361(1)	0.029(1)
Pd(1)	0.22869(2)	0.16102(2)	0.19020(1)	0.017(1)
C(2)	0.65139(19)	0.1826(2)	0.16751(15)	0.019(1)
I(2)	0.66719(1)	0.27749(1)	−0.04575(1)	0.024(1)
Pd(2)	0.66143(1)	0.22217(2)	0.07538(1)	0.016(1)
P(11)	0.23227(5)	0.01697(5)	0.21072(4)	0.019(1)
O(9)	1.0000	0.5000	0.0000	0.066(1)
C(11)	0.31086(19)	0.1016(2)	0.31291(15)	0.019(1)
P(12)	0.21527(5)	0.29868(6)	0.21480(4)	0.022(1)
C(12)	0.27613(19)	0.0236(2)	0.29410(15)	0.021(1)
C(13)	0.2660(2)	−0.0388(2)	0.33990(16)	0.025(1)
C(14)	0.2852(2)	−0.0230(2)	0.40492(17)	0.027(1)
C(15)	0.3137(2)	0.0553(2)	0.42348(17)	0.028(1)
C(16)	0.3272(2)	0.1167(2)	0.37838(16)	0.024(1)
P(21)	0.52317(5)	0.24298(5)	0.07745(4)	0.016(1)
C(21)	0.3566(2)	0.2511(2)	0.28317(15)	0.020(1)
P(22)	0.79370(5)	0.16841(6)	0.09114(4)	0.019(1)
C(22)	0.3109(2)	0.3221(2)	0.26250(16)	0.023(1)
C(26)	0.4284(2)	0.2652(2)	0.32375(15)	0.022(1)
C(25)	0.4539(2)	0.3450(2)	0.34195(16)	0.028(1)
C(24)	0.4079(3)	0.4141(2)	0.32028(18)	0.033(1)
C(23)	0.3364(2)	0.4023(2)	0.28094(17)	0.031(1)
C(31)	0.1304(2)	−0.0329(3)	0.2190(2)	0.037(1)
C(32)	0.2875(2)	−0.0652(2)	0.16639(18)	0.031(1)
C(33)	0.0769(2)	0.0241(3)	0.2566(2)	0.037(1)
C(94)	0.9289(18)	0.5887(19)	0.0616(15)	0.108(10)
C(95)	1.0890(15)	0.3932(19)	−0.0434(16)	0.065(6)
C(93)	0.9310(7)	0.5430(7)	0.0062(6)	0.069(4)
C(92)	1.0090(7)	0.4463(7)	−0.0482(5)	0.049(4)
C(34)	0.0857(2)	−0.0566(3)	0.1540(2)	0.037(1)
C(35)	0.3185(2)	−0.0296(2)	0.10504(17)	0.031(1)
C(36)	0.3536(2)	−0.1114(2)	0.20680(18)	0.031(1)
C(43)	0.1484(3)	0.2641(3)	0.3310(2)	0.042(1)
C(42)	0.1923(3)	0.3773(3)	0.1509(2)	0.038(1)
C(41)	0.1338(3)	0.3170(3)	0.2707(2)	0.042(1)
C(44)	0.0489(3)	0.3012(3)	0.2365(2)	0.042(1)
C(46)	0.2626(2)	0.3799(3)	0.1068(2)	0.038(1)
C(45)	0.1684(3)	0.4648(2)	0.1697(2)	0.038(1)

Continued on next page

Table S17. – continued from previous page

atom	x	y	x	U(eq)
C(54)	0.40429(19)	0.1232(2)	0.22377(15)	0.017(1)
C(55)	0.48277(19)	0.1020(2)	0.26418(15)	0.018(1)
C(56)	0.5591(2)	0.1184(2)	0.24773(15)	0.020(1)
C(61)	0.7302(2)	0.1756(2)	0.20820(15)	0.021(1)
C(62)	0.8043(2)	0.1615(2)	0.17839(16)	0.023(1)
C(63)	0.8796(2)	0.1527(2)	0.21527(17)	0.028(1)
C(64)	0.8836(2)	0.1621(3)	0.28144(18)	0.033(1)
C(65)	0.8124(2)	0.1815(3)	0.31108(17)	0.030(1)
C(66)	0.7373(2)	0.1873(2)	0.27527(16)	0.024(1)
C(71)	0.4632(2)	0.1953(2)	0.00745(16)	0.022(1)
C(72)	0.47774(19)	0.3463(2)	0.08870(14)	0.016(1)
C(73)	0.3694(2)	0.2063(2)	0.00217(16)	0.022(1)
C(74)	0.4863(2)	0.1024(2)	0.00464(16)	0.022(1)
C(75)	0.49276(18)	0.4059(2)	0.03365(14)	0.016(1)
C(76)	0.50863(18)	0.3843(2)	0.15335(14)	0.016(1)
C(81)	0.8056(2)	0.0622(2)	0.0581(2)	0.033(1)
C(82)	0.8875(2)	0.2260(2)	0.07079(19)	0.032(1)
C(83)	0.7333(2)	0.0070(2)	0.07329(19)	0.033(1)
C(84)	0.8881(2)	0.0195(2)	0.08015(19)	0.033(1)
C(85)	0.9016(2)	0.2189(2)	−0.00022(19)	0.032(1)
C(86)	0.8834(2)	0.3164(2)	0.09231(19)	0.032(1)
C(53)	0.42313(19)	0.17205(19)	0.16555(15)	0.017(1)
C(52)	0.50027(19)	0.18573(19)	0.14881(14)	0.016(1)
C(51)	0.5754(2)	0.16236(19)	0.18845(15)	0.017(1)
H(13)	0.2459	−0.0925	0.3265	0.030
H(14)	0.2786	−0.0655	0.4360	0.032
H(15)	0.3244	0.0676	0.4679	0.034
H(16)	0.3479	0.1699	0.3924	0.029
H(26)	0.4605	0.2184	0.3392	0.027
H(25)	0.5030	0.3526	0.3693	0.033
H(24)	0.4254	0.4691	0.3324	0.040
H(23)	0.3044	0.4495	0.2663	0.037
H(31)	0.1403	−0.0857	0.2443	0.044
H(32)	0.2449	−0.1080	0.1523	0.037
H(33A)	0.0654	0.0761	0.2325	0.055
H(33B)	0.1060	0.0373	0.2984	0.055
H(33C)	0.0247	−0.0043	0.2632	0.055
H(94A)	0.9791	0.6229	0.0679	0.162
H(94B)	0.9260	0.5506	0.0981	0.162
H(94C)	0.8803	0.6253	0.0583	0.162
H(95A)	1.0917	0.3604	−0.0829	0.078
H(95B)	1.0887	0.3551	−0.0065	0.078

Continued on next page

Table S17. – continued from previous page

atom	x	y	x	U(eq)
H(95C)	1.1371	0.4303	−0.0377	0.078
H(93A)	0.8841	0.5031	0.0037	0.083
H(93B)	0.9222	0.5817	−0.0308	0.083
H(92A)	1.0068	0.4787	−0.0888	0.058
H(92B)	0.9612	0.4076	−0.0512	0.058
H(34A)	0.0785	−0.0064	0.1270	0.055
H(34B)	0.0314	−0.0804	0.1608	0.055
H(34C)	0.1185	−0.0981	0.1326	0.055
H(35A)	0.3343	−0.0758	0.0776	0.047
H(35B)	0.3666	0.0063	0.1160	0.047
H(35C)	0.2746	0.0035	0.0821	0.047
H(36A)	0.3748	−0.1575	0.1817	0.047
H(36B)	0.3304	−0.1342	0.2450	0.047
H(36C)	0.3989	−0.0728	0.2200	0.047
H(43A)	0.1490	0.2046	0.3192	0.063
H(43B)	0.1041	0.2743	0.3592	0.063
H(43C)	0.2017	0.2792	0.3535	0.063
H(42)	0.1434	0.3549	0.1239	0.045
H(41)	0.1366	0.3773	0.2839	0.050
H(44A)	0.0387	0.3417	0.2014	0.063
H(44B)	0.0066	0.3076	0.2670	0.063
H(44C)	0.0467	0.2441	0.2189	0.063
H(46A)	0.2739	0.3230	0.0921	0.057
H(46B)	0.3122	0.4028	0.1303	0.057
H(46C)	0.2467	0.4156	0.0696	0.057
H(45A)	0.1502	0.4968	0.1310	0.057
H(45B)	0.2162	0.4927	0.1921	0.057
H(45C)	0.1233	0.4619	0.1981	0.057
H(54)	0.3823	0.0682	0.2069	0.021
H(55)	0.4777	0.0750	0.3042	0.021
H(56)	0.6049	0.1007	0.2758	0.024
H(63)	0.9283	0.1403	0.1949	0.034
H(64)	0.9348	0.1553	0.3064	0.039
H(65)	0.8154	0.1907	0.3562	0.036
H(66)	0.6891	0.1997	0.2964	0.029
H(71)	0.4834	0.2221	−0.0317	0.027
H(72)	0.4166	0.3386	0.0889	0.019
H(73A)	0.3559	0.2658	0.0068	0.034
H(73B)	0.3459	0.1740	0.0362	0.034
H(73C)	0.3463	0.1861	−0.0399	0.034
H(74A)	0.4559	0.0762	−0.0327	0.034
H(74B)	0.4719	0.0743	0.0440	0.034

Continued on next page

Table S17. – continued from previous page

**atom**	**x**	**y**	**x**	**U(eq)**
H(74C)	0.5458	0.0970	0.0008	0.034
H(75A)	0.4735	0.3797	−0.0073	0.024
H(75B)	0.5520	0.4179	0.0340	0.024
H(75C)	0.4625	0.4583	0.0389	0.024
H(76A)	0.5684	0.3938	0.1544	0.024
H(76B)	0.4968	0.3458	0.1880	0.024
H(76C)	0.4805	0.4379	0.1592	0.024
H(81)	0.8035	0.0678	0.0103	0.040
H(82)	0.9360	0.1996	0.0957	0.039
H(83A)	0.6814	0.0331	0.0562	0.050
H(83B)	0.7331	0.0006	0.1200	0.050
H(83C)	0.7388	−0.0482	0.0535	0.050
H(84A)	0.8918	−0.0345	0.0582	0.050
H(84B)	0.8911	0.0104	0.1267	0.050
H(84C)	0.9340	0.0555	0.0696	0.050
H(85A)	0.8529	0.2398	−0.0262	0.048
H(85B)	0.9110	0.1600	−0.0110	0.048
H(85C)	0.9500	0.2523	−0.0092	0.048
H(86A)	0.9344	0.3454	0.0834	0.048
H(86B)	0.8769	0.3184	0.1386	0.048
H(86C)	0.8361	0.3441	0.0689	0.048
H(53)	0.3782	0.1946	0.1390	0.021

Table S18. Anisotropic displacement parameters (Å^2) for {[PC(sp^2)P]PdI}$_2$·½Et$_2$O (**{2}$_2$·½ Et$_2$O**). The anisotropic displacement factor exponent takes the form: $-2\pi^2[h^2a^{*2}U_{11} + ...+ 2hka^*b^*U_{12}]$.

atom	U_{11}	U_{22}	U_{33}	U_{23}	U_{13}	U_{12}
C(1)	0.0152(16)	0.0200(16)	0.0180(16)	0.0006(13)	0.0029(13)	−0.0005(13)
I(1)	0.0218(1)	0.0364(1)	0.0275(1)	0.0036(1)	−0.0058(1)	0.0018(1)
Pd(1)	0.0141(1)	0.0197(1)	0.0181(1)	0.0013(1)	0.0018(1)	0.0004(1)
C(2)	0.0161(16)	0.0240(17)	0.0158(16)	−0.0003(13)	0.0018(12)	0.0007(13)
I(2)	0.0239(1)	0.0338(1)	0.0151(1)	0.0041(1)	0.0051(1)	−0.0015(1)
Pd(2)	0.0122(1)	0.0235(1)	0.0131(1)	0.0016(1)	0.0026(1)	−0.0009(1)
P(11)	0.0146(4)	0.0206(4)	0.0204(4)	0.0014(3)	0.0014(3)	−0.0028(3)
O(9)	0.045(3)	0.094(4)	0.061(3)	−0.017(3)	0.019(2)	−0.014(3)
C(11)	0.0131(15)	0.0244(17)	0.0200(16)	0.0013(13)	0.0036(12)	0.0007(13)
P(12)	0.0204(4)	0.0219(4)	0.0260(5)	0.0012(4)	0.0066(4)	0.0031(4)
C(12)	0.0154(16)	0.0264(18)	0.0205(16)	0.0001(14)	0.0043(13)	−0.0006(13)
C(13)	0.0209(17)	0.0263(18)	0.0273(18)	0.0063(15)	0.0030(14)	−0.0035(14)
C(14)	0.0223(18)	0.033(2)	0.0251(18)	0.0100(15)	0.0048(14)	−0.0020(15)
C(15)	0.0230(18)	0.042(2)	0.0201(17)	0.0019(16)	0.0044(14)	−0.0031(16)
C(16)	0.0224(18)	0.0277(19)	0.0216(17)	−0.0003(14)	0.0041(14)	−0.0040(14)
P(21)	0.0122(1)	0.0235(1)	0.0131(1)	0.0016(1)	0.0026(1)	−0.0009(1)
C(21)	0.0219(17)	0.0217(17)	0.0156(15)	−0.0006(13)	0.0065(13)	−0.0030(14)
P(22)	0.0119(4)	0.0304(5)	0.0152(4)	0.0002(3)	0.0021(3)	−0.0001(3)
C(22)	0.0252(18)	0.0243(18)	0.0209(17)	0.0009(14)	0.0065(14)	−0.0004(14)
C(26)	0.0243(18)	0.0251(18)	0.0176(16)	0.0004(13)	0.0040(14)	−0.0037(14)
C(25)	0.030(2)	0.034(2)	0.0191(17)	−0.0024(15)	0.0048(15)	−0.0115(16)
C(24)	0.049(2)	0.0240(19)	0.028(2)	−0.0054(15)	0.0085(17)	−0.0097(17)
C(23)	0.039(2)	0.0237(19)	0.0291(19)	−0.0014(15)	0.0059(16)	0.0012(16)
C(31)	0.0209(11)	0.0409(13)	0.0485(14)	0.0049(11)	0.0021(10)	−0.0082(10)
C(32)	0.0361(12)	0.0287(11)	0.0292(11)	−0.0048(9)	0.0048(9)	0.0035(9)
C(33)	0.0209(11)	0.0409(13)	0.0485(14)	0.0049(11)	0.0021(10)	−0.0082(10)
C(94)	0.11(2)	0.13(2)	0.088(18)	−0.005(13)	0.036(14)	0.030(16)
C(95)	0.039(8)	0.068(11)	0.088(18)	0.005(9)	0.010(8)	0.013(8)
C(93)	0.061(7)	0.070(7)	0.076(8)	−0.007(6)	0.001(6)	0.007(6)
C(92)	0.056(8)	0.044(6)	0.046(6)	0.005(5)	0.006(5)	0.010(5)
C(34)	0.0209(11)	0.0409(13)	0.0485(14)	0.0049(11)	0.0021(10)	−0.0082(10)
C(35)	0.0361(12)	0.0287(11)	0.0292(11)	−0.0048(9)	0.0048(9)	0.0035(9)
C(36)	0.0361(12)	0.0287(11)	0.0292(11)	−0.0048(9)	0.0048(9)	0.0035(9)
C(43)	0.0367(13)	0.0455(14)	0.0470(15)	−0.0035(11)	0.0223(12)	0.0030(11)
C(42)	0.0339(13)	0.0352(13)	0.0449(14)	0.0108(11)	0.0093(11)	0.0112(10)
C(41)	0.0367(13)	0.0455(14)	0.0470(15)	−0.0035(11)	0.0223(12)	0.0030(11)
C(44)	0.0367(13)	0.0455(14)	0.0470(15)	−0.0035(11)	0.0223(12)	0.0030(11)
C(46)	0.0339(13)	0.0352(13)	0.0449(14)	0.0108(11)	0.0093(11)	0.0112(10)
C(45)	0.0339(13)	0.0352(13)	0.0449(14)	0.0108(11)	0.0093(11)	0.0112(10)

Continued on next page

Table S18. – continued from previous page

atom	U_{11}	U_{22}	U_{33}	U_{23}	U_{13}	U_{12}
C(54)	0.0139(15)	0.0196(16)	0.0190(16)	0.0011(13)	0.0037(12)	−0.0010(13)
C(55)	0.0203(16)	0.0182(16)	0.0157(15)	0.0047(12)	0.0056(13)	0.0008(13)
C(56)	0.0185(16)	0.0232(17)	0.0173(16)	0.0026(13)	0.0000(13)	0.0020(13)
C(61)	0.0160(16)	0.0295(19)	0.0169(16)	0.0026(13)	0.0017(13)	−0.0021(14)
C(62)	0.0166(17)	0.034(2)	0.0186(17)	0.0004(14)	0.0020(13)	−0.0007(14)
C(63)	0.0156(17)	0.045(2)	0.0234(18)	0.0006(16)	0.0007(14)	0.0012(16)
C(64)	0.0194(18)	0.051(2)	0.0266(19)	0.0040(17)	−0.0041(15)	−0.0008(17)
C(65)	0.0257(19)	0.045(2)	0.0186(17)	−0.0020(16)	−0.0018(14)	−0.0006(17)
C(66)	0.0204(18)	0.0337(19)	0.0191(17)	0.0010(14)	0.0013(14)	0.0003(15)
C(71)	0.0224(11)	0.0250(10)	0.0196(9)	−0.0021(8)	−0.0002(8)	−0.0026(8)
C(72)	0.0122(1)	0.0235(1)	0.0131(1)	0.0016(1)	0.0026(1)	−0.0009(1)
C(73)	0.0224(11)	0.0250(10)	0.0196(9)	−0.0021(8)	−0.0002(8)	−0.0026(8)
C(74)	0.0224(11)	0.0250(10)	0.0196(9)	−0.0021(8)	−0.0002(8)	−0.0026(8)
C(75)	0.0122(1)	0.0235(1)	0.0131(1)	0.0016(1)	0.0026(1)	−0.0009(1)
C(76)	0.0122(1)	0.0235(1)	0.0131(1)	0.0016(1)	0.0026(1)	−0.0009(1)
C(81)	0.0256(12)	0.0327(12)	0.0406(13)	−0.0036(10)	−0.0017(10)	0.0009(10)
C(82)	0.0179(10)	0.0400(13)	0.0392(13)	0.0051(10)	0.0077(9)	−0.0005(9)
C(83)	0.0256(12)	0.0327(12)	0.0406(13)	−0.0036(10)	−0.0017(10)	0.0009(10)
C(84)	0.0256(12)	0.0327(12)	0.0406(13)	−0.0036(10)	−0.0017(10)	0.0009(10)
C(85)	0.0179(10)	0.0400(13)	0.0392(13)	0.0051(10)	0.0077(9)	−0.0005(9)
C(86)	0.0179(10)	0.0400(13)	0.0392(13)	0.0051(10)	0.0077(9)	−0.0005(9)
C(53)	0.0149(16)	0.0201(16)	0.0167(15)	0.0001(13)	0.0009(12)	0.0005(13)
C(52)	0.0160(16)	0.0161(15)	0.0163(15)	−0.0012(12)	0.0035(12)	0.0004(12)
C(51)	0.0178(16)	0.0201(16)	0.0144(15)	0.0014(12)	0.0024(12)	0.0014(13)

Table S19. Distances [Å] for {[PC(sp^2)P]PdI}$_2$·½ Et_2O (**{2}$_2$·½ Et$_2$O**).

atom – atom	distance	atom – atom	distance
C(1) – C(11)	1.529(4)	C(1) – C(21)	1.530(4)
C(1) – C(54)	1.588(4)	C(1) – Pd(1)	2.127(3)
I(1) – Pd(1)	2.6851(3)	Pd(1) – P(12)	2.2686(9)
Pd(1) – P(11)	2.3359(9)	C(2) – C(51)	1.380(4)
C(2) – C(61)	1.481(4)	C(2) – Pd(2)	2.043(3)
I(2) – Pd(2)	2.6868(3)	Pd(2) – P(21)	2.2728(8)
Pd(2) – P(22)	2.3104(9)	P(11) – C(12)	1.829(3)
P(11) – C(31)	1.855(4)	P(11) – C(32)	1.874(4)
O(9) – C(93)#1	1.329(11)	O(9) – C(93)	1.329(11)
O(9) – C(92)#1	1.338(11)	O(9) – C(92)	1.338(11)
C(11) – C(16)	1.392(5)	C(11) – C(12)	1.409(5)
P(12) – C(22)	1.813(4)	P(12) – C(42)	1.846(4)
P(12) – C(41)	1.857(4)	C(12) – C(13)	1.398(5)
C(13) – C(14)	1.390(5)	C(13) – H(13)	0.9500
C(14) – C(15)	1.377(5)	C(14) – H(14)	0.9500
C(15) – C(16)	1.387(5)	C(15) – H(15)	0.9500
C(16) – H(16)	0.9500	P(21) – C(52)	1.811(3)
P(21) – C(72)	1.828(3)	P(21) – C(71)	1.851(3)
C(21) – C(22)	1.402(5)	C(21) – C(26)	1.402(5)
P(22) – C(62)	1.819(3)	P(22) – C(81)	1.844(4)
P(22) – C(82)	1.856(4)	C(22) – C(23)	1.389(5)
C(26) – C(25)	1.383(5)	C(26) – H(26)	0.9500
C(25) – C(24)	1.386(5)	C(25) – H(25)	0.9500
C(24) – C(23)	1.378(6)	C(24) – H(24)	0.9500
C(23) – H(23)	0.9500	C(31) – C(33)	1.520(6)
C(31) – C(34)	1.534(6)	C(31) – H(31)	1.0000
C(32) – C(36)	1.503(5)	C(32) – C(35)	1.523(5)
C(32) – H(32)	1.0000	C(33) – H(33A)	0.9800
C(33) – H(33B)	0.9800	C(33) – H(33C)	0.9800
C(94) – C(93)	1.37(4)	C(94) – H(94A)	0.9800
C(94) – H(94B)	0.9800	C(94) – H(94C)	0.9800
C(95) – C(92)	1.55(3)	C(95) – H(95A)	0.9800
C(95) – H(95B)	0.9800	C(95) – H(95C)	0.9800
C(93) – H(93A)	0.9900	C(93) – H(93B)	0.9900
C(92) – H(92A)	0.9900	C(92) – H(92B)	0.9900
C(34) – H(34A)	0.9800	C(34) – H(34B)	0.9800
C(34) – H(34C)	0.9800	C(35) – H(35A)	0.9800
C(35) – H(35B)	0.9800	C(35) – H(35C)	0.9800
C(36) – H(36A)	0.9800	C(36) – H(36B)	0.9800
C(36) – H(36C)	0.9800	C(43) – C(41)	1.517(6)

Symmetry transformations used to generate equivalent atoms: #1 -x+2,-y+1,-z

Continued on next page

Table S19. – continued from previous page

atom – atom	distance	atom – atom	distance
C(43) – H(43A)	0.9800	C(43) – H(43B)	0.9800
C(43) – H(43C)	0.9800	C(42) – C(45)	1.509(6)
C(42) – C(46)	1.524(5)	C(42) – H(42)	1.0000
C(41) – C(44)	1.522(6)	C(41) – H(41)	1.0000
C(44) – H(44A)	0.9800	C(44) – H(44B)	0.9800
C(44) – H(44C)	0.9800	C(46) – H(46A)	0.9800
C(46) – H(46B)	0.9800	C(46) – H(46C)	0.9800
C(45) – H(45A)	0.9800	C(45) – H(45B)	0.9800
C(45) – H(45C)	0.9800	C(54) – C(53)	1.496(4)
C(54) – C(55)	1.508(4)	C(54) – H(54)	1.0000
C(55) – C(56)	1.339(4)	C(55) – H(55)	0.9500
C(56) – C(51)	1.465(4)	C(56) – H(56)	0.9500
C(61) – C(66)	1.408(5)	C(61) – C(62)	1.418(5)
C(62) – C(63)	1.396(5)	C(63) – C(64)	1.385(5)
C(63) – H(63)	0.9500	C(64) – C(65)	1.390(5)
C(64) – H(64)	0.9500	C(65) – C(66)	1.380(5)
C(65) – H(65)	0.9500	C(66) – H(66)	0.9500
C(71) – C(73)	1.528(5)	C(71) – C(74)	1.531(5)
C(71) – H(71)	1.0000	C(72) – C(75)	1.526(4)
C(72) – C(76)	1.526(4)	C(72) – H(72)	1.0000
C(73) – H(73A)	0.9800	C(73) – H(73B)	0.9800
C(73) – H(73C)	0.9800	C(74) – H(74A)	0.9800
C(74) – H(74B)	0.9800	C(74) – H(74C)	0.9800
C(75) – H(75A)	0.9800	C(75) – H(75B)	0.9800
C(75) – H(75C)	0.9800	C(76) – H(76A)	0.9800
C(76) – H(76B)	0.9800	C(76) – H(76C)	0.9800
C(81) – C(83)	1.522(5)	C(81) – C(84)	1.539(5)
C(81) – H(81)	1.0000	C(82) – C(86)	1.513(6)
C(82) – C(85)	1.523(5)	C(82) – H(82)	1.0000
C(83) – H(83A)	0.9800	C(83) – H(83B)	0.9800
C(83) – H(83C)	0.9800	C(84) – H(84A)	0.9800
C(84) – H(84B)	0.9800	C(84) – H(84C)	0.9800
C(85) – H(85A)	0.9800	C(85) – H(85B)	0.9800
C(85) – H(85C)	0.9800	C(86) – H(86A)	0.9800
C(86) – H(86B)	0.9800	C(86) – H(86C)	0.9800
C(53) – C(52)	1.345(4)	C(53) – H(53)	0.9500
C(52) – C(51)	1.465(4)		

Symmetry transformations used to generate equivalent atoms: #1 -x+2,-y+1,-z

Table S20. Angles [°] for {[PC(sp^2)P]PdI}$_2$·½ Et$_2$O (**{2}$_2$·½ Et$_2$O**).

atom – atom – atom	angle	atom – atom – atom	angle
C(11) – C(1) – C(21)	115.5(3)	C(11) – C(1) – C(54)	107.8(2)
C(21) – C(1) – C(54)	109.1(3)	C(11) – C(1) – Pd(1)	106.2(2)
C(21) – C(1) – Pd(1)	113.7(2)	C(54) – C(1) – Pd(1)	103.74(19)
C(1) – Pd(1) – P(12)	85.13(9)	C(1) – Pd(1) – P(11)	83.20(9)
P(12) – Pd(1) – P(11)	155.64(3)	C(1) – Pd(1) – I(1)	167.42(8)
P(12) – Pd(1) – I(1)	95.99(2)	P(11) – Pd(1) – I(1)	100.04(2)
C(51) – C(2) – C(61)	123.6(3)	C(51) – C(2) – Pd(2)	120.8(2)
C(61) – C(2) – Pd(2)	115.6(2)	C(2) – Pd(2) – P(21)	82.65(9)
C(2) – Pd(2) – P(22)	84.06(9)	P(21) – Pd(2) – P(22)	163.58(3)
C(2) – Pd(2) – I(2)	177.19(9)	P(21) – Pd(2) – I(2)	94.63(2)
P(22) – Pd(2) – I(2)	98.75(2)	C(12) – P(11) – C(31)	102.49(17)
C(12) – P(11) – C(32)	110.21(16)	C(31) – P(11) – C(32)	102.30(18)
C(12) – P(11) – Pd(1)	96.89(11)	C(31) – P(11) – Pd(1)	115.43(14)
C(32) – P(11) – Pd(1)	127.02(12)	C(93)#1 – O(9) – C(93)	180.0(11)
C(93)#1 – O(9) – C(92)#1	123.2(6)	C(93) – O(9) – C(92)#1	56.8(6)
C(93)#1 – O(9) – C(92)	56.8(6)	C(93) – O(9) – C(92)	123.2(6)
C(92)#1 – O(9) – C(92)	180.0(9)	C(16) – C(11) – C(12)	117.8(3)
C(16) – C(11) – C(1)	124.1(3)	C(12) – C(11) – C(1)	118.1(3)
C(22) – P(12) – C(42)	111.73(18)	C(22) – P(12) – C(41)	104.10(18)
C(42) – P(12) – C(41)	103.62(19)	C(22) – P(12) – Pd(1)	103.29(12)
C(42) – P(12) – Pd(1)	120.70(14)	C(41) – P(12) – Pd(1)	112.40(15)
C(13) – C(12) – C(11)	120.3(3)	C(13) – C(12) – P(11)	123.5(3)
C(11) – C(12) – P(11)	115.6(2)	C(14) – C(13) – C(12)	120.6(3)
C(14) – C(13) – H(13)	119.7	C(12) – C(13) – H(13)	119.7
C(15) – C(14) – C(13)	118.9(3)	C(15) – C(14) – H(14)	120.6
C(13) – C(14) – H(14)	120.6	C(14) – C(15) – C(16)	121.1(3)
C(14) – C(15) – H(15)	119.5	C(16) – C(15) – H(15)	119.5
C(15) – C(16) – C(11)	121.1(3)	C(15) – C(16) – H(16)	119.4
C(11) – C(16) – H(16)	119.4	C(52) – P(21) – C(72)	103.66(14)
C(52) – P(21) – C(71)	108.20(15)	C(72) – P(21) – C(71)	106.24(15)
C(52) – P(21) – Pd(2)	102.22(10)	C(72) – P(21) – Pd(2)	122.92(10)
C(71) – P(21) – Pd(2)	112.41(11)	C(22) – C(21) – C(26)	116.8(3)
C(22) – C(21) – C(1)	121.7(3)	C(26) – C(21) – C(1)	121.5(3)
C(62) – P(22) – C(81)	108.33(17)	C(62) – P(22) – C(82)	104.29(17)
C(81) – P(22) – C(82)	104.79(17)	C(62) – P(22) – Pd(2)	100.14(11)
C(81) – P(22) – Pd(2)	114.33(12)	C(82) – P(22) – Pd(2)	123.64(13)
C(23) – C(22) – C(21)	121.3(3)	C(23) – C(22) – P(12)	124.4(3)
C(21) – C(22) – P(12)	114.2(3)	C(25) – C(26) – C(21)	121.9(3)
C(25) – C(26) – H(26)	119.1	C(21) – C(26) – H(26)	119.1
C(26) – C(25) – C(24)	120.1(3)	C(26) – C(25) – H(25)	120.0

Symmetry transformations used to generate equivalent atoms: #1 -x+2,-y+1,-z

Continued on next page

Table S20. – continued from previous page

atom – atom – atom	angle	atom – atom – atom	angle
C(24) – C(25) – H(25)	120.0	C(23) – C(24) – C(25)	119.4(3)
C(23) – C(24) – H(24)	120.3	C(25) – C(24) – H(24)	120.3
C(24) – C(23) – C(22)	120.6(3)	C(24) – C(23) – H(23)	119.7
C(22) – C(23) – H(23)	119.7	C(33) – C(31) – C(34)	110.8(3)
C(33) – C(31) – P(11)	110.1(3)	C(34) – C(31) – P(11)	112.6(3)
C(33) – C(31) – H(31)	107.7	C(34) – C(31) – H(31)	107.7
P(11) – C(31) – H(31)	107.7	C(36) – C(32) – C(35)	112.6(3)
C(36) – C(32) – P(11)	114.5(3)	C(35) – C(32) – P(11)	111.1(3)
C(36) – C(32) – H(32)	106.0	C(35) – C(32) – H(32)	106.0
P(11) – C(32) – H(32)	106.0	C(31) – C(33) – H(33A)	109.5
C(31) – C(33) – H(33B)	109.5	H(33A) – C(33) – H(33B)	109.5
C(31) – C(33) – H(33C)	109.5	H(33A) – C(33) – H(33C)	109.5
H(33B) – C(33) – H(33C)	109.5	C(93) – C(94) – H(94A)	109.5
C(93) – C(94) – H(94B)	109.5	H(94A) – C(94) – H(94B)	109.5
C(93) – C(94) – H(94C)	109.5	H(94A) – C(94) – H(94C)	109.5
H(94B) – C(94) – H(94C)	109.5	O(9) – C(93) – C(94)	115.8(14)
O(9) – C(93) – H(93A)	108.3	C(94) – C(93) – H(93A)	108.3
O(9) – C(93) – H(93B)	108.3	C(94) – C(93) – H(93B)	108.3
H(93A) – C(93) – H(93B)	107.4	O(9) – C(92) – C(95)	116.4(15)
O(9) – C(92) – H(92A)	108.2	C(95) – C(92) – H(92A)	108.2
O(9) – C(92) – H(92B)	108.2	C(95) – C(92) – H(92B)	108.2
H(92A) – C(92) – H(92B)	107.3	C(31) – C(34) – H(34A)	109.5
C(31) – C(34) – H(34B)	109.5	H(34A) – C(34) – H(34B)	109.5
C(31) – C(34) – H(34C)	109.5	H(34A) – C(34) – H(34C)	109.5
H(34B) – C(34) – H(34C)	109.5	C(32) – C(35) – H(35A)	109.5
C(32) – C(35) – H(35B)	109.5	H(35A) – C(35) – H(35B)	109.5
C(32) – C(35) – H(35C)	109.5	H(35A) – C(35) – H(35C)	109.5
H(35B) – C(35) – H(35C)	109.5	C(32) – C(36) – H(36A)	109.5
C(32) – C(36) – H(36B)	109.5	H(36A) – C(36) – H(36B)	109.5
C(32) – C(36) – H(36C)	109.5	H(36A) – C(36) – H(36C)	109.5
H(36B) – C(36) – H(36C)	109.5	C(41) – C(43) – H(43A)	109.5
C(41) – C(43) – H(43B)	109.5	H(43A) – C(43) – H(43B)	109.5
C(41) – C(43) – H(43C)	109.5	H(43A) – C(43) – H(43C)	109.5
H(43B) – C(43) – H(43C)	109.5	C(45) – C(42) – C(46)	110.6(3)
C(45) – C(42) – P(12)	118.8(3)	C(46) – C(42) – P(12)	109.8(3)
C(45) – C(42) – H(42)	105.5	C(46) – C(42) – H(42)	105.5
P(12) – C(42) – H(42)	105.5	C(43) – C(41) – C(44)	111.8(4)
C(43) – C(41) – P(12)	111.3(3)	C(44) – C(41) – P(12)	110.0(3)
C(43) – C(41) – H(41)	107.8	C(44) – C(41) – H(41)	107.8
P(12) – C(41) – H(41)	107.8	C(41) – C(44) – H(44A)	109.5
C(41) – C(44) – H(44B)	109.5	H(44A) – C(44) – H(44B)	109.5

Symmetry transformations used to generate equivalent atoms: #1 -x+2,-y+1,-z

Continued on next page

Table S20. – continued from previous page

atom – atom – atom	angle	atom – atom – atom	angle
C(41) – C(44) – H(44C)	109.5	H(44A) – C(44) – H(44C)	109.5
H(44B) – C(44) – H(44C)	109.5	C(42) – C(46) – H(46A)	109.5
C(42) – C(46) – H(46B)	109.5	H(46A) – C(46) – H(46B)	109.5
C(42) – C(46) – H(46C)	109.5	H(46A) – C(46) – H(46C)	109.5
H(46B) – C(46) – H(46C)	109.5	C(42) – C(45) – H(45A)	109.5
C(42) – C(45) – H(45B)	109.5	H(45A) – C(45) – H(45B)	109.5
C(42) – C(45) – H(45C)	109.5	H(45A) – C(45) – H(45C)	109.5
H(45B) – C(45) – H(45C)	109.5	C(53) – C(54) – C(55)	110.6(3)
C(53) – C(54) – C(1)	113.1(3)	C(55) – C(54) – C(1)	116.7(3)
C(53) – C(54) – H(54)	105.1	C(55) – C(54) – H(54)	105.1
C(1) – C(54) – H(54)	105.1	C(56) – C(55) – C(54)	124.8(3)
C(56) – C(55) – H(55)	117.6	C(54) – C(55) – H(55)	117.6
C(55) – C(56) – C(51)	123.0(3)	C(55) – C(56) – H(56)	118.5
C(51) – C(56) – H(56)	118.5	C(66) – C(61) – C(62)	117.0(3)
C(66) – C(61) – C(2)	123.8(3)	C(62) – C(61) – C(2)	119.0(3)
C(63) – C(62) – C(61)	120.6(3)	C(63) – C(62) – P(22)	124.4(3)
C(61) – C(62) – P(22)	114.5(2)	C(64) – C(63) – C(62)	120.5(3)
C(64) – C(63) – H(63)	119.8	C(62) – C(63) – H(63)	119.8
C(63) – C(64) – C(65)	119.6(3)	C(63) – C(64) – H(64)	120.2
C(65) – C(64) – H(64)	120.2	C(66) – C(65) – C(64)	120.3(3)
C(66) – C(65) – H(65)	119.9	C(64) – C(65) – H(65)	119.9
C(65) – C(66) – C(61)	121.7(3)	C(65) – C(66) – H(66)	119.1
C(61) – C(66) – H(66)	119.1	C(73) – C(71) – C(74)	110.7(3)
C(73) – C(71) – P(21)	117.8(2)	C(74) – C(71) – P(21)	108.2(2)
C(73) – C(71) – H(71)	106.5	C(74) – C(71) – H(71)	106.5
P(21) – C(71) – H(71)	106.5	C(75) – C(72) – C(76)	111.0(3)
C(75) – C(72) – P(21)	111.9(2)	C(76) – C(72) – P(21)	111.4(2)
C(75) – C(72) – H(72)	107.4	C(76) – C(72) – H(72)	107.4
P(21) – C(72) – H(72)	107.4	C(71) – C(73) – H(73A)	109.5
C(71) – C(73) – H(73B)	109.5	H(73A) – C(73) – H(73B)	109.5
C(71) – C(73) – H(73C)	109.5	H(73A) – C(73) – H(73C)	109.5
H(73B) – C(73) – H(73C)	109.5	C(71) – C(74) – H(74A)	109.5
C(71) – C(74) – H(74B)	109.5	H(74A) – C(74) – H(74B)	109.5
C(71) – C(74) – H(74C)	109.5	H(74A) – C(74) – H(74C)	109.5
H(74B) – C(74) – H(74C)	109.5	C(72) – C(75) – H(75A)	109.5
C(72) – C(75) – H(75B)	109.5	H(75A) – C(75) – H(75B)	109.5
C(72) – C(75) – H(75C)	109.5	H(75A) – C(75) – H(75C)	109.5
H(75B) – C(75) – H(75C)	109.5	C(72) – C(76) – H(76A)	109.5
C(72) – C(76) – H(76B)	109.5	H(76A) – C(76) – H(76B)	109.5
C(72) – C(76) – H(76C)	109.5	H(76A) – C(76) – H(76C)	109.5
H(76B) – C(76) – H(76C)	109.5	C(83) – C(81) – C(84)	110.5(3)

Symmetry transformations used to generate equivalent atoms: #1 -x+2,-y+1,-z

Continued on next page

Table S20. – continued from previous page

atom – atom – atom	angle	atom – atom – atom	angle
C(83) – C(81) – P(22)	110.3(3)	C(84) – C(81) – P(22)	114.2(3)
C(83) – C(81) – H(81)	107.2	C(84) – C(81) – H(81)	107.2
P(22) – C(81) – H(81)	107.2	C(86) – C(82) – C(85)	111.9(3)
C(86) – C(82) – P(22)	110.3(2)	C(85) – C(82) – P(22)	112.2(3)
C(86) – C(82) – H(82)	107.4	C(85) – C(82) – H(82)	107.4
P(22) – C(82) – H(82)	107.4	C(81) – C(83) – H(83A)	109.5
C(81) – C(83) – H(83B)	109.5	H(83A) – C(83) – H(83B)	109.5
C(81) – C(83) – H(83C)	109.5	H(83A) – C(83) – H(83C)	109.5
H(83B) – C(83) – H(83C)	109.5	C(81) – C(84) – H(84A)	109.5
C(81) – C(84) – H(84B)	109.5	H(84A) – C(84) – H(84B)	109.5
C(81) – C(84) – H(84C)	109.5	H(84A) – C(84) – H(84C)	109.5
H(84B) – C(84) – H(84C)	109.5	C(82) – C(85) – H(85A)	109.5
C(82) – C(85) – H(85B)	109.5	H(85A) – C(85) – H(85B)	109.5
C(82) – C(85) – H(85C)	109.5	H(85A) – C(85) – H(85C)	109.5
H(85B) – C(85) – H(85C)	109.5	C(82) – C(86) – H(86A)	109.5
C(82) – C(86) – H(86B)	109.5	H(86A) – C(86) – H(86B)	109.5
C(82) – C(86) – H(86C)	109.5	H(86A) – C(86) – H(86C)	109.5
H(86B) – C(86) – H(86C)	109.5	C(52) – C(53) – C(54)	123.5(3)
C(52) – C(53) – H(53)	118.2	C(54) – C(53) – H(53)	118.2
C(53) – C(52) – C(51)	124.2(3)	C(53) – C(52) – P(21)	123.6(2)
C(51) – C(52) – P(21)	112.0(2)	C(2) – C(51) – C(52)	119.1(3)
C(2) – C(51) – C(56)	127.4(3)	C(52) – C(51) – C(56)	113.5(3)

Symmetry transformations used to generate equivalent atoms: #1 -x+2,-y+1,-z

8.2 Crystal data for [PC$^{\bullet}$(sp^2)P]PdBr (3)

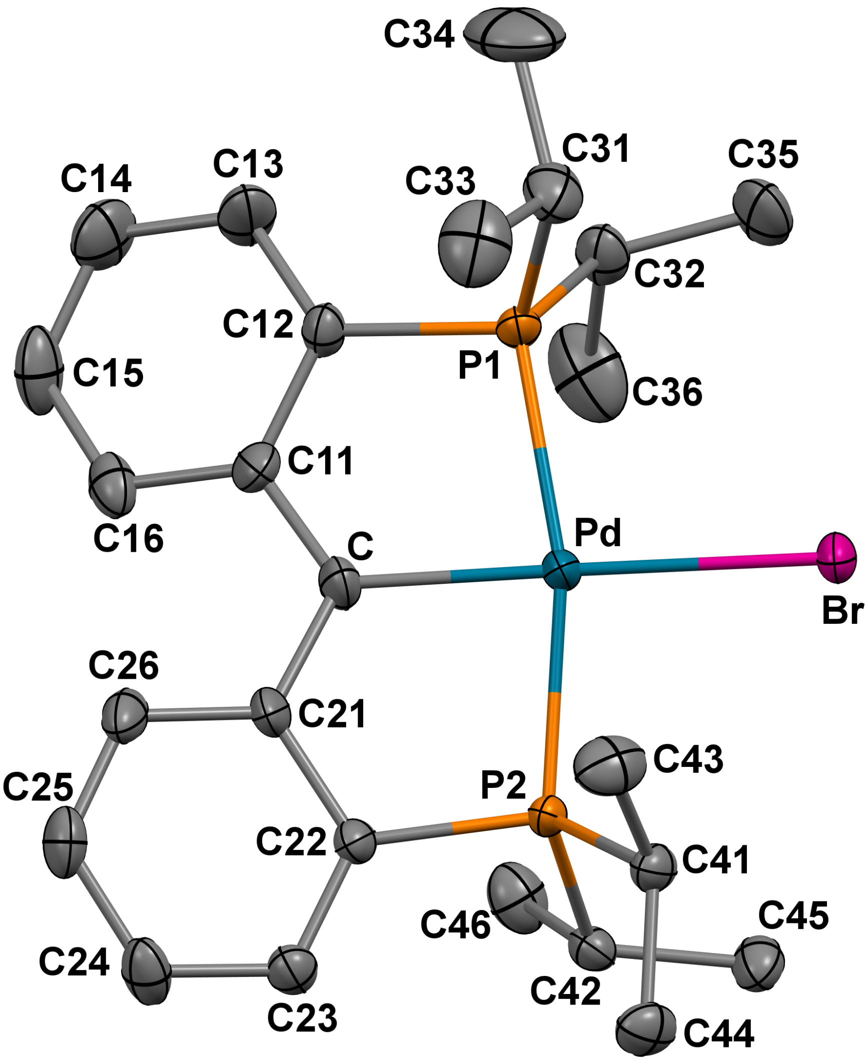

Figure S74. Thermal-ellipsoid (50% probability) representation of [PC$^{\bullet}$(sp^2)P]PdBr (**3**). Hydrogen atoms were omitted for clarity.

Table S21. Crystal data and structure refinement for [PC•(sp^2)P]PdBr (**3**).

Identification code:	cc277	
Empirical formula:	$C_{25}H_{36}BrP_2Pd$	
Formula weight:	584.79	
Temperature:	120(2) K	
Wavelength:	0.71073 Å	
Crystal system:	Monoclinic	
Space group:	$P2_1/n$	
Unit cell dimensions:	a = 12.5686(12) Å	α = 90°
	b = 13.9781(13) Å	β = 97.3300(18)°
	c = 14.9939(14) Å	γ = 90°
Volume:	2612.7(4) Å^3	
Z:	4	
Density (calculated):	1.487 g·cm^{-3}	
Absorption coefficient (μ):	2.372 mm^{-1}	
F(000):	1188	
Crystal size:	0.09 × 0.08 × 0.07 mm^3	
θ range for data collection:	1.99 to 24.99°	
Index ranges:	−14 ≤ h ≤ 14, −16 ≤ k ≤ 16, −17 ≤ l ≤ 17	
Reflections collected:	33653	
Independent reflections:	4601 [R_{int} = 0.0528]	
Completeness to θ = 24.99°:	100.0 %	
Absorption correction:	Semi-empirical from equivalents	
Max. and min. transmission:	0.7456 and 0.6456	
Refinement method:	Full-matrix least-squares on F^2	
Data / restraints / parameters:	4601 / 0 / 270	
Goodness-of-fit on F^2:	1.031	
Final R indices [I>2σ(I)]:	R_1 = 0.0258, wR_2 = 0.0542	
R indices (all data):	R_1 = 0.0375, wR_2 = 0.0569	
Largest diff. peak and hole:	0.617 and −0.548 e$^-$·Å^{-3}	

Table S22. Atomic coordinates and equivalent isotropic displacement parameters ($Å^2$) for [PC$^•$(sp^2)P]PdBr (**3**). U(eq) is defined as one third of the trace of the orthogonalized U_{ij} tensor

atom	x	y	z	U(eq)
Pd	0.46609(2)	0.67536(1)	0.28225(1)	0.022(1)
C	0.5626(3)	0.7301(3)	0.3880(3)	0.025(2)
Br	0.33607(2)	0.60275(2)	0.15975(2)	0.022(1)
P(2)	0.35448(5)	0.79619(5)	0.31262(5)	0.018(1)
P(1)	0.60486(6)	0.57043(5)	0.27643(5)	0.019(1)
C(11)	0.6776(2)	0.7085(2)	0.39585(19)	0.026(1)
C(12)	0.7106(2)	0.62699(19)	0.35196(18)	0.022(1)
C(13)	0.8179(2)	0.6013(2)	0.3607(2)	0.040(1)
C(14)	0.8942(3)	0.6566(3)	0.4114(2)	0.041(1)
C(15)	0.8636(3)	0.7393(2)	0.4501(2)	0.039(1)
C(16)	0.7581(2)	0.7659(2)	0.44299(19)	0.029(1)
C(21)	0.5161(2)	0.7942(2)	0.4498(2)	0.029(1)
C(22)	0.4175(2)	0.83952(18)	0.41989(19)	0.020(1)
C(23)	0.3745(2)	0.90667(19)	0.47272(19)	0.023(1)
C(24)	0.4240(2)	0.9260(2)	0.5588(2)	0.030(1)
C(25)	0.5164(2)	0.8765(2)	0.5920(2)	0.030(1)
C(26)	0.5620(2)	0.81261(19)	0.5386(2)	0.031(1)
C(31)	0.5865(3)	0.4469(2)	0.3139(2)	0.034(1)
C(32)	0.6617(2)	0.5635(2)	0.1692(2)	0.031(1)
C(33)	0.5474(3)	0.4495(3)	0.4060(2)	0.048(1)
C(34)	0.6825(3)	0.3812(3)	0.3145(3)	0.067(1)
C(35)	0.5962(3)	0.4993(2)	0.1011(2)	0.041(1)
C(36)	0.6660(3)	0.6650(3)	0.1335(2)	0.056(1)
C(41)	0.2199(2)	0.75156(19)	0.32807(19)	0.023(1)
C(42)	0.3372(2)	0.90126(19)	0.23770(19)	0.021(1)
C(43)	0.2325(3)	0.6685(2)	0.3953(2)	0.036(1)
C(44)	0.1401(2)	0.8257(2)	0.3534(2)	0.029(1)
C(45)	0.2627(2)	0.8796(2)	0.15154(19)	0.029(1)
C(46)	0.4476(2)	0.9329(2)	0.2169(2)	0.034(1)
H(13)	0.8391	0.5454	0.3316	0.048
H(14)	0.9673	0.6375	0.4194	0.061
H(15)	0.9166	0.7788	0.4825	0.047
H(16)	0.7389	0.8237	0.4701	0.035
H(23)	0.3107	0.9397	0.4500	0.028
H(24)	0.3950	0.9728	0.5947	0.036
H(25)	0.5482	0.8869	0.6521	0.036
H(26)	0.6259	0.7802	0.5621	0.037
H(31)	0.5273	0.4185	0.2712	0.041
H(32)	0.7364	0.5378	0.1811	0.037

Continued on next page

Table S22. – continued from previous page

atom	x	y	x	U(eq)
H(33A)	0.4828	0.4893	0.4031	0.072
H(33B)	0.6036	0.4765	0.4502	0.072
H(33C)	0.5306	0.3844	0.4240	0.072
H(34A)	0.7090	0.3838	0.2558	0.100
H(34B)	0.6613	0.3155	0.3266	0.100
H(34C)	0.7394	0.4018	0.3613	0.100
H(35A)	0.5227	0.5238	0.0890	0.062
H(35B)	0.5949	0.4342	0.1252	0.062
H(35C)	0.6288	0.4984	0.0451	0.062
H(36A)	0.7038	0.6652	0.0802	0.084
H(36B)	0.7042	0.7061	0.1799	0.084
H(36C)	0.5929	0.6890	0.1173	0.084
H(41)	0.1885	0.7239	0.2690	0.027
H(42)	0.3051	0.9544	0.2702	0.026
H(43A)	0.1620	0.6406	0.4003	0.055
H(43B)	0.2643	0.6922	0.4542	0.055
H(43C)	0.2793	0.6195	0.3742	0.055
H(44A)	0.1409	0.8815	0.3140	0.043
H(44B)	0.1600	0.8453	0.4160	0.043
H(44C)	0.0679	0.7978	0.3465	0.043
H(45A)	0.2612	0.9346	0.1108	0.043
H(45B)	0.1902	0.8672	0.1662	0.043
H(45C)	0.2890	0.8232	0.1223	0.043
H(46A)	0.4785	0.8828	0.1823	0.052
H(46B)	0.4946	0.9438	0.2733	0.052
H(46C)	0.4407	0.9922	0.1818	0.052

Table S23. Anisotropic displacement parameters (Å^2) for [PC$^•$(sp^2)P]PdBr (**3**). The anisotropic displacement factor exponent takes the form: $-2\pi^2[h^2a^{*2}U_{11} + ... + 2hka^*b^*U_{12}]$.

**atom**	**U_{11}**	**U_{22}**	**U_{33}**	**U_{23}**	**U_{13}**	**U_{12}**
Pd	0.0192(1)	0.0198(1)	0.0234(1)	−0.0088(1)	−0.0053(1)	0.0046(1)
C	0.024(2)	0.029(3)	0.020(3)	−0.009(3)	−0.0021(2)	0.0012(2)
Br	0.0217(2)	0.0209(2)	0.0222(2)	−0.0046(1)	−0.0054(1)	−0.0008(1)
P(2)	0.0138(4)	0.0175(4)	0.0219(4)	−0.0051(3)	−0.0014(3)	0.0011(3)
P(1)	0.0150(4)	0.0215(4)	0.0214(4)	−0.0051(3)	0.0013(3)	0.0031(3)
C(11)	0.0269(17)	0.0252(16)	0.0238(16)	−0.0001(13)	−0.0063(13)	0.0052(13)
C(12)	0.0168(15)	0.0277(16)	0.0200(15)	−0.0034(12)	0.0007(12)	−0.0020(12)
C(13)	0.0197(17)	0.057(2)	0.042(2)	−0.0200(17)	0.0029(15)	0.0085(15)
C(14)	0.0207(16)	0.052(3)	0.050(2)	−0.0025(2)	0.0030(15)	−0.0037(17)
C(15)	0.0283(19)	0.059(2)	0.0287(18)	−0.0075(17)	0.0036(15)	−0.0218(17)
C(16)	0.040(2)	0.0263(17)	0.0190(16)	0.0009(13)	−0.0027(14)	−0.0061(14)
C(21)	0.0275(17)	0.0211(15)	0.0333(18)	−0.0115(13)	−0.0109(14)	0.0022(13)
C(22)	0.0184(15)	0.0161(14)	0.0247(16)	−0.0025(12)	−0.0017(12)	−0.0004(11)
C(23)	0.0187(15)	0.0253(16)	0.0259(16)	−0.0041(13)	0.0041(12)	−0.0001(12)
C(24)	0.0257(17)	0.0427(19)	0.0238(17)	−0.0124(14)	0.0072(14)	−0.0057(14)
C(25)	0.0300(18)	0.0388(19)	0.0190(16)	−0.0015(14)	−0.0032(13)	−0.0082(14)
C(26)	0.0303(18)	0.0195(16)	0.0385(19)	−0.0011(13)	−0.0138(15)	−0.0014(13)
C(31)	0.039(2)	0.0249(17)	0.0381(19)	−0.0002(14)	−0.0020(15)	−0.0036(14)
C(32)	0.0240(17)	0.0420(19)	0.0260(17)	−0.0103(14)	0.0057(13)	0.0012(14)
C(33)	0.050(2)	0.050(2)	0.044(2)	0.0122(18)	0.0046(18)	−0.0146(18)
C(34)	0.090(3)	0.037(2)	0.073(3)	0.008(2)	0.010(3)	0.031(2)
C(35)	0.041(2)	0.053(2)	0.0304(19)	−0.0200(16)	0.0059(16)	0.0056(17)
C(36)	0.080(3)	0.059(3)	0.033(2)	−0.0011(18)	0.023(2)	−0.022(2)
C(41)	0.0163(15)	0.0209(15)	0.0301(17)	−0.0030(13)	−0.0010(12)	−0.0008(12)
C(42)	0.0230(16)	0.0185(14)	0.0225(15)	−0.0042(12)	0.0033(12)	0.0009(12)
C(43)	0.0316(19)	0.0271(17)	0.052(2)	0.0078(15)	0.0075(16)	−0.0027(14)
C(44)	0.0176(15)	0.0285(17)	0.0405(19)	−0.0016(14)	0.0071(14)	−0.0004(12)
C(45)	0.0327(18)	0.0242(16)	0.0273(17)	0.0024(13)	−0.0046(14)	0.0000(13)
C(46)	0.0363(19)	0.0343(18)	0.0340(19)	−0.0037(15)	0.0097(15)	−0.0108(14)

Table S24. Distances [Å] for [PC$^{\bullet}$(sp^2)P]PdBr (**3**).

**atom – atom**	**distance**	**atom – atom**	**distance**
Pd – C	2.020(3)	Pd – P(2)	2.2783(7)
Pd – P(1)	2.2891(7)	Pd – Br	2.5117(4)
C – C(21)	1.463(4)	C – C(11)	1.467(4)
P(2) – C(22)	1.805(3)	P(2) – C(41)	1.845(3)
P(2) – C(42)	1.845(3)	P(1) – C(12)	1.814(3)
P(1) – C(31)	1.839(3)	P(1) – C(32)	1.844(3)
C(11) – C(12)	1.404(4)	C(11) – C(16)	1.409(4)
C(12) – C(13)	1.385(4)	C(13) – C(14)	1.382(4)
C(13) – H(13)	0.9500	C(14) – C(15)	1.369(5)
C(14) – H(14)	0.9500	C(15) – C(16)	1.369(4)
C(15) – H(15)	0.9500	C(16) – H(16)	0.9500
C(21) – C(26)	1.404(4)	C(21) – C(22)	1.413(4)
C(22) – C(23)	1.382(4)	C(23) – C(24)	1.386(4)
C(23) – H(23)	0.9500	C(24) – C(25)	1.388(4)
C(24) – H(24)	0.9500	C(25) – C(26)	1.373(4)
C(25) – H(25)	0.9500	C(26) – H(26)	0.9500
C(31) – C(34)	1.516(5)	C(31) – C(33)	1.525(5)
C(31) – H(31)	1.0000	C(32) – C(36)	1.519(5)
C(32) – C(35)	1.520(4)	C(32) – H(32)	1.0000
C(33) – H(33A)	0.9800	C(33) – H(33B)	0.9800
C(33) – H(33C)	0.9800	C(34) – H(34A)	0.9800
C(34) – H(34B)	0.9800	C(34) – H(34C)	0.9800
C(35) – H(35A)	0.9800	C(35) – H(35B)	0.9800
C(35) – H(35C)	0.9800	C(36) – H(36A)	0.9800
C(36) – H(36B)	0.9800	C(36) – H(36C)	0.9800
C(41) – C(44)	1.524(4)	C(41) – C(43)	1.532(4)
C(41) – H(41)	1.0000	C(42) – C(45)	1.526(4)
C(42) – C(46)	1.526(4)	C(42) – H(42)	1.0000
C(43) – H(43A)	0.9800	C(43) – H(43B)	0.9800
C(43) – H(43C)	0.9800	C(44) – H(44A)	0.9800
C(44) – H(44B)	0.9800	C(44) – H(44C)	0.9800
C(45) – H(45A)	0.9800	C(45) – H(45B)	0.9800
C(45) – H(45C)	0.9800	C(46) – H(46A)	0.9800
C(46) – H(46B)	0.9800	C(46) – H(46C)	0.9800

Table S25. Angles [°] for [PC$^\bullet$(sp^2)P]PdBr (**3**).

**atom – atom – atom**	**angle**	**atom – atom – atom**	**angle**
C – Pd – P(2)	83.43(10)	C – Pd – P(1)	83.61(10)
P(2) – Pd – P(1)	166.96(3)	C – Pd – Br	175.31(15)
P(2) – Pd – Br	94.93(2)	P(1) – Pd – Br	98.10(2)
C(21) – C – C(11)	123.0(3)	C(21) – C – Pd	118.7(2)
C(11) – C – Pd	118.1(2)	C(22) – P(2) – C(41)	107.88(13)
C(22) – P(2) – C(42)	106.24(12)	C(41) – P(2) – C(42)	107.87(12)
C(22) – P(2) – Pd	102.43(9)	C(41) – P(2) – Pd	111.84(9)
C(42) – P(2) – Pd	119.76(9)	C(12) – P(1) – C(31)	109.20(14)
C(12) – P(1) – C(32)	103.48(13)	C(31) – P(1) – C(32)	107.14(15)
C(12) – P(1) – Pd	101.42(9)	C(31) – P(1) – Pd	117.65(11)
C(32) – P(1) – Pd	116.67(10)	C(12) – C(11) – C(16)	117.5(3)
C(12) – C(11) – C	118.5(3)	C(16) – C(11) – C	124.0(3)
C(13) – C(12) – C(11)	120.5(3)	C(13) – C(12) – P(1)	125.0(2)
C(11) – C(12) – P(1)	114.2(2)	C(14) – C(13) – C(12)	120.4(3)
C(14) – C(13) – H(13)	119.8	C(12) – C(13) – H(13)	119.8
C(15) – C(14) – C(13)	119.5(3)	C(15) – C(14) – H(14)	120.2
C(13) – C(14) – H(14)	120.2	C(16) – C(15) – C(14)	121.2(3)
C(16) – C(15) – H(15)	119.4	C(14) – C(15) – H(15)	119.4
C(15) – C(16) – C(11)	120.7(3)	C(15) – C(16) – H(16)	119.7
C(11) – C(16) – H(16)	119.7	C(26) – C(21) – C(22)	117.1(3)
C(26) – C(21) – C	124.3(3)	C(22) – C(21) – C	118.6(3)
C(23) – C(22) – C(21)	120.9(3)	C(23) – C(22) – P(2)	125.4(2)
C(21) – C(22) – P(2)	113.6(2)	C(22) – C(23) – C(24)	120.2(3)
C(22) – C(23) – H(23)	119.9	C(24) – C(23) – H(23)	119.9
C(23) – C(24) – C(25)	119.5(3)	C(23) – C(24) – H(24)	120.2
C(25) – C(24) – H(24)	120.2	C(26) – C(25) – C(24)	120.5(3)
C(26) – C(25) – H(25)	119.8	C(24) – C(25) – H(25)	119.8
C(25) – C(26) – C(21)	121.3(3)	C(25) – C(26) – H(26)	119.3
C(21) – C(26) – H(26)	119.3	C(34) – C(31) – C(33)	111.0(3)
C(34) – C(31) – P(1)	116.1(3)	C(33) – C(31) – P(1)	108.8(2)
C(34) – C(31) – H(31)	106.8	C(33) – C(31) – H(31)	106.8
P(1) – C(31) – H(31)	106.8	C(36) – C(32) – C(35)	110.8(3)
C(36) – C(32) – P(1)	107.1(2)	C(35) – C(32) – P(1)	112.3(2)
C(36) – C(32) – H(32)	108.8	C(35) – C(32) – H(32)	108.8
P(1) – C(32) – H(32)	108.8	C(31) – C(33) – H(33A)	109.5
C(31) – C(33) – H(33B)	109.5	H(33A) – C(33) – H(33B)	109.5
C(31) – C(33) – H(33C)	109.5	H(33A) – C(33) – H(33C)	109.5
H(33B) – C(33) – H(33C)	109.5	C(31) – C(34) – H(34A)	109.5
C(31) – C(34) – H(34B)	109.5	H(34A) – C(34) – H(34B)	109.5
C(31) – C(34) – H(34C)	109.5	H(34A) – C(34) – H(34C)	109.5
H(34B) – C(34) – H(34C)	109.5	C(32) – C(35) – H(35A)	109.5

Continued on next page

Table S25. – continued from previous page

atom – atom – atom	angle	atom – atom – atom	angle
C(32) – C(35) – H(35B)	109.5	H(35A) – C(35) – H(35B)	109.5
C(32) – C(35) – H(35C)	109.5	H(35A) – C(35) – H(35C)	109.5
H(35B) – C(35) – H(35C)	109.5	C(32) – C(36) – H(36A)	109.5
C(32) – C(36) – H(36B)	109.5	H(36A) – C(36) – H(36B)	109.5
C(32) – C(36) – H(36C)	109.5	H(36A) – C(36) – H(36C)	109.5
H(36B) – C(36) – H(36C)	109.5	C(44) – C(41) – C(43)	111.6(2)
C(44) – C(41) – P(2)	116.42(19)	C(43) – C(41) – P(2)	108.63(19)
C(44) – C(41) – H(41)	106.6	C(43) – C(41) – H(41)	106.6
P(2) – C(41) – H(41)	106.6	C(45) – C(42) – C(46)	111.1(2)
C(45) – C(42) – P(2)	111.70(18)	C(46) – C(42) – P(2)	108.4(2)
C(45) – C(42) – H(42)	108.5	C(46) – C(42) – H(42)	108.5
P(2) – C(42) – H(42)	108.5	C(41) – C(43) – H(43A)	109.5
C(41) – C(43) – H(43B)	109.5	H(43A) – C(43) – H(43B)	109.5
C(41) – C(43) – H(43C)	109.5	H(43A) – C(43) – H(43C)	109.5
H(43B) – C(43) – H(43C)	109.5	C(41) – C(44) – H(44A)	109.5
C(41) – C(44) – H(44B)	109.5	H(44A) – C(44) – H(44B)	109.5
C(41) – C(44) – H(44C)	109.5	H(44A) – C(44) – H(44C)	109.5
H(44B) – C(44) – H(44C)	109.5	C(42) – C(45) – H(45A)	109.5
C(42) – C(45) – H(45B)	109.5	H(45A) – C(45) – H(45B)	109.5
C(42) – C(45) – H(45C)	109.5	H(45A) – C(45) – H(45C)	109.5
H(45B) – C(45) – H(45C)	109.5	C(42) – C(46) – H(46A)	109.5
C(42) – C(46) – H(46B)	109.5	H(46A) – C(46) – H(46B)	109.5
C(42) – C(46) – H(46C)	109.5	H(46A) – C(46) – H(46C)	109.5
H(46B) – C(46) – H(46C)	109.5		

8.3 Crystal data for [PC(sp3)HP]PdBr (4)

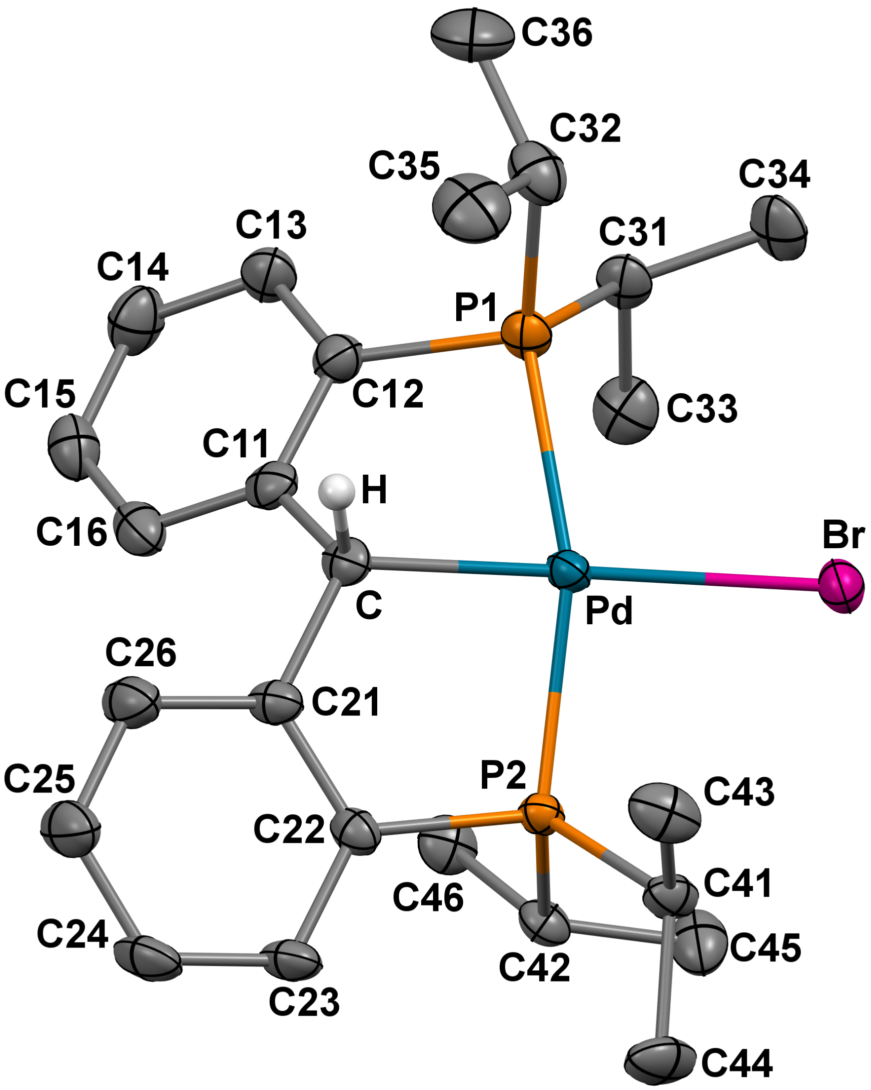

Figure S75. Thermal-ellipsoid representation of [PC(sp^3)HP]PdBr (**4**) at 50% probability. Most hydrogen atoms were omitted for clarity.

Table S26. Crystal data and structure refinement for [PC(sp^3)HP]PdBr (**4**).

Identification code:	cc271	
Empirical formula:	$C_{25}H_{37}BrP_2Pd$	
Formula weight:	585.80	
Temperature:	120(2) K	
Wavelength:	0.71073 Å	
Crystal system:	Monoclinic	
Space group:	$P2_1/n$	
Unit cell dimensions:	a = 11.2438(8) Å	α = 90°
	b = 13.7677(10) Å	β = 106.9775(12)°
	c = 17.4246(12) Å	γ = 90°
Volume:	2579.8(3) $Å^3$	
Z:	4	
Density (calculated):	1.508 $g·cm^{-3}$	
Absorption coefficient (μ):	2.402 mm^{-1}	
F(000):	1192	
Crystal size:	0.13 × 0.10 × 0.05 mm^3	
θ range for data collection:	1.92 to 25.00°	
Index ranges:	$-13 \le h \le 13$, $-16 \le k \le 16$, $-20 \le l \le 20$	
Reflections collected:	33493	
Independent reflections:	4546 [R_{int} = 0.0524]	
Completeness to θ = 25.00°:	100.0 %	
Absorption correction:	Semi-empirical from equivalents	
Max. and min. transmission:	0.7454 and 0.6879	
Refinement method:	Full-matrix least-squares on F^2	
Data / restraints / parameters:	4546 / 0 / 270	
Goodness-of-fit on F^2:	1.044	
Final R indices [I>2σ(I)]:	R_1 = 0.0348, wR_2 = 0.0742	
R indices (all data):	R_1 = 0.0463, wR_2 = 0.0776	
Largest diff. peak and hole:	1.970 and −1.106 $e^-·Å^{-3}$	

Table S27. Atomic coordinates and equivalent isotropic displacement parameters (Å^2) for [PC(sp^3)HP]PdBr (**4**). U(eq) is defined as one third of the trace of the orthogonalized U_{ij} tensor

**atom**	**x**	**y**	**z**	**U(eq)**
Pd	0.02187(3)	0.65787(2)	0.18217(2)	0.019(1)
P(1)	−0.16836(10)	0.57786(7)	0.15980(6)	0.022(1)
Br	0.14254(4)	0.58796(3)	0.31483(2)	0.025(1)
C	−0.0624(4)	0.7074(3)	0.0669(2)	0.022(1)
P(2)	0.16165(9)	0.77727(7)	0.18634(6)	0.017(1)
C(11)	−0.1977(4)	0.7319(3)	0.0586(2)	0.024(1)
C(12)	−0.2653(4)	0.6656(3)	0.0910(2)	0.023(1)
C(13)	−0.3923(4)	0.6779(3)	0.0787(3)	0.029(1)
C(14)	−0.4522(4)	0.7591(3)	0.0378(3)	0.034(1)
C(15)	−0.3865(4)	0.8268(3)	0.0096(3)	0.030(1)
C(16)	−0.2606(4)	0.8131(3)	0.0189(2)	0.029(1)
C(21)	0.0106(4)	0.7824(3)	0.0343(2)	0.023(1)
C(22)	0.1203(4)	0.8214(3)	0.0840(2)	0.021(1)
C(23)	0.1880(4)	0.8894(3)	0.0539(3)	0.026(1)
C(24)	0.1467(4)	0.9163(3)	−0.0261(3)	0.033(1)
C(26)	−0.0289(4)	0.8098(3)	−0.0465(3)	0.032(1)
C(31)	−0.2339(4)	0.5750(3)	0.2457(3)	0.027(1)
C(32)	−0.1970(4)	0.4556(3)	0.1143(3)	0.032(1)
C(33)	−0.2110(4)	0.6723(3)	0.2893(3)	0.035(1)
C(34)	−0.1804(4)	0.4908(3)	0.3027(3)	0.032(1)
C(35)	−0.1365(5)	0.4462(3)	0.0470(3)	0.045(1)
C(36)	−0.3332(5)	0.4262(4)	0.0873(3)	0.051(1)
C(41)	0.3250(3)	0.7389(3)	0.2201(3)	0.023(1)
C(42)	0.1446(4)	0.8840(3)	0.2458(2)	0.021(1)
C(43)	0.3431(4)	0.6533(3)	0.1689(3)	0.032(1)
C(44)	0.4241(4)	0.8165(3)	0.2261(3)	0.036(1)
C(45)	0.1960(5)	0.8659(3)	0.3357(3)	0.034(1)
C(46)	0.0057(4)	0.9097(3)	0.2230(3)	0.027(1)
C(25)	0.0395(5)	0.8752(3)	−0.0756(3)	0.037(1)
H	−0.0666	0.6491	0.0320	0.027
H(13)	−0.4380	0.6309	0.0982	0.035
H(14)	−0.5389	0.7676	0.0294	0.041
H(15)	−0.4271	0.8835	−0.0165	0.036
H(16)	−0.2165	0.8600	−0.0021	0.035
H(23)	0.2619	0.9169	0.0883	0.032
H(24)	0.1918	0.9627	−0.0468	0.040
H(26)	−0.1031	0.7832	−0.0813	0.039
H(31)	−0.3258	0.5653	0.2241	0.032
H(32)	−0.1536	0.4084	0.1570	0.039

Continued on next page

Table S27. – continued from previous page

**atom**	**x**	**y**	**x**	**U(eq)**
H(33A)	−0.2400	0.7250	0.2505	0.052
H(33B)	−0.1218	0.6802	0.3159	0.052
H(33C)	−0.2564	0.6744	0.3294	0.052
H(34A)	−0.2018	0.4291	0.2740	0.048
H(34B)	−0.2155	0.4922	0.3480	0.048
H(34C)	−0.0898	0.4971	0.3227	0.048
H(35A)	−0.1436	0.3789	0.0278	0.067
H(35B)	−0.0484	0.4640	0.0672	0.067
H(35C)	−0.1784	0.4895	0.0027	0.067
H(36A)	−0.3791	0.4692	0.0439	0.076
H(36B)	−0.3679	0.4315	0.1326	0.076
H(36C)	−0.3406	0.3590	0.0680	0.076
H(41)	0.3393	0.7128	0.2757	0.028
H(42)	0.1907	0.9397	0.2310	0.025
H(43A)	0.3269	0.6744	0.1130	0.048
H(43B)	0.2852	0.6011	0.1716	0.048
H(43C)	0.4287	0.6294	0.1890	0.048
H(44A)	0.4241	0.8362	0.1720	0.053
H(44B)	0.5059	0.7902	0.2550	0.053
H(44C)	0.4061	0.8730	0.2551	0.053
H(45A)	0.1612	0.8053	0.3495	0.051
H(45B)	0.1731	0.9200	0.3651	0.051
H(45C)	0.2868	0.8606	0.3504	0.051
H(46A)	−0.0263	0.9182	0.1647	0.041
H(46B)	−0.0054	0.9701	0.2498	0.041
H(46C)	−0.0398	0.8571	0.2398	0.041
H(25)	0.0126	0.8925	−0.1308	0.044

Table S28. Anisotropic displacement parameters (Å^2) for [PC(sp^3)HP]PdBr (**4**). The anisotropic displacement factor exponent takes the form: $-2\pi^2[h^2a^{*2}U_{11} + ...+ 2hka^*b^*U_{12}]$.

**atom**	**U_{11}**	**U_{22}**	**U_{33}**	**U_{23}**	**U_{13}**	**U_{12}**
Pd	0.0217(2)	0.0177(2)	0.0178(2)	0.0015(1)	0.0071(1)	−0.0036(1)
P(1)	0.0191(5)	0.0263(5)	0.0225(6)	0.0034(4)	0.0090(5)	−0.0002(4)
Br	0.0284(2)	0.0227(2)	0.0220(2)	0.0047(2)	0.0056(2)	0.0017(2)
C	0.028(2)	0.023(2)	0.015(2)	−0.0031(16)	0.0071(18)	−0.0042(17)
P(2)	0.0160(5)	0.0170(5)	0.0197(5)	0.0009(4)	0.0061(4)	−0.0014(4)
C(11)	0.027(2)	0.029(2)	0.016(2)	−0.0051(17)	0.0044(18)	−0.0080(18)
C(12)	0.021(2)	0.027(2)	0.022(2)	−0.0006(17)	0.0051(18)	−0.0029(17)
C(13)	0.024(2)	0.036(2)	0.028(2)	0.0054(19)	0.009(2)	−0.0033(19)
C(14)	0.023(2)	0.046(3)	0.033(3)	0.003(2)	0.006(2)	0.006(2)
C(15)	0.038(3)	0.028(2)	0.023(2)	0.0017(18)	0.008(2)	0.007(2)
C(16)	0.043(3)	0.022(2)	0.021(2)	−0.0006(17)	0.008(2)	−0.0036(19)
C(21)	0.025(2)	0.022(2)	0.022(2)	−0.0029(17)	0.0101(18)	−0.0061(17)
C(22)	0.027(2)	0.0189(19)	0.019(2)	0.0001(16)	0.0096(18)	−0.0017(16)
C(23)	0.030(2)	0.024(2)	0.029(2)	−0.0020(18)	0.013(2)	−0.0120(18)
C(24)	0.047(3)	0.030(2)	0.029(3)	−0.0017(19)	0.020(2)	−0.019(2)
C(26)	0.039(3)	0.035(2)	0.023(2)	−0.0009(19)	0.008(2)	−0.014(2)
C(31)	0.021(2)	0.035(2)	0.027(2)	0.0058(18)	0.0119(19)	−0.0021(18)
C(32)	0.042(3)	0.024(2)	0.033(3)	0.0018(19)	0.013(2)	0.004(2)
C(33)	0.036(3)	0.041(3)	0.031(3)	−0.003(2)	0.016(2)	0.002(2)
C(34)	0.033(3)	0.042(3)	0.029(2)	0.012(2)	0.019(2)	0.000(2)
C(35)	0.061(4)	0.034(3)	0.042(3)	−0.011(2)	0.019(3)	−0.001(2)
C(36)	0.055(4)	0.033(3)	0.060(4)	−0.007(2)	0.011(3)	−0.017(2)
C(41)	0.016(2)	0.022(2)	0.032(2)	0.0019(17)	0.0077(18)	−0.0013(16)
C(42)	0.025(2)	0.0153(18)	0.025(2)	0.0002(16)	0.0106(18)	0.0010(16)
C(43)	0.031(2)	0.024(2)	0.049(3)	−0.002(2)	0.022(2)	0.0041(19)
C(44)	0.016(2)	0.030(2)	0.058(3)	0.000(2)	0.008(2)	−0.0040(18)
C(45)	0.049(3)	0.025(2)	0.026(2)	−0.0039(18)	0.006(2)	0.004(2)
C(46)	0.026(2)	0.024(2)	0.034(3)	−0.0014(18)	0.012(2)	0.0056(18)
C(25)	0.048(3)	0.040(3)	0.022(2)	0.002(2)	0.009(2)	−0.016(2)

Table S29. Distances [Å] for [PC(sp^3)HP]PdBr (**4**).

atom – atom	distance	atom – atom	distance
Pd – C	2.071(4)	Pd – P(2)	2.2608(10)
Pd – P(1)	2.3352(11)	Pd – Br	2.5057(5)
P(1) – C(12)	1.822(4)	P(1) – C(32)	1.849(4)
P(1) – C(31)	1.852(4)	C – C(11)	1.523(6)
C – C(21)	1.527(5)	C – H	1.0000
P(2) – C(22)	1.812(4)	P(2) – C(41)	1.835(4)
P(2) – C(42)	1.840(4)	C(11) – C(16)	1.394(6)
C(11) – C(12)	1.407(5)	C(12) – C(13)	1.392(6)
C(13) – C(14)	1.389(6)	C(13) – H(13)	0.9500
C(14) – C(15)	1.366(6)	C(14) – H(14)	0.9500
C(15) – C(16)	1.390(6)	C(15) – H(15)	0.9500
C(16) – H(16)	0.9500	C(21) – C(22)	1.391(5)
C(21) – C(26)	1.398(6)	C(22) – C(23)	1.402(5)
C(23) – C(24)	1.384(6)	C(23) – H(23)	0.9500
C(24) – C(25)	1.382(6)	C(24) – H(24)	0.9500
C(26) – C(25)	1.374(6)	C(26) – H(26)	0.9500
C(31) – C(33)	1.524(6)	C(31) – C(34)	1.531(6)
C(31) – H(31)	1.0000	C(32) – C(36)	1.520(7)
C(32) – C(35)	1.524(6)	C(32) – H(32)	1.0000
C(33) – H(33A)	0.9800	C(33) – H(33B)	0.9800
C(33) – H(33C)	0.9800	C(34) – H(34A)	0.9800
C(34) – H(34B)	0.9800	C(34) – H(34C)	0.9800
C(35) – H(35A)	0.9800	C(35) – H(35B)	0.9800
C(35) – H(35C)	0.9800	C(36) – H(36A)	0.9800
C(36) – H(36B)	0.9800	C(36) – H(36C)	0.9800
C(41) – C(44)	1.526(5)	C(41) – C(43)	1.528(5)
C(41) – H(41)	1.0000	C(42) – C(45)	1.525(6)
C(42) – C(46)	1.536(5)	C(42) – H(42)	1.0000
C(43) – H(43A)	0.9800	C(43) – H(43B)	0.9800
C(43) – H(43C)	0.9800	C(44) – H(44A)	0.9800
C(44) – H(44B)	0.9800	C(44) – H(44C)	0.9800
C(45) – H(45A)	0.9800	C(45) – H(45B)	0.9800
C(45) – H(45C)	0.9800	C(46) – H(46A)	0.9800
C(46) – H(46B)	0.9800	C(46) – H(46C)	0.9800
C(25) – H(25)	0.9500		

Table S30. Angles [°] for [PC(sp^3)HP]PdBr (**4**).

atom – atom – atom	angle	atom – atom – atom	angle
C – Pd – P(2)	84.74(11)	C – Pd – P(1)	81.73(11)
P(2) – Pd – P(1)	160.49(4)	C – Pd – Br	173.06(11)
P(2) – Pd – Br	93.76(3)	P(1) – Pd – Br	101.28(3)
C(12) – P(1) – C(32)	109.0(2)	C(12) – P(1) – C(31)	104.12(19)
C(32) – P(1) – C(31)	105.46(19)	C(12) – P(1) – Pd	98.03(13)
C(32) – P(1) – Pd	122.51(15)	C(31) – P(1) – Pd	115.87(14)
C(11) – C – C(21)	116.4(3)	C(11) – C – Pd	109.0(3)
C(21) – C – Pd	116.1(3)	C(11) – C – H	104.6
C(21) – C – H	104.6	Pd – C – H	104.6
C(22) – P(2) – C(41)	110.95(19)	C(22) – P(2) – C(42)	104.33(17)
C(41) – P(2) – C(42)	107.96(18)	C(22) – P(2) – Pd	103.63(13)
C(41) – P(2) – Pd	114.97(13)	C(42) – P(2) – Pd	114.42(13)
C(16) – C(11) – C(12)	117.7(4)	C(16) – C(11) – C	124.9(4)
C(12) – C(11) – C	117.4(4)	C(13) – C(12) – C(11)	120.6(4)
C(13) – C(12) – P(1)	124.9(3)	C(11) – C(12) – P(1)	113.9(3)
C(14) – C(13) – C(12)	120.0(4)	C(14) – C(13) – H(13)	120.0
C(12) – C(13) – H(13)	120.0	C(15) – C(14) – C(13)	120.1(4)
C(15) – C(14) – H(14)	120.0	C(13) – C(14) – H(14)	120.0
C(14) – C(15) – C(16)	120.3(4)	C(14) – C(15) – H(15)	119.8
C(16) – C(15) – H(15)	119.8	C(15) – C(16) – C(11)	121.2(4)
C(15) – C(16) – H(16)	119.4	C(11) – C(16) – H(16)	119.4
C(22) – C(21) – C(26)	119.0(4)	C(22) – C(21) – C	120.3(3)
C(26) – C(21) – C	120.6(4)	C(21) – C(22) – C(23)	120.3(4)
C(21) – C(22) – P(2)	114.2(3)	C(23) – C(22) – P(2)	125.5(3)
C(24) – C(23) – C(22)	119.8(4)	C(24) – C(23) – H(23)	120.1
C(22) – C(23) – H(23)	120.1	C(25) – C(24) – C(23)	119.5(4)
C(25) – C(24) – H(24)	120.2	C(23) – C(24) – H(24)	120.2
C(25) – C(26) – C(21)	120.0(4)	C(25) – C(26) – H(26)	120.0
C(21) – C(26) – H(26)	120.0	C(33) – C(31) – C(34)	111.4(4)
C(33) – C(31) – P(1)	109.5(3)	C(34) – C(31) – P(1)	111.6(3)
C(33) – C(31) – H(31)	108.1	C(34) – C(31) – H(31)	108.1
P(1) – C(31) – H(31)	108.1	C(36) – C(32) – C(35)	112.1(4)
C(36) – C(32) – P(1)	114.1(3)	C(35) – C(32) – P(1)	110.3(3)
C(36) – C(32) – H(32)	106.6	C(35) – C(32) – H(32)	106.6
P(1) – C(32) – H(32)	106.6	C(31) – C(33) – H(33A)	109.5
C(31) – C(33) – H(33B)	109.5	H(33A) – C(33) – H(33B)	109.5
C(31) – C(33) – H(33C)	109.5	H(33A) – C(33) – H(33C)	109.5
H(33B) – C(33) – H(33C)	109.5	C(31) – C(34) – H(34A)	109.5
C(31) – C(34) – H(34B)	109.5	H(34A) – C(34) – H(34B)	109.5
C(31) – C(34) – H(34C)	109.5	H(34A) – C(34) – H(34C)	109.5
H(34B) – C(34) – H(34C)	109.5	C(32) – C(35) – H(35A)	109.5

Continued on next page

Table S30. – continued from previous page

atom – atom – atom	angle	atom – atom – atom	angle
C(32) – C(35) – H(35B)	109.5	H(35A) – C(35) – H(35B)	109.5
C(32) – C(35) – H(35C)	109.5	H(35A) – C(35) – H(35C)	109.5
H(35B) – C(35) – H(35C)	109.5	C(32) – C(36) – H(36A)	109.5
C(32) – C(36) – H(36B)	109.5	H(36A) – C(36) – H(36B)	109.5
C(32) – C(36) – H(36C)	109.5	H(36A) – C(36) – H(36C)	109.5
H(36B) – C(36) – H(36C)	109.5	C(44) – C(41) – C(43)	111.2(3)
C(44) – C(41) – P(2)	117.6(3)	C(43) – C(41) – P(2)	109.0(3)
C(44) – C(41) – H(41)	106.1	C(43) – C(41) – H(41)	106.1
P(2) – C(41) – H(41)	106.1	C(45) – C(42) – C(46)	110.8(3)
C(45) – C(42) – P(2)	112.1(3)	C(46) – C(42) – P(2)	107.7(3)
C(45) – C(42) – H(42)	108.7	C(46) – C(42) – H(42)	108.7
P(2) – C(42) – H(42)	108.7	C(41) – C(43) – H(43A)	109.5
C(41) – C(43) – H(43B)	109.5	H(43A) – C(43) – H(43B)	109.5
C(41) – C(43) – H(43C)	109.5	H(43A) – C(43) – H(43C)	109.5
H(43B) – C(43) – H(43C)	109.5	C(41) – C(44) – H(44A)	109.5
C(41) – C(44) – H(44B)	109.5	H(44A) – C(44) – H(44B)	109.5
C(41) – C(44) – H(44C)	109.5	H(44A) – C(44) – H(44C)	109.5
H(44B) – C(44) – H(44C)	109.5	C(42) – C(45) – H(45A)	109.5
C(42) – C(45) – H(45B)	109.5	H(45A) – C(45) – H(45B)	109.5
C(42) – C(45) – H(45C)	109.5	H(45A) – C(45) – H(45C)	109.5
H(45B) – C(45) – H(45C)	109.5	C(42) – C(46) – H(46A)	109.5
C(42) – C(46) – H(46B)	109.5	H(46A) – C(46) – H(46B)	109.5
C(42) – C(46) – H(46C)	109.5	H(46A) – C(46) – H(46C)	109.5
H(46B) – C(46) – H(46C)	109.5	C(26) – C(25) – C(24)	121.2(4)
C(26) – C(25) – H(25)	119.4	C(24) – C(25) – H(25)	119.4

8.4 Crystal data for [PC$^{\bullet}$(sp^2)P]PdCl (5)

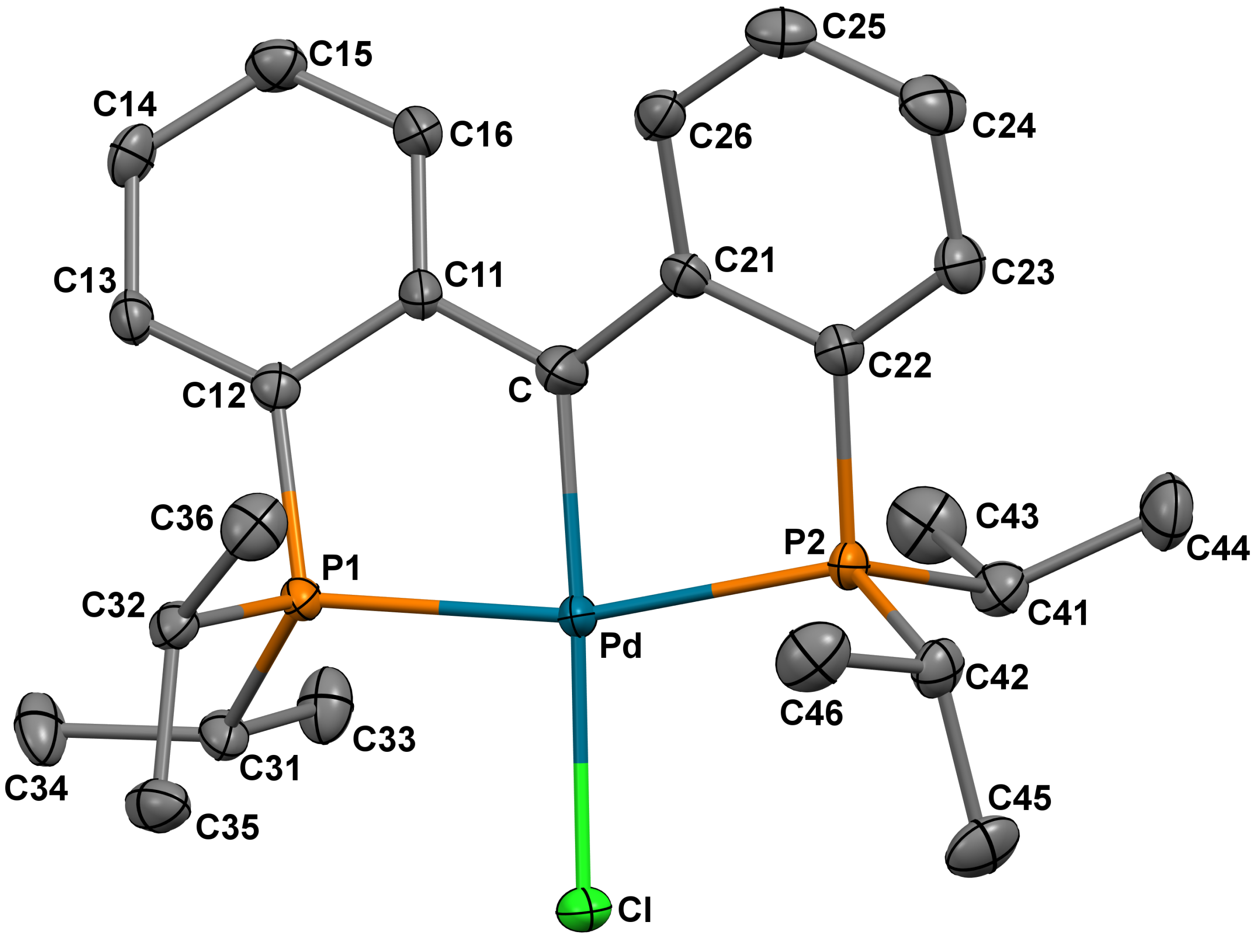

Figure S76. Thermal-ellipsoid (50% probability) representation of [PC$^{\bullet}$(sp^2)P]PdCl (**5**). Hydrogen atoms were omitted for clarity.

Table S31. Crystal data and structure refinement for [PC$^\bullet$(sp^2)P]PdCl (**5**).

Identification code:	cc250	
Empirical formula:	$C_{25}H_{36}ClP_2Pd$	
Formula weight:	540.33	
Temperature:	120(2) K	
Wavelength:	0.71073 Å	
Crystal system:	Monoclinic	
Space group:	$P2_1/n$	
Unit cell dimensions:	a = 11.6639(11) Å	α = 90°
	b = 13.5934(13) Å	β = 105.1159(14)°
	c = 16.5680(16) Å	γ = 90°
Volume:	2536.0(4) Å^3	
Z:	4	
Density (calculated):	1.415 g·cm^{-3}	
Absorption coefficient (μ):	0.973 mm^{-1}	
F(000):	1116	
Crystal size:	0.11 × 0.09 × 0.08 mm^3	
θ range for data collection:	1.97 to 25.00°	
Index ranges:	$-13 \le h \le 13$, $-16 \le k \le 16$, $-19 \le l \le 19$	
Reflections collected:	36052	
Independent reflections:	4436 [R_{int} = 0.0430]	
Completeness to θ = 25.00°:	99.4 %	
Absorption correction:	Semi-empirical from equivalents	
Max. and min. transmission:	0.7457 and 0.6929	
Refinement method:	Full-matrix least-squares on F^2	
Data / restraints / parameters:	4436 / 0 / 270	
Goodness-of-fit on F^2:	1.046	
Final R indices [I>2σ(I)]:	R_1 = 0.0240, wR_2 = 0.0523	
R indices (all data):	R_1 = 0.0298, wR_2 = 0.0541	
Largest diff. peak and hole:	0.664 and −0.356 e$^-$·Å^{-3}	

Table S32. Atomic coordinates and equivalent isotropic displacement parameters (Å^2) for [PC$^\bullet$(sp^2)P]PdCl (**5**). U(eq) is defined as one third of the trace of the orthogonalized U_{ij} tensor

atom	x	y	z	U(eq)
Pd	0.01580(2)	0.82790(1)	0.19451(1)	0.016(1)
P(1)	0.15024(5)	0.70808(4)	0.19016(4)	0.015(1)
Cl	0.14068(5)	0.90818(4)	0.31356(3)	0.021(1)
P(2)	−0.15260(5)	0.92214(4)	0.17604(4)	0.017(1)
C	−0.0783(2)	0.7603(2)	0.09076(16)	0.031(1)
C(11)	−0.0156(2)	0.69972(16)	0.04320(15)	0.020(1)
C(12)	0.09701(19)	0.66181(16)	0.08448(14)	0.017(1)
C(13)	0.1558(2)	0.59550(16)	0.04562(14)	0.018(1)
C(14)	0.1078(2)	0.57109(17)	−0.03797(15)	0.022(1)
C(15)	0.0016(2)	0.61327(17)	−0.08147(15)	0.023(1)
C(16)	−0.0600(2)	0.67567(17)	−0.04212(15)	0.024(1)
C(21)	−0.2064(2)	0.76799(16)	0.06865(14)	0.018(1)
C(22)	−0.2590(2)	0.84699(16)	0.10216(14)	0.018(1)
C(23)	−0.3814(2)	0.85604(18)	0.08361(16)	0.025(1)
C(24)	−0.4551(2)	0.7876(2)	0.03375(16)	0.030(1)
C(25)	−0.4050(2)	0.70819(18)	0.00288(15)	0.025(1)
C(31)	0.30244(19)	0.75402(16)	0.20566(15)	0.019(1)
C(32)	0.1532(2)	0.60218(16)	0.25986(14)	0.019(1)
C(33)	0.2996(2)	0.83900(18)	0.14424(17)	0.028(1)
C(34)	0.3980(2)	0.67946(18)	0.20206(18)	0.030(1)
C(35)	0.2189(2)	0.62597(18)	0.34978(15)	0.027(1)
C(36)	0.0242(2)	0.57315(19)	0.25372(16)	0.027(1)
C(41)	−0.1519(2)	1.04615(17)	0.13154(15)	0.023(1)
C(42)	−0.2167(2)	0.93535(17)	0.26625(15)	0.022(1)
C(43)	−0.0862(2)	1.0439(2)	0.06309(17)	0.036(1)
C(44)	−0.2738(2)	1.09319(19)	0.10055(18)	0.034(1)
C(45)	−0.1491(2)	1.0115(2)	0.32843(16)	0.030(1)
C(46)	−0.2148(3)	0.83412(19)	0.30722(17)	0.033(1)
C(26)	−0.2837(2)	0.69837(17)	0.01975(14)	0.021(1)
H(13)	0.2286	0.5669	0.0760	0.022
H(14)	0.1477	0.5259	−0.0650	0.026
H(15)	−0.0294	0.5990	−0.1392	0.028
H(16)	−0.1334	0.7029	−0.0730	0.028
H(23)	−0.4153	0.9101	0.1055	0.030
H(24)	−0.5389	0.7950	0.0208	0.035
H(25)	−0.4550	0.6601	−0.0303	0.030
H(31)	0.3258	0.7834	0.2630	0.023
H(32)	0.1939	0.5460	0.2400	0.022
H(33A)	0.2744	0.8141	0.0868	0.043

Continued on next page

Table S32. – continued from previous page

atom	x	y	x	U(eq)
H(33B)	0.3791	0.8678	0.1542	0.043
H(33C)	0.2436	0.8893	0.1525	0.043
H(34A)	0.3881	0.6584	0.1441	0.046
H(34B)	0.3912	0.6223	0.2366	0.046
H(34C)	0.4765	0.7096	0.2232	0.046
H(35A)	0.3033	0.6362	0.3535	0.040
H(35B)	0.2101	0.5712	0.3861	0.040
H(35C)	0.1856	0.6859	0.3676	0.040
H(36A)	−0.0181	0.5630	0.1949	0.041
H(36B)	−0.0146	0.6257	0.2773	0.041
H(36C)	0.0229	0.5122	0.2850	0.041
H(41)	−0.1048	1.0891	0.1772	0.028
H(42)	−0.3011	0.9573	0.2454	0.026
H(43A)	−0.1298	1.0024	0.0168	0.054
H(43B)	−0.0063	1.0169	0.0858	0.054
H(43C)	−0.0801	1.1108	0.0426	0.054
H(44A)	−0.2648	1.1609	0.0828	0.051
H(44B)	−0.3137	1.0936	0.1458	0.051
H(44C)	−0.3212	1.0554	0.0531	0.051
H(45A)	−0.0640	0.9965	0.3426	0.046
H(45B)	−0.1771	1.0096	0.3793	0.046
H(45C)	−0.1627	1.0771	0.3034	0.046
H(46A)	−0.2575	0.7867	0.2656	0.049
H(46B)	−0.2534	0.8385	0.3531	0.049
H(46C)	−0.1324	0.8125	0.3292	0.049
H(26)	−0.2513	0.6434	−0.0021	0.025

Table S33. Anisotropic displacement parameters (Å^2) for [PC$^{\bullet}$(sp^2)P]PdCl (**5**). The anisotropic displacement factor exponent takes the form: $-2\pi^2[h^2a^{*2}U_{11} + ...+ 2hka^*b^*U_{12}]$.

**atom**	U_{11}	U_{22}	U_{33}	U_{23}	U_{13}	U_{12}
Pd	0.0121(1)	0.0166(1)	0.0164(1)	−0.0046(1)	0.0004(1)	0.0038(1)
P(1)	0.0108(3)	0.0148(3)	0.0186(3)	−0.0024(2)	0.0019(2)	0.0011(2)
Cl	0.0214(3)	0.0189(3)	0.0192(3)	−0.0044(2)	−0.0005(2)	−0.0010(2)
P(2)	0.0135(3)	0.0178(3)	0.0185(3)	−0.0035(2)	0.0042(2)	0.0037(2)
C	0.0197(13)	0.0349(15)	0.0312(15)	−0.0150(12)	−0.0040(11)	0.0074(11)
C(11)	0.0166(12)	0.0153(11)	0.0244(13)	−0.0025(9)	−0.0001(10)	0.0038(9)
C(12)	0.0153(11)	0.0160(11)	0.0185(12)	−0.0002(9)	0.0044(9)	−0.0006(9)
C(13)	0.0146(11)	0.0166(11)	0.0234(13)	0.0008(10)	0.0042(10)	0.0032(9)
C(14)	0.0230(13)	0.0217(12)	0.0252(14)	−0.0031(10)	0.0133(11)	0.0040(10)
C(15)	0.0285(14)	0.0236(13)	0.0168(13)	−0.0002(10)	0.0054(11)	0.0002(11)
C(16)	0.0222(13)	0.0208(12)	0.0233(13)	−0.0032(10)	−0.0022(10)	0.0058(10)
C(21)	0.0187(12)	0.0198(12)	0.0141(12)	0.0003(9)	0.0010(9)	0.0030(10)
C(22)	0.0175(12)	0.0173(12)	0.0177(12)	0.0000(9)	0.0037(10)	0.0013(9)
C(23)	0.0166(12)	0.0287(14)	0.0288(14)	−0.0052(11)	0.0055(11)	0.0044(10)
C(24)	0.0169(13)	0.0412(16)	0.0303(15)	−0.0062(12)	0.0057(11)	−0.0022(11)
C(25)	0.0229(13)	0.0290(13)	0.0212(13)	−0.0029(11)	0.0038(11)	−0.0079(11)
C(31)	0.0137(11)	0.0173(11)	0.0259(13)	−0.0021(10)	0.0025(10)	−0.0010(9)
C(32)	0.0199(12)	0.0144(11)	0.0213(13)	−0.0029(9)	0.0051(10)	−0.0010(9)
C(33)	0.0249(14)	0.0207(13)	0.0430(16)	0.0038(11)	0.0148(12)	0.0008(11)
C(34)	0.0136(12)	0.0245(13)	0.0522(18)	0.0009(12)	0.0070(12)	0.0009(11)
C(35)	0.0342(15)	0.0222(13)	0.0202(13)	0.0013(10)	−0.0005(11)	−0.0023(11)
C(36)	0.0265(14)	0.0263(14)	0.0322(15)	−0.0011(11)	0.0141(12)	−0.0047(11)
C(41)	0.0239(13)	0.0199(12)	0.0251(14)	−0.0017(10)	0.0056(11)	−0.0008(10)
C(42)	0.0202(12)	0.0238(13)	0.0236(13)	−0.0034(10)	0.0105(10)	0.0025(10)
C(43)	0.0356(16)	0.0409(16)	0.0342(16)	0.0058(13)	0.0137(13)	−0.0040(13)
C(44)	0.0370(16)	0.0229(13)	0.0409(17)	0.0037(12)	0.0091(13)	0.0097(12)
C(45)	0.0360(15)	0.0338(15)	0.0267(14)	−0.0107(11)	0.0174(12)	−0.0043(12)
C(46)	0.0403(16)	0.0324(15)	0.0300(15)	−0.0006(12)	0.0160(13)	−0.0030(13)
C(26)	0.0289(14)	0.0171(12)	0.0157(12)	−0.0001(9)	0.0036(10)	0.0040(10)

Table S34. Distances [Å] for [PC•(sp^2)P]PdCl (**5**).

**atom – atom**	**distance**	**atom – atom**	**distance**
Pd – C	2.005(2)	Pd – P(1)	2.2751(6)
Pd – P(2)	2.2977(6)	Pd – Cl	2.3884(6)
P(1) – C(12)	1.811(2)	P(1) – C(31)	1.836(2)
P(1) – C(32)	1.840(2)	P(2) – C(22)	1.814(2)
P(2) – C(41)	1.841(2)	P(2) – C(42)	1.846(2)
C – C(21)	1.446(3)	C – C(11)	1.461(3)
C(11) – C(12)	1.411(3)	C(11) – C(16)	1.411(3)
C(12) – C(13)	1.389(3)	C(13) – C(14)	1.392(3)
C(13) – H(13)	0.9500	C(14) – C(15)	1.385(3)
C(14) – H(14)	0.9500	C(15) – C(16)	1.380(3)
C(15) – H(15)	0.9500	C(16) – H(16)	0.9500
C(21) – C(26)	1.409(3)	C(21) – C(22)	1.420(3)
C(22) – C(23)	1.385(3)	C(23) – C(24)	1.384(4)
C(23) – H(23)	0.9500	C(24) – C(25)	1.387(4)
C(24) – H(24)	0.9500	C(25) – C(26)	1.375(3)
C(25) – H(25)	0.9500	C(31) – C(34)	1.519(3)
C(31) – C(33)	1.534(3)	C(31) – H(31)	1.0000
C(32) – C(35)	1.522(3)	C(32) – C(36)	1.533(3)
C(32) – H(32)	1.0000	C(33) – H(33A)	0.9800
C(33) – H(33B)	0.9800	C(33) – H(33C)	0.9800
C(34) – H(34A)	0.9800	C(34) – H(34B)	0.9800
C(34) – H(34C)	0.9800	C(35) – H(35A)	0.9800
C(35) – H(35B)	0.9800	C(35) – H(35C)	0.9800
C(36) – H(36A)	0.9800	C(36) – H(36B)	0.9800
C(36) – H(36C)	0.9800	C(41) – C(44)	1.520(3)
C(41) – C(43)	1.526(3)	C(41) – H(41)	1.0000
C(42) – C(45)	1.526(3)	C(42) – C(46)	1.532(3)
C(42) – H(42)	1.0000	C(43) – H(43A)	0.9800
C(43) – H(43B)	0.9800	C(43) – H(43C)	0.9800
C(44) – H(44A)	0.9800	C(44) – H(44B)	0.9800
C(44) – H(44C)	0.9800	C(45) – H(45A)	0.9800
C(45) – H(45B)	0.9800	C(45) – H(45C)	0.9800
C(46) – H(46A)	0.9800	C(46) – H(46B)	0.9800
C(46) – H(46C)	0.9800	C(26) – H(26)	0.9500

Table S35. Angles [°] for [PC$^\bullet$(sp^2)P]PdCl (**5**).

**atom – atom – atom**	**angle**	**atom – atom – atom**	**angle**
C – Pd – P(1)	82.69(7)	C – Pd – P(2)	83.30(7)
P(1) – Pd – P(2)	165.30(2)	C – Pd – Cl	175.61(8)
P(1) – Pd – Cl	94.38(2)	P(2) – Pd – Cl	99.86(2)
C(12) – P(1) – C(31)	109.19(11)	C(12) – P(1) – C(32)	106.30(10)
C(31) – P(1) – C(32)	108.61(10)	C(12) – P(1) – Pd	102.35(7)
C(31) – P(1) – Pd	113.67(7)	C(32) – P(1) – Pd	116.13(8)
C(22) – P(2) – C(41)	108.61(11)	C(22) – P(2) – C(42)	104.53(11)
C(41) – P(2) – C(42)	106.27(11)	C(22) – P(2) – Pd	100.76(7)
C(41) – P(2) – Pd	118.10(8)	C(42) – P(2) – Pd	117.32(8)
C(21) – C – C(11)	122.2(2)	C(21) – C – Pd	118.87(17)
C(11) – C – Pd	118.78(17)	C(12) – C(11) – C(16)	116.9(2)
C(12) – C(11) – C	118.6(2)	C(16) – C(11) – C	124.5(2)
C(13) – C(12) – C(11)	121.5(2)	C(13) – C(12) – P(1)	125.98(18)
C(11) – C(12) – P(1)	112.51(16)	C(12) – C(13) – C(14)	119.8(2)
C(12) – C(13) – H(13)	120.1	C(14) – C(13) – H(13)	120.1
C(15) – C(14) – C(13)	119.5(2)	C(15) – C(14) – H(14)	120.3
C(13) – C(14) – H(14)	120.3	C(16) – C(15) – C(14)	120.9(2)
C(16) – C(15) – H(15)	119.5	C(14) – C(15) – H(15)	119.5
C(15) – C(16) – C(11)	121.0(2)	C(15) – C(16) – H(16)	119.5
C(11) – C(16) – H(16)	119.5	C(26) – C(21) – C(22)	117.1(2)
C(26) – C(21) – C	124.1(2)	C(22) – C(21) – C	118.6(2)
C(23) – C(22) – C(21)	120.4(2)	C(23) – C(22) – P(2)	125.73(18)
C(21) – C(22) – P(2)	113.60(16)	C(24) – C(23) – C(22)	121.1(2)
C(24) – C(23) – H(23)	119.5	C(22) – C(23) – H(23)	119.5
C(23) – C(24) – C(25)	119.2(2)	C(23) – C(24) – H(24)	120.4
C(25) – C(24) – H(24)	120.4	C(26) – C(25) – C(24)	120.7(2)
C(26) – C(25) – H(25)	119.7	C(24) – C(25) – H(25)	119.7
C(34) – C(31) – C(33)	111.4(2)	C(34) – C(31) – P(1)	117.41(16)
C(33) – C(31) – P(1)	108.10(16)	C(34) – C(31) – H(31)	106.4
C(33) – C(31) – H(31)	106.4	P(1) – C(31) – H(31)	106.4
C(35) – C(32) – C(36)	110.8(2)	C(35) – C(32) – P(1)	111.74(16)
C(36) – C(32) – P(1)	107.60(16)	C(35) – C(32) – H(32)	108.9
C(36) – C(32) – H(32)	108.9	P(1) – C(32) – H(32)	108.9
C(31) – C(33) – H(33A)	109.5	C(31) – C(33) – H(33B)	109.5
H(33A) – C(33) – H(33B)	109.5	C(31) – C(33) – H(33C)	109.5
H(33A) – C(33) – H(33C)	109.5	H(33B) – C(33) – H(33C)	109.5
C(31) – C(34) – H(34A)	109.5	C(31) – C(34) – H(34B)	109.5
H(34A) – C(34) – H(34B)	109.5	C(31) – C(34) – H(34C)	109.5
H(34A) – C(34) – H(34C)	109.5	H(34B) – C(34) – H(34C)	109.5
C(32) – C(35) – H(35A)	109.5	C(32) – C(35) – H(35B)	109.5
H(35A) – C(35) – H(35B)	109.5	C(32) – C(35) – H(35C)	109.5

Continued on next page

Table S35. – continued from previous page

atom – atom – atom	angle	atom – atom – atom	angle
H(35A) – C(35) – H(35C)	109.5	H(35B) – C(35) – H(35C)	109.5
C(32) – C(36) – H(36A)	109.5	C(32) – C(36) – H(36B)	109.5
H(36A) – C(36) – H(36B)	109.5	C(32) – C(36) – H(36C)	109.5
H(36A) – C(36) – H(36C)	109.5	H(36B) – C(36) – H(36C)	109.5
C(44) – C(41) – C(43)	111.4(2)	C(44) – C(41) – P(2)	114.78(17)
C(43) – C(41) – P(2)	109.55(17)	C(44) – C(41) – H(41)	106.9
C(43) – C(41) – H(41)	106.9	P(2) – C(41) – H(41)	106.9
C(45) – C(42) – C(46)	111.4(2)	C(45) – C(42) – P(2)	111.44(16)
C(46) – C(42) – P(2)	108.04(16)	C(45) – C(42) – H(42)	108.6
C(46) – C(42) – H(42)	108.6	P(2) – C(42) – H(42)	108.6
C(41) – C(43) – H(43A)	109.5	C(41) – C(43) – H(43B)	109.5
H(43A) – C(43) – H(43B)	109.5	C(41) – C(43) – H(43C)	109.5
H(43A) – C(43) – H(43C)	109.5	H(43B) – C(43) – H(43C)	109.5
C(41) – C(44) – H(44A)	109.5	C(41) – C(44) – H(44B)	109.5
H(44A) – C(44) – H(44B)	109.5	C(41) – C(44) – H(44C)	109.5
H(44A) – C(44) – H(44C)	109.5	H(44B) – C(44) – H(44C)	109.5
C(42) – C(45) – H(45A)	109.5	C(42) – C(45) – H(45B)	109.5
H(45A) – C(45) – H(45B)	109.5	C(42) – C(45) – H(45C)	109.5
H(45A) – C(45) – H(45C)	109.5	H(45B) – C(45) – H(45C)	109.5
C(42) – C(46) – H(46A)	109.5	C(42) – C(46) – H(46B)	109.5
H(46A) – C(46) – H(46B)	109.5	C(42) – C(46) – H(46C)	109.5
H(46A) – C(46) – H(46C)	109.5	H(46B) – C(46) – H(46C)	109.5
C(25) – C(26) – C(21)	121.5(2)	C(25) – C(26) – H(26)	119.3
C(21) – C(26) – H(26)	119.3		

8.5 Crystal data for [PC(CH$_2$)P]Pd(PMe$_3$) (6)

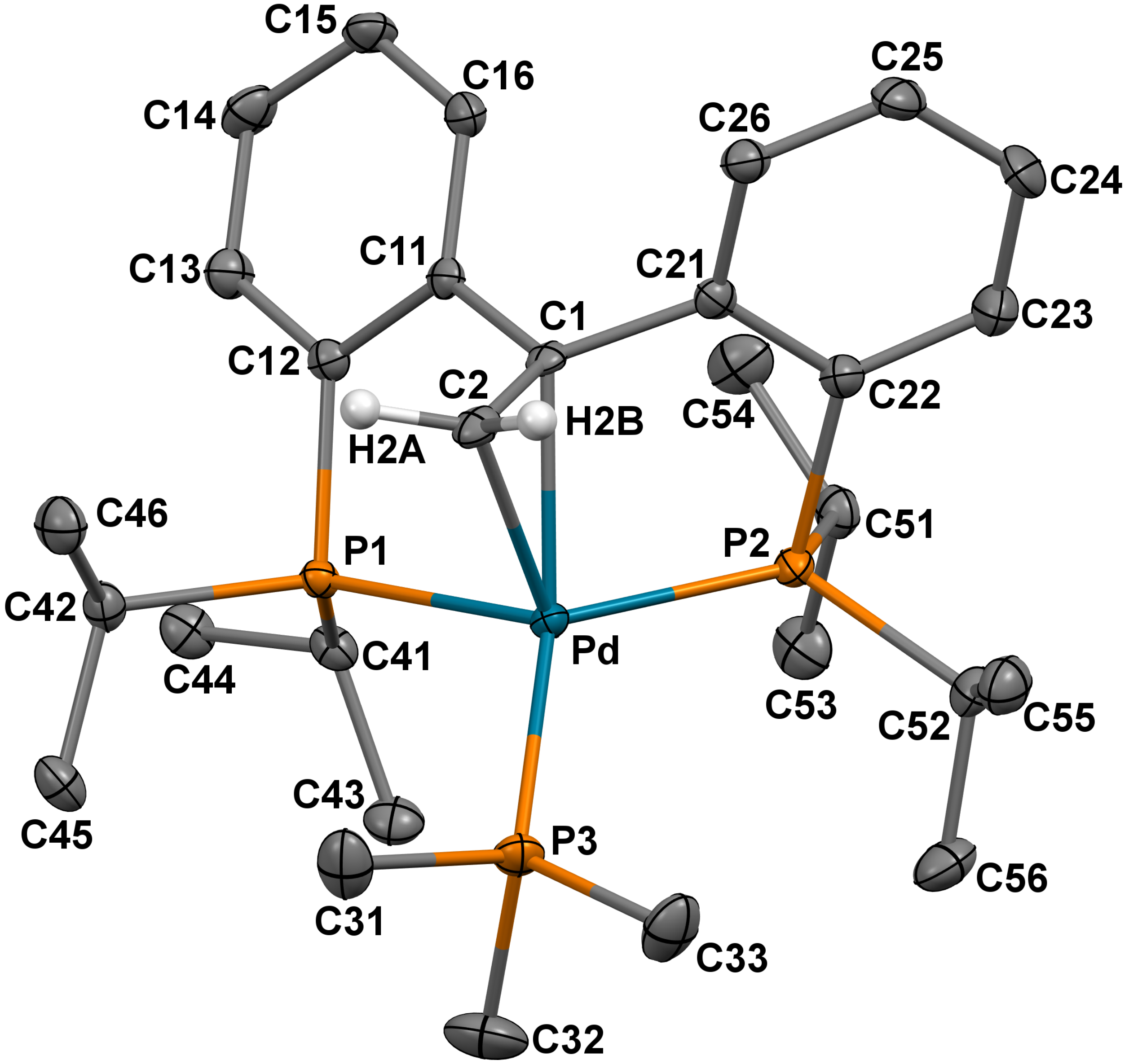

Figure S77. Thermal-ellipsoid representation of [PC(CH$_2$)P]Pd(PMe$_3$) (**6**) at 50% probability. Most hydrogen atoms were omitted for clarity.

Table S36. Crystal data and structure refinement for [PC(CH_2)P]Pd(PMe_3) (**6**).

Identification code:	cc252	
Empirical formula:	$C_{29}H_{47}P_3Pd$	
Formula weight:	594.98	
Temperature:	120(2) K	
Wavelength:	0.71073 Å	
Crystal system:	Monoclinic	
Space group:	$P2_1/n$	
Unit cell dimensions:	a = 10.3086(7) Å	α = 90°
	b = 24.0617(16) Å	β = 107.5771(18)°
	c = 12.5709(8) Å	γ = 90°
Volume:	2972.5(3) $Å^3$	
Z:	4	
Density (calculated):	1.329 g·cm^{-3}	
Absorption coefficient (μ):	0.801 mm^{-1}	
F(000):	1248	
Crystal size:	0.10 × 0.08 × 0.07 mm^3	
θ range for data collection:	1.69 to 25.00°	
Index ranges:	$-12 \leq h \leq 12$, $-28 \leq k \leq 28$, $-14 \leq l \leq 14$	
Reflections collected:	71407	
Independent reflections:	5233 [R_{int} = 0.0510]	
Completeness to θ = 25.00°:	100.0 %	
Absorption correction:	Semi-empirical from equivalents	
Max. and min. transmission:	0.7459 and 0.7038	
Refinement method:	Full-matrix least-squares on F^2	
Data / restraints / parameters:	5233 / 0 / 309	
Goodness-of-fit on F^2:	1.077	
Final R indices [I>2σ(I)]:	R_1 = 0.0220, wR_2 = 0.0495	
R indices (all data):	R_1 = 0.0257, wR_2 = 0.0512	
Largest diff. peak and hole:	0.407 and −0.292 e^-·$Å^{-3}$	

Table S37. Atomic coordinates and equivalent isotropic displacement parameters ($Å^2$) for $[PC(CH_2)P]Pd(PMe_3)$ (**6**). U(eq) is defined as one third of the trace of the orthogonalized U_{ij} tensor

atom	x	y	z	U(eq)
Pd	0.32963(2)	0.90247(1)	0.77305(1)	0.011(1)
C(1)	0.36707(19)	0.83153(8)	0.89193(15)	0.012(1)
P(1)	0.31818(5)	0.83411(2)	0.63170(4)	0.012(1)
C(2)	0.4938(2)	0.85538(8)	0.90181(16)	0.015(1)
P(2)	0.13457(5)	0.91283(2)	0.82673(4)	0.014(1)
P(3)	0.43807(5)	0.98108(2)	0.73622(5)	0.017(1)
C(12)	0.31246(19)	0.77155(8)	0.71434(16)	0.013(1)
C(11)	0.32562(19)	0.77739(8)	0.82891(16)	0.012(1)
C(13)	0.2883(2)	0.71872(8)	0.66585(17)	0.018(1)
C(14)	0.2678(2)	0.67300(8)	0.72535(18)	0.022(1)
C(15)	0.2720(2)	0.67916(8)	0.83545(18)	0.020(1)
C(16)	0.3021(2)	0.73050(8)	0.88673(17)	0.016(1)
C(22)	0.1882(2)	0.87742(8)	0.96274(16)	0.016(1)
C(21)	0.3031(2)	0.84337(8)	0.98238(16)	0.014(1)
C(23)	0.1256(2)	0.88392(9)	1.04636(18)	0.023(1)
C(24)	0.1791(2)	0.85897(9)	1.14957(18)	0.026(1)
C(25)	0.2963(2)	0.82744(8)	1.17066(17)	0.022(1)
C(26)	0.3582(2)	0.81956(8)	1.08791(16)	0.017(1)
C(31)	0.6124(2)	0.97107(9)	0.7320(2)	0.028(1)
C(32)	0.3622(3)	1.01382(10)	0.6000(2)	0.036(1)
C(33)	0.4656(2)	1.04266(9)	0.8262(2)	0.030(1)
C(41)	0.1569(2)	0.83120(8)	0.51212(16)	0.015(1)
C(42)	0.4498(2)	0.81260(8)	0.56574(16)	0.016(1)
C(43)	0.1218(2)	0.89011(9)	0.46509(17)	0.021(1)
C(44)	0.1475(2)	0.79022(9)	0.41706(17)	0.019(1)
C(45)	0.4695(2)	0.85690(9)	0.48451(17)	0.020(1)
C(46)	0.5858(2)	0.80074(9)	0.65435(18)	0.022(1)
C(51)	−0.0360(2)	0.87909(8)	0.76584(17)	0.018(1)
C(52)	0.0845(2)	0.98315(8)	0.86488(17)	0.019(1)
C(53)	−0.1084(2)	0.89886(9)	0.64709(18)	0.025(1)
C(54)	−0.0206(2)	0.81582(9)	0.7674(2)	0.026(1)
C(55)	0.1983(2)	1.00752(9)	0.96142(18)	0.025(1)
C(56)	0.0548(2)	1.02229(9)	0.76451(19)	0.027(1)
H(2A)	0.5573	0.8331	0.8736	0.018
H(2B)	0.5387	0.8751	0.9724	0.018
H(13)	0.2860	0.7142	0.5902	0.021
H(14)	0.2509	0.6376	0.6906	0.026
H(15)	0.2542	0.6482	0.8760	0.023
H(16)	0.3070	0.7341	0.9631	0.020

Continued on next page

Table S37. – continued from previous page

atom	x	y	x	U(eq)
H(23)	0.0454	0.9057	1.0322	0.028
H(24)	0.1355	0.8635	1.2057	0.031
H(25)	0.3347	0.8111	1.2421	0.026
H(26)	0.4386	0.7978	1.1031	0.020
H(31A)	0.6124	0.9447	0.6726	0.042
H(31B)	0.6687	0.9564	0.8038	0.042
H(31C)	0.6495	1.0067	0.7171	0.042
H(32A)	0.3604	0.9871	0.5407	0.053
H(32B)	0.4164	1.0463	0.5929	0.053
H(32C)	0.2691	1.0256	0.5935	0.053
H(33A)	0.5180	1.0703	0.7992	0.045
H(33B)	0.5161	1.0321	0.9028	0.045
H(33C)	0.3776	1.0585	0.8248	0.045
H(41)	0.0833	0.8204	0.5449	0.018
H(42)	0.4183	0.7776	0.5228	0.019
H(43A)	0.1855	0.9012	0.4248	0.031
H(43B)	0.1287	0.9161	0.5265	0.031
H(43C)	0.0288	0.8906	0.4139	0.031
H(44A)	0.0606	0.7953	0.3586	0.029
H(44B)	0.1535	0.7521	0.4457	0.029
H(44C)	0.2226	0.7970	0.3860	0.029
H(45A)	0.5402	0.8447	0.4522	0.030
H(45B)	0.4971	0.8920	0.5246	0.030
H(45C)	0.3838	0.8623	0.4248	0.030
H(46A)	0.6524	0.7883	0.6179	0.034
H(46B)	0.5732	0.7716	0.7049	0.034
H(46C)	0.6188	0.8347	0.6970	0.034
H(51)	−0.0942	0.8891	0.8138	0.022
H(52)	0.0006	0.9794	0.8885	0.023
H(53A)	−0.0485	0.8936	0.6005	0.037
H(53B)	−0.1311	0.9384	0.6484	0.037
H(53C)	−0.1921	0.8773	0.6163	0.037
H(54A)	−0.1100	0.7987	0.7347	0.039
H(54B)	0.0180	0.8031	0.8446	0.039
H(54C)	0.0400	0.8052	0.7240	0.039
H(55A)	0.2848	1.0049	0.9445	0.037
H(55B)	0.2046	0.9867	1.0298	0.037
H(55C)	0.1784	1.0466	0.9719	0.037
H(56A)	0.1338	1.0235	0.7366	0.040
H(56B)	0.0362	1.0597	0.7872	0.040
H(56C)	−0.0246	1.0087	0.7055	0.040

Table S38. Anisotropic displacement parameters ($Å^2$) for [PC(CH_2)P]Pd(PMe_3) (**6**). The anisotropic displacement factor exponent takes the form: $-2\pi^2[h^2a^{*2}U_{11} + ... + 2hka^*b^*U_{12}]$.

**atom**	U_{11}	U_{22}	U_{33}	U_{23}	U_{13}	U_{12}
Pd	0.0127(1)	0.0089(1)	0.0116(1)	0.0004(1)	0.0042(1)	0.0000(1)
C(1)	0.0138(10)	0.0102(9)	0.0094(9)	0.0028(7)	0.0009(8)	0.0045(8)
P(1)	0.0133(3)	0.0108(2)	0.0107(2)	−0.0003(2)	0.0040(2)	−0.0014(2)
C(2)	0.0153(10)	0.0137(10)	0.0132(10)	0.0020(8)	0.0008(8)	0.0039(8)
P(2)	0.0139(3)	0.0143(3)	0.0131(3)	0.0021(2)	0.0050(2)	0.0033(2)
P(3)	0.0195(3)	0.0117(2)	0.0213(3)	0.0006(2)	0.0078(2)	−0.0025(2)
C(12)	0.0108(10)	0.0133(10)	0.0148(10)	0.0005(8)	0.0037(8)	−0.0007(8)
C(11)	0.0093(9)	0.0130(9)	0.0154(10)	0.0019(8)	0.0049(8)	0.0022(8)
C(13)	0.0207(11)	0.0169(10)	0.0150(10)	−0.0020(8)	0.0047(9)	−0.0014(8)
C(14)	0.0231(12)	0.0119(10)	0.0289(12)	−0.0033(9)	0.0070(10)	−0.0027(9)
C(15)	0.0204(11)	0.0132(10)	0.0272(12)	0.0046(9)	0.0108(9)	−0.0018(8)
C(16)	0.0168(10)	0.0168(10)	0.0186(11)	0.0030(8)	0.0092(9)	0.0029(8)
C(22)	0.0203(11)	0.0135(10)	0.0157(10)	0.0011(8)	0.0072(9)	0.0011(8)
C(21)	0.0175(10)	0.0113(9)	0.0136(10)	−0.0017(8)	0.0056(8)	−0.0020(8)
C(23)	0.0264(12)	0.0231(11)	0.0240(12)	0.0040(9)	0.0131(10)	0.0084(10)
C(24)	0.0393(14)	0.0254(12)	0.0189(11)	0.0030(9)	0.0187(10)	0.0050(10)
C(25)	0.0338(13)	0.0179(11)	0.0140(11)	0.0038(8)	0.0085(9)	0.0023(9)
C(26)	0.0208(11)	0.0142(10)	0.0158(10)	0.0012(8)	0.0048(9)	0.0005(8)
C(31)	0.0246(12)	0.0254(12)	0.0377(14)	−0.0105(10)	0.0160(11)	−0.0097(10)
C(32)	0.0415(15)	0.0307(13)	0.0347(14)	0.0151(11)	0.0118(12)	−0.0067(11)
C(33)	0.0287(13)	0.0162(11)	0.0465(15)	−0.0092(10)	0.0146(11)	−0.0041(10)
C(41)	0.0133(10)	0.0188(10)	0.0130(10)	0.0011(8)	0.0039(8)	−0.0016(8)
C(42)	0.0166(10)	0.0166(10)	0.0172(10)	−0.0030(8)	0.0078(9)	−0.0014(8)
C(43)	0.0203(11)	0.0228(11)	0.0164(11)	0.0042(9)	0.0013(9)	0.0011(9)
C(44)	0.0187(11)	0.0225(11)	0.0148(10)	−0.0022(9)	0.0023(9)	−0.0041(9)
C(45)	0.0211(11)	0.0257(11)	0.0166(11)	−0.0011(9)	0.0094(9)	−0.0051(9)
C(46)	0.0170(11)	0.0258(12)	0.0260(12)	0.0017(9)	0.0091(9)	0.0009(9)
C(51)	0.0143(10)	0.0219(11)	0.0203(11)	0.0007(9)	0.0072(9)	0.0012(8)
C(52)	0.0197(11)	0.0177(10)	0.0217(11)	0.0006(9)	0.0083(9)	0.0061(9)
C(53)	0.0150(11)	0.0318(13)	0.0254(12)	0.0017(10)	0.0034(9)	−0.0002(9)
C(54)	0.0175(11)	0.0228(12)	0.0370(14)	0.0002(10)	0.0056(10)	−0.0038(9)
C(55)	0.0303(13)	0.0220(11)	0.0224(12)	−0.0031(9)	0.0081(10)	0.0033(10)
C(56)	0.0308(13)	0.0177(11)	0.0281(13)	0.0034(9)	0.0038(10)	0.0071(10)

Table S39. Distances [Å] for [PC(CH_2)P]Pd(PMe_3) (**6**).

**atom – atom**	**distance**	**atom – atom**	**distance**
Pd – C(1)	2.2242(18)	Pd – C(2)	2.2613(19)
Pd – P(3)	2.3140(5)	Pd – P(2)	2.3196(5)
Pd – P(1)	2.3978(5)	C(1) – C(2)	1.398(3)
C(1) – C(21)	1.504(3)	C(1) – C(11)	1.517(3)
P(1) – C(12)	1.8402(19)	P(1) – C(42)	1.864(2)
P(1) – C(41)	1.876(2)	C(2) – H(2A)	0.9900
C(2) – H(2B)	0.9900	P(2) – C(22)	1.839(2)
P(2) – C(52)	1.873(2)	P(2) – C(51)	1.877(2)
P(3) – C(31)	1.830(2)	P(3) – C(32)	1.832(2)
P(3) – C(33)	1.834(2)	C(12) – C(13)	1.399(3)
C(12) – C(11)	1.412(3)	C(11) – C(16)	1.402(3)
C(13) – C(14)	1.382(3)	C(13) – H(13)	0.9500
C(14) – C(15)	1.380(3)	C(14) – H(14)	0.9500
C(15) – C(16)	1.385(3)	C(15) – H(15)	0.9500
C(16) – H(16)	0.9500	C(22) – C(23)	1.398(3)
C(22) – C(21)	1.399(3)	C(21) – C(26)	1.398(3)
C(23) – C(24)	1.384(3)	C(23) – H(23)	0.9500
C(24) – C(25)	1.383(3)	C(24) – H(24)	0.9500
C(25) – C(26)	1.387(3)	C(25) – H(25)	0.9500
C(26) – H(26)	0.9500	C(31) – H(31A)	0.9800
C(31) – H(31B)	0.9800	C(31) – H(31C)	0.9800
C(32) – H(32A)	0.9800	C(32) – H(32B)	0.9800
C(32) – H(32C)	0.9800	C(33) – H(33A)	0.9800
C(33) – H(33B)	0.9800	C(33) – H(33C)	0.9800
C(41) – C(44)	1.530(3)	C(41) – C(43)	1.536(3)
C(41) – H(41)	1.0000	C(42) – C(46)	1.531(3)
C(42) – C(45)	1.532(3)	C(42) – H(42)	1.0000
C(43) – H(43A)	0.9800	C(43) – H(43B)	0.9800
C(43) – H(43C)	0.9800	C(44) – H(44A)	0.9800
C(44) – H(44B)	0.9800	C(44) – H(44C)	0.9800
C(45) – H(45A)	0.9800	C(45) – H(45B)	0.9800
C(45) – H(45C)	0.9800	C(46) – H(46A)	0.9800
C(46) – H(46B)	0.9800	C(46) – H(46C)	0.9800
C(51) – C(53)	1.529(3)	C(51) – C(54)	1.530(3)
C(51) – H(51)	1.0000	C(52) – C(55)	1.527(3)
C(52) – C(56)	1.529(3)	C(52) – H(52)	1.0000
C(53) – H(53A)	0.9800	C(53) – H(53B)	0.9800
C(53) – H(53C)	0.9800	C(54) – H(54A)	0.9800
C(54) – H(54B)	0.9800	C(54) – H(54C)	0.9800
C(55) – H(55A)	0.9800	C(55) – H(55B)	0.9800
C(56) – H(56B)	0.9800	C(56) – H(56C)	0.9800

Table S40. Angles [°] for [PC(CH_2)P]Pd(PMe_3) (**6**).

atom – atom – atom	angle	atom – atom – atom	angle
C(1) – Pd – C(2)	36.30(7)	C(1) – Pd – P(3)	139.94(5)
C(2) – Pd – P(3)	104.50(5)	C(1) – Pd – P(2)	82.92(5)
C(2) – Pd – P(2)	111.89(5)	P(3) – Pd – P(2)	118.946(19)
C(1) – Pd – P(1)	86.06(5)	C(2) – Pd – P(1)	92.61(5)
P(3) – Pd – P(1)	109.332(19)	P(2) – Pd – P(1)	115.868(19)
C(2) – C(1) – C(21)	117.82(17)	C(2) – C(1) – C(11)	120.48(17)
C(21) – C(1) – C(11)	116.01(16)	C(2) – C(1) – Pd	73.29(11)
C(21) – C(1) – Pd	109.62(12)	C(11) – C(1) – Pd	110.10(12)
C(12) – P(1) – C(42)	100.11(9)	C(12) – P(1) – C(41)	104.48(9)
C(42) – P(1) – C(41)	102.78(9)	C(12) – P(1) – Pd	98.37(6)
C(42) – P(1) – Pd	129.83(7)	C(41) – P(1) – Pd	116.92(6)
C(1) – C(2) – Pd	70.40(11)	C(1) – C(2) – H(2A)	116.6
Pd – C(2) – H(2A)	116.6	C(1) – C(2) – H(2B)	116.6
Pd – C(2) – H(2B)	116.6	H(2A) – C(2) – H(2B)	113.6
C(22) – P(2) – C(52)	102.07(9)	C(22) – P(2) – C(51)	99.22(9)
C(52) – P(2) – C(51)	101.46(9)	C(22) – P(2) – Pd	100.20(7)
C(52) – P(2) – Pd	120.10(7)	C(51) – P(2) – Pd	128.50(7)
C(31) – P(3) – C(32)	100.09(12)	C(31) – P(3) – C(33)	98.85(11)
C(32) – P(3) – C(33)	99.91(12)	C(31) – P(3) – Pd	115.87(8)
C(32) – P(3) – Pd	116.29(8)	C(33) – P(3) – Pd	122.09(8)
C(13) – C(12) – C(11)	118.97(18)	C(13) – C(12) – P(1)	121.94(15)
C(11) – C(12) – P(1)	119.00(14)	C(16) – C(11) – C(12)	118.04(18)
C(16) – C(11) – C(1)	119.06(17)	C(12) – C(11) – C(1)	122.89(17)
C(14) – C(13) – C(12)	121.57(19)	C(14) – C(13) – H(13)	119.2
C(12) – C(13) – H(13)	119.2	C(15) – C(14) – C(13)	119.60(19)
C(15) – C(14) – H(14)	120.2	C(13) – C(14) – H(14)	120.2
C(14) – C(15) – C(16)	119.83(19)	C(14) – C(15) – H(15)	120.1
C(16) – C(15) – H(15)	120.1	C(15) – C(16) – C(11)	121.75(19)
C(15) – C(16) – H(16)	119.1	C(11) – C(16) – H(16)	119.1
C(23) – C(22) – C(21)	119.39(18)	C(23) – C(22) – P(2)	124.73(16)
C(21) – C(22) – P(2)	115.84(15)	C(26) – C(21) – C(22)	119.14(18)
C(26) – C(21) – C(1)	119.62(17)	C(22) – C(21) – C(1)	121.24(17)
C(24) – C(23) – C(22)	120.8(2)	C(24) – C(23) – H(23)	119.6
C(22) – C(23) – H(23)	119.6	C(25) – C(24) – C(23)	119.7(2)
C(25) – C(24) – H(24)	120.2	C(23) – C(24) – H(24)	120.2
C(24) – C(25) – C(26)	120.26(19)	C(24) – C(25) – H(25)	119.9
C(26) – C(25) – H(25)	119.9	C(25) – C(26) – C(21)	120.55(19)
C(25) – C(26) – H(26)	119.7	C(21) – C(26) – H(26)	119.7
P(3) – C(31) – H(31A)	109.5	P(3) – C(31) – H(31B)	109.5
H(31A) – C(31) – H(31B)	109.5	P(3) – C(31) – H(31C)	109.5
H(31A) – C(31) – H(31C)	109.5	H(31B) – C(31) – H(31C)	109.5

Continued on next page

Table S40. – continued from previous page

atom – atom – atom	angle	atom – atom – atom	angle
P(3) – C(32) – H(32A)	109.5	P(3) – C(32) – H(32B)	109.5
H(32A) – C(32) – H(32B)	109.5	P(3) – C(32) – H(32C)	109.5
H(32A) – C(32) – H(32C)	109.5	H(32B) – C(32) – H(32C)	109.5
P(3) – C(33) – H(33A)	109.5	P(3) – C(33) – H(33B)	109.5
H(33A) – C(33) – H(33B)	109.5	P(3) – C(33) – H(33C)	109.5
H(33A) – C(33) – H(33C)	109.5	H(33B) – C(33) – H(33C)	109.5
C(44) – C(41) – C(43)	110.01(16)	C(44) – C(41) – P(1)	118.77(14)
C(43) – C(41) – P(1)	108.81(13)	C(44) – C(41) – H(41)	106.2
C(43) – C(41) – H(41)	106.2	P(1) – C(41) – H(41)	106.2
C(46) – C(42) – C(45)	109.76(17)	C(46) – C(42) – P(1)	110.95(14)
C(45) – C(42) – P(1)	111.35(14)	C(46) – C(42) – H(42)	108.2
C(45) – C(42) – H(42)	108.2	P(1) – C(42) – H(42)	108.2
C(41) – C(43) – H(43A)	109.5	C(41) – C(43) – H(43B)	109.5
H(43A) – C(43) – H(43B)	109.5	C(41) – C(43) – H(43C)	109.5
H(43A) – C(43) – H(43C)	109.5	H(43B) – C(43) – H(43C)	109.5
C(41) – C(44) – H(44A)	109.5	C(41) – C(44) – H(44B)	109.5
H(44A) – C(44) – H(44B)	109.5	C(41) – C(44) – H(44C)	109.5
H(44A) – C(44) – H(44C)	109.5	H(44B) – C(44) – H(44C)	109.5
C(42) – C(45) – H(45A)	109.5	C(42) – C(45) – H(45B)	109.5
H(45A) – C(45) – H(45B)	109.5	C(42) – C(45) – H(45C)	109.5
H(45A) – C(45) – H(45C)	109.5	H(45B) – C(45) – H(45C)	109.5
C(42) – C(46) – H(46A)	109.5	C(42) – C(46) – H(46B)	109.5
H(46A) – C(46) – H(46B)	109.5	C(42) – C(46) – H(46C)	109.5
H(46A) – C(46) – H(46C)	109.5	H(46B) – C(46) – H(46C)	109.5
C(53) – C(51) – C(54)	109.90(18)	C(53) – C(51) – P(2)	112.61(14)
C(54) – C(51) – P(2)	110.14(14)	C(53) – C(51) – H(51)	108.0
C(54) – C(51) – H(51)	108.0	P(2) – C(51) – H(51)	108.0
C(55) – C(52) – C(56)	109.49(18)	C(55) – C(52) – P(2)	110.22(14)
C(56) – C(52) – P(2)	110.30(14)	C(55) – C(52) – H(52)	108.9
C(56) – C(52) – H(52)	108.9	P(2) – C(52) – H(52)	108.9
C(51) – C(53) – H(53A)	109.5	C(51) – C(53) – H(53B)	109.5
H(53A) – C(53) – H(53B)	109.5	C(51) – C(53) – H(53C)	109.5
H(53A) – C(53) – H(53C)	109.5	H(53B) – C(53) – H(53C)	109.5
C(51) – C(54) – H(54A)	109.5	C(51) – C(54) – H(54B)	109.5
H(54A) – C(54) – H(54B)	109.5	C(51) – C(54) – H(54C)	109.5
H(54A) – C(54) – H(54C)	109.5	H(54B) – C(54) – H(54C)	109.5
C(52) – C(55) – H(55A)	109.5	C(52) – C(55) – H(55B)	109.5
H(55A) – C(55) – H(55B)	109.5	C(52) – C(55) – H(55C)	109.5
H(55A) – C(55) – H(55C)	109.5	H(55B) – C(55) – H(55C)	109.5
C(52) – C(56) – H(56A)	109.5	C(52) – C(56) – H(56B)	109.5
H(56A) – C(56) – H(56B)	109.5	C(52) – C(56) – H(56C)	109.5
H(56A) – C(56) – H(56C)	109.5	H(56B) – C(56) – H(56C)	109.5

8.6 Crystal data for [PC(CH_3)HP]PdCl_2 (8)

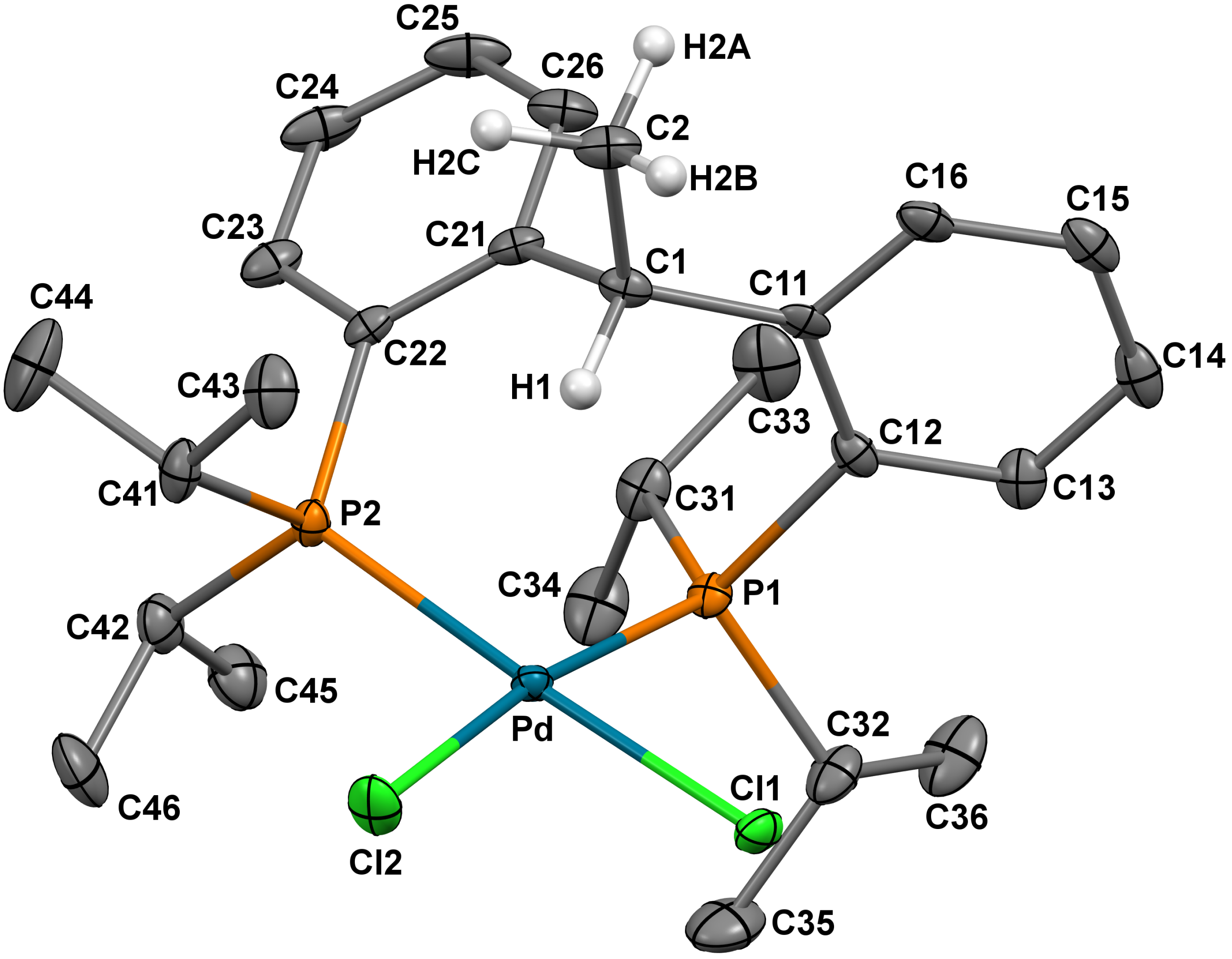

Figure S78. Thermal-ellipsoid representation of [PC(CH_3)HP]PdCl_2 (**8**) at 50% probability. Most hydrogen atoms were omitted for clarity.

Table S41. Crystal data and structure refinement for [PC(CH_3)HP]PdCl_2 (**8**).

Identification code:	cc251	
Empirical formula:	$C_{26}H_{40}Cl_2P_2Pd$	
Formula weight:	591.82	
Temperature:	120(2) K	
Wavelength:	0.71073 Å	
Crystal system:	Monoclinic	
Space group:	$C2/c$	
Unit cell dimensions:	a = 13.0569(14) Å	α = 90°
	b = 16.8080(14) Å	β = 93.209(2)°
	c = 31.261(3) Å	γ = 90°
Volume:	6849.8(11) $Å^3$	
Z:	8	
Density (calculated):	1.148 g·cm^{-3}	
Absorption coefficient (μ):	0.801 mm^{-1}	
F(000):	2448	
Crystal size:	0.11 × 0.09 × 0.08 mm^3	
θ range for data collection:	2.11 to 25.00°	
Index ranges:	$-15 \leq h \leq 15$, $-19 \leq k \leq 19$, $-37 \leq l \leq 37$	
Reflections collected:	38696	
Independent reflections:	6002 [R_{int} = 0.0536]	
Completeness to θ = 25.00°:	99.4 %	
Absorption correction:	Semi-empirical from equivalents	
Max. and min. transmission:	0.7455 and 0.6774	
Refinement method:	Full-matrix least-squares on F^2	
Data / restraints / parameters:	6002 / 0 / 289	
Goodness-of-fit on F^2:	1.051	
Final R indices [I>2σ(I)]:	R_1 = 0.0372, wR_2 = 0.0751	
R indices (all data):	R_1 = 0.0457, wR_2 = 0.0779	
Largest diff. peak and hole:	0.596 and −0.503 e^-·$Å^{-3}$	

Table S42. Atomic coordinates and equivalent isotropic displacement parameters (Å^2) for [PC(CH_3)HP]PdCl_2 (**8**). U(eq) is defined as one third of the trace of the orthogonalized U_{ij} tensor

atom	x	y	z	U(eq)
Pd	0.40321(2)	0.33460(2)	0.89095(1)	0.016(1)
C(1)	0.4436(2)	0.20985(19)	0.97301(10)	0.019(1)
Cl(1)	0.22633(6)	0.36860(5)	0.88942(3)	0.024(1)
P(1)	0.34981(6)	0.20549(5)	0.87347(3)	0.019(1)
C(2)	0.4730(3)	0.2126(2)	1.02138(11)	0.028(1)
Cl(2)	0.43656(7)	0.47011(5)	0.90297(3)	0.033(1)
P(2)	0.57394(6)	0.31313(5)	0.90471(3)	0.018(1)
C(12)	0.2913(2)	0.1660(2)	0.92139(10)	0.021(1)
C(11)	0.3403(2)	0.16972(19)	0.96279(10)	0.018(1)
C(13)	0.1941(3)	0.1325(2)	0.91653(12)	0.025(1)
C(14)	0.1457(3)	0.0990(2)	0.95070(13)	0.030(1)
C(15)	0.1935(3)	0.1008(2)	0.99046(12)	0.029(1)
C(16)	0.2885(3)	0.1367(2)	0.99630(12)	0.025(1)
C(21)	0.5330(2)	0.1720(2)	0.95103(10)	0.020(1)
C(22)	0.6007(2)	0.2127(2)	0.92514(10)	0.020(1)
C(23)	0.6866(3)	0.1721(2)	0.91099(11)	0.028(1)
C(24)	0.7032(3)	0.0922(2)	0.92015(12)	0.035(1)
C(25)	0.6343(3)	0.0518(2)	0.94356(12)	0.038(1)
C(26)	0.5517(3)	0.0911(2)	0.95948(12)	0.031(1)
C(31)	0.4364(3)	0.1245(2)	0.85593(11)	0.027(1)
C(32)	0.2437(3)	0.2123(2)	0.83131(11)	0.031(1)
C(33)	0.4059(3)	0.0416(2)	0.87072(15)	0.042(1)
C(34)	0.4513(3)	0.1229(3)	0.80729(12)	0.041(1)
C(35)	0.2710(3)	0.2704(3)	0.79609(13)	0.048(1)
C(36)	0.1992(3)	0.1350(3)	0.81069(14)	0.047(1)
C(41)	0.6389(3)	0.3820(2)	0.94404(12)	0.028(1)
C(42)	0.6503(3)	0.3286(2)	0.85717(12)	0.030(1)
C(43)	0.5898(3)	0.3863(2)	0.98697(13)	0.036(1)
C(44)	0.7534(3)	0.3639(3)	0.95072(16)	0.053(1)
C(45)	0.6133(3)	0.2771(3)	0.81971(13)	0.038(1)
C(46)	0.6492(3)	0.4162(3)	0.84395(15)	0.045(1)
H(1)	0.4376	0.2661	0.9627	0.022
H(2A)	0.4773	0.1582	1.0327	0.042
H(2B)	0.4208	0.2423	1.0361	0.042
H(2C)	0.5396	0.2389	1.0262	0.042
H(13)	0.1596	0.1323	0.8890	0.030
H(14)	0.0799	0.0752	0.9463	0.036
H(15)	0.1619	0.0776	1.0141	0.034
H(16)	0.3199	0.1390	1.0244	0.030

Continued on next page

Table S42. – continued from previous page

atom	x	y	x	U(eq)
H(23)	0.7344	0.2000	0.8948	0.033
H(24)	0.7617	0.0657	0.9103	0.042
H(25)	0.6434	−0.0035	0.9489	0.045
H(26)	0.5063	0.0626	0.9766	0.037
H(31)	0.5055	0.1356	0.8701	0.033
H(32)	0.1853	0.2376	0.8456	0.037
H(33A)	0.3414	0.0257	0.8555	0.063
H(33B)	0.3969	0.0424	0.9016	0.063
H(33C)	0.4598	0.0035	0.8645	0.063
H(34A)	0.3907	0.0994	0.7923	0.061
H(34B)	0.5119	0.0910	0.8017	0.061
H(34C)	0.4609	0.1773	0.7970	0.061
H(35A)	0.3250	0.2473	0.7794	0.071
H(35B)	0.2954	0.3205	0.8091	0.071
H(35C)	0.2101	0.2807	0.7772	0.071
H(36A)	0.1284	0.1444	0.7999	0.070
H(36B)	0.2004	0.0926	0.8322	0.070
H(36C)	0.2407	0.1191	0.7869	0.070
H(41)	0.6333	0.4365	0.9312	0.034
H(42)	0.7229	0.3135	0.8652	0.036
H(43A)	0.5156	0.3939	0.9822	0.054
H(43B)	0.6190	0.4310	1.0036	0.054
H(43C)	0.6031	0.3366	1.0028	0.054
H(44A)	0.7854	0.3663	0.9231	0.079
H(44B)	0.7626	0.3105	0.9630	0.079
H(44C)	0.7856	0.4032	0.9703	0.079
H(45A)	0.5437	0.2933	0.8099	0.058
H(45B)	0.6127	0.2212	0.8287	0.058
H(45C)	0.6594	0.2835	0.7963	0.058
H(46A)	0.6847	0.4224	0.8174	0.067
H(46B)	0.6840	0.4481	0.8666	0.067
H(46C)	0.5781	0.4344	0.8394	0.067

Table S43. Anisotropic displacement parameters (Å^2) for [PC(CH_3)HP]$PdCl_2$ (**8**). The anisotropic displacement factor exponent takes the form: $-2\pi^2[h^2a^{*2}U_{11} + ...+ 2hka^*b^*U_{12}]$.

atom	U_{11}	U_{22}	U_{33}	U_{23}	U_{13}	U_{12}
Pd	0.0157(1)	0.0149(1)	0.0176(1)	0.0023(1)	0.0019(1)	0.0028(1)
C(1)	0.0270(18)	0.0130(17)	0.0159(17)	−0.0017(13)	0.0024(13)	0.0041(13)
Cl(1)	0.0175(4)	0.0299(5)	0.0260(4)	0.0048(4)	0.0022(3)	0.0076(3)
P(1)	0.0197(4)	0.0217(5)	0.0142(4)	−0.0034(4)	−0.0001(3)	−0.0011(3)
C(2)	0.038(2)	0.025(2)	0.0199(18)	0.0002(15)	−0.0021(15)	0.0108(16)
Cl(2)	0.0303(5)	0.0142(4)	0.0546(6)	0.0013(4)	0.0082(4)	0.0024(3)
P(2)	0.0145(4)	0.0176(5)	0.0230(5)	−0.0019(3)	0.0026(3)	−0.0001(3)
C(12)	0.0266(17)	0.0147(17)	0.0233(17)	−0.0004(15)	0.0066(14)	−0.0004(14)
C(11)	0.0268(17)	0.0077(16)	0.0206(17)	0.0017(14)	0.0042(13)	0.0068(14)
C(13)	0.0266(19)	0.0198(19)	0.030(2)	−0.0093(15)	0.0050(15)	−0.0013(15)
C(14)	0.032(2)	0.0168(19)	0.041(2)	−0.0073(16)	0.0157(18)	−0.0044(15)
C(15)	0.037(2)	0.0169(19)	0.034(2)	0.0023(16)	0.0181(17)	0.0047(15)
C(16)	0.0315(19)	0.0167(18)	0.0266(19)	0.0055(15)	0.0053(15)	0.0069(15)
C(21)	0.0234(17)	0.0202(18)	0.0163(16)	−0.0012(14)	−0.0045(13)	0.0075(14)
C(22)	0.0177(16)	0.0224(19)	0.0185(17)	−0.0042(14)	−0.0053(13)	0.0041(14)
C(23)	0.0276(18)	0.036(2)	0.0200(18)	−0.0032(16)	−0.0029(14)	0.0099(17)
C(24)	0.041(2)	0.041(3)	0.023(2)	−0.0052(17)	−0.0014(17)	0.0274(19)
C(25)	0.060(3)	0.026(2)	0.027(2)	−0.0012(17)	−0.0025(19)	0.0245(19)
C(26)	0.045(2)	0.024(2)	0.0227(19)	0.0046(16)	0.0004(17)	0.0094(17)
C(31)	0.0270(19)	0.027(2)	0.028(2)	−0.0134(16)	0.0012(15)	0.0003(15)
C(32)	0.0252(19)	0.047(3)	0.0201(19)	−0.0084(17)	−0.0036(15)	0.0007(17)
C(33)	0.050(3)	0.023(2)	0.055(3)	−0.016(2)	0.011(2)	−0.0005(18)
C(34)	0.038(2)	0.058(3)	0.027(2)	−0.022(2)	0.0052(17)	−0.003(2)
C(35)	0.051(3)	0.070(3)	0.021(2)	0.006(2)	−0.0038(18)	0.011(2)
C(36)	0.038(2)	0.068(3)	0.033(2)	−0.026(2)	−0.0050(18)	−0.002(2)
C(41)	0.0199(18)	0.024(2)	0.040(2)	−0.0087(17)	0.0023(15)	−0.0051(15)
C(42)	0.0240(18)	0.034(2)	0.032(2)	0.0005(17)	0.0098(15)	−0.0003(16)
C(43)	0.036(2)	0.034(2)	0.038(2)	−0.0156(18)	0.0046(18)	−0.0092(17)
C(44)	0.022(2)	0.070(3)	0.065(3)	−0.030(3)	−0.004(2)	−0.004(2)
C(45)	0.041(2)	0.045(3)	0.031(2)	0.0004(19)	0.0104(18)	−0.0046(19)
C(46)	0.046(3)	0.039(3)	0.052(3)	0.007(2)	0.026(2)	−0.010(2)

Table S44. Distances [Å] for [PC(CH_3)HP]PdCl_2 (**8**).

**atom – atom**	**distance**	**atom – atom**	**distance**
Pd – P(2)	2.2754(9)	Pd – P(1)	2.3348(9)
Pd – Cl(2)	2.3452(9)	Pd – Cl(1)	2.3771(8)
C(1) – C(11)	1.526(5)	C(1) – C(21)	1.527(4)
C(1) – C(2)	1.540(5)	C(1) – H(1)	1.0000
P(1) – C(12)	1.843(3)	P(1) – C(32)	1.862(4)
P(1) – C(31)	1.871(3)	C(2) – H(2A)	0.9800
C(2) – H(2B)	0.9800	C(2) – H(2C)	0.9800
P(2) – C(22)	1.831(3)	P(2) – C(42)	1.854(3)
P(2) – C(41)	1.858(4)	C(12) – C(13)	1.390(5)
C(12) – C(11)	1.412(5)	C(11) – C(16)	1.393(5)
C(13) – C(14)	1.390(5)	C(13) – H(13)	0.9500
C(14) – C(15)	1.360(6)	C(14) – H(14)	0.9500
C(15) – C(16)	1.382(5)	C(15) – H(15)	0.9500
C(16) – H(16)	0.9500	C(21) – C(26)	1.403(5)
C(21) – C(22)	1.409(5)	C(22) – C(23)	1.404(5)
C(23) – C(24)	1.389(5)	C(23) – H(23)	0.9500
C(24) – C(25)	1.371(6)	C(24) – H(24)	0.9500
C(25) – C(26)	1.381(5)	C(25) – H(25)	0.9500
C(26) – H(26)	0.9500	C(31) – C(33)	1.528(5)
C(31) – C(34)	1.544(5)	C(31) – H(31)	1.0000
C(32) – C(35)	1.529(6)	C(32) – C(36)	1.549(6)
C(32) – H(32)	1.0000	C(33) – H(33A)	0.9800
C(33) – H(33B)	0.9800	C(33) – H(33C)	0.9800
C(34) – H(34A)	0.9800	C(34) – H(34B)	0.9800
C(34) – H(34C)	0.9800	C(35) – H(35A)	0.9800
C(35) – H(35B)	0.9800	C(35) – H(35C)	0.9800
C(36) – H(36A)	0.9800	C(36) – H(36B)	0.9800
C(36) – H(36C)	0.9800	C(41) – C(43)	1.521(5)
C(41) – C(44)	1.529(5)	C(41) – H(41)	1.0000
C(42) – C(45)	1.513(5)	C(42) – C(46)	1.530(6)
C(42) – H(42)	1.0000	C(43) – H(43A)	0.9800
C(43) – H(43B)	0.9800	C(43) – H(43C)	0.9800
C(44) – H(44A)	0.9800	C(44) – H(44B)	0.9800
C(44) – H(44C)	0.9800	C(45) – H(45A)	0.9800
C(45) – H(45B)	0.9800	C(45) – H(45C)	0.9800
C(46) – H(46A)	0.9800	C(46) – H(46B)	0.9800
C(46) – H(46C)	0.9800		

Table S45. Angles [°] for $[PC(CH_3)HP]PdCl_2$ (**8**).

atom – atom – atom	angle	atom – atom – atom	angle
P(2) – Pd – P(1)	99.98(3)	P(2) – Pd – Cl(2)	87.28(3)
P(1) – Pd – Cl(2)	172.10(3)	P(2) – Pd – Cl(1)	169.23(3)
P(1) – Pd – Cl(1)	86.66(3)	Cl(2) – Pd – Cl(1)	86.63(3)
C(11) – C(1) – C(21)	114.3(3)	C(11) – C(1) – C(2)	112.8(3)
C(21) – C(1) – C(2)	107.4(3)	C(11) – C(1) – H(1)	107.3
C(21) – C(1) – H(1)	107.3	C(2) – C(1) – H(1)	107.3
C(12) – P(1) – C(32)	105.75(16)	C(12) – P(1) – C(31)	105.16(16)
C(32) – P(1) – C(31)	105.98(17)	C(12) – P(1) – Pd	106.04(11)
C(32) – P(1) – Pd	108.01(13)	C(31) – P(1) – Pd	124.55(12)
C(1) – C(2) – H(2A)	109.5	C(1) – C(2) – H(2B)	109.5
H(2A) – C(2) – H(2B)	109.5	C(1) – C(2) – H(2C)	109.5
H(2A) – C(2) – H(2C)	109.5	H(2B) – C(2) – H(2C)	109.5
C(22) – P(2) – C(42)	107.93(16)	C(22) – P(2) – C(41)	105.80(17)
C(42) – P(2) – C(41)	101.38(17)	C(22) – P(2) – Pd	112.22(11)
C(42) – P(2) – Pd	113.04(12)	C(41) – P(2) – Pd	115.60(11)
C(13) – C(12) – C(11)	118.8(3)	C(13) – C(12) – P(1)	118.4(3)
C(11) – C(12) – P(1)	122.8(2)	C(16) – C(11) – C(12)	117.2(3)
C(16) – C(11) – C(1)	118.6(3)	C(12) – C(11) – C(1)	124.1(3)
C(14) – C(13) – C(12)	122.1(4)	C(14) – C(13) – H(13)	118.9
C(12) – C(13) – H(13)	118.9	C(15) – C(14) – C(13)	119.2(3)
C(15) – C(14) – H(14)	120.4	C(13) – C(14) – H(14)	120.4
C(14) – C(15) – C(16)	119.6(3)	C(14) – C(15) – H(15)	120.2
C(16) – C(15) – H(15)	120.2	C(15) – C(16) – C(11)	123.0(3)
C(15) – C(16) – H(16)	118.5	C(11) – C(16) – H(16)	118.5
C(26) – C(21) – C(22)	118.1(3)	C(26) – C(21) – C(1)	116.6(3)
C(22) – C(21) – C(1)	125.2(3)	C(23) – C(22) – C(21)	118.8(3)
C(23) – C(22) – P(2)	118.6(3)	C(21) – C(22) – P(2)	122.3(2)
C(24) – C(23) – C(22)	121.6(4)	C(24) – C(23) – H(23)	119.2
C(22) – C(23) – H(23)	119.2	C(25) – C(24) – C(23)	119.3(3)
C(25) – C(24) – H(24)	120.4	C(23) – C(24) – H(24)	120.4
C(24) – C(25) – C(26)	120.3(4)	C(24) – C(25) – H(25)	119.8
C(26) – C(25) – H(25)	119.8	C(25) – C(26) – C(21)	121.8(4)
C(25) – C(26) – H(26)	119.1	C(21) – C(26) – H(26)	119.1
C(33) – C(31) – C(34)	109.4(3)	C(33) – C(31) – P(1)	113.7(3)
C(34) – C(31) – P(1)	114.4(3)	C(33) – C(31) – H(31)	106.2
C(34) – C(31) – H(31)	106.2	P(1) – C(31) – H(31)	106.2
C(35) – C(32) – C(36)	109.5(3)	C(35) – C(32) – P(1)	110.8(3)
C(36) – C(32) – P(1)	119.3(3)	C(35) – C(32) – H(32)	105.4
C(36) – C(32) – H(32)	105.4	P(1) – C(32) – H(32)	105.4
C(31) – C(33) – H(33A)	109.5	C(31) – C(33) – H(33B)	109.5
H(33A) – C(33) – H(33B)	109.5	C(31) – C(33) – H(33C)	109.5

Continued on next page

Table S45. – continued from previous page

atom – atom – atom	angle	atom – atom – atom	angle
H(33A) – C(33) – H(33C)	109.5	H(33B) – C(33) – H(33C)	109.5
C(31) – C(34) – H(34A)	109.5	C(31) – C(34) – H(34B)	109.5
H(34A) – C(34) – H(34B)	109.5	C(31) – C(34) – H(34C)	109.5
H(34A) – C(34) – H(34C)	109.5	H(34B) – C(34) – H(34C)	109.5
C(32) – C(35) – H(35A)	109.5	C(32) – C(35) – H(35B)	109.5
H(35A) – C(35) – H(35B)	109.5	C(32) – C(35) – H(35C)	109.5
H(35A) – C(35) – H(35C)	109.5	H(35B) – C(35) – H(35C)	109.5
C(32) – C(36) – H(36A)	109.5	C(32) – C(36) – H(36B)	109.5
H(36A) – C(36) – H(36B)	109.5	C(32) – C(36) – H(36C)	109.5
H(36A) – C(36) – H(36C)	109.5	H(36B) – C(36) – H(36C)	109.5
C(43) – C(41) – C(44)	110.2(3)	C(43) – C(41) – P(2)	114.5(2)
C(44) – C(41) – P(2)	111.9(3)	C(43) – C(41) – H(41)	106.6
C(44) – C(41) – H(41)	106.6	P(2) – C(41) – H(41)	106.6
C(45) – C(42) – C(46)	110.1(3)	C(45) – C(42) – P(2)	112.2(3)
C(46) – C(42) – P(2)	110.7(3)	C(45) – C(42) – H(42)	107.9
C(46) – C(42) – H(42)	107.9	P(2) – C(42) – H(42)	107.9
C(41) – C(43) – H(43A)	109.5	C(41) – C(43) – H(43B)	109.5
H(43A) – C(43) – H(43B)	109.5	C(41) – C(43) – H(43C)	109.5
H(43A) – C(43) – H(43C)	109.5	H(43B) – C(43) – H(43C)	109.5
C(41) – C(44) – H(44A)	109.5	C(41) – C(44) – H(44B)	109.5
H(44A) – C(44) – H(44B)	109.5	C(41) – C(44) – H(44C)	109.5
H(44A) – C(44) – H(44C)	109.5	H(44B) – C(44) – H(44C)	109.5
C(42) – C(45) – H(45A)	109.5	C(42) – C(45) – H(45B)	109.5
H(45A) – C(45) – H(45B)	109.5	C(42) – C(45) – H(45C)	109.5
H(45A) – C(45) – H(45C)	109.5	H(45B) – C(45) – H(45C)	109.5
C(42) – C(46) – H(46A)	109.5	C(42) – C(46) – H(46B)	109.5
H(46A) – C(46) – H(46B)	109.5	C(42) – C(46) – H(46C)	109.5
H(46A) – C(46) – H(46C)	109.5	H(46B) – C(46) – H(46C)	109.5

8.7 Crystal data for [PC(CH_3)P]PdCl (9)

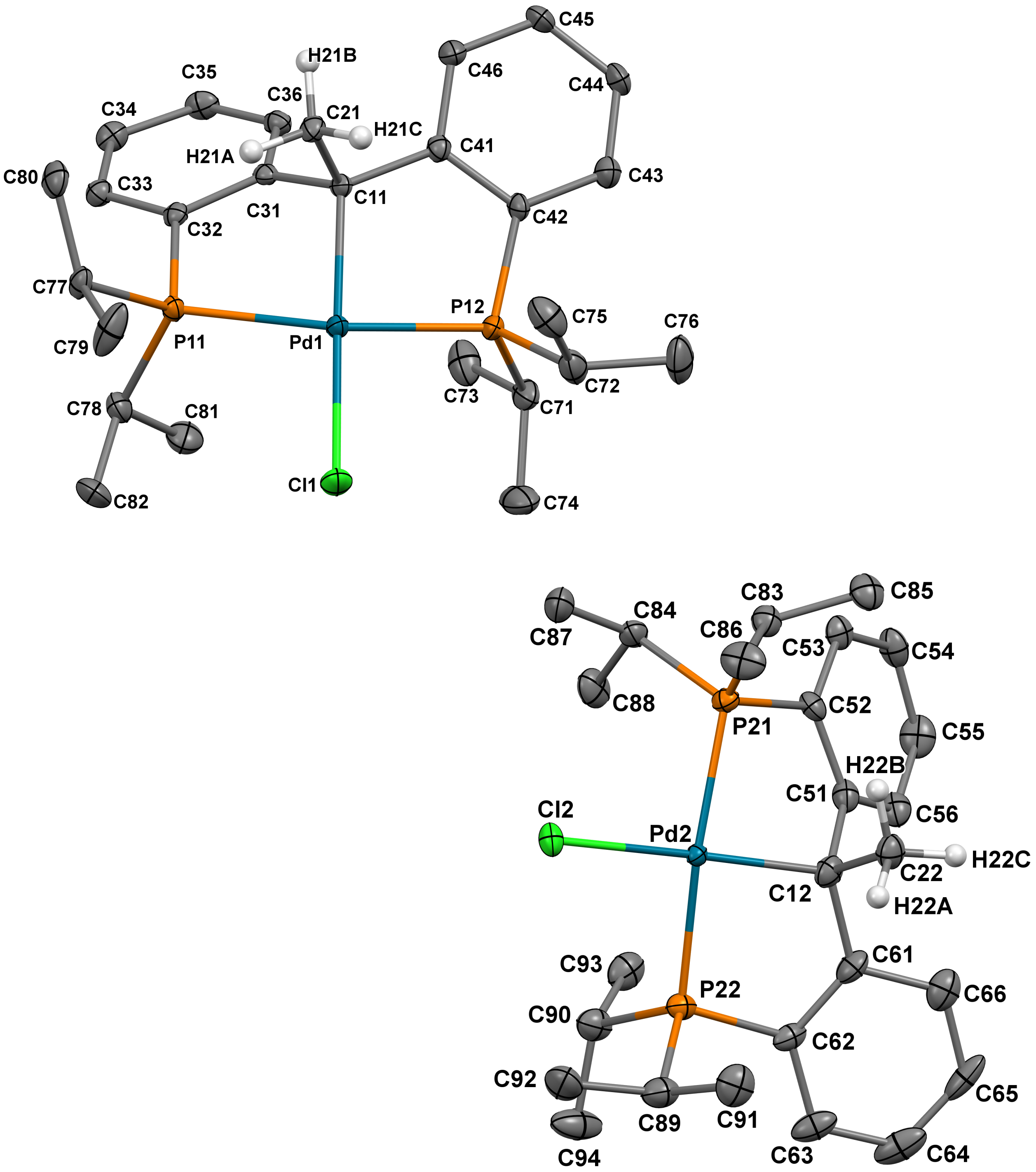

Figure S79. Thermal-ellipsoid representation (50% probability) of the two independent molecules of [PC(CH_3)P]PdCl (**9**). Most hydrogen atoms were omitted for clarity.

Table S46. Crystal data and structure refinement for [PC(CH_3)P]PdCl (**9**).

Identification code:	cc254	
Empirical formula:	$C_{26}H_{39}ClP_2Pd$	
Formula weight:	555.36	
Temperature:	120(2) K	
Wavelength:	0.71073 Å	
Crystal system:	Monoclinic	
Space group:	$P2_1/c$	
Unit cell dimensions:	a = 11.2685(8) Å	α = 90°
	b = 20.7303(14) Å	β = 99.2340(13)°
	c = 23.1605(16) Å	γ = 90°
Volume:	5340.2(6) Å^3	
Z:	8	
Density (calculated):	1.382 g·cm^{-3}	
Absorption coefficient (μ):	0.926 mm^{-1}	
F(000):	2304	
Crystal size:	0.10 × 0.09 × 0.08 mm^3	
θ range for data collection:	1.33 to 25.00°	
Index ranges:	$-13 \leq h \leq 13$, $-24 \leq k \leq 24$, $-27 \leq l \leq 27$	
Reflections collected:	62725	
Independent reflections:	9411 [R_{int} = 0.0607]	
Completeness to θ = 25.00°:	100.0 %	
Absorption correction:	Semi-empirical from equivalents	
Max. and min. transmission:	0.7454 and 0.7137	
Refinement method:	Full-matrix least-squares on F^2	
Data / restraints / parameters:	9411 / 0 / 569	
Goodness-of-fit on F^2:	1.108	
Final R indices [I>2σ(I)]:	R_1 = 0.0419, wR_2 = 0.0827	
R indices (all data):	R_1 = 0.0557, wR_2 = 0.0867	
Largest diff. peak and hole:	0.851 and −0.547 e$^-$·Å^{-3}	

Table S47. Atomic coordinates and equivalent isotropic displacement parameters (Å^2) for [PC(CH_3)P]PdCl (**9**). U(eq) is defined as one third of the trace of the orthogonalized U_{ij} tensor

atom	x	y	z	U(eq)
C(12)	0.5034(4)	0.2251(2)	0.47734(19)	0.030(1)
C(22)	0.6189(4)	0.1951(2)	0.4586(2)	0.033(1)
Pd(3)	0.439(2)	0.1817(7)	0.5558(4)	0.030(2)
Cl(2)	0.45948(9)	0.09518(5)	0.62723(5)	0.027(1)
Pd(2)	0.4786(2)	0.16822(9)	0.54898(5)	0.015(1)
P(22)	0.30408(10)	0.13795(5)	0.49142(5)	0.025(1)
P(21)	0.62632(10)	0.23339(5)	0.60021(5)	0.022(1)
Pd(1)	0.08191(3)	0.51140(1)	0.30826(1)	0.018(1)
Cl(1)	0.09327(10)	0.62588(5)	0.29280(5)	0.033(1)
C(11)	0.0853(3)	0.41436(18)	0.33183(17)	0.018(1)
P(11)	0.22457(9)	0.47953(5)	0.25065(5)	0.019(1)
P(12)	−0.09210(9)	0.51448(5)	0.34486(5)	0.021(1)
C(21)	0.2024(3)	0.4071(2)	0.37683(18)	0.023(1)
C(31)	0.0956(3)	0.37538(18)	0.27703(17)	0.018(1)
C(32)	0.1788(3)	0.39557(18)	0.24116(17)	0.019(1)
C(33)	0.2088(3)	0.35674(19)	0.19694(17)	0.022(1)
C(34)	0.1491(4)	0.2988(2)	0.18408(19)	0.026(1)
C(35)	0.0571(4)	0.2815(2)	0.21410(19)	0.027(1)
C(36)	0.0314(3)	0.31892(19)	0.26011(18)	0.023(1)
C(41)	−0.0164(3)	0.39225(19)	0.36368(17)	0.021(1)
C(43)	−0.2004(4)	0.4140(2)	0.40293(19)	0.029(1)
C(42)	−0.1071(3)	0.43467(19)	0.37406(18)	0.021(1)
C(44)	−0.2029(4)	0.3516(2)	0.4228(2)	0.033(1)
C(45)	−0.1115(4)	0.3094(2)	0.4147(2)	0.031(1)
C(46)	−0.0195(4)	0.3295(2)	0.38596(19)	0.027(1)
C(51)	0.5321(4)	0.2951(2)	0.49934(19)	0.029(1)
C(52)	0.6091(4)	0.3044(2)	0.55354(19)	0.025(1)
C(54)	0.6182(4)	0.4188(2)	0.5359(2)	0.036(1)
C(55)	0.5330(4)	0.4109(2)	0.4863(2)	0.039(1)
C(56)	0.4915(4)	0.3503(2)	0.4676(2)	0.035(1)
C(61)	0.4043(4)	0.2213(2)	0.42489(19)	0.033(1)
C(62)	0.3043(4)	0.1834(2)	0.42524(18)	0.027(1)
C(63)	0.2183(5)	0.1761(2)	0.3751(2)	0.041(1)
C(64)	0.2295(6)	0.2080(2)	0.3247(2)	0.053(2)
C(65)	0.3308(6)	0.2443(3)	0.3228(2)	0.048(2)
C(66)	0.4180(5)	0.2510(2)	0.3708(2)	0.041(1)
C(71)	−0.2309(4)	0.5222(2)	0.2908(2)	0.032(1)
C(72)	−0.0998(4)	0.5764(2)	0.4014(2)	0.036(1)
C(73)	−0.2332(4)	0.4705(3)	0.2438(2)	0.045(1)

Continued on next page

Table S47. – continued from previous page

atom	x	y	x	U(eq)
C(74)	−0.2441(5)	0.5891(3)	0.2650(3)	0.053(2)
C(75)	0.0132(5)	0.5743(3)	0.4457(2)	0.046(1)
C(76)	−0.2127(5)	0.5755(3)	0.4309(2)	0.051(2)
C(77)	0.3923(3)	0.48509(19)	0.2656(2)	0.025(1)
C(78)	0.1884(4)	0.5105(2)	0.17456(19)	0.029(1)
C(79)	0.4261(4)	0.5456(2)	0.3015(3)	0.051(2)
C(80)	0.4574(4)	0.4258(2)	0.2922(2)	0.033(1)
C(81)	0.0529(4)	0.5082(2)	0.1536(2)	0.041(1)
C(82)	0.2363(5)	0.5785(2)	0.1673(2)	0.043(1)
C(83)	0.7894(4)	0.2129(2)	0.6167(2)	0.031(1)
C(84)	0.5950(4)	0.2647(2)	0.67091(19)	0.029(1)
C(85)	0.8701(4)	0.2496(2)	0.5808(2)	0.039(1)
C(86)	0.8054(4)	0.1399(2)	0.6135(2)	0.045(1)
C(87)	0.6253(5)	0.2170(2)	0.7204(2)	0.040(1)
C(88)	0.4637(4)	0.2865(2)	0.6648(2)	0.041(1)
C(89)	0.2750(4)	0.0542(2)	0.4674(2)	0.040(1)
C(90)	0.1713(4)	0.1658(2)	0.5228(2)	0.038(1)
C(91)	0.3755(5)	0.0323(2)	0.4365(2)	0.046(1)
C(92)	0.2568(5)	0.0096(2)	0.5187(2)	0.050(1)
C(93)	0.1794(4)	0.2400(2)	0.5280(2)	0.041(1)
C(94)	0.0513(4)	0.1451(3)	0.4886(3)	0.060(2)
C(53)	0.6552(4)	0.3650(2)	0.5706(2)	0.028(1)
H(22A)	0.6031	0.1501	0.4468	0.050
H(22B)	0.6852	0.1968	0.4915	0.050
H(22C)	0.6406	0.2196	0.4256	0.050
H(21A)	0.2710	0.4222	0.3593	0.034
H(21B)	0.2141	0.3617	0.3879	0.034
H(21C)	0.1960	0.4330	0.4116	0.034
H(33)	0.2700	0.3698	0.1756	0.026
H(34)	0.1711	0.2711	0.1549	0.032
H(35)	0.0112	0.2438	0.2031	0.032
H(36)	−0.0312	0.3059	0.2806	0.028
H(43)	−0.2622	0.4433	0.4088	0.034
H(44)	−0.2668	0.3375	0.4420	0.039
H(45)	−0.1121	0.2664	0.4289	0.037
H(46)	0.0430	0.3001	0.3812	0.033
H(54)	0.6512	0.4601	0.5462	0.043
H(55)	0.5023	0.4478	0.4646	0.047
H(56)	0.4346	0.3462	0.4328	0.042
H(63)	0.1513	0.1485	0.3760	0.050
H(64)	0.1683	0.2052	0.2914	0.063
H(65)	0.3405	0.2653	0.2874	0.057

Continued on next page

Table S47. – continued from previous page

atom	x	y	x	U(eq)
H(66)	0.4882	0.2755	0.3681	0.050
H(71)	−0.3008	0.5143	0.3116	0.038
H(72)	−0.0998	0.6190	0.3812	0.043
H(73A)	−0.3102	0.4723	0.2173	0.067
H(73B)	−0.2234	0.4279	0.2623	0.067
H(73C)	−0.1674	0.4782	0.2216	0.067
H(74A)	−0.1748	0.5987	0.2458	0.080
H(74B)	−0.2483	0.6206	0.2961	0.080
H(74C)	−0.3180	0.5914	0.2362	0.080
H(75A)	0.0827	0.5829	0.4264	0.069
H(75B)	0.0216	0.5316	0.4640	0.069
H(75C)	0.0089	0.6071	0.4758	0.069
H(76A)	−0.2845	0.5723	0.4009	0.077
H(76B)	−0.2162	0.6152	0.4534	0.077
H(76C)	−0.2093	0.5382	0.4572	0.077
H(77)	0.4191	0.4920	0.2269	0.031
H(78)	0.2272	0.4811	0.1489	0.035
H(79A)	0.5113	0.5554	0.3018	0.077
H(79B)	0.4118	0.5387	0.3417	0.077
H(79C)	0.3770	0.5819	0.2842	0.077
H(80A)	0.4296	0.3879	0.2685	0.049
H(80B)	0.4406	0.4198	0.3321	0.049
H(80C)	0.5441	0.4312	0.2932	0.049
H(81A)	0.0357	0.5202	0.1122	0.061
H(81B)	0.0127	0.5385	0.1766	0.061
H(81C)	0.0233	0.4644	0.1586	0.061
H(82A)	0.2108	0.5930	0.1270	0.065
H(82B)	0.3243	0.5781	0.1761	0.065
H(82C)	0.2045	0.6079	0.1942	0.065
H(83)	0.8167	0.2252	0.6584	0.037
H(84)	0.6467	0.3037	0.6809	0.034
H(85A)	0.8393	0.2443	0.5390	0.059
H(85B)	0.9521	0.2324	0.5894	0.059
H(85C)	0.8707	0.2955	0.5910	0.059
H(86A)	0.7859	0.1258	0.5727	0.067
H(86B)	0.7517	0.1185	0.6369	0.067
H(86C)	0.8890	0.1287	0.6289	0.067
H(87A)	0.5830	0.1763	0.7099	0.061
H(87B)	0.6003	0.2344	0.7559	0.061
H(87C)	0.7122	0.2092	0.7275	0.061
H(88A)	0.4472	0.3184	0.6333	0.062
H(88B)	0.4494	0.3059	0.7017	0.062

Continued on next page

Table S47. – continued from previous page

atom	x	y	x	U(eq)
H(88C)	0.4107	0.2492	0.6556	0.062
H(89)	0.1991	0.0537	0.4384	0.048
H(90)	0.1778	0.1477	0.5632	0.045
H(91A)	0.3640	−0.0132	0.4257	0.070
H(91B)	0.4525	0.0377	0.4625	0.070
H(91C)	0.3754	0.0583	0.4011	0.070
H(92A)	0.2416	−0.0346	0.5042	0.075
H(92B)	0.1879	0.0247	0.5360	0.075
H(92C)	0.3292	0.0102	0.5484	0.075
H(93A)	0.1683	0.2591	0.4887	0.062
H(93B)	0.2585	0.2522	0.5492	0.062
H(93C)	0.1166	0.2558	0.5491	0.062
H(94A)	0.0412	0.1638	0.4493	0.089
H(94B)	−0.0136	0.1601	0.5088	0.089
H(94C)	0.0486	0.0980	0.4857	0.089
H(53)	0.7115	0.3697	0.6055	0.034

Table S48. Anisotropic displacement parameters (Å^2) for [PC(CH_3)P]PdCl (**9**). The anisotropic displacement factor exponent takes the form: $-2\pi^2[h^2a^{*2}U_{11} + ... + 2hka^*b^*U_{12}]$.

atom	U_{11}	U_{22}	U_{33}	U_{23}	U_{13}	U_{12}
C(12)	0.031(2)	0.034(3)	0.025(2)	−0.002(2)	0.008(2)	0.005(2)
C(22)	0.037(3)	0.034(3)	0.031(3)	−0.002(2)	0.013(2)	0.002(2)
Pd(3)	0.031(5)	0.034(3)	0.025(2)	0.0032(17)	0.007(3)	0.009(4)
Cl(2)	0.0274(5)	0.0285(6)	0.0283(6)	0.0067(5)	0.0107(5)	0.0016(4)
Pd(2)	0.0137(6)	0.0186(4)	0.0144(3)	−0.0005(2)	0.0028(2)	0.0017(3)
P(22)	0.0200(5)	0.0283(6)	0.0270(6)	−0.0063(5)	0.0033(5)	0.0023(5)
P(21)	0.0233(6)	0.0228(6)	0.0195(6)	0.0005(4)	−0.0002(5)	0.0005(4)
Pd(1)	0.0165(2)	0.0152(2)	0.0240(2)	0.0013(1)	0.0072(1)	0.0004(1)
Cl(1)	0.0393(6)	0.0164(5)	0.0468(7)	0.0027(5)	0.0177(5)	0.0020(4)
C(11)	0.0129(19)	0.018(2)	0.024(2)	0.0034(17)	0.0048(17)	0.0002(15)
P(11)	0.0182(5)	0.0178(5)	0.0229(6)	−0.0002(4)	0.0078(4)	−0.0013(4)
P(12)	0.0174(5)	0.0200(5)	0.0257(6)	0.0035(5)	0.0076(4)	0.0039(4)
C(21)	0.018(2)	0.027(2)	0.023(2)	0.0025(18)	0.0034(17)	0.0042(17)
C(31)	0.0159(19)	0.018(2)	0.019(2)	0.0054(16)	0.0011(16)	0.0018(16)
C(32)	0.0137(19)	0.020(2)	0.022(2)	0.0016(17)	0.0011(16)	0.0021(16)
C(33)	0.020(2)	0.022(2)	0.023(2)	−0.0007(18)	0.0029(17)	0.0002(17)
C(34)	0.026(2)	0.026(2)	0.026(2)	−0.0030(19)	0.0000(19)	0.0015(18)
C(35)	0.024(2)	0.020(2)	0.034(3)	−0.0022(19)	−0.0007(19)	−0.0059(18)
C(36)	0.019(2)	0.021(2)	0.030(2)	0.0058(18)	0.0040(18)	−0.0009(17)
C(41)	0.017(2)	0.024(2)	0.021(2)	0.0007(17)	0.0034(17)	0.0005(17)
C(43)	0.023(2)	0.030(2)	0.036(3)	0.003(2)	0.010(2)	0.0013(19)
C(42)	0.0132(19)	0.025(2)	0.025(2)	0.0034(18)	0.0061(17)	−0.0012(16)
C(44)	0.029(2)	0.035(3)	0.038(3)	0.010(2)	0.018(2)	−0.002(2)
C(45)	0.034(3)	0.025(2)	0.035(3)	0.010(2)	0.016(2)	−0.0007(19)
C(46)	0.026(2)	0.026(2)	0.032(2)	0.009(2)	0.0097(19)	0.0068(19)
C(51)	0.026(2)	0.033(3)	0.029(2)	0.008(2)	0.008(2)	0.0035(19)
C(52)	0.018(2)	0.024(2)	0.033(3)	0.0027(19)	0.0064(18)	−0.0017(17)
C(54)	0.043(3)	0.022(2)	0.047(3)	0.001(2)	0.021(2)	0.000(2)
C(55)	0.039(3)	0.034(3)	0.047(3)	0.009(2)	0.012(2)	0.019(2)
C(56)	0.029(2)	0.038(3)	0.036(3)	0.009(2)	0.005(2)	0.005(2)
C(61)	0.040(3)	0.037(3)	0.021(2)	0.003(2)	0.000(2)	0.010(2)
C(62)	0.021(2)	0.037(3)	0.023(2)	−0.0096(19)	0.0006(18)	0.0109(19)
C(63)	0.048(3)	0.037(3)	0.034(3)	−0.011(2)	−0.010(2)	0.017(2)
C(64)	0.079(4)	0.034(3)	0.037(3)	−0.011(2)	−0.015(3)	0.024(3)
C(65)	0.083(4)	0.042(3)	0.016(2)	0.002(2)	0.003(3)	0.033(3)
C(66)	0.048(3)	0.041(3)	0.038(3)	0.002(2)	0.013(3)	0.014(2)
C(71)	0.019(2)	0.041(3)	0.036(3)	0.011(2)	0.007(2)	0.0051(19)
C(72)	0.046(3)	0.029(3)	0.038(3)	−0.003(2)	0.023(2)	−0.003(2)
C(73)	0.029(3)	0.073(4)	0.031(3)	−0.001(3)	0.003(2)	−0.005(3)
C(74)	0.037(3)	0.054(3)	0.068(4)	0.034(3)	0.011(3)	0.015(3)

Continued on next page

Table S48. – continued from previous page

atom	U_{11}	U_{22}	U_{33}	U_{23}	U_{13}	U_{12}
C(75)	0.055(3)	0.051(3)	0.033(3)	−0.001(2)	0.011(3)	−0.015(3)
C(76)	0.054(3)	0.052(3)	0.058(4)	−0.018(3)	0.036(3)	−0.004(3)
C(77)	0.018(2)	0.023(2)	0.038(3)	−0.005(2)	0.0121(19)	−0.0023(17)
C(78)	0.034(2)	0.031(2)	0.025(2)	0.003(2)	0.013(2)	0.000(2)
C(79)	0.018(2)	0.046(3)	0.090(4)	−0.037(3)	0.010(3)	−0.008(2)
C(80)	0.017(2)	0.044(3)	0.039(3)	0.000(2)	0.008(2)	0.001(2)
C(81)	0.044(3)	0.047(3)	0.030(3)	0.009(2)	0.004(2)	0.007(2)
C(82)	0.052(3)	0.035(3)	0.047(3)	0.019(2)	0.023(3)	0.001(2)
C(83)	0.028(2)	0.034(3)	0.031(3)	0.002(2)	0.006(2)	0.003(2)
C(84)	0.031(2)	0.029(2)	0.027(2)	−0.0072(19)	0.008(2)	−0.0056(19)
C(85)	0.027(3)	0.044(3)	0.046(3)	0.003(2)	0.008(2)	0.005(2)
C(86)	0.040(3)	0.031(3)	0.060(4)	0.005(2)	−0.004(3)	0.012(2)
C(87)	0.046(3)	0.046(3)	0.031(3)	0.000(2)	0.013(2)	−0.007(2)
C(88)	0.036(3)	0.045(3)	0.049(3)	−0.011(2)	0.023(2)	0.002(2)
C(89)	0.036(3)	0.045(3)	0.037(3)	−0.009(2)	−0.001(2)	0.000(2)
C(90)	0.032(3)	0.040(3)	0.041(3)	−0.012(2)	0.009(2)	0.001(2)
C(91)	0.050(3)	0.041(3)	0.050(3)	−0.004(3)	0.015(3)	0.013(2)
C(92)	0.062(4)	0.039(3)	0.050(3)	−0.006(3)	0.010(3)	−0.020(3)
C(93)	0.037(3)	0.050(3)	0.039(3)	−0.007(2)	0.009(2)	0.014(2)
C(94)	0.026(3)	0.081(4)	0.071(4)	−0.042(3)	0.005(3)	0.001(3)
C(53)	0.025(2)	0.030(2)	0.033(3)	−0.002(2)	0.013(2)	0.0057(19)

Table S49. Distances [Å] for [PC(CH_3)P]PdCl (**9**).

atom – atom	distance	atom – atom	distance
C(12) – C(61)	1.514(6)	C(12) – C(51)	1.555(6)
C(12) – C(22)	1.566(6)	C(12) – Pd(2)	2.091(5)
C(22) – H(22A)	0.9800	C(22) – H(22B)	0.9800
C(22) – H(22C)	0.9800	Pd(3) – Pd(2)	0.57(2)
Pd(3) – P(22)	2.154(7)	Pd(3) – Cl(2)	2.425(7)
Pd(3) – P(21)	2.440(11)	Cl(2) – Pd(2)	2.3978(12)
Pd(2) – P(22)	2.2795(16)	Pd(2) – P(21)	2.3176(13)
P(22) – C(62)	1.799(5)	P(22) – C(89)	1.836(5)
P(22) – C(90)	1.856(5)	P(21) – C(52)	1.818(4)
P(21) – C(84)	1.847(4)	P(21) – C(83)	1.865(4)
Pd(1) – C(11)	2.083(4)	Pd(1) – P(12)	2.2585(10)
Pd(1) – P(11)	2.3430(10)	Pd(1) – Cl(1)	2.4065(10)
C(11) – C(31)	1.525(5)	C(11) – C(41)	1.530(5)
C(11) – C(21)	1.551(5)	P(11) – C(32)	1.819(4)
P(11) – C(78)	1.859(4)	P(11) – C(77)	1.869(4)
P(12) – C(42)	1.806(4)	P(12) – C(72)	1.845(5)
P(12) – C(71)	1.847(4)	C(21) – H(21A)	0.9800
C(21) – H(21B)	0.9800	C(21) – H(21C)	0.9800
C(31) – C(36)	1.399(5)	C(31) – C(32)	1.412(5)
C(32) – C(33)	1.387(5)	C(33) – C(34)	1.386(6)
C(33) – H(33)	0.9500	C(34) – C(35)	1.385(6)
C(34) – H(34)	0.9500	C(35) – C(36)	1.385(6)
C(35) – H(35)	0.9500	C(36) – H(36)	0.9500
C(41) – C(42)	1.398(5)	C(41) – C(46)	1.403(6)
C(43) – C(44)	1.376(6)	C(43) – C(42)	1.400(5)
C(43) – H(43)	0.9500	C(44) – C(45)	1.387(6)
C(44) – H(44)	0.9500	C(45) – C(46)	1.383(6)
C(45) – H(45)	0.9500	C(46) – H(46)	0.9500
C(51) – C(56)	1.396(6)	C(51) – C(52)	1.420(6)
C(52) – C(53)	1.392(6)	C(54) – C(55)	1.383(7)
C(54) – C(53)	1.398(6)	C(54) – H(54)	0.9500
C(55) – C(56)	1.386(7)	C(55) – H(55)	0.9500
C(56) – H(56)	0.9500	C(61) – C(62)	1.375(6)
C(61) – C(66)	1.425(6)	C(62) – C(63)	1.397(6)
C(63) – C(64)	1.366(7)	C(63) – H(63)	0.9500
C(64) – C(65)	1.373(8)	C(64) – H(64)	0.9500
C(65) – C(66)	1.367(7)	C(65) – H(65)	0.9500
C(66) – H(66)	0.9500	C(71) – C(74)	1.508(6)
C(71) – C(73)	1.524(7)	C(71) – H(71)	1.0000
C(72) – C(75)	1.503(7)	C(72) – C(76)	1.538(6)
C(72) – H(72)	1.0000	C(73) – H(73A)	0.9800

Continued on next page

Table S49. – continued from previous page

atom – atom	distance	atom – atom	distance
C(73) – H(73B)	0.9800	C(73) – H(73C)	0.9800
C(74) – H(74A)	0.9800	C(74) – H(74B)	0.9800
C(74) – H(74C)	0.9800	C(75) – H(75A)	0.9800
C(75) – H(75B)	0.9800	C(75) – H(75C)	0.9800
C(76) – H(76A)	0.9800	C(76) – H(76B)	0.9800
C(76) – H(76C)	0.9800	C(77) – C(80)	1.511(6)
C(77) – C(79)	1.521(6)	C(77) – H(77)	1.0000
C(78) – C(82)	1.527(6)	C(78) – C(81)	1.527(6)
C(78) – H(78)	1.0000	C(79) – H(79A)	0.9800
C(79) – H(79B)	0.9800	C(79) – H(79C)	0.9800
C(80) – H(80A)	0.9800	C(80) – H(80B)	0.9800
C(80) – H(80C)	0.9800	C(81) – H(81A)	0.9800
C(81) – H(81B)	0.9800	C(81) – H(81C)	0.9800
C(82) – H(82A)	0.9800	C(82) – H(82B)	0.9800
C(82) – H(82C)	0.9800	C(83) – C(86)	1.526(6)
C(83) – C(85)	1.530(6)	C(83) – H(83)	1.0000
C(84) – C(87)	1.512(6)	C(84) – C(88)	1.531(6)
C(84) – H(84)	1.0000	C(85) – H(85A)	0.9800
C(85) – H(85B)	0.9800	C(85) – H(85C)	0.9800
C(86) – H(86A)	0.9800	C(86) – H(86B)	0.9800
C(86) – H(86C)	0.9800	C(87) – H(87A)	0.9800
C(87) – H(87B)	0.9800	C(87) – H(87C)	0.9800
C(88) – H(88A)	0.9800	C(88) – H(88B)	0.9800
C(88) – H(88C)	0.9800	C(89) – C(91)	1.503(7)
C(89) – C(92)	1.545(7)	C(89) – H(89)	1.0000
C(90) – C(94)	1.516(6)	C(90) – C(93)	1.545(7)
C(90) – H(90)	1.0000	C(91) – H(91A)	0.9800
C(91) – H(91B)	0.9800	C(91) – H(91C)	0.9800
C(92) – H(92A)	0.9800	C(92) – H(92B)	0.9800
C(92) – H(92C)	0.9800	C(93) – H(93A)	0.9800
C(93) – H(93B)	0.9800	C(93) – H(93C)	0.9800
C(94) – H(94A)	0.9800	C(94) – H(94B)	0.9800
C(94) – H(94C)	0.9800	C(53) – H(53)	0.9500

Table S50. Angles [°] for [PC(CH_3)P]PdCl (**9**).

atom – atom – atom	angle	atom – atom – atom	angle
C(61) – C(12) – C(51)	113.5(4)	C(61) – C(12) – C(22)	107.2(4)
C(51) – C(12) – C(22)	108.9(4)	C(61) – C(12) – Pd(2)	115.6(3)
C(51) – C(12) – Pd(2)	108.3(3)	C(22) – C(12) – Pd(2)	102.7(3)
Pd(2) – Pd(3) – P(22)	95.5(9)	Pd(2) – Pd(3) – Cl(2)	80.5(8)
P(22) – Pd(3) – Cl(2)	98.0(2)	Pd(2) – Pd(3) – P(21)	71.0(9)
P(22) – Pd(3) – P(21)	160.7(10)	Cl(2) – Pd(3) – P(21)	93.3(4)
Pd(3) – Pd(2) – C(12)	98.9(6)	Pd(3) – Pd(2) – P(22)	70.2(6)
C(12) – Pd(2) – P(22)	84.48(13)	Pd(3) – Pd(2) – P(21)	95.6(6)
C(12) – Pd(2) – P(21)	84.32(13)	P(22) – Pd(2) – P(21)	160.27(13)
Pd(3) – Pd(2) – Cl(2)	86.0(6)	C(12) – Pd(2) – Cl(2)	174.7(2)
P(22) – Pd(2) – Cl(2)	95.43(6)	P(21) – Pd(2) – Cl(2)	97.20(4)
C(62) – P(22) – C(89)	105.2(2)	C(62) – P(22) – C(90)	106.4(2)
C(89) – P(22) – C(90)	107.3(2)	C(62) – P(22) – Pd(3)	105.8(2)
C(89) – P(22) – Pd(3)	132.8(5)	C(90) – P(22) – Pd(3)	97.0(7)
C(62) – P(22) – Pd(2)	103.59(15)	C(89) – P(22) – Pd(2)	122.17(18)
C(90) – P(22) – Pd(2)	111.05(17)	C(52) – P(21) – C(84)	103.0(2)
C(52) – P(21) – C(83)	108.33(19)	C(84) – P(21) – C(83)	102.8(2)
C(52) – P(21) – Pd(2)	99.67(14)	C(84) – P(21) – Pd(2)	115.79(16)
C(83) – P(21) – Pd(2)	125.09(17)	C(52) – P(21) – Pd(3)	95.9(3)
C(84) – P(21) – Pd(3)	104.6(6)	C(83) – P(21) – Pd(3)	137.9(6)
C(11) – Pd(1) – P(12)	84.81(10)	C(11) – Pd(1) – P(11)	83.77(11)
P(12) – Pd(1) – P(11)	159.73(4)	C(11) – Pd(1) – Cl(1)	172.80(11)
P(12) – Pd(1) – Cl(1)	95.63(4)	P(11) – Pd(1) – Cl(1)	97.79(4)
C(31) – C(11) – C(41)	113.9(3)	C(31) – C(11) – C(21)	109.8(3)
C(41) – C(11) – C(21)	105.6(3)	C(31) – C(11) – Pd(1)	107.1(2)
C(41) – C(11) – Pd(1)	115.6(3)	C(21) – C(11) – Pd(1)	104.3(2)
C(32) – P(11) – C(78)	101.63(19)	C(32) – P(11) – C(77)	109.87(17)
C(78) – P(11) – C(77)	102.4(2)	C(32) – P(11) – Pd(1)	97.57(13)
C(78) – P(11) – Pd(1)	112.13(14)	C(77) – P(11) – Pd(1)	130.02(14)
C(42) – P(12) – C(72)	110.7(2)	C(42) – P(12) – C(71)	102.29(19)
C(72) – P(12) – C(71)	106.8(2)	C(42) – P(12) – Pd(1)	104.71(13)
C(72) – P(12) – Pd(1)	115.38(15)	C(71) – P(12) – Pd(1)	116.12(15)
C(11) – C(21) – H(21A)	109.5	C(11) – C(21) – H(21B)	109.5
H(21A) – C(21) – H(21B)	109.5	C(11) – C(21) – H(21C)	109.5
H(21A) – C(21) – H(21C)	109.5	H(21B) – C(21) – H(21C)	109.5
C(36) – C(31) – C(32)	116.5(4)	C(36) – C(31) – C(11)	124.9(3)
C(32) – C(31) – C(11)	118.6(3)	C(33) – C(32) – C(31)	121.8(4)
C(33) – C(32) – P(11)	123.2(3)	C(31) – C(32) – P(11)	114.5(3)
C(34) – C(33) – C(32)	119.6(4)	C(34) – C(33) – H(33)	120.2
C(32) – C(33) – H(33)	120.2	C(35) – C(34) – C(33)	119.6(4)
C(35) – C(34) – H(34)	120.2	C(33) – C(34) – H(34)	120.2

Continued on next page

Table S50. – continued from previous page

atom – atom – atom	angle	atom – atom – atom	angle
C(34) – C(35) – C(36)	120.5(4)	C(34) – C(35) – H(35)	119.8
C(36) – C(35) – H(35)	119.8	C(35) – C(36) – C(31)	121.4(4)
C(35) – C(36) – H(36)	119.3	C(31) – C(36) – H(36)	119.3
C(42) – C(41) – C(46)	117.3(4)	C(42) – C(41) – C(11)	121.3(3)
C(46) – C(41) – C(11)	121.3(3)	C(44) – C(43) – C(42)	120.3(4)
C(44) – C(43) – H(43)	119.8	C(42) – C(43) – H(43)	119.8
C(41) – C(42) – C(43)	121.0(4)	C(41) – C(42) – P(12)	113.4(3)
C(43) – C(42) – P(12)	125.6(3)	C(43) – C(44) – C(45)	119.5(4)
C(43) – C(44) – H(44)	120.3	C(45) – C(44) – H(44)	120.3
C(46) – C(45) – C(44)	120.4(4)	C(46) – C(45) – H(45)	119.8
C(44) – C(45) – H(45)	119.8	C(45) – C(46) – C(41)	121.4(4)
C(45) – C(46) – H(46)	119.3	C(41) – C(46) – H(46)	119.3
C(56) – C(51) – C(52)	117.1(4)	C(56) – C(51) – C(12)	124.0(4)
C(52) – C(51) – C(12)	118.8(4)	C(53) – C(52) – C(51)	121.4(4)
C(53) – C(52) – P(21)	124.2(3)	C(51) – C(52) – P(21)	114.1(3)
C(55) – C(54) – C(53)	119.1(4)	C(55) – C(54) – H(54)	120.4
C(53) – C(54) – H(54)	120.4	C(54) – C(55) – C(56)	121.4(4)
C(54) – C(55) – H(55)	119.3	C(56) – C(55) – H(55)	119.3
C(55) – C(56) – C(51)	120.8(4)	C(55) – C(56) – H(56)	119.6
C(51) – C(56) – H(56)	119.6	C(62) – C(61) – C(66)	117.1(4)
C(62) – C(61) – C(12)	121.6(4)	C(66) – C(61) – C(12)	120.8(4)
C(61) – C(62) – C(63)	121.1(4)	C(61) – C(62) – P(22)	114.7(3)
C(63) – C(62) – P(22)	123.8(4)	C(64) – C(63) – C(62)	120.8(5)
C(64) – C(63) – H(63)	119.6	C(62) – C(63) – H(63)	119.6
C(63) – C(64) – C(65)	118.9(5)	C(63) – C(64) – H(64)	120.6
C(65) – C(64) – H(64)	120.6	C(66) – C(65) – C(64)	121.5(5)
C(66) – C(65) – H(65)	119.2	C(64) – C(65) – H(65)	119.2
C(65) – C(66) – C(61)	120.3(5)	C(65) – C(66) – H(66)	119.8
C(61) – C(66) – H(66)	119.8	C(74) – C(71) – C(73)	111.9(4)
C(74) – C(71) – P(12)	111.6(3)	C(73) – C(71) – P(12)	109.8(3)
C(74) – C(71) – H(71)	107.8	C(73) – C(71) – H(71)	107.8
P(12) – C(71) – H(71)	107.8	C(75) – C(72) – C(76)	111.5(4)
C(75) – C(72) – P(12)	109.5(3)	C(76) – C(72) – P(12)	116.3(3)
C(75) – C(72) – H(72)	106.3	C(76) – C(72) – H(72)	106.3
P(12) – C(72) – H(72)	106.3	C(71) – C(73) – H(73A)	109.5
C(71) – C(73) – H(73B)	109.5	H(73A) – C(73) – H(73B)	109.5
C(71) – C(73) – H(73C)	109.5	H(73A) – C(73) – H(73C)	109.5
H(73B) – C(73) – H(73C)	109.5	C(71) – C(74) – H(74A)	109.5
C(71) – C(74) – H(74B)	109.5	H(74A) – C(74) – H(74B)	109.5
C(71) – C(74) – H(74C)	109.5	H(74A) – C(74) – H(74C)	109.5
H(74B) – C(74) – H(74C)	109.5	C(72) – C(75) – H(75A)	109.5
C(72) – C(75) – H(75B)	109.5	H(75A) – C(75) – H(75B)	109.5

Continued on next page

Table S50. – continued from previous page

atom – atom – atom	angle	atom – atom – atom	angle
C(72) – C(75) – H(75C)	109.5	H(75A) – C(75) – H(75C)	109.5
H(75B) – C(75) – H(75C)	109.5	C(72) – C(76) – H(76A)	109.5
C(72) – C(76) – H(76B)	109.5	H(76A) – C(76) – H(76B)	109.5
C(72) – C(76) – H(76C)	109.5	H(76A) – C(76) – H(76C)	109.5
H(76B) – C(76) – H(76C)	109.5	C(80) – C(77) – C(79)	112.7(4)
C(80) – C(77) – P(11)	115.8(3)	C(79) – C(77) – P(11)	108.0(3)
C(80) – C(77) – H(77)	106.6	C(79) – C(77) – H(77)	106.6
P(11) – C(77) – H(77)	106.6	C(82) – C(78) – C(81)	110.3(4)
C(82) – C(78) – P(11)	113.3(3)	C(81) – C(78) – P(11)	110.2(3)
C(82) – C(78) – H(78)	107.6	C(81) – C(78) – H(78)	107.6
P(11) – C(78) – H(78)	107.6	C(77) – C(79) – H(79A)	109.5
C(77) – C(79) – H(79B)	109.5	H(79A) – C(79) – H(79B)	109.5
C(77) – C(79) – H(79C)	109.5	H(79A) – C(79) – H(79C)	109.5
H(79B) – C(79) – H(79C)	109.5	C(77) – C(80) – H(80A)	109.5
C(77) – C(80) – H(80B)	109.5	H(80A) – C(80) – H(80B)	109.5
C(77) – C(80) – H(80C)	109.5	H(80A) – C(80) – H(80C)	109.5
H(80B) – C(80) – H(80C)	109.5	C(78) – C(81) – H(81A)	109.5
C(78) – C(81) – H(81B)	109.5	H(81A) – C(81) – H(81B)	109.5
C(78) – C(81) – H(81C)	109.5	H(81A) – C(81) – H(81C)	109.5
H(81B) – C(81) – H(81C)	109.5	C(78) – C(82) – H(82A)	109.5
C(78) – C(82) – H(82B)	109.5	H(82A) – C(82) – H(82B)	109.5
C(78) – C(82) – H(82C)	109.5	H(82A) – C(82) – H(82C)	109.5
H(82B) – C(82) – H(82C)	109.5	C(86) – C(83) – C(85)	112.4(4)
C(86) – C(83) – P(21)	109.7(3)	C(85) – C(83) – P(21)	115.3(3)
C(86) – C(83) – H(83)	106.3	C(85) – C(83) – H(83)	106.3
P(21) – C(83) – H(83)	106.3	C(87) – C(84) – C(88)	111.1(4)
C(87) – C(84) – P(21)	112.9(3)	C(88) – C(84) – P(21)	110.0(3)
C(87) – C(84) – H(84)	107.5	C(88) – C(84) – H(84)	107.5
P(21) – C(84) – H(84)	107.5	C(83) – C(85) – H(85A)	109.5
C(83) – C(85) – H(85B)	109.5	H(85A) – C(85) – H(85B)	109.5
C(83) – C(85) – H(85C)	109.5	H(85A) – C(85) – H(85C)	109.5
H(85B) – C(85) – H(85C)	109.5	C(83) – C(86) – H(86A)	109.5
C(83) – C(86) – H(86B)	109.5	H(86A) – C(86) – H(86B)	109.5
C(83) – C(86) – H(86C)	109.5	H(86A) – C(86) – H(86C)	109.5
H(86B) – C(86) – H(86C)	109.5	C(84) – C(87) – H(87A)	109.5
C(84) – C(87) – H(87B)	109.5	H(87A) – C(87) – H(87B)	109.5
C(84) – C(87) – H(87C)	109.5	H(87A) – C(87) – H(87C)	109.5
H(87B) – C(87) – H(87C)	109.5	C(84) – C(88) – H(88A)	109.5
C(84) – C(88) – H(88B)	109.5	H(88A) – C(88) – H(88B)	109.5
C(84) – C(88) – H(88C)	109.5	H(88A) – C(88) – H(88C)	109.5
H(88B) – C(88) – H(88C)	109.5	C(91) – C(89) – C(92)	112.9(4)
C(91) – C(89) – P(22)	108.6(4)	C(92) – C(89) – P(22)	111.9(3)

Continued on next page

Table S50. – continued from previous page

atom – atom – atom	angle	atom – atom – atom	angle
C(91) – C(89) – H(89)	107.8	C(92) – C(89) – H(89)	107.8
P(22) – C(89) – H(89)	107.8	C(94) – C(90) – C(93)	111.0(4)
C(94) – C(90) – P(22)	114.6(3)	C(93) – C(90) – P(22)	107.4(3)
C(94) – C(90) – H(90)	107.9	C(93) – C(90) – H(90)	107.9
P(22) – C(90) – H(90)	107.9	C(89) – C(91) – H(91A)	109.5
C(89) – C(91) – H(91B)	109.5	H(91A) – C(91) – H(91B)	109.5
C(89) – C(91) – H(91C)	109.5	H(91A) – C(91) – H(91C)	109.5
H(91B) – C(91) – H(91C)	109.5	C(89) – C(92) – H(92A)	109.5
C(89) – C(92) – H(92B)	109.5	H(92A) – C(92) – H(92B)	109.5
C(89) – C(92) – H(92C)	109.5	H(92A) – C(92) – H(92C)	109.5
H(92B) – C(92) – H(92C)	109.5	C(90) – C(93) – H(93A)	109.5
C(90) – C(93) – H(93B)	109.5	H(93A) – C(93) – H(93B)	109.5
C(90) – C(93) – H(93C)	109.5	H(93A) – C(93) – H(93C)	109.5
H(93B) – C(93) – H(93C)	109.5	C(90) – C(94) – H(94A)	109.5
C(90) – C(94) – H(94B)	109.5	H(94A) – C(94) – H(94B)	109.5
C(90) – C(94) – H(94C)	109.5	H(94A) – C(94) – H(94C)	109.5
H(94B) – C(94) – H(94C)	109.5	C(52) – C(53) – C(54)	119.5(4)
C(52) – C(53) – H(53)	120.2	C(54) – C(53) – H(53)	120.2

8.8 Crystal data for [PC(sp^3)HP]PdI (11)

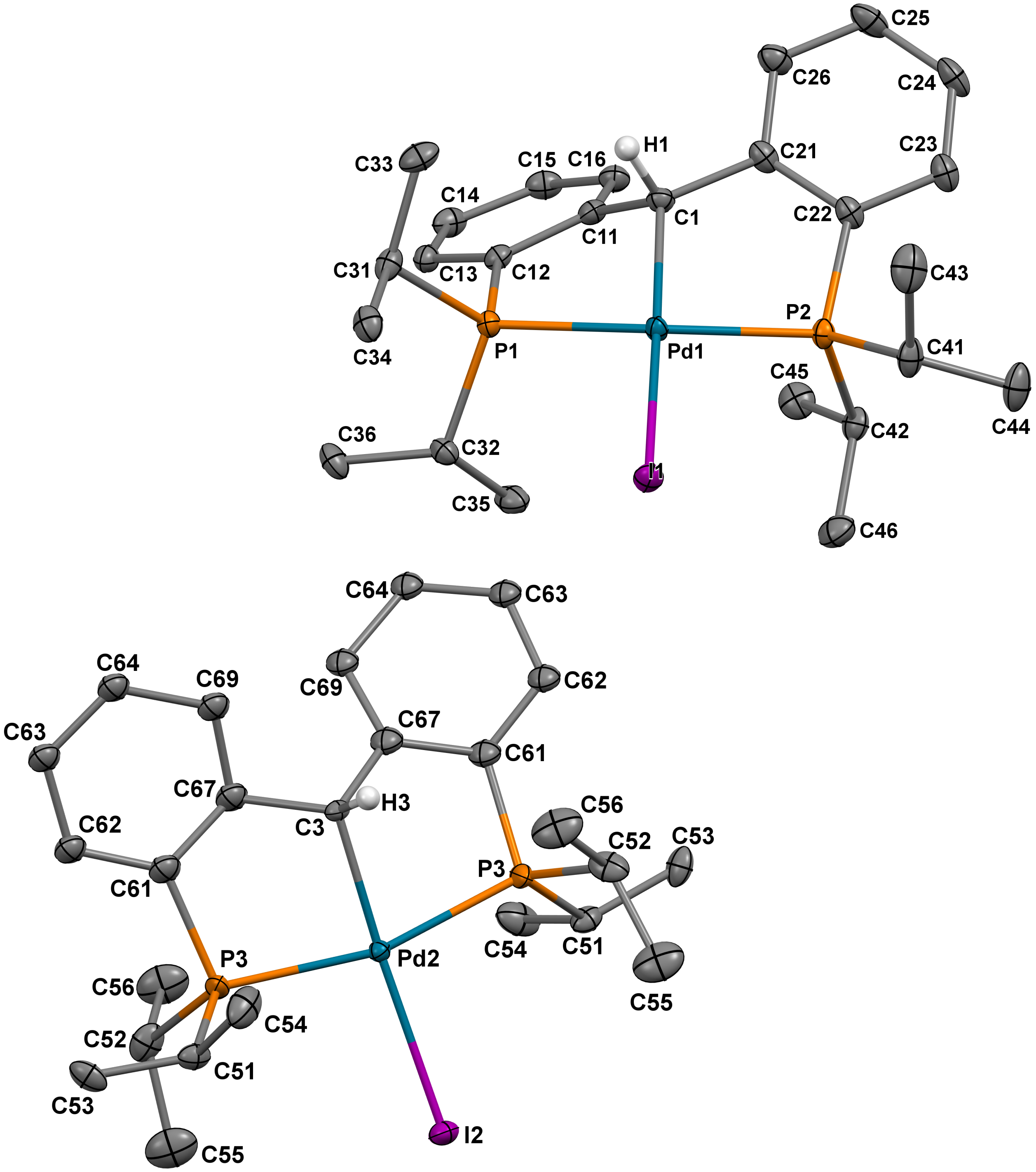

Figure S80. Thermal-ellipsoid representation for the two independent molecules of [PC(sp^3)HP]PdI (**11**) at 50% probability. Most hydrogen atoms were omitted for clarity.

Table S51. Crystal data and structure refinement for [PC(sp^3)HP]PdI (**11**).

Identification code:	cc56	
Empirical formula:	$C_{25}H_{37}IP_2Pd$	
Formula weight:	632.79	
Temperature:	120(2) K	
Wavelength:	0.71073 Å	
Crystal system:	Monoclinic	
Space group:	*C*2/*c*	
Unit cell dimensions:	*a* = 29.176(6) Å	α = 90°
	b = 12.515(2) Å	β = 105.315(4)°
	c = 22.247(4) Å	γ = 90°
Volume:	7835(2) $Å^3$	
Z:	12	
Density (calculated):	1.609 $g{\cdot}cm^{-3}$	
Absorption coefficient (μ):	2.025 mm^{-1}	
F(000):	3792	
Crystal size:	0.02 × 0.02 × 0.01 mm^3	
θ range for data collection:	1.78 to 25.00°	
Index ranges:	$-34 \le h \le 34$, $-14 \le k \le 14$, $-26 \le l \le 26$	
Reflections collected:	55501	
Independent reflections:	6893 [R_{int} = 0.0219]	
Completeness to θ = 25.00°:	100.0 %	
Absorption correction:	Semi-empirical from equivalents	
Max. and min. transmission:	0.7454 and 0.6649	
Refinement method:	Full-matrix least-squares on F^2	
Data / restraints / parameters:	6893 / 0 / 384	
Goodness-of-fit on F^2:	1.080	
Final R indices [I>2σ(I)]:	R_1 = 0.0235, wR_2 = 0.0511	
R indices (all data):	R_1 = 0.0252, wR_2 = 0.0519	
Largest diff. peak and hole:	1.412 and −1.244 $e^{-}{\cdot}Å^{-3}$	

Table S52. Atomic coordinates and equivalent isotropic displacement parameters (Å^2) for [PC(sp^3)HP]PdI (**11**). U(eq) is defined as one third of the trace of the orthogonalized U_{ij} tensor

atom	x	y	z	U(eq)
C(67)	−0.0326(2)	0.2608(5)	0.2713(3)	0.027(1)
C(69)	−0.0431(2)	0.1606(5)	0.2441(3)	0.027(1)
C(66)	−0.0527(2)	0.2590(5)	0.2412(3)	0.027(1)
C(70)	−0.0660(2)	0.1541(5)	0.2216(3)	0.027(1)
C(64)	−0.09194(11)	0.1039(3)	0.24571(14)	0.027(1)
I(1)	0.27189(1)	0.64632(2)	0.36531(1)	0.024(1)
C(1)	0.39437(10)	0.4058(2)	0.46606(13)	0.019(1)
Pd(1)	0.33760(1)	0.50634(2)	0.42675(1)	0.016(1)
P(1)	0.31054(2)	0.35751(6)	0.36599(3)	0.017(1)
I(2)	0.0000	0.69270(2)	0.2500	0.025(1)
Pd(2)	−0.00594(4)	0.47853(3)	0.23895(3)	0.014(1)
P(2)	0.36354(3)	0.59939(6)	0.51720(3)	0.018(1)
P(3)	−0.06005(3)	0.45376(6)	0.29734(4)	0.024(1)
C(3)	−0.01539(19)	0.3167(4)	0.2178(2)	0.016(1)
C(11)	0.37607(10)	0.2939(2)	0.47133(13)	0.020(1)
C(12)	0.34020(10)	0.2541(2)	0.42041(13)	0.019(1)
C(13)	0.32637(10)	0.1472(2)	0.41872(14)	0.022(1)
C(14)	0.34504(11)	0.0804(2)	0.46918(15)	0.027(1)
C(15)	0.37763(11)	0.1208(2)	0.52116(15)	0.026(1)
C(16)	0.39344(10)	0.2258(2)	0.52215(14)	0.023(1)
C(21)	0.43089(10)	0.4491(2)	0.52306(13)	0.022(1)
C(22)	0.42107(10)	0.5405(2)	0.55357(13)	0.021(1)
C(23)	0.45474(11)	0.5803(3)	0.60619(14)	0.027(1)
C(24)	0.49820(11)	0.5299(3)	0.62705(15)	0.032(1)
C(25)	0.50869(11)	0.4411(3)	0.59638(16)	0.033(1)
C(26)	0.47551(11)	0.4011(3)	0.54443(15)	0.028(1)
C(31)	0.32089(11)	0.3272(2)	0.28938(14)	0.024(1)
C(32)	0.24592(10)	0.3413(2)	0.35603(13)	0.021(1)
C(36)	0.22246(11)	0.2461(3)	0.31670(14)	0.028(1)
C(35)	0.23516(11)	0.3420(3)	0.41967(14)	0.029(1)
C(34)	0.29222(11)	0.4026(3)	0.23943(14)	0.028(1)
C(33)	0.37407(12)	0.3354(3)	0.29457(16)	0.033(1)
C(43)	0.40940(14)	0.7730(3)	0.48481(17)	0.041(1)
C(42)	0.32747(11)	0.5722(2)	0.57239(13)	0.025(1)
C(41)	0.36929(12)	0.7455(2)	0.51405(14)	0.027(1)
C(44)	0.37399(14)	0.8065(3)	0.57491(15)	0.038(1)
C(45)	0.32662(12)	0.4521(3)	0.58528(15)	0.031(1)
C(46)	0.27719(12)	0.6163(3)	0.54834(16)	0.035(1)
C(51)	−0.11538(11)	0.5207(2)	0.25533(13)	0.024(1)

Continued on next page

Table S52. – continued from previous page

atom	x	y	x	U(eq)
C(52)	−0.05156(12)	0.4810(3)	0.38051(15)	0.033(1)
C(53)	−0.15841(11)	0.5008(3)	0.28087(15)	0.031(1)
C(54)	−0.12646(15)	0.4913(3)	0.18617(15)	0.043(1)
C(55)	−0.04938(15)	0.6002(3)	0.39489(19)	0.051(1)
C(56)	−0.00661(14)	0.4238(3)	0.41657(19)	0.051(1)
C(61)	−0.07152(11)	0.3109(2)	0.28764(15)	0.027(1)
C(62)	−0.10734(11)	0.2546(2)	0.30357(15)	0.027(1)
C(63)	−0.11705(11)	0.1501(2)	0.28348(14)	0.027(1)
H(69)	−0.0215	0.1262	0.2250	0.033
H(70)	−0.0547	0.1225	0.1894	0.033
H(64)	−0.0951	0.0292	0.2382	0.033
H(1)	0.4121	0.3998	0.4334	0.023
H(3)	−0.0267	0.3173	0.1739	0.019
H(13)	0.3041	0.1199	0.3829	0.026
H(14)	0.3355	0.0077	0.4680	0.032
H(15)	0.3893	0.0763	0.5565	0.031
H(16)	0.4164	0.2516	0.5579	0.028
H(23)	0.4476	0.6415	0.6273	0.033
H(24)	0.5210	0.5566	0.6627	0.038
H(25)	0.5387	0.4072	0.6108	0.040
H(26)	0.4832	0.3405	0.5233	0.034
H(31)	0.3103	0.2523	0.2777	0.029
H(32)	0.2305	0.4067	0.3337	0.026
H(36A)	0.1881	0.2484	0.3116	0.043
H(36B)	0.2354	0.1795	0.3376	0.043
H(36C)	0.2289	0.2494	0.2757	0.043
H(35A)	0.2007	0.3391	0.4139	0.044
H(35B)	0.2478	0.4076	0.4420	0.044
H(35C)	0.2501	0.2798	0.4438	0.044
H(34A)	0.3003	0.3896	0.2000	0.042
H(34B)	0.2998	0.4768	0.2524	0.042
H(34C)	0.2582	0.3899	0.2338	0.042
H(33A)	0.3800	0.3161	0.2546	0.049
H(33B)	0.3915	0.2865	0.3270	0.049
H(33C)	0.3848	0.4088	0.3055	0.049
H(43A)	0.4053	0.7328	0.4459	0.062
H(43B)	0.4399	0.7541	0.5137	0.062
H(43C)	0.4088	0.8497	0.4759	0.062
H(42)	0.3428	0.6090	0.6126	0.030
H(41)	0.3394	0.7717	0.4844	0.033
H(44A)	0.3739	0.8835	0.5668	0.057
H(44B)	0.4038	0.7866	0.6050	0.057

Continued on next page

Table S52. – continued from previous page

atom	x	y	x	U(eq)
H(44C)	0.3472	0.7886	0.5919	0.057
H(45A)	0.3111	0.4145	0.5466	0.047
H(45B)	0.3089	0.4391	0.6164	0.047
H(45C)	0.3592	0.4259	0.6011	0.047
H(46A)	0.2787	0.6935	0.5416	0.052
H(46B)	0.2592	0.6026	0.5791	0.052
H(46C)	0.2614	0.5813	0.5089	0.052
H(51)	−0.1090	0.5993	0.2584	0.028
H(52)	−0.0791	0.4500	0.3934	0.040
H(53A)	−0.1846	0.5474	0.2594	0.047
H(53B)	−0.1499	0.5163	0.3256	0.047
H(53C)	−0.1683	0.4260	0.2740	0.047
H(54A)	−0.1547	0.5304	0.1630	0.064
H(54B)	−0.1324	0.4143	0.1812	0.064
H(54C)	−0.0994	0.5104	0.1700	0.064
H(55A)	−0.0231	0.6325	0.3815	0.077
H(55B)	−0.0442	0.6107	0.4398	0.077
H(55C)	−0.0794	0.6339	0.3726	0.077
H(56A)	−0.0084	0.3483	0.4046	0.077
H(56B)	−0.0036	0.4297	0.4614	0.077
H(56C)	0.0211	0.4568	0.4070	0.077
H(62)	−0.1254	0.2876	0.3283	0.033
H(63)	−0.1409	0.1106	0.2957	0.033

Table S53. Anisotropic displacement parameters (Å^2) for [PC(sp^3)HP]PdI (**11**). The anisotropic displacement factor exponent takes the form: $-2\pi^2[h^2a^{*2}U_{11} + ...+ 2hka^*b^*U_{12}]$.

atom	U_{11}	U_{22}	U_{33}	U_{23}	U_{13}	U_{12}
C(67)	0.0314(8)	0.0218(7)	0.0286(8)	0.0054(6)	0.0073(6)	0.0044(6)
C(69)	0.0314(8)	0.0218(7)	0.0286(8)	0.0054(6)	0.0073(6)	0.0044(6)
C(66)	0.0314(8)	0.0218(7)	0.0286(8)	0.0054(6)	0.0073(6)	0.0044(6)
C(70)	0.0314(8)	0.0218(7)	0.0286(8)	0.0054(6)	0.0073(6)	0.0044(6)
C(64)	0.0314(8)	0.0218(7)	0.0286(8)	0.0054(6)	0.0073(6)	0.0044(6)
I(1)	0.0250(1)	0.0224(1)	0.0209(1)	0.0013(1)	0.0012(1)	0.0077(1)
C(1)	0.0168(13)	0.0186(14)	0.0219(14)	0.0010(11)	0.0033(11)	0.0022(11)
Pd(1)	0.0169(1)	0.0147(1)	0.0151(1)	−0.0004(1)	0.0010(1)	0.0012(1)
P(1)	0.0174(3)	0.0165(3)	0.0172(3)	−0.0013(3)	0.0038(3)	−0.0015(3)
I(2)	0.0290(2)	0.0151(1)	0.0313(2)	0.000	0.0087(1)	0.000
Pd(2)	0.0151(5)	0.0137(2)	0.0144(6)	−0.0003(2)	0.0039(4)	−0.0009(2)
P(2)	0.0227(4)	0.0170(4)	0.0142(3)	0.0005(3)	0.0030(3)	−0.0006(3)
P(3)	0.0224(4)	0.0174(4)	0.0386(5)	0.0059(3)	0.0173(3)	0.0053(3)
C(3)	0.019(3)	0.012(3)	0.015(3)	−0.001(2)	0.003(2)	0.000(2)
C(11)	0.0200(14)	0.0182(14)	0.0221(14)	−0.0006(11)	0.0082(12)	0.0031(11)
C(12)	0.0202(14)	0.0187(14)	0.0194(14)	0.0006(11)	0.0085(11)	0.0017(11)
C(13)	0.0210(14)	0.0192(14)	0.0259(15)	−0.0044(12)	0.0080(12)	−0.0020(12)
C(14)	0.0257(15)	0.0167(14)	0.0402(18)	0.0022(13)	0.0138(14)	−0.0005(12)
C(15)	0.0258(16)	0.0245(16)	0.0295(16)	0.0080(13)	0.0098(13)	0.0048(13)
C(16)	0.0209(14)	0.0237(15)	0.0247(15)	0.0007(12)	0.0038(12)	0.0036(12)
C(21)	0.0219(14)	0.0200(15)	0.0221(15)	0.0044(12)	0.0006(12)	−0.0035(12)
C(22)	0.0223(15)	0.0191(14)	0.0198(14)	0.0039(12)	0.0004(12)	−0.0041(12)
C(23)	0.0312(17)	0.0255(16)	0.0211(15)	0.0004(13)	0.0004(13)	−0.0066(13)
C(24)	0.0263(16)	0.0344(18)	0.0266(16)	0.0044(14)	−0.0077(13)	−0.0099(14)
C(25)	0.0227(16)	0.0343(18)	0.0356(18)	0.0100(15)	−0.0049(14)	−0.0003(14)
C(26)	0.0240(16)	0.0258(16)	0.0327(17)	0.0037(13)	0.0026(13)	0.0015(13)
C(31)	0.0338(17)	0.0190(15)	0.0228(15)	−0.0023(12)	0.0130(13)	−0.0039(13)
C(32)	0.0182(14)	0.0252(15)	0.0190(14)	0.0011(12)	0.0025(11)	−0.0017(12)
C(36)	0.0251(16)	0.0326(17)	0.0253(16)	−0.0001(13)	0.0027(13)	−0.0116(13)
C(35)	0.0221(15)	0.0432(19)	0.0239(16)	0.0001(14)	0.0085(12)	0.0001(14)
C(34)	0.0326(17)	0.0327(17)	0.0199(15)	0.0000(13)	0.0082(13)	−0.0071(14)
C(33)	0.0361(18)	0.0329(18)	0.0364(18)	0.0045(15)	0.0221(15)	0.0063(15)
C(43)	0.063(2)	0.0269(18)	0.0349(19)	0.0042(15)	0.0153(18)	−0.0096(17)
C(42)	0.0326(17)	0.0272(16)	0.0161(14)	−0.0020(12)	0.0079(12)	−0.0010(13)
C(41)	0.0416(18)	0.0155(14)	0.0217(15)	−0.0001(12)	0.0030(13)	−0.0004(13)
C(44)	0.064(2)	0.0196(16)	0.0281(17)	−0.0039(14)	0.0084(16)	−0.0028(16)
C(45)	0.0361(18)	0.0337(18)	0.0255(16)	0.0065(14)	0.0117(14)	−0.0040(14)
C(46)	0.0369(19)	0.040(2)	0.0322(18)	−0.0024(15)	0.0166(15)	0.0060(16)
C(51)	0.0300(16)	0.0200(15)	0.0185(14)	0.0022(12)	0.0018(12)	0.0046(12)
C(52)	0.0280(17)	0.0357(19)	0.0297(17)	0.0130(14)	−0.0021(13)	−0.0003(14)

Continued on next page

Table S53. – continued from previous page

atom	U_{11}	U_{22}	U_{33}	U_{23}	U_{13}	U_{12}
C(53)	0.0219(15)	0.0398(19)	0.0288(17)	−0.0050(14)	0.0018(13)	0.0120(14)
C(54)	0.063(2)	0.043(2)	0.0216(17)	−0.0002(15)	0.0094(16)	−0.0166(19)
C(55)	0.0472(16)	0.0444(16)	0.0484(17)	0.0065(13)	−0.0123(13)	0.0077(13)
C(56)	0.0472(16)	0.0444(16)	0.0484(17)	0.0065(13)	−0.0123(13)	0.0077(13)
C(61)	0.0314(8)	0.0218(7)	0.0286(8)	0.0054(6)	0.0073(6)	0.0044(6)
C(62)	0.0314(8)	0.0218(7)	0.0286(8)	0.0054(6)	0.0073(6)	0.0044(6)
C(63)	0.0314(8)	0.0218(7)	0.0286(8)	0.0054(6)	0.0073(6)	0.0044(6)

Table S54. Distances [Å] for [PC(sp^3)HP]PdI (**11**).

atom – atom	distance	atom – atom	distance
C(67) – C(69)	1.391(9)	C(67) – C(61)	1.427(7)
C(67) – C(3)#1	1.525(8)	C(67) – C(3)	1.572(8)
C(69) – C(64)	1.602(7)	C(69) – H(69)	0.9500
C(66) – C(70)	1.406(9)	C(66) – C(61)	1.446(7)
C(66) – C(3)	1.508(8)	C(70) – C(64)	1.211(7)
C(70) – H(70)	0.9500	C(64) – C(63)	1.379(4)
C(64) – H(64)	0.9500	I(1) – Pd(1)	2.6887(4)
C(1) – C(11)	1.515(4)	C(1) – C(21)	1.524(4)
C(1) – Pd(1)	2.080(3)	C(1) – H(1)	1.0000
Pd(1) – P(2)	2.2744(8)	Pd(1) – P(1)	2.3144(8)
P(1) – C(12)	1.827(3)	P(1) – C(31)	1.847(3)
P(1) – C(32)	1.851(3)	I(2) – Pd(2)	2.6930(6)
I(2) – Pd(2)#1	2.6930(6)	Pd(2) – Pd(2)#1	0.5212(9)
Pd(2) – C(3)	2.082(5)	Pd(2) – C(3)#1	2.259(5)
Pd(2) – P(3)#1	2.2968(15)	Pd(2) – P(3)	2.3151(15)
P(2) – C(22)	1.816(3)	P(2) – C(41)	1.839(3)
P(2) – C(42)	1.848(3)	P(3) – C(61)	1.821(3)
P(3) – C(52)	1.833(4)	P(3) – C(51)	1.840(3)
P(3) – Pd(2)#1	2.2968(15)	C(3) – C(3)#1	1.478(10)
C(3) – C(67)#1	1.525(8)	C(3) – Pd(2)#1	2.259(5)
C(3) – H(3)	0.9440	C(11) – C(16)	1.400(4)
C(11) – C(12)	1.414(4)	C(12) – C(13)	1.395(4)
C(13) – C(14)	1.390(4)	C(13) – H(13)	0.9500
C(14) – C(15)	1.384(5)	C(14) – H(14)	0.9500
C(15) – C(16)	1.391(4)	C(15) – H(15)	0.9500
C(16) – H(16)	0.9500	C(21) – C(22)	1.398(4)
C(21) – C(26)	1.398(4)	C(22) – C(23)	1.406(4)
C(23) – C(24)	1.382(5)	C(23) – H(23)	0.9500
C(24) – C(25)	1.380(5)	C(24) – H(24)	0.9500
C(25) – C(26)	1.390(4)	C(25) – H(25)	0.9500
C(26) – H(26)	0.9500	C(31) – C(34)	1.528(4)
C(31) – C(33)	1.529(4)	C(31) – H(31)	1.0000
C(32) – C(35)	1.529(4)	C(32) – C(36)	1.529(4)
C(32) – H(32)	1.0000	C(36) – H(36A)	0.9800
C(36) – H(36B)	0.9800	C(36) – H(36C)	0.9800
C(35) – H(35A)	0.9800	C(35) – H(35B)	0.9800
C(35) – H(35C)	0.9800	C(34) – H(34A)	0.9800
C(34) – H(34B)	0.9800	C(34) – H(34C)	0.9800
C(33) – H(33A)	0.9800	C(33) – H(33B)	0.9800
C(33) – H(33C)	0.9800	C(43) – C(41)	1.521(5)

Symmetry transformations used to generate equivalent atoms: #1 −x,y,−z+$\frac{1}{2}$

Continued on next page

Table S54. – continued from previous page

atom – atom	distance	atom – atom	distance
C(43) – H(43A)	0.9800	C(43) – H(43B)	0.9800
C(43) – H(43C)	0.9800	C(42) – C(46)	1.526(4)
C(42) – C(45)	1.532(4)	C(42) – H(42)	1.0000
C(41) – C(44)	1.529(4)	C(41) – H(41)	1.0000
C(44) – H(44A)	0.9800	C(44) – H(44B)	0.9800
C(44) – H(44C)	0.9800	C(45) – H(45A)	0.9800
C(45) – H(45B)	0.9800	C(45) – H(45C)	0.9800
C(46) – H(46A)	0.9800	C(46) – H(46B)	0.9800
C(46) – H(46C)	0.9800	C(51) – C(53)	1.529(4)
C(51) – C(54)	1.531(4)	C(51) – H(51)	1.0000
C(52) – C(55)	1.523(5)	C(52) – C(56)	1.523(5)
C(52) – H(52)	1.0000	C(53) – H(53A)	0.9800
C(53) – H(53B)	0.9800	C(53) – H(53C)	0.9800
C(54) – H(54A)	0.9800	C(54) – H(54B)	0.9800
C(54) – H(54C)	0.9800	C(55) – H(55A)	0.9800
C(55) – H(55B)	0.9800	C(55) – H(55C)	0.9800
C(56) – H(56A)	0.9800	C(56) – H(56B)	0.9800
C(56) – H(56C)	0.9800	C(61) – C(62)	1.382(4)
C(62) – C(63)	1.387(4)	C(62) – H(62)	0.9500
C(63) – H(63)	0.9500		

Symmetry transformations used to generate equivalent atoms: #1 $-x,y,-z+\frac{1}{2}$

Table S55. Angles [°] for [PC(sp^3)HP]PdI (**11**).

atom – atom – atom	angle	atom – atom – atom	angle
C(69) – C(67) – C(61)	114.1(6)	C(69) – C(67) – C(3)#1	124.8(6)
C(61) – C(67) – C(3)#1	121.1(5)	C(69) – C(67) – C(3)	98.6(5)
C(61) – C(67) – C(3)	115.6(5)	C(3)#1 – C(67) – C(3)	57.0(4)
C(67) – C(69) – C(64)	119.0(5)	C(67) – C(69) – H(69)	120.5
C(64) – C(69) – H(69)	120.5	C(70) – C(66) – C(61)	120.9(5)
C(70) – C(66) – C(3)	120.4(6)	C(61) – C(66) – C(3)	118.5(5)
C(64) – C(70) – C(66)	120.1(6)	C(64) – C(70) – H(70)	120.0
C(66) – C(70) – H(70)	120.0	C(70) – C(64) – C(63)	123.1(4)
C(63) – C(64) – C(69)	116.5(3)	C(70) – C(64) – H(64)	118.4
C(63) – C(64) – H(64)	118.4	C(69) – C(64) – H(64)	118.6
C(11) – C(1) – C(21)	116.6(2)	C(11) – C(1) – Pd(1)	109.52(18)
C(21) – C(1) – Pd(1)	116.03(19)	C(11) – C(1) – H(1)	104.4
C(21) – C(1) – H(1)	104.4	Pd(1) – C(1) – H(1)	104.4
C(1) – Pd(1) – P(2)	84.02(8)	C(1) – Pd(1) – P(1)	82.51(8)
P(2) – Pd(1) – P(1)	155.68(3)	C(1) – Pd(1) – I(1)	171.75(8)
P(2) – Pd(1) – I(1)	98.17(2)	P(1) – Pd(1) – I(1)	98.05(2)
C(12) – P(1) – C(31)	107.58(13)	C(12) – P(1) – C(32)	106.93(13)
C(31) – P(1) – C(32)	106.07(13)	C(12) – P(1) – Pd(1)	98.88(9)
C(31) – P(1) – Pd(1)	126.19(10)	C(32) – P(1) – Pd(1)	109.73(10)
Pd(2)#1 – Pd(2) – C(3)	103.23(14)	Pd(2)#1 – Pd(2) – C(3)#1	63.79(13)
C(3) – Pd(2) – C(3)#1	39.5(3)	Pd(2)#1 – Pd(2) – P(3)#1	85.5(3)
C(3) – Pd(2) – P(3)#1	82.08(15)	C(3)#1 – Pd(2) – P(3)#1	82.34(14)
Pd(2)#1 – Pd(2) – P(3)	81.5(3)	C(3) – Pd(2) – P(3)	85.84(15)
C(3)#1 – Pd(2) – P(3)	78.03(14)	P(3)#1 – Pd(2) – P(3)	159.77(4)
Pd(2)#1 – Pd(2) – I(2)	84.447(10)	C(3) – Pd(2) – I(2)	172.19(14)
C(3)#1 – Pd(2) – I(2)	148.20(13)	P(3)#1 – Pd(2) – I(2)	97.28(4)
P(3) – Pd(2) – I(2)	96.84(4)	C(22) – P(2) – C(41)	109.72(14)
C(22) – P(2) – C(42)	104.53(14)	C(41) – P(2) – C(42)	106.38(14)
C(22) – P(2) – Pd(1)	103.82(10)	C(41) – P(2) – Pd(1)	119.19(10)
C(42) – P(2) – Pd(1)	112.27(10)	C(61) – P(3) – C(52)	105.90(15)
C(61) – P(3) – C(51)	106.11(14)	C(52) – P(3) – C(51)	107.14(14)
C(61) – P(3) – Pd(2)#1	103.64(11)	C(52) – P(3) – Pd(2)#1	115.31(11)
C(51) – P(3) – Pd(2)#1	117.68(10)	C(61) – P(3) – Pd(2)	101.32(11)
C(52) – P(3) – Pd(2)	127.68(11)	C(51) – P(3) – Pd(2)	106.91(10)
C(3)#1 – C(3) – C(66)	88.5(5)	C(3)#1 – C(3) – C(67)#1	63.1(4)
C(66) – C(3) – C(67)#1	116.1(5)	C(3)#1 – C(3) – C(67)	59.9(4)
C(67)#1 – C(3) – C(67)	97.9(5)	C(3)#1 – C(3) – Pd(2)	76.70(14)
C(66) – C(3) – Pd(2)	116.9(4)	C(67)#1 – C(3) – Pd(2)	110.4(4)
C(67) – C(3) – Pd(2)	108.1(4)	C(3)#1 – C(3) – Pd(2)#1	63.76(13)
C(66) – C(3) – Pd(2)#1	115.1(4)	C(67)#1 – C(3) – Pd(2)#1	101.8(4)

Symmetry transformations used to generate equivalent atoms: #1 $-x,y,-z+\frac{1}{2}$

Continued on next page

Table S55. – continued from previous page

atom – atom – atom	angle	atom – atom – atom	angle
C(67) – C(3) – Pd(2)#1	100.5(3)	C(3)#1 – C(3) – H(3)	163.8
C(66) – C(3) – H(3)	105.9	C(67)#1 – C(3) – H(3)	103.0
C(67) – C(3) – H(3)	133.8	Pd(2) – C(3) – H(3)	102.4
Pd(2)#1 – C(3) – H(3)	114.5	C(16) – C(11) – C(12)	117.8(3)
C(16) – C(11) – C(1)	124.5(3)	C(12) – C(11) – C(1)	117.7(2)
C(13) – C(12) – C(11)	120.5(3)	C(13) – C(12) – P(1)	125.4(2)
C(11) – C(12) – P(1)	113.7(2)	C(14) – C(13) – C(12)	120.4(3)
C(14) – C(13) – H(13)	119.8	C(12) – C(13) – H(13)	119.8
C(15) – C(14) – C(13)	119.4(3)	C(15) – C(14) – H(14)	120.3
C(13) – C(14) – H(14)	120.3	C(14) – C(15) – C(16)	120.7(3)
C(14) – C(15) – H(15)	119.7	C(16) – C(15) – H(15)	119.7
C(15) – C(16) – C(11)	121.0(3)	C(15) – C(16) – H(16)	119.5
C(11) – C(16) – H(16)	119.5	C(22) – C(21) – C(26)	118.6(3)
C(22) – C(21) – C(1)	120.5(3)	C(26) – C(21) – C(1)	120.9(3)
C(21) – C(22) – C(23)	120.4(3)	C(21) – C(22) – P(2)	113.6(2)
C(23) – C(22) – P(2)	126.0(2)	C(24) – C(23) – C(22)	119.7(3)
C(24) – C(23) – H(23)	120.1	C(22) – C(23) – H(23)	120.1
C(25) – C(24) – C(23)	120.3(3)	C(25) – C(24) – H(24)	119.8
C(23) – C(24) – H(24)	119.8	C(24) – C(25) – C(26)	120.3(3)
C(24) – C(25) – H(25)	119.8	C(26) – C(25) – H(25)	119.8
C(25) – C(26) – C(21)	120.6(3)	C(25) – C(26) – H(26)	119.7
C(21) – C(26) – H(26)	119.7	C(34) – C(31) – C(33)	111.3(2)
C(34) – C(31) – P(1)	110.7(2)	C(33) – C(31) – P(1)	109.1(2)
C(34) – C(31) – H(31)	108.6	C(33) – C(31) – H(31)	108.6
P(1) – C(31) – H(31)	108.6	C(35) – C(32) – C(36)	111.3(3)
C(35) – C(32) – P(1)	109.90(19)	C(36) – C(32) – P(1)	116.2(2)
C(35) – C(32) – H(32)	106.2	C(36) – C(32) – H(32)	106.2
P(1) – C(32) – H(32)	106.2	C(32) – C(36) – H(36A)	109.5
C(32) – C(36) – H(36B)	109.5	H(36A) – C(36) – H(36B)	109.5
C(32) – C(36) – H(36C)	109.5	H(36A) – C(36) – H(36C)	109.5
H(36B) – C(36) – H(36C)	109.5	C(32) – C(35) – H(35A)	109.5
C(32) – C(35) – H(35B)	109.5	H(35A) – C(35) – H(35B)	109.5
C(32) – C(35) – H(35C)	109.5	H(35A) – C(35) – H(35C)	109.5
H(35B) – C(35) – H(35C)	109.5	C(31) – C(34) – H(34A)	109.5
C(31) – C(34) – H(34B)	109.5	H(34A) – C(34) – H(34B)	109.5
C(31) – C(34) – H(34C)	109.5	H(34A) – C(34) – H(34C)	109.5
H(34B) – C(34) – H(34C)	109.5	C(31) – C(33) – H(33A)	109.5
C(31) – C(33) – H(33B)	109.5	H(33A) – C(33) – H(33B)	109.5
C(31) – C(33) – H(33C)	109.5	H(33A) – C(33) – H(33C)	109.5
H(33B) – C(33) – H(33C)	109.5	C(41) – C(43) – H(43A)	109.5

Symmetry transformations used to generate equivalent atoms: #1 $-x,y,-z+\frac{1}{2}$

Continued on next page

Table S55. – continued from previous page

atom – atom – atom	angle	atom – atom – atom	angle
C(41) – C(43) – H(43B)	109.5	H(43A) – C(43) – H(43B)	109.5
C(41) – C(43) – H(43C)	109.5	H(43A) – C(43) – H(43C)	109.5
H(43B) – C(43) – H(43C)	109.5	C(46) – C(42) – C(45)	111.0(3)
C(46) – C(42) – P(2)	111.3(2)	C(45) – C(42) – P(2)	110.2(2)
C(46) – C(42) – H(42)	108.0	C(45) – C(42) – H(42)	108.0
P(2) – C(42) – H(42)	108.0	C(43) – C(41) – C(44)	111.5(3)
C(43) – C(41) – P(2)	109.2(2)	C(44) – C(41) – P(2)	116.8(2)
C(43) – C(41) – H(41)	106.2	C(44) – C(41) – H(41)	106.2
P(2) – C(41) – H(41)	106.2	C(41) – C(44) – H(44A)	109.5
C(41) – C(44) – H(44B)	109.5	H(44A) – C(44) – H(44B)	109.5
C(41) – C(44) – H(44C)	109.5	H(44A) – C(44) – H(44C)	109.5
H(44B) – C(44) – H(44C)	109.5	C(42) – C(45) – H(45A)	109.5
C(42) – C(45) – H(45B)	109.5	H(45A) – C(45) – H(45B)	109.5
C(42) – C(45) – H(45C)	109.5	H(45A) – C(45) – H(45C)	109.5
H(45B) – C(45) – H(45C)	109.5	C(42) – C(46) – H(46A)	109.5
C(42) – C(46) – H(46B)	109.5	H(46A) – C(46) – H(46B)	109.5
C(42) – C(46) – H(46C)	109.5	H(46A) – C(46) – H(46C)	109.5
H(46B) – C(46) – H(46C)	109.5	C(53) – C(51) – C(54)	111.0(3)
C(53) – C(51) – P(3)	115.7(2)	C(54) – C(51) – P(3)	109.0(2)
C(53) – C(51) – H(51)	106.9	C(54) – C(51) – H(51)	106.9
P(3) – C(51) – H(51)	106.9	C(55) – C(52) – C(56)	111.5(3)
C(55) – C(52) – P(3)	112.4(2)	C(56) – C(52) – P(3)	108.2(3)
C(55) – C(52) – H(52)	108.2	C(56) – C(52) – H(52)	108.2
P(3) – C(52) – H(52)	108.2	C(51) – C(53) – H(53A)	109.5
C(51) – C(53) – H(53B)	109.5	H(53A) – C(53) – H(53B)	109.5
C(51) – C(53) – H(53C)	109.5	H(53A) – C(53) – H(53C)	109.5
H(53B) – C(53) – H(53C)	109.5	C(51) – C(54) – H(54A)	109.5
C(51) – C(54) – H(54B)	109.5	H(54A) – C(54) – H(54B)	109.5
C(51) – C(54) – H(54C)	109.5	H(54A) – C(54) – H(54C)	109.5
H(54B) – C(54) – H(54C)	109.5	C(52) – C(55) – H(55A)	109.5
C(52) – C(55) – H(55B)	109.5	H(55A) – C(55) – H(55B)	109.5
C(52) – C(55) – H(55C)	109.5	H(55A) – C(55) – H(55C)	109.5
H(55B) – C(55) – H(55C)	109.5	C(52) – C(56) – H(56A)	109.5
C(52) – C(56) – H(56B)	109.5	H(56A) – C(56) – H(56B)	109.5
C(52) – C(56) – H(56C)	109.5	H(56A) – C(56) – H(56C)	109.5
H(56B) – C(56) – H(56C)	109.5	C(62) – C(61) – C(67)	123.3(4)
C(62) – C(61) – C(66)	114.2(4)	C(62) – C(61) – P(3)	126.7(2)
C(67) – C(61) – P(3)	109.1(3)	C(66) – C(61) – P(3)	115.6(3)
C(61) – C(62) – C(63)	120.1(3)	C(61) – C(62) – H(62)	119.9
C(64) – C(63) – H(63)	120.1	C(62) – C(63) – H(63)	120.1

Symmetry transformations used to generate equivalent atoms: #1 $-x,y,-z+\frac{1}{2}$

8.9 Crystal data for $[PC(sp^3)H_2P]PdBr_2 \cdot CH_2Cl_2$ ($12 \cdot CH_2Cl_2$)

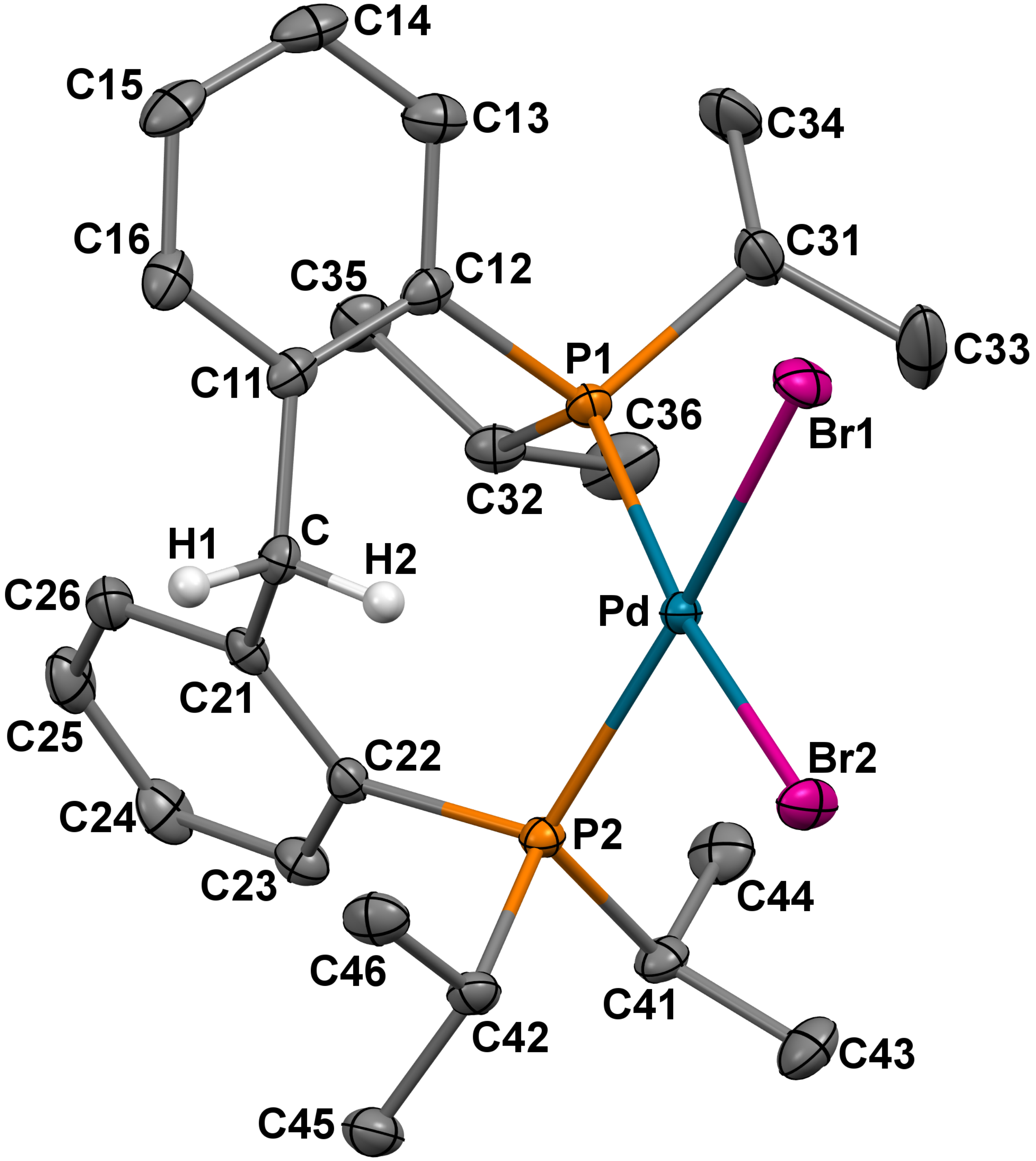

Figure S81. Thermal-ellipsoid representation of $[PC(sp^3)H_2P]PdBr_2 \cdot CH_2Cl_2$ (**$12 \cdot CH_2Cl_2$**) at 50% probability. Most hydrogen atoms and the solvent were omitted for clarity.

Table S56. Crystal data and structure refinement for $[PC(sp^3)H_2P]PdBr_2 \cdot CH_2Cl_2$ (**12·CH₂Cl₂**).

Identification code:	cc298	
Empirical formula:	$C_{51}H_{78}Br_4Cl_2P_4Pd_2$	
Formula weight:	1418.35	
Temperature:	120(2) K	
Wavelength:	0.71073 Å	
Crystal system:	Monoclinic	
Space group:	$P2_1/n$	
Unit cell dimensions:	a = 10.3876(9) Å	α = 90°
	b = 16.2993(14) Å	β = 103.4770(14)°
	c = 17.0895(15) Å	γ = 90°
Volume:	2813.8(4) Å^3	
Z:	2	
Density (calculated):	1.674 g·cm^{-3}	
Absorption coefficient (μ):	3.721 mm^{-1}	
F(000):	1420	
Crystal size:	0.12 × 0.10 × 0.09 mm^3	
θ range for data collection:	1.75 to 25.00°	
Index ranges:	$-12 \le h \le 12$, $-19 \le k \le 19$, $-20 \le l \le 20$	
Reflections collected:	50587	
Independent reflections:	4956 [R_{int} = 0.0399]	
Completeness to θ = 25.00°:	100.0 %	
Absorption correction:	Semi-empirical from equivalents	
Max. and min. transmission:	0.7458 and 0.5977	
Refinement method:	Full-matrix least-squares on F^2	
Data / restraints / parameters:	4956 / 0 / 307	
Goodness-of-fit on F^2:	1.031	
Final R indices [I>2σ(I)]:	R_1 = 0.0185, wR_2 = 0.0423	
R indices (all data):	R_1 = 0.0239, wR_2 = 0.0435	
Largest diff. peak and hole:	0.371 and −0.325 e^-·Å^{-3}	

Table S57. Atomic coordinates and equivalent isotropic displacement parameters (Å^2) for [PC(sp^3)H$_2$P]PdBr$_2$·CH$_2$Cl$_2$ (**12·CH$_2$Cl$_2$**). U(eq) is defined as one third of the trace of the orthogonalized U$_{ij}$ tensor

**atom**	**x**	**y**	**z**	**U(eq)**
Pd	0.39516(2)	0.40354(1)	0.72121(1)	0.012(1)
Br(1)	0.49003(2)	0.54512(1)	0.73904(2)	0.022(1)
P(1)	0.61024(5)	0.35928(3)	0.72070(3)	0.015(1)
P(2)	0.27881(5)	0.28512(3)	0.68228(3)	0.013(1)
Br(2)	0.18270(2)	0.46667(1)	0.73201(2)	0.023(1)
C	0.4226(2)	0.34341(13)	0.54284(13)	0.015(1)
C(11)	0.5573(2)	0.38260(13)	0.55160(13)	0.016(1)
C(12)	0.6446(2)	0.39761(13)	0.62652(13)	0.016(1)
C(13)	0.7599(2)	0.44243(14)	0.62756(15)	0.022(1)
C(14)	0.7928(2)	0.46752(14)	0.55758(16)	0.026(1)
C(15)	0.7099(2)	0.44941(14)	0.48379(15)	0.024(1)
C(16)	0.5924(2)	0.40860(13)	0.48176(14)	0.020(1)
C(21)	0.4219(2)	0.25286(13)	0.56285(12)	0.014(1)
C(22)	0.3565(2)	0.21949(13)	0.61934(12)	0.014(1)
C(23)	0.3576(2)	0.13442(13)	0.63028(13)	0.019(1)
C(24)	0.4260(2)	0.08330(14)	0.58972(14)	0.023(1)
C(25)	0.4951(2)	0.11613(14)	0.53724(14)	0.024(1)
C(26)	0.4905(2)	0.19980(14)	0.52280(13)	0.020(1)
C(31)	0.7298(2)	0.41123(16)	0.80335(14)	0.026(1)
C(32)	0.6620(2)	0.24818(14)	0.72453(13)	0.019(1)
C(33)	0.6844(3)	0.4066(2)	0.88244(16)	0.042(1)
C(5)	0.4278(15)	0.5135(6)	0.9606(9)	0.043(3)
Cl(52)	0.34493(16)	0.41964(9)	0.96465(9)	0.045(1)
Cl(51)	0.5775(3)	0.51970(16)	1.0320(2)	0.045(1)
C(34)	0.8769(2)	0.38661(17)	0.81821(16)	0.031(1)
C(35)	0.7672(2)	0.23084(14)	0.67644(14)	0.023(1)
C(36)	0.7074(2)	0.21172(17)	0.80940(15)	0.032(1)
C(41)	0.2548(2)	0.22043(13)	0.76663(13)	0.018(1)
C(42)	0.1085(2)	0.29878(13)	0.61879(13)	0.017(1)
C(43)	0.1622(2)	0.26151(15)	0.81267(15)	0.027(1)
C(44)	0.3858(2)	0.19839(15)	0.82388(14)	0.026(1)
C(45)	0.0426(2)	0.21639(14)	0.59144(15)	0.024(1)
C(46)	0.1027(2)	0.35380(15)	0.54563(14)	0.023(1)
H(1)	0.3720	0.3511	0.4866	0.018
H(2)	0.3751	0.3733	0.5780	0.018
H(13)	0.8172	0.4560	0.6778	0.027
H(14)	0.8724	0.4972	0.5601	0.032
H(15)	0.7332	0.4648	0.4353	0.029
H(16)	0.5338	0.3980	0.4312	0.024

Continued on next page

Table S57. – continued from previous page

atom	x	y	x	U(eq)
H(23)	0.3104	0.1113	0.6663	0.022
H(24)	0.4255	0.0257	0.5979	0.028
H(25)	0.5456	0.0815	0.5111	0.029
H(26)	0.5351	0.2216	0.4848	0.024
H(31)	0.7263	0.4707	0.7885	0.031
H(32)	0.5815	0.2165	0.6974	0.022
H(33A)	0.5899	0.4198	0.8722	0.064
H(33B)	0.7349	0.4460	0.9210	0.064
H(33C)	0.6994	0.3511	0.9047	0.064
H(5A)	0.4447	0.5201	0.9063	0.052
H(5B)	0.3698	0.5591	0.9693	0.052
H(34A)	0.9029	0.3840	0.7667	0.046
H(34B)	0.8898	0.3327	0.8443	0.046
H(34C)	0.9314	0.4274	0.8530	0.046
H(35A)	0.7329	0.2471	0.6202	0.034
H(35B)	0.7881	0.1721	0.6790	0.034
H(35C)	0.8475	0.2623	0.6995	0.034
H(36A)	0.7010	0.1518	0.8063	0.049
H(36B)	0.6507	0.2325	0.8435	0.049
H(36C)	0.7994	0.2276	0.8325	0.049
H(41)	0.2120	0.1682	0.7433	0.021
H(42)	0.0548	0.3260	0.6529	0.021
H(43A)	0.0750	0.2697	0.7764	0.041
H(43B)	0.1991	0.3147	0.8334	0.041
H(43C)	0.1533	0.2264	0.8577	0.041
H(44A)	0.4423	0.1697	0.7942	0.038
H(44B)	0.3692	0.1627	0.8666	0.038
H(44C)	0.4302	0.2486	0.8477	0.038
H(45A)	−0.0495	0.2256	0.5627	0.036
H(45B)	0.0449	0.1816	0.6385	0.036
H(45C)	0.0903	0.1892	0.5555	0.036
H(46A)	0.1493	0.3271	0.5088	0.035
H(46B)	0.1450	0.4065	0.5633	0.035
H(46C)	0.0101	0.3631	0.5179	0.035

Table S58. Anisotropic displacement parameters (Å^2) for [PC(sp^3)H_2P]PdBr_2·CH_2Cl_2 (**12·CH_2Cl_2**). The anisotropic displacement factor exponent takes the form: $-2\pi^2[h^2a^{*2}U_{11} + ... + 2hka^*b^*U_{12}]$.

atom	U_{11}	U_{22}	U_{33}	U_{23}	U_{13}	U_{12}
Pd	0.0121(1)	0.0110(1)	0.0149(1)	−0.0023(1)	0.0049(1)	−0.0004(1)
Br(1)	0.0212(1)	0.0135(1)	0.0330(1)	−0.0074(1)	0.0082(1)	−0.0041(1)
P(1)	0.0120(3)	0.0182(3)	0.0146(3)	−0.0019(2)	0.0034(2)	0.0003(2)
P(2)	0.0133(3)	0.0115(3)	0.0153(3)	0.0000(2)	0.0050(2)	−0.0009(2)
Br(2)	0.0177(1)	0.0164(1)	0.0373(1)	−0.0073(1)	0.0125(1)	0.0012(1)
C	0.0165(11)	0.0164(11)	0.0120(11)	0.0004(9)	0.0039(9)	0.0005(9)
C(11)	0.0182(11)	0.0102(11)	0.0216(12)	0.0010(9)	0.0092(9)	0.0032(9)
C(12)	0.0158(11)	0.0131(11)	0.0214(12)	0.0000(9)	0.0081(9)	0.0020(9)
C(13)	0.0180(12)	0.0180(12)	0.0315(14)	−0.0004(10)	0.0065(10)	−0.0012(9)
C(14)	0.0216(13)	0.0198(13)	0.0425(16)	0.0060(11)	0.0162(12)	−0.0012(10)
C(15)	0.0297(14)	0.0176(12)	0.0312(14)	0.0073(10)	0.0179(12)	0.0015(10)
C(16)	0.0282(13)	0.0145(12)	0.0207(12)	0.0014(9)	0.0105(10)	0.0028(10)
C(21)	0.0128(11)	0.0150(11)	0.0131(11)	−0.0011(9)	−0.0011(9)	0.0000(9)
C(22)	0.0123(11)	0.0147(11)	0.0145(11)	−0.0028(9)	0.0013(9)	0.0007(9)
C(23)	0.0189(12)	0.0152(11)	0.0205(12)	0.0001(9)	0.0029(9)	−0.0023(9)
C(24)	0.0265(13)	0.0114(12)	0.0292(13)	−0.0045(10)	0.0022(11)	0.0036(10)
C(25)	0.0247(13)	0.0205(13)	0.0268(13)	−0.0101(10)	0.0052(11)	0.0043(10)
C(26)	0.0196(12)	0.0221(13)	0.0177(12)	−0.0050(9)	0.0046(10)	−0.0012(10)
C(31)	0.0177(12)	0.0353(15)	0.0236(13)	−0.0129(11)	0.0015(10)	−0.0005(10)
C(32)	0.0143(11)	0.0185(12)	0.0221(12)	0.0030(9)	0.0028(9)	0.0013(9)
C(33)	0.0228(14)	0.079(2)	0.0232(14)	−0.0211(14)	0.0000(11)	0.0099(14)
C(5)	0.054(5)	0.037(6)	0.044(5)	−0.004(6)	0.024(4)	0.007(6)
Cl(52)	0.0599(10)	0.0248(7)	0.0433(9)	0.0039(6)	−0.0011(7)	0.0090(7)
Cl(51)	0.0369(11)	0.0539(19)	0.0465(13)	−0.0100(15)	0.0167(8)	0.0107(15)
C(34)	0.0150(12)	0.0429(16)	0.0318(15)	−0.0126(12)	−0.0010(11)	−0.0035(11)
C(35)	0.0175(12)	0.0220(13)	0.0285(13)	−0.0015(10)	0.0053(10)	0.0060(10)
C(36)	0.0240(14)	0.0418(16)	0.0308(15)	0.0137(12)	0.0045(11)	0.0064(12)
C(41)	0.0195(12)	0.0159(12)	0.0201(12)	0.0024(9)	0.0084(10)	−0.0025(9)
C(42)	0.0119(11)	0.0199(12)	0.0209(12)	−0.0014(9)	0.0045(9)	−0.0006(9)
C(43)	0.0311(14)	0.0290(14)	0.0263(14)	0.0047(11)	0.0162(11)	−0.0003(11)
C(44)	0.0278(13)	0.0264(14)	0.0225(13)	0.0087(10)	0.0053(11)	0.0002(10)
C(45)	0.0180(12)	0.0237(13)	0.0291(13)	−0.0036(10)	0.0050(10)	−0.0046(10)
C(46)	0.0164(12)	0.0265(13)	0.0247(13)	0.0046(10)	0.0010(10)	0.0002(10)

Table S59. Distances [Å] for [PC(sp^3)H_2P]PdBr_2·CH_2Cl_2 (**12·CH_2Cl_2**).

**atom – atom**	**distance**	**atom – atom**	**distance**
Pd – P(2)	2.2922(6)	Pd – P(1)	2.3497(6)
Pd – Br(2)	2.4806(3)	Pd – Br(1)	2.4999(3)
P(1) – C(12)	1.837(2)	P(1) – C(31)	1.854(2)
P(1) – C(32)	1.886(2)	P(2) – C(22)	1.832(2)
P(2) – C(41)	1.849(2)	P(2) – C(42)	1.861(2)
C – C(11)	1.513(3)	C – C(21)	1.515(3)
C – H(1)	0.9900	C – H(2)	0.9900
C(11) – C(16)	1.392(3)	C(11) – C(12)	1.407(3)
C(12) – C(13)	1.400(3)	C(13) – C(14)	1.380(3)
C(13) – H(13)	0.9500	C(14) – C(15)	1.383(4)
C(14) – H(14)	0.9500	C(15) – C(16)	1.383(3)
C(15) – H(15)	0.9500	C(16) – H(16)	0.9500
C(21) – C(26)	1.397(3)	C(21) – C(22)	1.412(3)
C(22) – C(23)	1.399(3)	C(23) – C(24)	1.382(3)
C(23) – H(23)	0.9500	C(24) – C(25)	1.380(3)
C(24) – H(24)	0.9500	C(25) – C(26)	1.385(3)
C(25) – H(25)	0.9500	C(26) – H(26)	0.9500
C(31) – C(33)	1.534(3)	C(31) – C(34)	1.542(3)
C(31) – H(31)	1.0000	C(32) – C(36)	1.536(3)
C(32) – C(35)	1.539(3)	C(32) – H(32)	1.0000
C(33) – H(33A)	0.9800	C(33) – H(33B)	0.9800
C(33) – H(33C)	0.9800	C(5) – Cl(51)	1.741(12)
C(5) – Cl(52)	1.764(11)	C(5) – H(5A)	0.9900
C(5) – H(5B)	0.9900	C(34) – H(34A)	0.9800
C(34) – H(34B)	0.9800	C(34) – H(34C)	0.9800
C(35) – H(35A)	0.9800	C(35) – H(35B)	0.9800
C(35) – H(35C)	0.9800	C(36) – H(36A)	0.9800
C(36) – H(36B)	0.9800	C(36) – H(36C)	0.9800
C(41) – C(44)	1.522(3)	C(41) – C(43)	1.532(3)
C(41) – H(41)	1.0000	C(42) – C(46)	1.528(3)
C(42) – C(45)	1.530(3)	C(42) – H(42)	1.0000
C(43) – H(43A)	0.9800	C(43) – H(43B)	0.9800
C(43) – H(43C)	0.9800	C(44) – H(44A)	0.9800
C(44) – H(44B)	0.9800	C(44) – H(44C)	0.9800
C(45) – H(45A)	0.9800	C(45) – H(45B)	0.9800
C(45) – H(45C)	0.9800	C(46) – H(46A)	0.9800
C(46) – H(46B)	0.9800	C(46) – H(46C)	0.9800

Table S60. Angles [°] for $[PC(sp^3)H_2P]PdBr_2 \cdot CH_2Cl_2$ (**12·CH$_2$Cl$_2$**).

atom – atom – atom	angle	atom – atom – atom	angle
P(2) – Pd – P(1)	100.25(2)	P(2) – Pd – Br(2)	87.297(16)
P(1) – Pd – Br(2)	172.235(16)	P(2) – Pd – Br(1)	168.212(17)
P(1) – Pd – Br(1)	86.311(16)	Br(2) – Pd – Br(1)	86.579(10)
C(12) – P(1) – C(31)	106.23(11)	C(12) – P(1) – C(32)	104.17(10)
C(31) – P(1) – C(32)	106.08(11)	C(12) – P(1) – Pd	106.13(7)
C(31) – P(1) – Pd	109.02(8)	C(32) – P(1) – Pd	123.94(7)
C(22) – P(2) – C(41)	106.41(10)	C(22) – P(2) – C(42)	102.51(10)
C(41) – P(2) – C(42)	103.89(10)	C(22) – P(2) – Pd	112.80(7)
C(41) – P(2) – Pd	114.31(8)	C(42) – P(2) – Pd	115.71(7)
C(11) – C – C(21)	116.24(17)	C(11) – C – H(1)	108.2
C(21) – C – H(1)	108.2	C(11) – C – H(2)	108.2
C(21) – C – H(2)	108.2	H(1) – C – H(2)	107.4
C(16) – C(11) – C(12)	119.0(2)	C(16) – C(11) – C	117.6(2)
C(12) – C(11) – C	123.24(19)	C(13) – C(12) – C(11)	118.1(2)
C(13) – C(12) – P(1)	120.45(17)	C(11) – C(12) – P(1)	121.49(16)
C(14) – C(13) – C(12)	121.8(2)	C(14) – C(13) – H(13)	119.1
C(12) – C(13) – H(13)	119.1	C(13) – C(14) – C(15)	120.0(2)
C(13) – C(14) – H(14)	120.0	C(15) – C(14) – H(14)	120.0
C(16) – C(15) – C(14)	118.9(2)	C(16) – C(15) – H(15)	120.5
C(14) – C(15) – H(15)	120.5	C(15) – C(16) – C(11)	122.0(2)
C(15) – C(16) – H(16)	119.0	C(11) – C(16) – H(16)	119.0
C(26) – C(21) – C(22)	118.56(19)	C(26) – C(21) – C	117.52(19)
C(22) – C(21) – C	123.92(18)	C(23) – C(22) – C(21)	118.86(19)
C(23) – C(22) – P(2)	119.44(16)	C(21) – C(22) – P(2)	121.61(16)
C(24) – C(23) – C(22)	121.3(2)	C(24) – C(23) – H(23)	119.3
C(22) – C(23) – H(23)	119.3	C(25) – C(24) – C(23)	119.9(2)
C(25) – C(24) – H(24)	120.1	C(23) – C(24) – H(24)	120.1
C(24) – C(25) – C(26)	119.8(2)	C(24) – C(25) – H(25)	120.1
C(26) – C(25) – H(25)	120.1	C(25) – C(26) – C(21)	121.5(2)
C(25) – C(26) – H(26)	119.3	C(21) – C(26) – H(26)	119.3
C(33) – C(31) – C(34)	109.8(2)	C(33) – C(31) – P(1)	111.08(17)
C(34) – C(31) – P(1)	117.95(17)	C(33) – C(31) – H(31)	105.7
C(34) – C(31) – H(31)	105.7	P(1) – C(31) – H(31)	105.7
C(36) – C(32) – C(35)	109.48(19)	C(36) – C(32) – P(1)	115.25(17)
C(35) – C(32) – P(1)	112.96(15)	C(36) – C(32) – H(32)	106.2
C(35) – C(32) – H(32)	106.2	P(1) – C(32) – H(32)	106.2
C(31) – C(33) – H(33A)	109.5	C(31) – C(33) – H(33B)	109.5
H(33A) – C(33) – H(33B)	109.5	C(31) – C(33) – H(33C)	109.5
H(33A) – C(33) – H(33C)	109.5	H(33B) – C(33) – H(33C)	109.5
Cl(51) – C(5) – Cl(52)	112.8(6)	Cl(51) – C(5) – H(5A)	109.0
Cl(52) – C(5) – H(5A)	109.0	Cl(51) – C(5) – H(5B)	109.0

Continued on next page

Table S60. – continued from previous page

atom – atom – atom	angle	atom – atom – atom	angle
Cl(52) – C(5) – H(5B)	109.0	H(5A) – C(5) – H(5B)	107.8
C(31) – C(34) – H(34A)	109.5	C(31) – C(34) – H(34B)	109.5
H(34A) – C(34) – H(34B)	109.5	C(31) – C(34) – H(34C)	109.5
H(34A) – C(34) – H(34C)	109.5	H(34B) – C(34) – H(34C)	109.5
C(32) – C(35) – H(35A)	109.5	C(32) – C(35) – H(35B)	109.5
H(35A) – C(35) – H(35B)	109.5	C(32) – C(35) – H(35C)	109.5
H(35A) – C(35) – H(35C)	109.5	H(35B) – C(35) – H(35C)	109.5
C(32) – C(36) – H(36A)	109.5	C(32) – C(36) – H(36B)	109.5
H(36A) – C(36) – H(36B)	109.5	C(32) – C(36) – H(36C)	109.5
H(36A) – C(36) – H(36C)	109.5	H(36B) – C(36) – H(36C)	109.5
C(44) – C(41) – C(43)	110.33(19)	C(44) – C(41) – P(2)	111.89(15)
C(43) – C(41) – P(2)	111.48(15)	C(44) – C(41) – H(41)	107.6
C(43) – C(41) – H(41)	107.6	P(2) – C(41) – H(41)	107.6
C(46) – C(42) – C(45)	109.61(19)	C(46) – C(42) – P(2)	113.44(15)
C(45) – C(42) – P(2)	111.70(15)	C(46) – C(42) – H(42)	107.3
C(45) – C(42) – H(42)	107.3	P(2) – C(42) – H(42)	107.3
C(41) – C(43) – H(43A)	109.5	C(41) – C(43) – H(43B)	109.5
H(43A) – C(43) – H(43B)	109.5	C(41) – C(43) – H(43C)	109.5
H(43A) – C(43) – H(43C)	109.5	H(43B) – C(43) – H(43C)	109.5
C(41) – C(44) – H(44A)	109.5	C(41) – C(44) – H(44B)	109.5
H(44A) – C(44) – H(44B)	109.5	C(41) – C(44) – H(44C)	109.5
H(44A) – C(44) – H(44C)	109.5	H(44B) – C(44) – H(44C)	109.5
C(42) – C(45) – H(45A)	109.5	C(42) – C(45) – H(45B)	109.5
H(45A) – C(45) – H(45B)	109.5	C(42) – C(45) – H(45C)	109.5
H(45A) – C(45) – H(45C)	109.5	H(45B) – C(45) – H(45C)	109.5
C(42) – C(46) – H(46A)	109.5	C(42) – C(46) – H(46B)	109.5
H(46A) – C(46) – H(46B)	109.5	C(42) – C(46) – H(46C)	109.5
H(46A) – C(46) – H(46C)	109.5	H(46B) – C(46) – H(46C)	109.5
